# Non‐parametric combination and related permutation tests for neuroimaging

**DOI:** 10.1002/hbm.23115

**Published:** 2016-02-05

**Authors:** Anderson M. Winkler, Matthew A. Webster, Jonathan C. Brooks, Irene Tracey, Stephen M. Smith, Thomas E. Nichols

**Affiliations:** ^1^ Oxford Centre for Functional MRI of the Brain University of Oxford Oxford United Kingdom; ^2^ Clinical Research and Imaging Centre, University of Bristol Bristol United Kingdom; ^3^ Department of Statistics & Warwick Manufacturing Group University of Warwick Coventry United Kingdom

**Keywords:** permutation tests, non‐parametric combination, multiple testing, conjunctions, general linear model

## Abstract

In this work, we show how permutation methods can be applied to combination analyses such as those that include multiple imaging modalities, multiple data acquisitions of the same modality, or simply multiple hypotheses on the same data. Using the well‐known definition of union‐intersection tests and closed testing procedures, we use synchronized permutations to correct for such multiplicity of tests, allowing flexibility to integrate imaging data with different spatial resolutions, surface and/or volume‐based representations of the brain, including non‐imaging data. For the problem of joint inference, we propose and evaluate a modification of the recently introduced non‐parametric combination (NPC) methodology, such that instead of a two‐phase algorithm and large data storage requirements, the inference can be performed in a single phase, with reasonable computational demands. The method compares favorably to classical multivariate tests (such as MANCOVA), even when the latter is assessed using permutations. We also evaluate, in the context of permutation tests, various combining methods that have been proposed in the past decades, and identify those that provide the best control over error rate and power across a range of situations. We show that one of these, the method of Tippett, provides a link between correction for the multiplicity of tests and their combination. Finally, we discuss how the correction can solve certain problems of multiple comparisons in one‐way ANOVA designs, and how the combination is distinguished from conjunctions, even though both can be assessed using permutation tests. We also provide a common algorithm that accommodates combination and correction. *Hum Brain Mapp 37:1486‐1511, 2016*. © 2016 Wiley Periodicals, Inc.

**Abbreviations**
ANOVA: analysis of variance
CCA: canonical correlation analysis
CVA: canonical variates analysis
CMV: classical multivariate test (e.g. manova, cca);
CTP: closed testing procedure
DTI: diffusion tensor imaging
DTP: dual truncated product
EE: exchangeable errors
EEG: electroencephalography
FA: fractional anisotropy
FMRI: functional magnetic resonance imaging
FDR: false discovery rate
FWER: familywise error rate
GLM: general linear model
ICA: independent component analysis
ISE: independent and symmetric errors
IQ: intelligence quotient
IUT: intersection–union test
JNH: joint null hypothesis
LSD: least significant difference
MANOVA: multivariate analysis of variance
MANCOVA: multivariate analysis of covariance
MD: mean diffusivity
MRI: magnetic resonance imaging
MTP‐I: multiple testing problem I
MTP‐II: multiple testing problem II
NPC: non‐parametric combination
PALM: Permutation Analysis of Linear Models
PET: Positron emission tomography
RD: radial diffusivity
RTP: rank truncated product;
SII: secondary somatosensory cortex
TFCE: threshold‐free cluster enhancement
TPM: truncated product method
TS: tail strength
TTS: truncated tail strength
UIT: union–intersection test

## INTRODUCTION

In this paper we show that permutation tests can provide a common solution to seemingly disparate problems that arise when dealing with multiple imaging measurements. These problems refer to the multiplicity of tests, and to the combination of information across multiple modalities for joint inference. We begin by describing each of these problems separately, then show how they are related, and offer a complete and generic solution that can accommodate a myriad of designs that can mix imaging and non‐imaging data. We also present an algorithm that has with amenable computational demands for treating these problems.

### Multiple Tests — but Not the Usual Multiplicity

Because in neuroimaging one statistical test is typically performed at each of many thousands of imaging units (e.g., voxels or vertices), the problems related to such multiplicity of tests were recognized almost as early as these techniques were developed [for pioneering examples, see Fox et al., 1988; Friston et al., 1991]. There is now a comprehensive body of literature on multiple testing correction methods that include those based on the random field theory, on permutation tests, as well as on other strategies that control the familywise error rate (fwer) or the false discovery rate (fdr) [for reviews, see Nichols and Hayasaka, 2003; Nichols, 2012]. However, the multiplicity of tests in neuroimaging can appear in other ways that are less explicit, and most importantly, that have not been fully appreciated or made available in software packages. In the context of the general linear model [glm, Scheffé, 1959], these *other* multiple tests include:

- *Multiple hypotheses in the same model*: Testing more than one hypothesis regarding a set of explanatory variables. An example is testing the effects of multiple variables, such as presence of a disease along with its duration, some clinical score, age and/or sex of the subjects, on a given imaging measurement, such as maps from functional magnetic resonance imaging (fmri) experiments.
- *Multiple pairwise group comparisons*: Often an initial global (omnibus) test is performed, such as an F‐test in the context of analysis of variance (anova), and if this test is significant, subsequent (post hoc) tests are performed to verify which pairwise difference(s) drove the global result, thus introducing a multiple comparisons problem.
- *Multiple models*: Testing more than one set of explanatory variables on one given dataset, that is, assembling and testing more than one design matrix, each with its own set of regressors, which may differ across designs, and each with its own set of contrasts. An example is interrogating the effect of distinct seeds, one at a time, in a resting‐state fmri experiment; another is in an imaging genetics experiment, testing multiple candidate polymorphisms.
- *Multiple modalities*: Testing separately, in the same study, more than one imaging modality as the response variable, such as fmri and positron‐emission tomography (pet), or different metrics from the same modality, such as various measurements from diffusion tensor imaging (dti), as fractional anisotropy (fa), mean diffusivity (md), or radial diffusivity (rd), or the effect of various networks identified using independent component analysis (ica).
- *Imaging and non‐imaging*: Testing separately, in the same study, imaging and non‐imaging measurements as response variables. An example is studying group effects on fmri and on behavioral or cognitive scores, such as iq, or disease severity scores, among countless other non‐imaging measurements.
- *Multiple processing pipelines*: Testing the same imaging modality multiple times, each time after a different processing pipeline, such as using filters with different widths for smoothing, or using different strategies for registration to a common space.
- *Multiple multivariate analyses*: Testing more than one multivariate hypothesis with the glm in repeated measurements designs, such as in profile analyses, in which the same data allows various different hypotheses about the relationships between explanatory and response variables.

In all these cases, the multiple tests cannot be assumed to be independent, so that the simple fwer correction using the conventional Bonferroni method risks a considerable loss in power. Modelling the degree of dependence between these tests can be a daunting task, and be suboptimal by invariably requiring the introduction of assumptions about the data, which, if at all valid, may not be sufficient. By contrast, robust, generic, multistep procedures, which do not depend as much on assumptions, or on independence among tests, such as the Benjamini–Hochberg procedure that controls the false discovery rate (fdr) [Benjamini and Hochberg, 1995; Genovese et al., 2002], do not guarantee that the spatial relationship between voxels or vertices within test is preserved when applied across *these* multiple tests, therefore being not as useful as in other settings. More specifically, the difficulty relates to correcting across various distinct imaging tests, while maintaining control across space within any given test, as opposed to controlling only within a single imaging test as commonly done. For the same reason, various multiple testing approaches that are applicable to many particular cases can hardly be used for the problems we discuss here; extensive details on these tests can be found in Hochberg and Tamhane [1987] and in Hsu [1996].

We call the multiple tests that arise in situations as those listed above “multiple testing problem ii” (mtp‐ii), to allow a distinction from the usual multiple testing problem due to the many voxels/vertices/faces that constitute an image, which we denote “multiple testing problem i” (mtp‐i). Methods that can be used in neuroimaging for the mtp‐i not always can be considered for the mtp‐ii, a problem that has remained largely without treatment; for two rare counter examples in which the mtp‐ii *was* considered, we point to the studies by Licata et al. [2013] and Abou Elseoud et al. [2014].

### Combination of Imaging Modalities

Acquisition of multiple imaging modalities on the same subjects can allow the examination of more complex hypotheses about physiological processes, and has potential to increase power to detect group differences. Such combination of modalities can refer strictly to data acquired from different instruments (e.g., mri, pet, eeg), or more broadly, to data acquired from the same instrument using different acquisition parameters (e.g., different mri sequences, different pet ligands); for overviews, see Uludağ and Roebroeck [2014], Zhu et al. [2014] and Calhoun and Sui [2016]; for example applications, see Hayasaka et al. [2006] and Thomas et al. [2015]. Irrespective of which the modalities are, the options in the context of the glm rest in testing for a single multivariate hypothesis, or in testing for a combination of multiple univariate hypotheses. Single multivariate tests encompass various classical tests, known in particular cases as multivariate analysis of variance (manova), multivariate analysis of covariance (mancova), or canonical correlation/variates analysis (cca/cva); these tests will be referred here as *classical multivariate tests*, or cmv.

The combination of multiple univariate hypotheses requires that each is analyzed separately, and that these results are grouped together to test, at each voxel (or vertex, or face) a *joint null hypothesis* (jnh); in this context, the separate tests are termed *partial tests*. Different criteria to decide upon rejection of the jnh give rise to three broad categories of combined tests: (i) reject if any partial test is significant; (ii) reject if all partial tests are significant; and (iii) reject if some aggregate measure from the partial tests is significant. The first of these can be traced back to Tippett [1931], and in current terminology, could be defined as rejecting the joint null hypothesis if any partial test is rejected at the fwer level using the Šidák correction [Šidák, 1967]; it also corresponds to a *union–intersection test* [uit, Roy, 1953]. The second is the *intersection–union test* [iut, Berger, 1982], that in neuroimaging came to be known as *conjunction test* [Nichols et al., 2005]. The third offers a trade‐off between the two other approaches, and gives rise to a large number of possible tests, each with a different rejection region, and therefore with different sensitivity and specificity profiles; some of these tests are popular in meta‐analyses, with the method of Fisher [Fisher, 1932] being one of the most used, and new approaches are continually being developed. A summary is shown in Table 1, and a brief overview of these and yet other tests, along with bibliographic information, is in Appendix A.

**Table 1:** A list of various functions for joint inference.

Method	Test statistic ( T)	p‐value ( P)
Tippett	$min\left(p_{k}\right)$	$1-{\left(1-T\right)}^{K}$
Fisher	$-2\sum_{k=1}^{K}ln\left(p_{k}\right)$	$1-\chi_{\mathrm{cdf}}^{2}\left(T;\;\nu =2K\right)$
Stouffer	$\frac{1}{\sqrt{K}}\sum_{k=1}^{K}\Phi^{-1}\left(1-p_{k}\right)$	$1-\Phi \left(T;\;\mu =0,\;\sigma^{2}=1\right)$
Wilkinson	$\sum_{k=1}^{K}I\left(p_{k}\leq \alpha \right)$	∑k=TKKkαk(1−α)K−k
Good	$\prod_{k=1}^{K}p_{k}^{w_{k}}$	$\sum_{k=1}^{K}w_{k}^{K-1}T^{1/w_{k}}\left(\prod_{i=1}^{k-1}{\left(w_{k}-w_{i}\right)}^{-1}\right)\left(\prod_{i=k+1}^{K}{\left(w_{k}-w_{i}\right)}^{-1}\right)$
Lancaster	$\sum_{k=1}^{K}w_{k}F_{k}^{-1}\left(1-p_{k}\right)$	$1-G\left(T\right)$
Winer	$\sum_{k=1}^{K}t_{\mathrm{cdf}}^{-1}\left(1-p_{k};\nu_{k}\right)/\sqrt{\sum_{k=1}^{K}\frac{\nu_{k}}{\nu_{k}-2}}$	$1-\Phi \left(T;\;\mu =0,\;\sigma^{2}=1\right)$
Edgington	$\sum_{k=1}^{K}p_{k}$	∑j=0⌊T⌋(−1)jKjT−jKK!
Mudholkar–George	$\frac{1}{\pi }\sqrt{\frac{3(5K+4)}{K(5K+2)}}\sum_{k=1}^{K}ln\left(\frac{1-p_{k}}{p_{k}}\right)$	$1-t_{cdf}(T;\;\nu =5K+4)$
Darlington–Hayes	$\frac{1}{r}\sum_{k=1}^{r}\Phi^{-1}\left(1-p_{\left(k\right)}\right)$	Computed through Monte Carlo methods. Tables are available.
Zaykin et al. (tpm)	$\prod_{k=1}^{K}p_{k}^{I\left(p_{k}\leq \alpha \right)}$	∑k=1KKk1−αK−kIT>αkαk+IT≤αkT∑j=0k−1kln⁡α−ln⁡Tjj!
Dudbridge–Koeleman (rtp)	$\prod_{k=1}^{r}p_{\left(k\right)}$	Kr+1r+1∫011−tK−r−1AT,t,Kdt
Dudbridge–Koeleman (dtp)	$\max\left(\prod_{k=1}^{r}p_{\left(k\right)},\prod_{k=1}^{K}p_{k}^{I\left(p_{k}\leq \alpha \right)}\right)$	∑k=1rKk1−αK−kAT,α,k+Ir<KKr+1r+1∫0α1−tK−r−1AT,t,Kdt
Taylor–Tibshirani (ts)	$\frac{1}{K}\sum_{k=1}^{K}\left(1-p_{\left(k\right)}\frac{K+1}{k}\right)$	$1-\Phi \left(T;\;\mu =0,\;\sigma^{2}\approx \frac{1}{K}\right)$
Jiang et al. (tts)	$\frac{1}{K}\sum_{k=1}^{K}I\left(p_{\left(k\right)}\leq \alpha \right)\left(1-p_{\left(k\right)}\frac{K+1}{k}\right)$	Computed through Monte Carlo methods.

Various functions are available for joint inference on multiple tests. For each method, both its statistic ( T) and associated p‐value ( P) are shown. These p‐values are only valid if, for each method, certain assumptions are met, particularly with respect to the independence between tests, but sometimes also with respect to underlying distributions. Under exchangeability, the p‐values can be computed using permutation tests, and the formulae in the last column are no longer necessary. The tests are shown in chronological order; see Appendix A for details and bibliographic information. T is the statistic for each method and P its asymptotic p‐value. All methods are shown as function of the p‐values for the partial tests. For certain methods, however, the test statistic for the partial tests, if available, can be used directly. K is the number of tests being combined; $p_{k}$, k={1, 2,…,K} are the partial p‐values; $w_{k}$ are positive weights assigned to the respective $p_{k}$; $p_{\left(r\right)}$ are the $p_{k}$ with rank r in ascending order (most significant first); α is the significance level for the partial tests; $I\left(\cdot \right)$ is an indicator function that evaluates as 1 if the condition is satisfied, 0 otherwise; $\left\lfloor \cdot \right\rfloor$ represents the floor function; $\chi_{\mathrm{cdf}}^{2}$ is the cumulative distribution function (cdf) for a chi‐squared distribution, with ν degrees of freedom; $t_{cdf}$ is the cdf of the Student's t distribution with degrees of freedom ν, and $t_{\mathrm{cdf}}^{-1}$ its inverse; Φ is the cdf of the normal distribution with mean μ and variance $\sigma^{2}$, and $\Phi^{-1}$ its inverse; and F and G are the cdf of arbitrary, yet well chosen distributions. For the two Dudbridge–Koeleman methods, $A\left(T,a,b\right)=I\left(T>a^{b}\right)a^{b}+I\left(T\leq a^{b}\right)T\sum_{j=0}^{b-1}{\left(b\ln a-\ln T\right)}^{j}/j!$.

Both cases — a single multivariate test or the combination of multiple univariate tests — can be assessed parametrically when the asymptotic distribution of the test statistic is known, which may sometimes be the case if various assumptions about the data are met. These generally refer to the independence or common dependence between observations and between tests, to the distribution of the error terms, and for brain imaging, to yet other assumptions regarding the relationship, across space, between the tests. However, if the observations are exchangeable, that is, if their joint distribution remains unchanged after shuffling, then all such assumptions can be eschewed at once, and instead, permutation tests can be performed. The p‐values can then be computed for either the classical multivariate tests, or for the combination of univariate tests; when used in the last case, the strategy corresponds to Pesarin's method of *non‐parametric combination* [npc, Pesarin, 1990, 2001], discussed below. Exchangeability is assumed only for the observations within each partial test (or for the errors terms of the respective models, see below); exchangeability is not assumed between the partial tests for either cmv or npc. Moreover, non‐independence does not need to be explicitly modelled, either between observations, between partial tests, or across space for imaging data, thus making such tests applicable to a wide variety of situations.

### Overview of the Article

We show that a single, elegant permutation solution is available for all the situations described above, addressing the comparisons of response variables when these can be put in comparable scale, the correction of p‐values, via adjustment to allow exact control over fwer in the various multiple testing scenarios described above, and the combination of multiple imaging modalities to allow for joint inference. The conjunction of multiple tests is a special case in which the null hypothesis differs from that of a combination, even though it can be approached in a similar fashion; because the distinction is quite an important one, it is also discussed.

In the next section, we outline the notation used throughout the paper. We then use the definition of union‐intersection tests, closed testing procedures, and synchronized permutations to correct for multiple hypotheses, allowing flexibility to mix in the same framework imaging data with different spatial resolutions, surface and/or volume‐based representations of the brain, and even non‐imaging data. For the problem of joint inference, we propose and evaluate a modification of the npc, such that instead of two phases and large data storage requirements, the permutation inference can be performed in a single phase, without prohibitive memory needs. We also evaluate, in the context of permutation tests, various combining methods that have been proposed in the past decades, and identify those that provide the best control over error rate and power across a range of situations. We also exemplify the potential gains in power with the reanalysis of the data from a pain study. In the Appendices, we provide a brief historical review of various combining functions and discuss criteria of consistency and admissibility. In the Supporting Information we provide an algorithm that allows combination and correction in a unified framework.

## THEORY

### Notation and General Aspects

For a given voxel (or vertex, or face), consider a multivariate glm:

(1) Y=Xβ+ϵ

where Y is the N×K matrix of observed data, with N observations of K distinct (possibly non‐independent) variables, X is the full‐rank N×R design matrix that includes explanatory variables (i.e., effects of interest and possibly nuisance effects), β is the R×K matrix of R regression coefficients for each of the K variables, and ϵ is the N×K array of random errors. Estimates for β can be computed by ordinary least squares, i.e., $\hat{\beta }=X^{+}Y$, where the superscript ${(}^{+})$ denotes a pseudo‐inverse. One generally wants to test the null hypothesis that a given combination (contrast) of the elements in β equals to zero, that is, $H^{0}:C'\beta D=0$, where C is a R×S full‐rank matrix of S contrasts of coefficients on the regressors encoded in X, 1≤S≤R and D is a K×Q full‐rank matrix of Q contrasts of coefficients on the dependent, response variables in Y, 1≤Q≤K. Often more than one such standard multivariate hypothesis is tested, each regarding different aspects of the same data, and each using a different pair of contrasts C and D. Not uncommonly, even different sets of explanatory variables are considered, sometimes arranged in entirely different designs. We denote the set of such design matrices as $X=\left\{X\right\}$, the set of pairs of contrasts for each hypothesis related to that design as $C_{X}=\left\{\left(C,D\right)\right\}$, and the set of sets of such contrasts as $\left\{C_{X}\right\}$.

Depending on the values of K, Q, and S, $H^{0}$ can be tested using various common statistics. If K=1, or if K>1 and Q=1, the problem reduces to the univariate case, in which a t statistic can be used if S=1, or an F‐statistic if S≥1. If K>1 and Q>1, the problem is a multivariate proper and can be approached via cmv when respective multivariate Gaussian assumptions are satisfied; in these cases, if S=1, the Hotelling's $T^{2}$ statistic can be used [Hotelling, 1931], whereas if S>1, various other statistics are available, such as the Wilks’ λ [Wilks, 1932], the Lawley–Hotelling's trace [Hotelling, 1951; Lawley, 1938], the Roy's largest root(s) [Kuhfeld, 1986; Roy, 1953], and the Pillai's trace [Pillai, 1955]; the merits of each in the parametric case are discussed in various textbooks [Anderson, 2003; e.g., Christensen, 2001; Johnson and Wichern, 2007; Timm, 2002], and such tests have been applied to neuroimaging applications [Chen et al., 2014].

The model in Eq. (1) can be rewritten as $\tilde{Y}=X\tilde{\beta }+\tilde{\epsilon }$, where $\tilde{Y}=YD$, $\tilde{\beta }=\beta D$ and $\tilde{\epsilon }=\epsilon D$. If Q=1, this is a univariate model, otherwise it remains multivariate, with $\tilde{Y}$ having $\tilde{K}=Q$ columns, and the null hypothesis simplified as $H^{0}:C'\tilde{\beta }=0$. This null is equivalent to the original, and can be split into multiple partial hypotheses $H_{\tilde{k}}^{0}:C'{\tilde{\beta }}_{\tilde{k}}=0$, where ${\tilde{\beta }}_{\tilde{k}}$ is the $\tilde{k}$‐th column of $\tilde{\beta }$, $\tilde{k}=\;1,\ldots ,\tilde{K}$. This transformation is useful as it defines a set of separate, even if not independent, partial hypotheses, that can be tested and interpreted separately. We drop heretofore the “ ∼” symbol, with the modified model always implied.

Non‐parametric inference for these tests can be obtained via permutations, by means of shuffling the data, the model, the residuals, or variants of these, in a direct extension from the univariate case [Winkler et al., 2014, Table 2]. To allow such rearrangements, some assumptions need to be made: either of exchangeable errors (ee) or of independent and symmetric errors (ise). The first allows permutations, the second sign flippings; if both are available for a given model, permutations and sign flippings can be performed together. We use generically the terms *rearrangement* or *shuffling* when the distinction between permutations or sign flippings is not pertinent. These are represented by permutation and/or sign flipping matrices $P_{j}$, j= 1,…,J, where J is the number of such rearrangements.

Another aspect that concerns permutation tests refers to the use of statistics that are pivotal, i.e., that have sampling distributions that do not depend on unknown parameters. Most statistics used with parametric tests (and all the uni‐ and multivariate examples from the previous paragraph) are pivotal if certain assumptions are met, especially homoscedasticity. Their benefits in non‐parametric tests are well known [Hall and Wilson, 1991], and for neuroimaging, pivotal statistics are useful to allow exact correction for the mtp‐i.

### Union–Intersection and Intersection–Union Tests

**Figure 1:**
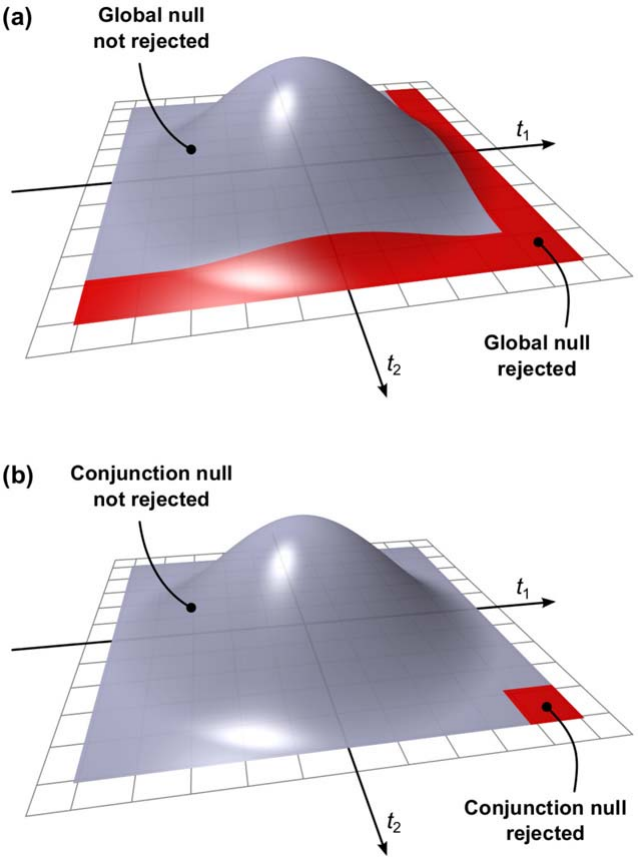
(a) Rejection region of a union–intersection test (uit) based on two independent *t*‐tests. The null is rejected if either of the partial tests has a statistic that is large enough to be qualified as significant. (b) Rejection region of an intersection–union test (iut) based the same tests. The null is rejected if both the partial tests have a statistic is large enough to be qualified as significant. [Color figure can be viewed in the online issue, which is available at http://wileyonlinelibrary.com.]

Consider the set of p‐values $\left\{p_{k}\right\}$ for testing the respective set of partial null hypotheses $\left\{H_{k}^{0}\right\}$. A union–intersection test [uit, Roy, 1953] considers the jnh corresponding to a *global null hypothesis* that all $H_{k}^{0}$ are true; if any such partial null is rejected, the global null hypothesis is also rejected. An intersection–union test [iut, Berger, 1982] considers the jnh corresponding to a *conjunction null hypothesis* (also termed disjunction of null hypotheses) that any $H_{k}^{0}$ is true; if all partial nulls are rejected, the conjunction null hypothesis is also rejected. In the uit, the null is the intersection of the null hypotheses for all partial tests; the alternative is the union of the alternatives. In the iut, the null is the union of the null hypotheses for all partial tests; the alternative is the intersection of the alternatives. A uit is significant if the smallest $p_{k}$ is significant, whereas an iut is significant if the largest $p_{k}$ is significant. Figure 1 illustrates the rejection regions for uit and iut cases based on two independent t‐tests, in which the statistic larger than a certain critical level is considered significant. Table 2 shows the null and alternative hypotheses for each case.

**Table 2:** Joint hypotheses tested with union–intersection and intersection–union of K partial tests

	uit	iut
Null hypothesis ( $H^{0}$)	$\overset{K}{\underset{k=1}{\cap }}H_{k}^{0}$	$\overset{K}{\underset{k=1}{\cup }}H_{k}^{0}$
Alternative hypothesis ( $H^{1}$)	$\cup_{k=1}^{K}H_{k}^{1}$	$\overset{K}{\underset{k=1}{\cap }}H_{k}^{1}$

In the uit, the null is also called *global null hypothesis*, whereas in the iut, the null is also called *conjunction null hypothesis*.

Enlarging the number of tests affects uits and iuts differently. For the uit with a given statistic threshold, more tests increase the chances of false positives, and correction for this multiplicity needs to be applied. In fact, it can be shown that a uit at a significance level α is equivalent to controlling the fwer at α for the same tests. In other words, a union‐intersection procedure is an fwer procedure. For an iut, in contrast, the procedure does not change with more tests. The conjunction null hypothesis is composite, consisting of different parameter settings. For the extreme case that exactly one partial null is true and K−1 effects are real, an iut is exact for any K; if two or more partial nulls are true, an iut becomes increasingly conservative with larger K.

The null hypothesis of the uit can be rejected if the smallest $p_{k}$ is significant or, equivalently, its corresponding statistic, that is, the extremum statistic. For tests in which larger statistics provide evidence against the null hypothesis, the relevant extremum is the maximum. Conversely, for tests in which smaller statistics provide evidence against the null, the extremum is the minimum. Clearly, if the most extreme statistic is significant, at least one partial hypothesis is rejected, therefore the global null hypothesis can be rejected without the need to continue testing the other K−1 partial hypotheses. The null hypothesis of the iut can be rejected if the largest $p_{k}$ is significant or, equivalently, its corresponding least extreme statistic. Clearly, if the least extreme statistic is significant, all partial hypotheses can be rejected, therefore the conjunction hypothesis can be rejected without the need to continue testing all other K−1 partial hypotheses.

In brain imaging, the term *conjunction* refers to a test performed when one wants to localize regions where there is signal in all partial tests, that is, a logical and of all alternative hypotheses [Nichols et al., 2005], and is synonymous with the iut. In noting the lack of power of such a proper conjunction test, Friston et al. [2005] suggested a partial conjunction, in which fewer than all alternatives need to intersect. Using the same notation of Table 1, both approaches have the same statistic, $T=max\left(p_{k}\right)$, but the p‐value of the latter can be computed as $T^{K-v+1}$, so that the test is a conjunction of at least v alternative hypotheses; if v=K, it is an iut, and if v=1 the null is equivalent to that of a uit (such a test, however, is inconsistent for a uit; see Appendix B). Benjamini and Heller [2008] further generalized the procedure by allowing the combination of the largest p‐values using any of various possible combining functions, such as those we present in Table 1 and in Appendix A.

### Closed Testing

In a closed testing procedure (ctp), each $H_{k}^{0}$ is rejected if, and only if, it is significant in its own right at a certain level α, and if all possible sub‐jnhs that include the same $H_{k}^{0}$ and comprise some or all of the partial hypotheses (that is, subsets of the global jnh formed by some of the partial tests) are also rejected at α using a suitable test. Various such tests can be considered, including cmvs and npc (next section).

A ctp guarantees strong control over fwer [Marcus et al., 1976]. To produce adjusted p‐values, the original method requires that all $2^{K}-1$ sub‐jnhs are tested^1^, a requirement that is computationally onerous, even for a moderate number of tests, a problem aggravated by the large number of tests that are considered in an imaging experiment. There exists, however, a particular test for the sub‐jnhs that obviates the need for such a gargantuan computational venture: the union–intersection test. In a uit using the extremum statistic, the most extreme of the global jnh that comprises all the K partial tests is also the most extreme of any other sub‐jnh that includes that particular partial hypothesis, such that the other joint subtests can be bypassed altogether. As a uit is also an fwer‐controlling procedure, this raises various possibilities for correction of both mtp‐i and mtp‐ii. While such a shortcut can be considered for both parametric [Holm, 1979] and non‐parametric cases [Westfall and Young, 1993], for the non‐parametric methods using permutation, one additional feature is needed: that the joint sampling distribution of the statistic used to test each of the sub‐jnh is the same regardless whether the null is true for all the K partial tests, or just some of them. This property is called subset pivotality [Westfall and Troendle, 2008; Westfall and Young, 1993], and it constitutes the multivariate counterpart to the univariate pivotality.

### Non‐Parametric Combination

The npc consists of testing each of the $H_{k}^{0}$ using shufflings that are performed synchronously for all K partial tests. The resulting statistics for each permutation are recorded, allowing an estimate of the complete empirical null distribution to be constructed for each partial test. In a second stage, the empirical p‐values for each statistic are combined, for each permutation, into a joint statistic. As such a combined joint statistic is produced from the previous permutations, an estimate of its empirical distribution function is immediately known, and so the p‐value of the unpermuted statistic, hence of the joint test, can be assessed. The method was proposed by Pesarin [1990; 1992], and independently, though less generically, by Blair et al. [1994]; a thorough description is available in Pesarin [2001] and Pesarin and Salmaso [2010a]. An early application to brain imaging can be found in Hayasaka et al. [2006], its use to combine different statistics within the same modality in Hayasaka and Nichols [2004], and a summary description and practical examples are presented in Brombin et al. [2013]. The jnh of the combined test is that all partial null hypotheses are true, and the alternative that any is false, which is the same null of a uit, although the rejection region may differ widely from the example in Figure 1a, depending on the combining function.

The only two requirements for the validity of the npc are that the partial test statistics have the same direction suggesting the rejection of the null hypothesis, and that they are consistent (see Appendix B). For the combining function, it is desirable that (i) it is non‐decreasing with respect to all its arguments (which are the p‐values $p_{k}$, or $1-p_{k}$, depending on the combining function), (ii) that it approaches its maximum (or minimum, depending on the function) when at least one of the partial tests approaches maximum significance (that is, when at least one p‐value approaches zero), and (iii) that for a test level α>0, the critical significance threshold is smaller than the function maximum value. These requirements are easily satisfied by almost all functions shown in Table 1, which therefore can be used as combining functions in the framework of npc (see Appendix B for a discussion on the few exceptions).

One of the most remarkable features of npc is that the synchronized permutations implicitly account for the dependence structure among the partial tests. This means that even combining methods originally derived under an assumption of independence, such as Tippett or Fisher, can be used even when independence is untenable. In fact, modifications to these procedures to account for non‐independence [e.g., Brown, 1975; Kost and McDermott, 2002 for the Fisher method] are made redundant. As the p‐values are assessed via permutations, distributional restrictions are likewise not necessary, rendering the npc free of most assumptions that thwart parametric methods in general. This is why npc methods are an alternative to cmv tests, as each of the response variables in a manova or mancova analysis can be seen as an univariate partial test in the context of the combination.

### Transformation of the Statistics

While npc offers flexibility in a simple and uncomplicated formulation, its implementation for brain imaging applications poses certain challenges. Because the statistics for all partial tests for all permutations need to be recorded, enormous amounts of data storage space may be necessary, a problem further aggravated when more recent, high resolution imaging methods are considered. Even if storage space were not a problem, however, the discreteness of the p‐values for the partial tests becomes problematic when correcting for multiple testing, because with thousands of tests in an image, ties are very likely to occur among the p‐values, further causing ties among the combined statistics. If too many tests across an image share the same most extreme statistic, correction for the mtp‐i, while still valid, becomes less powerful [Pantazis et al., 2005; Westfall and Young, 1993]. The most obvious workaround — run an ever larger number of permutations to break the ties — may not be possible for small sample sizes, or when possible, requires correspondingly larger data storage.

However, another possible approach can be considered after examining the two requirements for the partial tests, and also the desirable properties (i)–(iii) of the combining functions, all listed earlier. These requirements and properties are quite mild, and if the sample size is reasonably large and the test statistics homogeneous, i.e., they share the same asymptotic permutation distribution, a direct combination based not on the p‐values, but on the statistics themselves, such as their sum, can be considered [Pesarin and Salmaso, 2010a]. Sums of statistics are indeed present in combining functions such as of Stouffer, Lancaster, Winer, and Darlington–Hayes, but not others listed in Table 1 and Appendix A. In order to use these other combining functions, most of them based on p‐values for the partial tests, and under the same premises, the statistics need to be transformed to quantities that behave as p‐values. In the parametric case, these would be the parametric p‐values, computed from the parametric cumulative distribution function (cdf) of the test statistic. If the parametric assumptions are all met for the partial tests, their respective parametric p‐values are all valid and exact; if the assumptions are not met, these values are no longer appropriate for inference on the partial tests, but may still be valid for npc, for satisfying all requirements and desirable properties of the combining functions. As they are not guaranteed to be appropriate for inference on the partial tests, to avoid confusion, we call these parametric p‐values “u‐values”.

Another reason for not treating u‐values as valid p‐values is that they do not necessarily need to be obtained via an assumed, parametric cumulative distribution function for the statistics of the partial tests. If appropriate, other transformations applied to the statistics for the partial tests can be considered; whichever is more accurate to yield values in the interval [0;1] can be used. The interpretation of a u‐value should not be that of a probability, but merely of a monotonic, deterministic transformation of the statistic of a partial test, so that it conforms to the needs of the combining functions.

**Figure 2:**
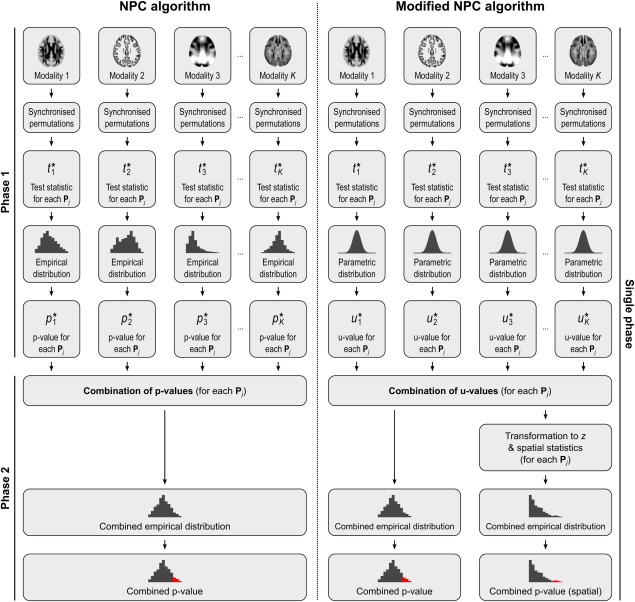
The original npc algorithm combines non‐parametric p‐values and, for imaging applications, requires substantial amount of data storage space. Two modifications simplify the procedures: (1) the statistic *t_k_* for each partial test *k* is transformed into a related quantity *u*_*k*_ that has a behavior similar to the p‐values, and (2) the combined statistic is transformed to a variable that follows approximately a normal distribution, so that spatial statistics (such as cluster extent, cluster mass, and tfce) can be computed as usual. The first simplification allows the procedure to run in a single phase, without the need to retrieve data for the empirical distribution of the partial tests. [Color figure can be viewed in the online issue, which is available at http://wileyonlinelibrary.com.]

Transformation of the statistic to produce quantities that can be used in place of the non‐parametric p‐values effectively simplifies the npc algorithm, greatly reducing the data storage requirements and computational overhead, and avoiding the losses in power induced by the discreteness of p‐values. This simplification is shown in Figure 2, alongside the original npc algorithm.

Regardless of the above transformation, the distribution of the combined statistic, T, may vary greatly depending on the combining function, and it is always assessed non‐parametrically, via permutations. Different distributions for different combining functions can, however, pose practical difficulties when computing spatial statistics such as cluster extent, cluster mass, and threshold‐free cluster enhancement [tfce, Smith and Nichols, 2009]. Consider for instance the threshold used to define clusters: prescribed values such as 2.3 or 3.1 [Woo et al., 2014] relate to the normal distribution and are not necessarily sensible choices for combining functions such as Tippett or Fisher. Moreover, for some combining functions, such as Tippett and Edgington, smaller values for the statistic are evidence towards the rejection of the null, as opposed to larger as with most of the others. To address these practical issues, a monotonic transformation can be applied to the combined statistic, so that its behavior becomes more similar to, for instance, the z‐statistic [Efron, 2004]. This can be done again by resorting to the asymptotic behavior of the tests: the combined statistic is converted to a parametric p‐value (the formulas are summarized in Table 1) which, although not valid for inference unless certain assumptions are met, particularly with respect to the independence among the partial tests, are useful to transform, at each permutation, the combined statistic to the z‐statistic, which can then be used for inference using cluster extent, mass, or tfce.

### Directed, Non‐Directed, and Concordant Hypotheses

When the partial hypotheses are one‐sided, i.e., $H_{k}^{0}:C'\beta_{k}>0$ or $H_{k}^{0}:C'\beta_{k}<0$, and all have the same direction (either), the methods presented thus far can be used as described. If not all have the same direction, a subset of the tests can be scaled by −1 to ensure a common direction for all.

If the direction is not relevant, but the concordance of signs towards one of them (either) is, a new combining test can be constructed using one‐sided p‐values, $p_{k}$, and another using $1-p_{k}$, then taking the best of these two results after correcting for the fact that two tests were performed. For example, for the Fisher method, we would have:

(2) $$T=\text{max}\left(-2\overset{K}{\underset{k=1}{\sum }}\text{ln}\left(p_{k}\right),-2\overset{K}{\underset{k=1}{\sum }}\text{ln}\left(1-p_{k}\right)\right)$$

where T is the combined test statistic, with its p‐value, P, assessed via permutations.

If direction or concordance of the signs are not relevant, two‐sided (non‐directed) tests and p‐values can be used before combining, that is, ignoring the sign of the test statistic for the partial tests, or using a statistic that is non‐directional (e.g., with F‐tests for the partial hypotheses). It worth mentioning, however, that it is not appropriate to simultaneously ignore directions of the partial tests *and* use a combination that favors concordant signs. Such a test would lack meaning and would be inadmissible, with examples shown in Appendix C.

**Figure 3:**
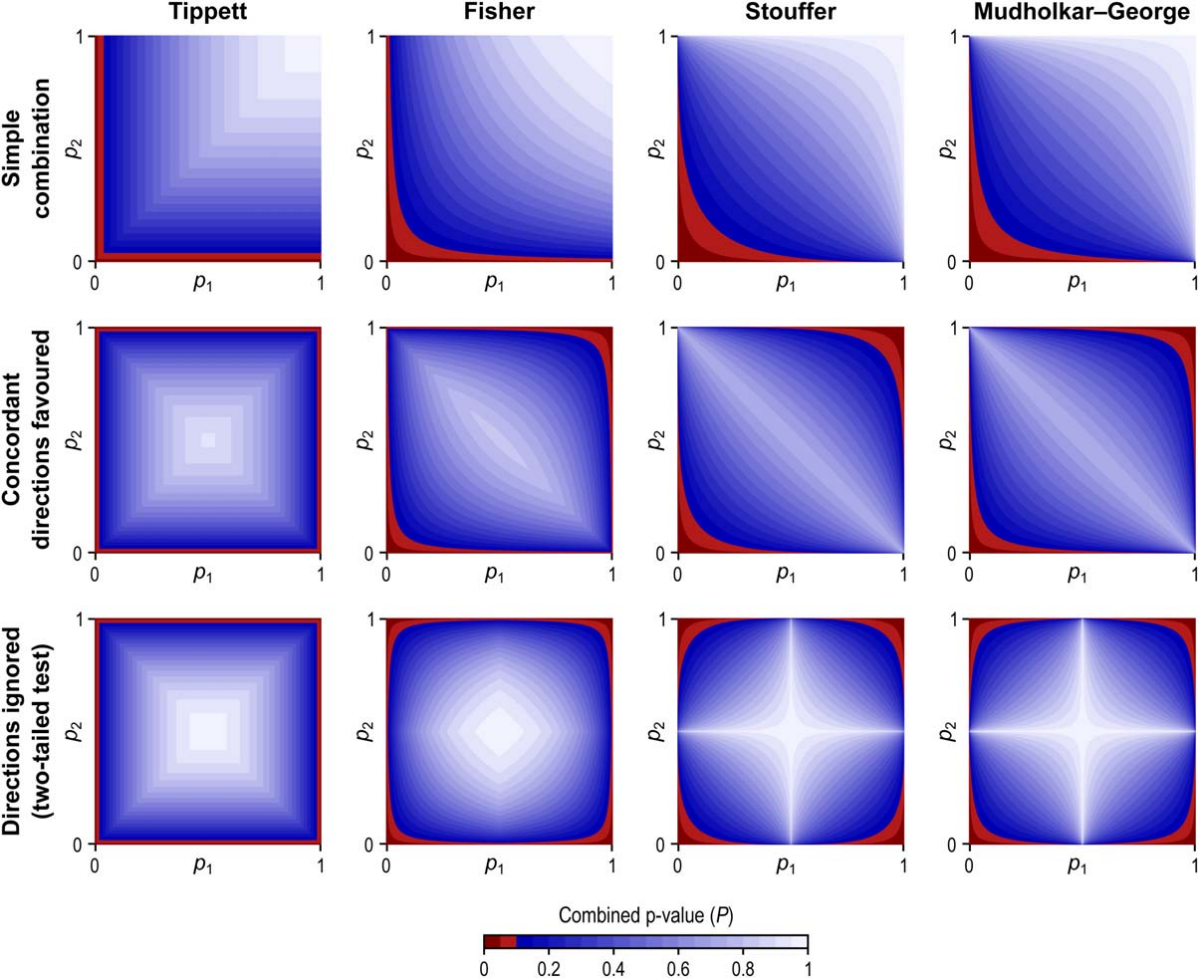
Upper row: Rejection regions for the combination of two partial tests using four different combining functions, and with the p‐values assessed parametrically (Table I). The regions are shown as function of the p‐values of the partial tests (*p_k_*). Middle row: Rejection regions for the same functions with the modification to favor alternative hypotheses with concordant directions. Lower row: Rejection regions for the same functions with the modification to ignore the direction altogether, that is, for two‐tailed partial tests. [Color figure can be viewed in the online issue, which is available at http://wileyonlinelibrary.com.]

Rejection regions for these three cases, for four different combining functions, are shown in Figure 3, as functions of the partial p‐values, for K=2 partial tests.

### The Method of Tippett

From the various combining functions listed in Table 1, consider the combining function of Tippett [1931], that has statistic $T=min(p_{k})$ and, when all partial tests are independent, a p‐value $P=1-{\left(1-T\right)}^{K}$. This test has interesting properties that render it particularly attractive for imaging:

- It defines a uit test: If the minimum p‐value remains significant when all tests are considered, clearly the global null hypothesis can be rejected.
- It controls the fwer: Controlling the error rate of a uit is equivalent to an fwer‐controlling procedure over the partial tests.
- If the partial tests are independent, it defines an exact fwer threshold: The function is closely related to Šidák [1967] correction: set $P=\alpha^{\mathrm{FWER}}$, then $T^{\text{FWER}}=1-{\left(1-\alpha^{\text{FWER}}\right)}^{1/K}$; one can retain only the partial p‐values that satisfy $p_{k}\leq T^{\text{FWER}}$. Adjusted p‐values can be obtained similarly through the Šidák procedure, that is $p_{k}^{\text{FWER}}=1-{\left(1-p_{k}\right)}^{1/K}$.
- If the partial tests are not independent, it still defines an fwer threshold and adjusted p‐values: As a uit, the Tippett function can be used in a closed testing procedure. Further, it is the function that makes ctp with large K feasible in practice; adjusted p‐values are obtained with the distribution of the minimum p‐value (or of the extremum statistic).
- Because it subsumes correction using the extremum statistic that is already in use in imaging to account for mtp‐i, the correction for the mtp‐ii can be done by pooling the maximum statistics across both space and the set of partial tests. This allows algorithmic advantages that we exploit in the proposed implementation shown in the Supporting Information.
- It can be used as the combining function with npc, thus providing a common procedure for correction and for combination of p‐values.
- It is fast to compute: Taking the extremum statistic or minimum p‐value is trivial compared with other functions that require cumulative sums or products, multiple parameters, integrations, or that depend on Monte Carlo simulations.

While the Tippett function is advantageous for all these reasons, note that, even when other combining functions are used for npc, the extremal statistic (equivalent to the Tippett combining function) is also used for the mtp‐i to control fwer over space.

### A Unified Procedure

Armed with these concepts, and with the modifications to the original npc algorithm, we are positioned to tackle the various problems identified in the Introduction:

#### Combination of multiple modalities

With K modalities, all in register and with the same spatial resolution, each is tested separately, using synchronized permutations, and their statistics converted to u‐values for each shuffling. These are combined using a suitable combining function, such as one from those shown in Table 1. The p‐values for the combined statistic are produced using the same set of permutations used to assess each test separately. This is the modified npc algorithm that we propose, shown in Figure 2.

#### Correction for multiple modalities

With K modalities, which are not necessarily in register, nor with the same resolution, nor of the same type (e.g., some from volumetric, some from surface representations of the brain), or which may not necessarily be all related to imaging (e.g., some imaging and some non‐imaging data), each is tested separately using a suitable test statistic. The permutation distribution of the extremum statistic across *all* tests is produced and used to compute fwer‐adjusted p‐values that simultaneously address the mtp‐i and mtp‐ii.

#### Correction for multiple designs and contrasts

Each pair of contrasts defined by $\left(C,D\right)$ allows the corresponding design matrix to be partitioned into effects of interest and nuisance effects [Winkler et al., 2014, Appendix A], and also the redefinition of the response variables (Section “Notation and general aspects”). Thus, multiple designs and their respective contrasts can be tested separately. Differently than for the correction for multiple modalities, however, with different contrasts, their respective statistics may possess different asymptotic behavior (due to, e.g., the contrasts having different ranks, or the designs having different degrees of freedom), thus precluding the use of the distribution of the extremum statistic. When known, the asymptotic behavior can be used to convert these statistics — univariate or multivariate — to a z‐statistic. The distribution of the maximum across the results of the various designs and contrasts can then be computed and used for correction.

#### Correction for multiple modalities, designs, and contrasts

Following the same principles, it is also possible to account for the multiplicity of input modalities, each tested with their respective design and set of contrasts, or each tested versus all designs and contrasts. Each test is applied separately, statistics converted to a z‐statistic based on their asymptotic behavior, and the distribution of the extremum used to obtain adjusted p‐values for all in a ctp using a uit. It is not necessary that all are in register, neither that all use the same kind of image representation of the brain (i.e., volume or surface), nor that they are even all (or any) imaging‐related, and can therefore include clinical or behavioral, biomarkers, and other types of data.

#### Conjunctions

An iut can be assessed through permutations simply by computing $max\left(p_{k}\right)$, which is, in its own right, the p‐value of the iut, such that there is no need for transformation into u‐values for the assessment of the combined statistic. In the context of imaging, such conjunctions can be used with statistics at every voxel (or vertex or face), thus allowing also certain spatial statistics such as tfce.

Since combinations and conjunctions are performed at each individual image point, it is necessary that all images have been registered to the same common space and possess similar spatial resolution [Lazar et al., 2002]. This can be accomplished through intrasubject and intersubject registration, and resampling. By contrast, correction for the multiplicity of tests uses the maximum statistic across such tests, thus not requiring that the tests match on space, or even that they are all related to imaging. However, they explicitly require pivotal statistics [for pivotality in this context, see Winkler et al., 2014], so that the extreme is taken from statistics that share the same sampling distribution. The statistics used with cmv and npc are all pivotal and therefore can be used. Spatial statistics, however, lack this property and require similar search volumes and resolutions, even for correction. Moreover, by including information from neighboring voxels, such as using spatial smoothing or spatial statistics like tfce [Smith and Nichols, 2009], subset pivotality is lost, meaning that strong control of fwer cannot be guaranteed. In practice, though, the power gained by pooling information over space is essential. In the Supporting Information we provide an algorithm that generically implements the combination and correction methods presented.

## EVALUATION METHODS

### Validity of the Modified NPC

To assess the validity of the proposed modification to the npc, we consider one of the simplest scenarios that would have potential to invalidate the method and reduce power: this is the case of having a small number of partial tests, small sample size, and with each partial test possessing substantially different distributions for the error terms. We investigated such a scenario with K=2, varying sample sizes N={ 8, 12, 20, 30, 40, 50, 60, 70, 80, 120, 200 }, and different error distributions. Using the notation defined in Section “Notation and general aspects”, response variables were generated for each simulation using the model Y=Xβ+ϵ, with Y sized N×K. Each modality was simulated as having 500 points, these representing, for instance, voxels or vertices of an image representation of the brain. The errors, $\epsilon =\left[\epsilon_{1},\epsilon_{2}\right]$, were simulated following either a Gaussian distribution with zero mean and unit variance, or a Weibull distribution (skewed), with scale parameter 1 and shape parameter 1/3, shifted and scaled so as to have expected zero mean and unit variance. Different combinations of error distributions were used: Gaussian for both partial tests, Weibull for both partial tests, or Gaussian for the first, and Weibull for the second partial test.

The response data, Y, were constructed by adding the simulated effects, Xβ, to the simulated errors, where $\beta =\left[\beta_{1},\beta_{2}\right]$, with $\beta_{k}\;=\;[\beta_{1},0]'$, $\beta_{1}$ being either 0 (no signal) or $t_{\mathrm{cdf}}^{-1}\left(1-\alpha ;N-rank\left(X\right)\right)/\sqrt{N}$ (with signal), where α = 0.05 is the significance level of the permutation test to be performed. This procedure ensures a calibrated signal strength sufficient to yield an approximate power of 50% for each partial test, with Gaussian errors, irrespective of the sample size; for non‐Gaussian errors this procedure does not guarantee power at the same level. The actual effect was coded in the first regressor of X, constructed as a vector of random values following a Gaussian distribution with zero mean and unit variance; the second regressor was modelled an intercept. All four possible combinations of presence/absence of effect among the K=2 partial tests were simulated, that is, (1) with no signal in any of the two partial tests, (2) with signal in the first partial test only, (3) with signal in the second partial test only, and (4) with signal in both partial tests.

The simulated data was tested using the Tippett and Fisher methods. The case with complete absence of signal was used to assess error rates, and the others to assess power. The p‐values were computed with 500 permutations, and the whole process was repeated 500 times, allowing histograms of p‐values to be constructed, as well as to estimate the variability around the heights of the histogram bars. Confidence intervals (95%) were computed for the empirical error rates and power using the Wilson method [Wilson, 1927]. The p‐values were also compared using Bland–Altman plots [Bland and Altman, 1986], modified so as to include the confidence intervals around the means of the methods.

### Performance of Combined Tests

We also took the opportunity to compare the combining functions shown in Table 1. While other comparisons have been made in the past (for a list of references, see Appendix A), none included all these functions, nor explored their performance under permutation or npc, and therefore, did not consider the modifications that we introduce to the procedure to render it feasible for imaging applications. In addition, we investigate the performance of two classical multivariate tests, the Hotelling's $T^{2}$, and the Wilks’ λ, both assessed through permutations.

**Figure 4:**
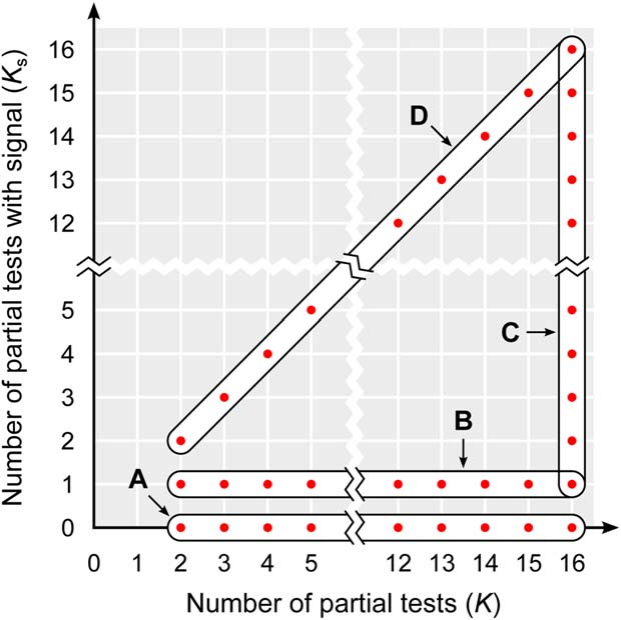
The simulations a–d. Each was constructed with a set of *K* partial tests, a number of which (*K_s_*) had synthetic signal added. [Color figure can be viewed in the online issue, which is available at http://wileyonlinelibrary.com.]

Four different simulation sets were conducted, named a–d; in all, the number of partial tests being combined could vary in the range K=2,…,16, and the number of partial tests containing true, synthetic signal could vary in the range $K_{s}=0,\ldots ,K$. In simulation a, K varied, while $K_{s}$ was held fixed at 0, that is, no synthetic signal was added. In simulation b, K varied, while $K_{s}$ was held fixed at 1, that is, just one partial test had signal added. In simulation c, K was held fixed at 16, while $K_{s}$ varied. Finally, in simulation d, K varied, and $K_{s}$ was set as equal to K, that is, all partial tests had synthetic signal added. Figure 4 shows graphically how K and $K_{s}$ varied in each simulation.

The response variables Y had size N×K, N=20, that is, simulating measurements for 20 subjects, each with K image modalities (partial tests). Each modality was simulated as having 500 points, these representing, for instance, voxels or vertices. The errors were simulated following either a Gaussian distribution with zero mean and unit variance, or a Weibull distribution, with scale parameter 1 and shape parameter $\frac{1}{3}$, shifted and scaled so as to have expected zero mean and unit variance. The response data were constructed by adding to the errors the simulated effects — either no signal, or a signal with strength calibrated to yield an approximate power of 50% with Gaussian errors, irrespective of the sample size, as described above for the simulations that tested the validity of the modified npc; for the Weibull errors, the signal was further decreased, in all these four simulations, by a factor $\frac{5}{8}$, thus minimising saturation at maximum power in simulation d. The actual effect was coded in the first regressor only, which was constructed as a set of random values following a Gaussian distribution with zero mean and unit variance; the second regressor was modelled as an intercept.

The simulated data was tested using 500 shufflings (permutations, sign‐flippings, and permutations with sign‐flippings). For all the simulations, the whole process was repeated 100 times, allowing histograms of p‐values to be constructed, as well as to estimate the variability around the heights of the histogram bars. Confidence intervals (95%) were computed for the empirical error rates and power using the Wilson method.

### Example: Pain Study

While the proposed correction for the mtp‐ii has a predictable consequence, that is, controlling the familywise error rate at the nominal level, the combination of modalities, designs, and contrasts may not be quite as obvious. In this section we show a re‐analysis of the data of the pain study by Brooks et al. [2005]. In brief, subjects received, in separate tests, painful, hot stimuli in the right side of the face (just below the lower lip), dorsum of the right hand, and dorsum of the right foot. The objective was to investigate somatotopic organization of the pain response in the insular cortex using fmri, and the complete experimental details, stimulation and imaging acquisition protocols, analysis and conclusions can be found in the original publication. Here we sought to identify, at the group level, in standard space, areas within the insula that jointly respond to hot painful stimuli across the three topologically distinct body regions. We used the modified npc, comparing the combining functions of Tippett, Fisher, Stouffer and Mudholkar–George, as well as the Hotelling's $T^{2}$ statistic, and an iut (conjunction). At the group level, the design is a one‐sample *t*‐test, for which only sign flippings can be used to test the null hypothesis. We used twelve of the original subjects, and performed exhaustively all the 4096 sign flippings possible.

## RESULTS

A large number of plots and tables were produced and are shown in the Supporting Information. The Figures below contain only the most representative results that are sufficient to highlight the major points.

### Validity of the Modified NPC

**Figure 5:**
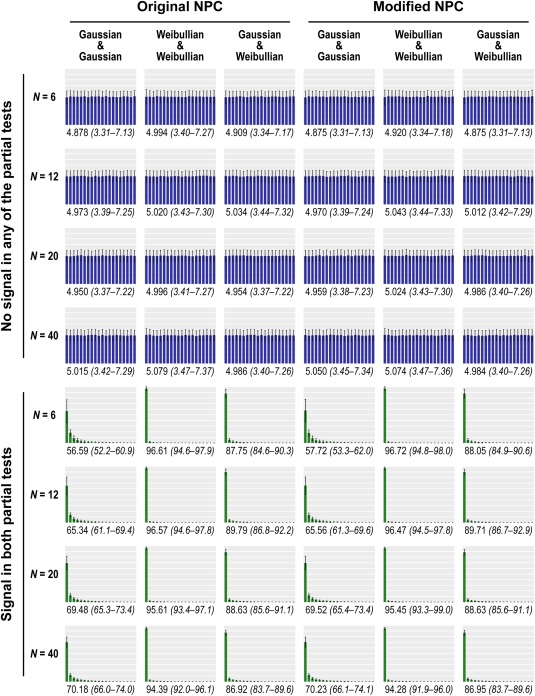
Histograms of frequency of p‐values for the simulation without signal in either of the two partial tests (upper panel, blue bars) or with signal in both (lower panel, green bars). The values below each plot indicate the height (in percentage) of the first bar, which corresponds to p‐values smaller than or equal to 0.05, along with the confidence interval (95%, italic). Both original and modified npc methods controlled the error rates at the nominal level, and produced flat histograms in the absence of signal. The histograms suggest similar power for both approaches. See also the Supporting Information. [Color figure can be viewed in the online issue, which is available at http://wileyonlinelibrary.com.]

Both the original and the modified npc methods controlled the error rates at exactly the level of the test. Such validity was not limited to α=0.05, and the histograms of uncorrected p‐values under complete absence of signal were flat throughout the whole [0;1] interval for both the original and modified npc methods, using either the Tippett or the Fisher combining functions. A representative subset of the results, for the Fisher method only, and for sample sizes N={ 8, 12, 20, 40 }, is shown in Figure 5.

**Figure 6:**
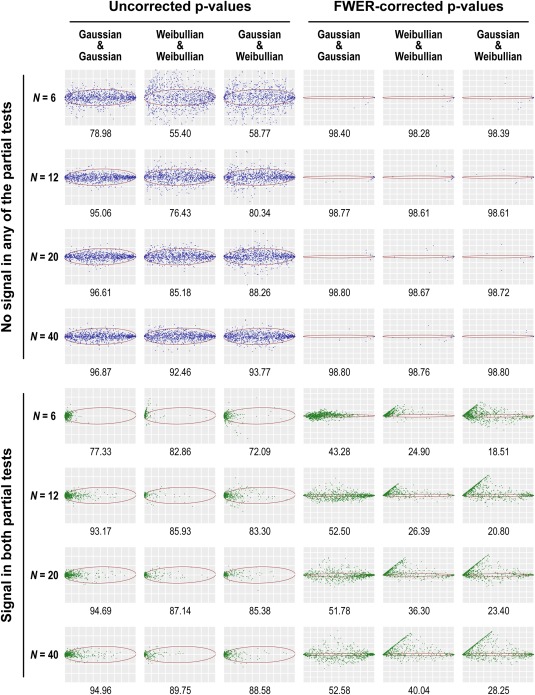
Bland–Altman plots comparing the original and modified npc, for both uncorrected and corrected p‐values, without signal in either of the two partial tests (upper panel, blue dots) or with signal in both (lower panel, green dots). The values below each plot indicate the percentage of points within the 95% confidence interval ellipsoid. For smaller sample sizes and non‐Gaussian error distributions, the methods differ, but the differences become negligible as the sample size increases. In the presence of signal, the modification caused increases in power, particularly for the corrected p‐values, with dots outside and above the ellipsoid. See the Supporting Information for zoomed in plots, in which axes tick labels are visible. [Color figure can be viewed in the online issue, which is available at http://wileyonlinelibrary.com.]

When considering the uncorrected p‐values, the modified npc yielded a mostly negligible increase in power when compared with the original npc, with the difference always within the 95% confidence interval. Although this slight gain can be hardly observed in the histograms and Bland–Altman plots for the uncorrected p‐values, they are clearly visible in the Bland–Altman plots for the p‐values corrected across the 500 tests. In these plots, the predominance of smaller (towards more significant) p‐values can be seen as a positive difference between the original and modified npc p‐values. A representative subset of the results is shown in Figure 6.

### Performance of Combined Tests

**Figure 7:**
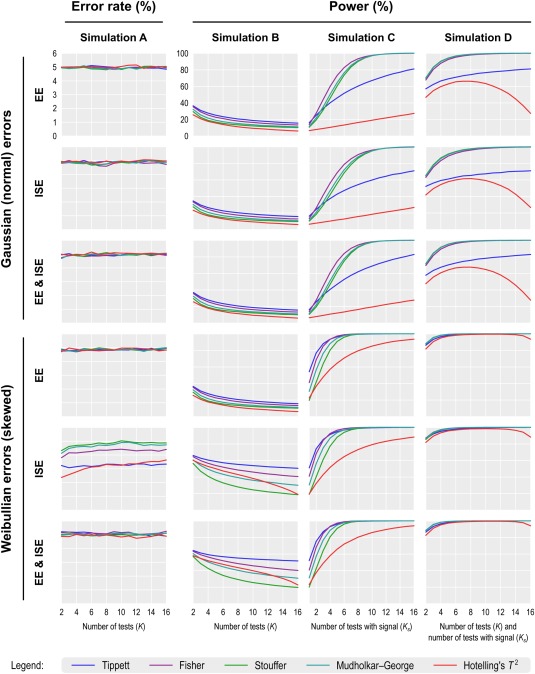
Performance of the modified npc with four representative combining functions (Tippett, Fisher, Stouffer, and Mudholkar–George) and of one cmv (Hotelling's *T* ^2^), using normal or skewed errors, and using permutations (ee), sign flippings (ise), or both. All resulted in error rates controlled at or below the level of the test. The Tippett and Fisher were generally the most powerful, with Tippett outperforming others with signal present in a small fraction of the tests, and with Fisher having the best power in the other settings. [Color figure can be viewed in the online issue, which is available at http://wileyonlinelibrary.com.]

Representative results demonstrating the performance of the methods of Tippett, Fisher, Stouffer, Mudholkar–George, as well as Hotelling's $T^{2}$, is shown in Figure 7. The remaining results are browsable in the Supporting Information. In the absence of signal (simulation a), all combining functions controlled the error rate at the level of the test or below it, never above, thus confirming their validity. With normally distributed (Gaussian) errors, most functions yielded uniformly distributed p‐values, although some functions seemed to converge towards uniformity only as the number of partial tests is increased; this was the case for the methods of Wilkinson, Zaykin, Dudbridge–Koeleman (dtp) and Jiang. With skewed (Weibullian) errors, the error rate was controlled at the test level with the use of permutations; with sign‐flippings or permutations with sign‐flippings, the combined results tended to be conservative, and more so for the Hotelling's $T^{2}$ statistics (and likewise the Wilks’ λ).

With signal added to just one of the partial tests (simulation b), the method of Tippett was generally the most powerful, followed by the methods of Fisher and Dudbridge–Koeleman (both rtp and dtp variants). As the number of tests was increased, predictably, the power was reduced for all tests. The method of Stouffer did not in general have good performance with skewed errors, presumably because the dependence on z‐statistics strengthens the dependence on the assumption of normality of the statistics for the partial tests in the modified npc. The cmv did not deliver a good performance either, being generally among the least powerful.

With the number of partial tests held fixed, as the number of tests with signal was increased (simulation c), the power of the method of Fisher increased more quickly than of the other methods, although when most of the partial tests had signal, most of the combining functions reached similar power, all close to 100% for both normal or skewed errors. Hotelling's $T^{2}$ test was considerably less powerful than any of the combining functions used with the modified npc.

As the total number of partial tests and the number of partial tests with signal were both increased (simulation d), almost all combined tests had similar power, and reached saturation (100% power) quickly, particularly for the Weibullian errors, in which the calibration, even after reduction with the $\frac{5}{8}$ factor, yielded power above 50% for each partial test. With Gaussian errors, in which calibration ensured average 50% power, two tests had considerably lower sensitivity: Tippett's and Hotelling's $T^{2}$, the last with the remarkable result that power reached a peak, then began to fall as the number of tests kept increasing.

### Example: Pain Study

Using a conventional, mass univariate voxelwise tests, assessed through sign flippings, and after correction for multiple testing (mtp‐i), only a few, sparse voxels could be identified at the group level for face, hand, and foot stimulation separately, in all cases with multiple distinct foci of activity observed bilaterally in the anterior and posterior insula. However, the joint analysis using the modified npc with Fisher, Stouffer and Mudholkar–George evidenced robust activity in the anterior insula bilaterally, posterior insula, secondary somatosensory cortex (sii), and a small focus of activity in the midbrain, in the periaqueductal gray area. The combining function of Tippett, however, did not identify these regions, presumably because this method is less sensitive than the others when signal is present in more than a single partial test, as suggested by the findings in the previous section.

The Hotelling's $T^{2}$ was not able to identify these regions, with almost negligible, sparse, single‐voxel findings in the anterior insula, bilaterally. The conjunction test, that has a different jnh, and searches for areas where all partial tests are significant, identified a single, barely visible, isolated voxel in the right anterior insula.

**Figure 8:**
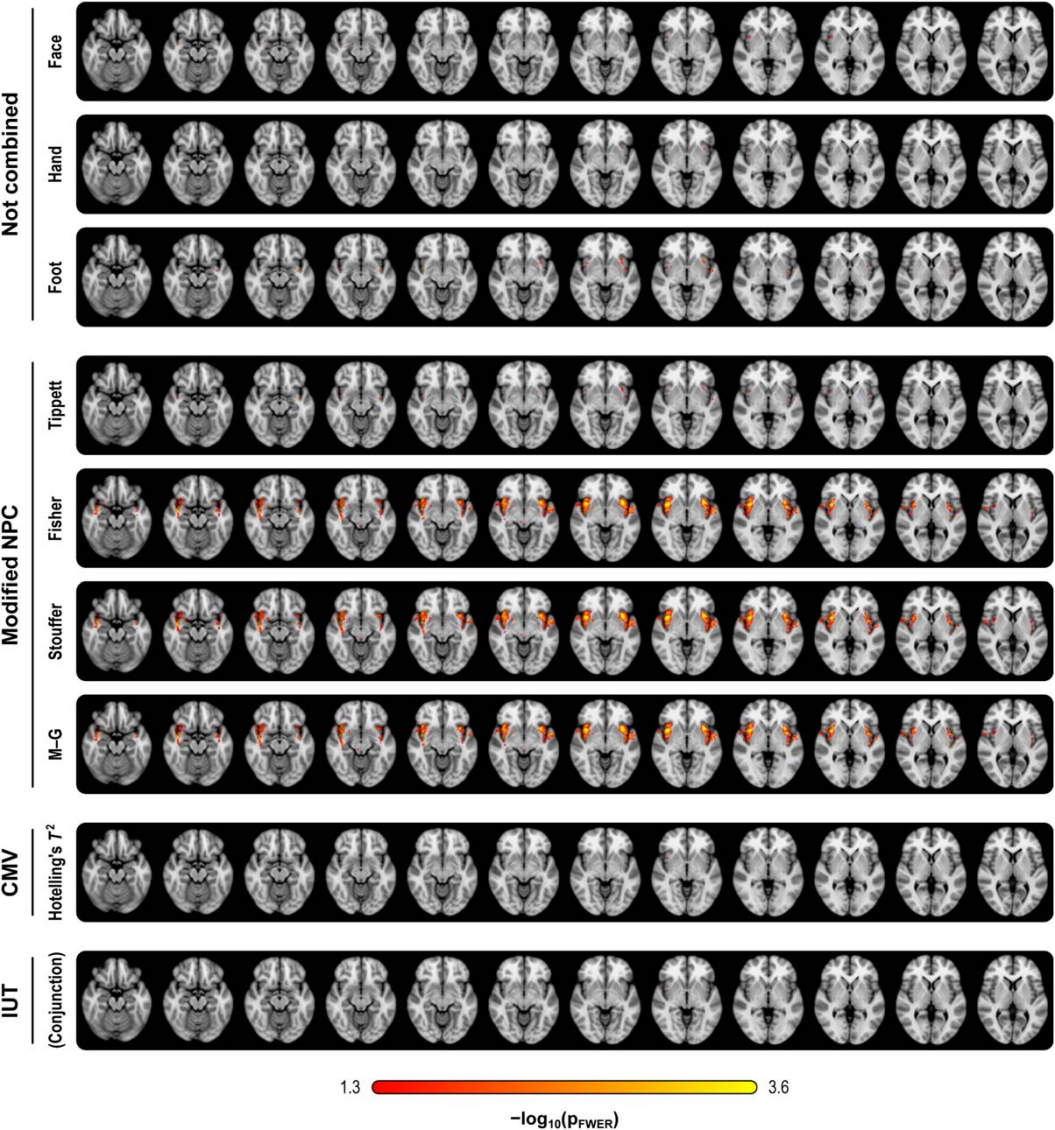
Without combination, and with correction across voxels (mtp‐i), no significant results were observed at the group level for any of the three tests. Combination using the methods of Fisher, Stouffer and Mudholkar–George (M–G), however, evidenced bilateral activity in the insula in response to hot, painful stimulation. A classical multivariate test, Hotelling's *T* ^2^, as well as the Tippett method, failed to identify these areas. An intersection‐union test (conjunction) could not locate significant results; such a test has a different null hypothesis that distinguishes it from the others. Images are in radiological orientation. For cluster‐level results, comparable to Brooks et al. [2005], see the Supporting Information. [Color figure can be viewed in the online issue, which is available at http://wileyonlinelibrary.com.]

The above results are shown in Figure 8. Cluster‐level maps that can directly be compared to the original findings of Brooks et al. [2005] are shown in the Supporting Information.

## DISCUSSION

### Validity of the Modified NPC

The modified npc combines u‐values, which are simply parametric p‐values here renamed to avoid confusion. The renaming, however, emphasizes the fact that the conversion to u‐values via a parametric approximation should only be seen as a data transformation, in which the interpretation as a p‐value is not preserved because of unsound assumptions. The combination method continues to be non‐parametric as the combined statistic is assessed non‐parametrically. More importantly, irrespective of the validity of parametric assumptions, any dependence between the tests is accounted for, implicitly, by the combination procedure, without the need of any modelling that could, at best, introduce complex and perhaps untenable assumptions, and at worst, be completely intractable.

The results suggest that, even in the cases in which the modified npc could have failed, i.e., with small sample sizes and different distributions, the combined statistic controlled the error rate at the level of the test. This control, maintained even in such difficult scenarios, supports the notion that the modified npc controls the error rates in general. The results also suggest that the modification increases power, even if such increase is minute in some scenarios. The Bland–Altman plots indicate that gains in sensitivity are more pronounced in the results corrected for the mtp‐i, suggesting that the modified method is appropriate not merely due to its expediency for imaging applications, but also for having increased sensitivity compared to the original npc.

### Performance of Combined Tests

The results also demonstrate that the npc method is more powerful than the Hotelling's $T^{2}$. The superiority of combined permutation tests when compared with classical multivariate tests has been observed in the literature [Blair et al., 1994], and the fact that power increases as the number of partial tests with signal increases is one of its most remarkable features. While cmv depends on the positive‐definiteness of the covariance matrix of the vectors of residuals, such limitation does not apply to npc [Pesarin and Salmaso, 2010b]. As a consequence, although in the comparisons only the Hotelling's $T^{2}$ and the Wilks’ λ statistics were used (in the simulations, $rank\left(C\right)=1$), and had their p‐values assessed through permutations, similar behavior can be expected when using other cmvs, such as Pillai's trace (and with $rank\left(C\right)>1$). With effect, npc can be used even when the number of variables equals or even greatly exceeds the number of observations, that is, when K≥N. In the results shown in Figure 7, this can be noted as a reduction in power that can be seen with the Hotelling's $T^{2}$, particularly for simulation d, and this is the case even considering that the test is assessed through permutations.

Regarding the different combining functions, the simulations show that the method of Tippett is the most powerful when signal is present in only a small fraction of the partial tests. For other cases, other combining functions, particularly that of Fisher, tend to be considerably more powerful.

The results also indicate that the use of sign flipping when the errors are not symmetric (a violation of assumptions) tends to produce a conservative test, with error rates below the nominal level, even if the power eventually remained unaltered when compared with permutations. While permutations together with sign flippings did alleviate conservativeness, at least for the Tippett method, the error rate remained below the nominal level. In general, if the errors are known to be skewed, only permutations should be used; if sign flippings are used, the error rate can be expected to be below the test level.

### Interpretation of Combined Tests

The key aspect of the npc is that these tests seek to identify, on the aggregate of the partial tests, a measure of evidence against the jnh, even if only some or none of them can be considered significant when seen in isolation, just as originally pointed out by Fisher [1932]:

*When a number of quite independent tests of significance have been made, it sometimes happens that although few or none can be claimed individually as significant, yet the aggregate gives an impression that the probabilities are on the whole lower than would often have been obtained by chance. It is sometimes desired (…) to obtain a single test of the significance of the aggregate*.

This is the logic and interpretation of all of these combining statistics, with the exception of the conjunction inference. Combination of information is known to be able to answer questions that could otherwise not be answered be at all, or be answered less accurately if each information source were considered separately [Draper et al., 1992]. Here the simulations and the pain study exemplify these aspects, and the improved sensitivity compared to each partial test when seen in separate.

As they depend on fewer assumptions than classical multivariate tests, npc can be considered whenever the validity of the former cannot be guaranteed. Even when parametric cmv assumptions hold, note that the npc can have superior power when sample size is small and prevents precise estimation of a covariance.

It should be noted that the aggregation of information follows a different principle than using different measurements separately to interrogate particular aspects of the brain (or of any other experiment or physiological phenomenon). Used judiciously, npc provides a complete framework that can be used for both the aggregate and for the correction of tests separately, with the valuable feature of being based on minimal assumptions.

### Correction over Contrasts and over Modalities

Correction over contrasts using synchronized permutations provides a novel solution to the multiple comparisons problem for certain common experimental designs, in particular, for the popular one‐way anova layout, that is, when the means of multiple groups are compared. The classical Fisher's protected least significant difference (lsd), that consists of performing an omnibus F‐test and only proceeding to the group‐wise post hoc tests if this initial test is significant, is known to fail to control the error rate if there are more than three groups [Hayter, 1986; Hsu, 1996; Meier, 2006], and the failure can be by a wide margin, that grows as the number of groups being compared increases. Even though the same may not happen with other correction methods [e.g., Tukey's range test, Tukey, 1949], the correction done non‐parametrically also renders these older, parametric methods, redundant.

The correction over contrasts further obviates methods that are based on what has been termed “logical constraints” among hypotheses [Hochberg and Tamhane, 1987; Shaffer, 1986], as the dependencies among the tests are implicitly taken into account by the correction using the distribution of the extremum across contrasts, with or without concomitant combination or correction across multiple K variables. In fact, the use of an omnibus F‐test as a way to guard against multiple testing becomes quite unnecessary.

In the same manner, while combination across multiple modalities is a powerful substitute for classical multivariate tests as shown earlier, the correction across such modalities can replace the post hoc tests that are usually performed after significant results are found with cmvs.

### Pain Study

Joint significance is an important consideration when trying to interpret data such as these, that are distinct in some aspects (here, the topography of the stimulation), but similar in others (here, the type of stimulation, hot and painful), strengthening the case for distinct representations in some brain regions, but not in others. In terms of identifying areas with significant joint activity, the results suggest involvement of large portions of the anterior insula and secondary somatosensory cortex. The Fisher, Stouffer and Mudholkar–George combining functions were particularly successful in recovering a small area of activity in the midbrain and periaqueductal gray area that would be expected from previous studies on pain [Petrovic et al., 2002; Reynolds, 1969; Roy et al., 2014; Tracey et al., 2002], but that could not be located from the original, non‐combined data.

### Relationship with Meta‐Analysis

Most of the combining functions shown in Table 1 were originally defined based on p‐values, and some of them are popular in meta‐analyses, such as those of Fisher and Stouffer [Borenstein et al., 2009]. Although there are commonalities between these meta‐analytical methods and npc, it is worth emphasising that the two constitute distinct approaches to entirely different problems. In the npc, the objective is to interrogate joint significance across the multiple observed variables (or multiple designs and contrasts if these are instead combined) when the data for each individual observation is readily available to the researcher. Meta‐analyses methods based on p‐values, while sometimes using the same combining functions, attempt to identify a joint effect across multiple studies that not have necessarily been performed on the same experimental units, and when the data for the individual observations are not available. Moreover, the p‐value of the combined statistic in the npc is produced through permutations, a procedure that is not available for ordinary meta‐analytical methods.

The fact that npc and meta‐analysis form different approaches to separate problems also imply that certain criticisms levelled at the use of certain combined functions in the context of meta‐analysis do not extend trivially to npc. As the simulations show, various of the combining functions more recently developed did not in general outperform older combining methods, such as Fisher and Stouffer, even though these were developed precisely for that purpose, in the context of meta‐analyses, or for problems framed as such.

## CONCLUSION

We proposed and evaluated a modified version of Non‐Parametric Combination that is feasible and useful for imaging applications, and serves as a more powerful alternative to classical multivariate tests. We presented and discussed aspects related multiple testing problems in brain imaging, and proposed a single framework that addresses all these concerns at once. We showed that combination and correction of multiple imaging modalities, designs, and contrasts, are related to each other in the logic of their implementation, and also through the use of the simplest and the oldest of the combining functions, attributed to Tippett.

An open‐source working implementation, that can be executed in Matlab [The MathWorks Inc., 2013] or Octave [Eaton et al., 2015], is available in the tool Permutation Analysis of Linear Models (palm), available for download at http://www.fmrib.ox.ac.uk/fsl.

## Supporting information

Supporting Information

## Figures and Tables

**Figure B1:**
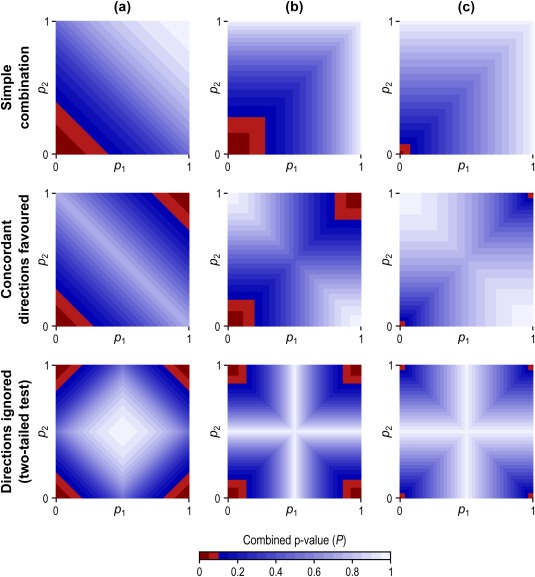
Examples of inconsistent combining functions for testing the global null hypothesis: (a) Addition of p‐values for the partial tests [Edgington 1972]; (b) Maximum of p‐values for the partial tests, with the p‐value computed as *T^K^* [Friston et al., 2005]; (c) Maximum of p‐values for the partial tests, but with the p‐value computed as *T* [Nichols et al., 2005]. While the last is not appropriate for testing the global null, it is appropriate for the conjunction null. [Color figure can be viewed in the online issue, which is available at http://wileyonlinelibrary.com.]

**Figure C1:**
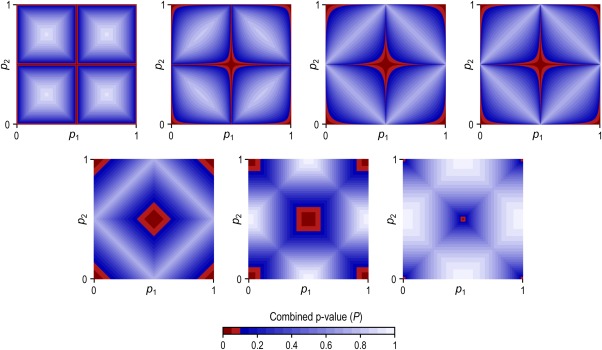
Upper row: Inadmissible versions of the four consistent combining functions shown in Figure 3 (in the same order). Lower row: Inadmissible versions of the three inconsistent combining functions shown in Figure 9 (in the same order). These inadmissible functions arise if one attempts to favor alternatives with the same sign while performing two‐tailed partial tests. [Color figure can be viewed in the online issue, which is available at http://wileyonlinelibrary.com.]

## Footnotes

1 From the Pascal triangle: ∑i=1KKi=2K−1.

## APPENDIX A BRIEF OVERVIEW OF COMBINING FUNCTIONS

Below are a few details and references for the methods shown in Table 1, plus a few others, presented in chronological order. A number of studies comparing some of these functions in various scenarios have been published [Berk and Cohen, 1979; Bhandary and Zhang, 2011; Birnbaum, 1954; Chang et al., 2013; Chen, 2011; Lazar et al., 2002; Loughin, 2004; Oosterhoff, 1969; Rosenthal, 1978; Westberg, 1985; Whitlock, 2005; Won et al., 2009; Wu, 2006; Zaykin, 2011; Zwet and Oosterhoff, 1967]. Some of these are permutationally equivalent to each other, that is, their rejection region under permutation is the same, and it becomes immaterial which is chosen.

### Tippett

This is probably the oldest, the simplest, and the most intuitive of the combination methods, having appeared in the first edition of Tippett's book *The Methods of Statistics* [Tippett, 1931]. The combined test statistic is simply the minimum p‐value across all partial tests, and Tippett shows its distribution has a simple closed form.

### Fisher

This method appeared in the fourth edition of *Statistical Methods for Research Workers* [Fisher, 1932], and follows the idea of treating the joint probability as the intersection of all partial tests, which is given by their product $\prod_{k}p_{k}$. This product, however, is not uniformly distributed, even if the global null hypothesis is true. Using a few properties of the uniform distribution, Fisher showed that twice the negative logarithm of the products follows a $\chi^{2}$ distribution, with degrees of freedom 2K.

### Stouffer

This method appeared in footnotes in the extensive report of the sociological study conducted among veterans of the World War ii by Stouffer et al. [1949, footnote 15, and page 151, footnote 14]. The idea is to sum z‐scores, normalize the variance of this sum, and from this statistic obtain a p‐value for the joint hypothesis.

### Wilkinson

The probability of observing r significant p‐values at the level α can be computed using a binomial expansion, as proposed by Wilkinson [1951]. The statistic is simply r, and the probability does not depend on the actual p‐values for the partial tests, but only on r and α.

### Good

A generalization of the Fisher method that assigns arbitrary, unequal positive weights $w_{k}$ for each of the partial tests, was suggested by Good [1955]. The weights are defined according to some criteria, such as the sample size for each of the partial test, the number of degrees of freedom, or some other desirable feature, such as ecological or internal validity [Rosenthal, 1978].

### Lipták

Another generalized combined statistic can be produced using the inverse cdf, $F^{-1}$, of the $p_{k}$, summing the values of the statistics, and computing a new p‐value for the global null using the cdf G of the sum of the statistics, a method proposed by Lipták [1958]. Each summand can be arbitrarily weighted, as in the Good method. In principle, any continuously increasing function with support in the interval [0; 1] can be used for F, albeit a more obvious choice is the cdf of the normal distribution, which can be used as both F and G, and which equals the approach to the Stouffer method if all weights are 1.

### Lancaster

While the Lipták method generalizes combining strategies such as Fisher and Stouffer, the Lancaster method [Lancaster, 1961] further generalizes the Lipták approach by allowing different $F_{k}^{-1}$ for each partial test. Choices for $F_{k}^{-1}$ include, for instance, the cdf of the gamma distribution with scale parameter θ=2, possibly with different shape parameters taking the place of the weights for each partial test. If the weights are all positive integers, the p‐values can be assessed from the cdf of a $\chi^{2}$ distribution with degrees of freedom $\nu =2\sum_{k}w_{k}$ [Berk and Cohen, 1979].

### Winer

A combination strategy that resembles the Stouffer method, but uses the Student's t statistic, was proposed by Winer [1962], albeit not found in later editions of the book. The idea is to sum the t statistics for all the partial tests, then normalize the sum so that the resulting statistic follows a standard normal distribution. The normalization is based on the fact that the variance of the t distribution can be determined from its degrees of freedom ν as ν/(ν−2). The method cannot be applied if $\nu_{k}\leq 2$ for any of the partial tests. Moreover, $\nu_{k}$ should not be too small for the normal approximation to be reasonably valid (e.g., $\nu_{k}\geq 10$). The Winer method is a particular case of the Lancaster method.

### Edgington

The probability of observing, due to chance, a value equal or smaller than the sum of the partial p‐values was proposed by Edgington [1972] as what would be a more powerful alternative to the Fisher method. The method however, lacks consistency (see Appendix B).

### Mudholkar–George

It is possible to use a simple logit transformation to compute a statistic that approximates a scaled version of the Student's t distribution, as shown by Mudholkar and George [1979]. If the scaling is taken into account, the combined statistic follows a t distribution.

### Darlington–Hayes

In a discussion about pooling p‐values for meta‐analysis, Darlington and Hayes [2000] raised a number of limitations of these methods, and proposed a modification over the method of Stouffer that would address some of these concerns. The modified method, called by the authors as *Stouffer‐max*, uses as test statistic the mean of the r highest z‐scores, rather than the normalized sum of all the z‐scores as in the original method. When r=1, it is equivalent to the Tippett method, whereas when r=K, is equivalent to the original Stouffer. The p‐values for intermediate values of r can be computed through Monte Carlo simulation, and the authors provided tables with critical values.

### Zaykin et al.

This method, called truncated product method (tpm) was proposed by Zaykin et al. [2002] as a way to combine features of the Fisher and Wilkinson methods. The statistic is the product of only the partial p‐values that are significant at the level α, whereas in the Fisher method, all p‐values are used. If $\alpha =min\left(p_{k}\right)$, the approach is equivalent to the Tippett method. If $max\left(p_{k}\right)\leq \alpha \leq 1$, the approach is equivalent to the Fisher method. An expression for the p‐values that produces exact values was provided by the authors. The expresion, however, is prone to over/underflows for certain combinations of large K and α, and when p‐values cannot be obtained analytically, Monte Carlo methods can be used.

### Dudbridge–Koeleman

While the Zaykin method combines only the partial tests that are significant at the level α, it is also possible to create a statistic that combines only the most r significant tests, where r is specified in advance. This method was proposed by Dudbridge and Koeleman [2003] and called rank truncated product (rtp). The main benefit of this strategy is that it depends only on a predetermined number of partial tests to be rejected, rather than on their p‐values, which are random quantities. As with the Zaykin method, for certain combinations of r and large K, the p‐values need to be computed through Monte Carlo methods. In the same article, the authors also introduced a combination of the tpm and rtp, and named it rank‐and‐threshold truncated product or dual truncated product (dtp). The statistic is the largest of either if these two, and its p‐value can be computed analytically or via Monte Carlo methods.

### Taylor–Tibshirani

If the p‐values are sorted in ascending order, these ranked p‐values can be compared to their expectations under the global null hypothesis. Large deviations from the expected values suggest the presence of the effect among the tests. Taylor and Tibshirani [2006] suggested that a measurement of this deviation could be used to infer the overall significance of the tests. The corresponding statistic was termed tail strength (ts), and under the assumptions that the global null is true and that the tests are independent, it follows a normal distribution with zero mean and a variance that can be approximated as $\frac{1}{K}$ for large K, from which the p‐value can be assessed. When these assumptions are not met, non‐parametric methods can be used.

### Jiang et al.

The statistic of the Taylor–Tibshirani method has a variance that depends asymptotically only on the number of tests. However, the value of the statistic can be small when effect is truly present in only a few partial tests, therefore potentially reducing power. By analogy to the Zaykin method, Jiang et al. [2011] proposed to compute the tail strength using only partial tests with p‐values smaller than a certain level α. The method is called truncated tail strength (tts). The analytical form for the distribution is not known, and the authors propose computing the p‐value using Monte Carlo or permutation methods.

### Li–Tseng

Li and Tseng [2001] proposed a modification of the Fisher method that is used not to test the jnh (hence not shown in Table 1), but to identify which of the partial tests contribute the most to the resulting combined statistic. The authors define a quantity $A_{W}=-\sum_{k=1}^{K}w_{k}ln(p_{k})$, where $w_{k}$ is a weight that can be either 0 or 1. All possible $2^{K}-1$ non‐trivial combinations $W=\left[w_{1},\ldots ,w_{K}\right]$ are evaluated to produce a value for $A_{W}$. The respective p‐values $p_{W}$ are computed via permutations, and the W that yields the smallest such p‐value over all possible combinations of weights, is the one that identifies the subset among the K tests that contributes the most to the combined p‐values.

## APPENDIX B CONSISTENCY OF COMBINED TESTS

A hypothesis test is said to be consistent if, for a fixed test level, its power goes to unity as the sample size increases to infinity. The use of a non‐consistent combining function to form an npc test is problematic, as the rejection region may not be reached even if the p‐value for one or more of the partial tests approach zero, thus violating the second of the three desirable properties of the combining functions, presented in Section “Non‐Parametric Combination”.

Among the functions shown in Table 1, the notable non‐consistent combining functions are the Edgington and Wilkinson (see Appendix A). Also, it should be noted that functions that define conjunctions (iut), such as those based on $max\left(p_{k}\right)$, are likewise not consistent in the context of npc, as the latter serves to test the global null hypothesis. Figure B1 shows rejection regions for some inconsistent combining functions, and variants, similarly as for the (consistent) shown in Figure 3.

## APPENDIX C ADMISSIBILITY OF COMBINED TESTS

A combined hypothesis test is said to be admissible if there exists no other test that, at the same significance level, without being less powerful to all possible alternative hypotheses, is more powerful to at least one alternative [Lehmann and Romano, 2005]. This can be stated in terms of either of two sufficient conditions for admissibility: (i) that rejection of the null for a given p‐value implies the rejection of the null for all other p‐values smaller or equal than that, or (ii) that the rejection region is convex in the space of the test statistic.

Combinations that favor tests with concordant directions (Section “Directed, non‐directed, and concordant hypotheses”), if used with of non‐directional partial tests, create tests that are inadmissible, that is, tests that are not optimal in the sense that there exist other tests that, without being less powerful to some true alternative hypotheses, are more powerful to at least one true alternative. Inadmissibility implies that the test cannot be used, as certain combinations of partial tests lead to nonsensical results, such as rejecting the jnh for some partial p‐values, and failing to reject for some p‐values that are even smaller. Figure C1 shows rejection regions of inadmissible versions of the combining functions considered in Figures 3 and B1; clearly none of the two conditions above are satisfied. The particular combining function shown in Equation (2) was suggested by Pearson [1933] and used by David [1934], but after a paper by Birnbaum [1954], it was for decades thought to be inadmissible. However, it is in fact admissible [Owen, 2009].

Admissibility is important in that it allows, for more than just two partial tests, combined tests that favor alternative hypotheses with the same direction. Other possibilities favoring alternatives with common direction, such as multiplying together the partial test statistics to produce a combined statistic, do not extend trivially to more than two tests [Hayasaka et al., 2006].
