# Supplementary material for: Non‐parametric combination and related permutation tests for neuroimaging
Source: Hum Brain Mapp. 2016 Feb 5;37(4):1486–511. doi: 10.1002/hbm.23115 (PMC4783210; doi:10.1002/hbm.23115)
Supplement: Supplementary file 1 — Supporting Information [file HBM-37-1486-s001.zip › supporting_information/algorithm.pdf]

Non-Parametric Combination and related permutation tests for neuroimaging*

Supporting Information: Implementation

Anderson M. Winkler, Matthew A. Webster, Jonathan C. Brooks, Irene Tracey, Stephen M. Smith, Thomas E. Nichols

A unified algorithm for combination and correction that is amenable for use with imaging applications is shown below. It has many similarities with the `randomise` algorithm (Winkler et al., 2014), with various modifications to accommodate combination and correction. The p-values adjusted for the multiplicity of tests are computed using the distribution of the extremum statistic, which can be collapsed across modalities and/or designs and contrasts for each case, rendering the algorithm simpler. The notation below is slightly different than that used throughout the paper. The inputs are:

- $\mathbf{Y}$: The input data for each of the K modalities and image points. Each column vector of N observations for the k-th modality is accessed as $\mathbf{Y}[k, \mathbf{v}]$, where $\mathbf{v} = [x, y, z]$ is used to specify the point position in space; this is so without loss of generality for non-imaging data.
- $\mathcal{X}$: The set of design matrices $\mathbf{X}$.
- $\{\mathcal{C}_\mathbf{X}\}$: The set of sets of contrasts for each design matrix $\mathbf{X}$. Each element of each subset is a pair of multivariate contrasts $(\mathbf{C}, \mathbf{D})$. This definition allows each design to be tested with multiple such pairs of contrasts, and allows various designs to be tested with the same input data.
- $\mathbf{B}$: Definition of multi-level exchangeability blocks, used to define valid shufflings that respect the data structure (Winkler et al., 2015).
- $\mathbf{V}$: Definition of the variance groups, useful to compute statistics that are robust to heteroscedasticity.
- EE, ISE: Boolean flags (true/false) indicating whether errors can be treated as exchangeable (EE), allowing permutations, independent and symmetric (ISE), allowing sign-flippings, or both.
- J: Number of permutations to be performed.

*Published in the journal *Human Brain Mapping*, 2016.

– NPCMOD, NPCCON: Boolean indicating whether combination should be performed respectively across modalities, across designs and contrasts, or both.

– FWEMOD, FWECON: Boolean indicating whether familywise error rate correction should be performed respectively across modalities, across designs and contrasts, or both.

The output of interest is the p-value. For simplicity, as shown, the output is always FWER-adjusted across the image points indexed by $\mathbf{v}$, and for the non-combined, further adjusted based on the contrasts and modalities; these are shown in the algorithm topped by a tilde, that is, as "p̃-value", as opposed to simply "p-value". Also for simplicity, p-values for combination of modalities are not shown adjusted for multiple contrasts, nor vice-versa. These can also be obtained following the same logic used for the FWER-adjustment of the non-combined statistics. Uncorrected p-values, useful for correction using false discovery rate (FDR, Benjamini and Hochberg, 1995) can be obtained with trivial modifications.

Algorithm 1: Unified algorithm. See the main text for details.

Require: $\mathbf{Y}, \mathcal{X}, \{\mathcal{C}_{\mathbf{X}}\}, \mathbf{B}, \mathbf{V}$, EE, ISE, J, NPCMOD, NPCCON, FWEMOD, FWECON.

$\mathcal{P} \leftarrow \mathsf{sync\_perms}(\mathcal{X}, \{\mathcal{C}_{\mathbf{X}}\}, \mathbf{B}, \text{EE}, \text{ISE}, J-1)$ ▷ Define the permutation set.
$\mathcal{P} \leftarrow \{\mathbf{I}, \mathcal{P}\}$ ▷ Ensure first permutation is no permutation.
for $j = 1, \ldots, J$ **do** ▷ For each shuffling.
  $c \leftarrow 1$ ▷ Counter for the number of designs and contrasts.
  for all $\mathbf{X} \in \mathcal{X}$ **do** ▷ For each design matrix.
    for all $(\mathbf{C}, \mathbf{D}) \in \mathcal{C}_{\mathbf{X}}$ **do** ▷ For each pair of contrasts.
      $\mathbf{Y} \leftarrow \mathbf{YD}$ ▷ Redefine the data, discard $\mathbf{D}$.
      $\mathbf{X}^* \leftarrow \mathbf{P}_j\mathbf{X}$ ▷ Shuffle the model.
      for all $k \in \{1, \ldots, K\}$ **do** ▷ For each partial test.
        for all $\mathbf{v}$ **do** ▷ For each image point.
          $\hat{\boldsymbol{\beta}} \leftarrow (\mathbf{X}^*)^+\mathbf{Y}[k, \mathbf{v}]$ ▷ Estimated regression coefficients.
          $\hat{\mathbf{E}} \leftarrow \mathbf{Y}[k, \mathbf{v}] - \mathbf{X}^*\hat{\boldsymbol{\beta}}$ ▷ Estimation residuals.
          $\mathbf{G} \leftarrow \mathsf{pivotal}(\mathbf{X}^*, \hat{\boldsymbol{\beta}}, \hat{\boldsymbol{\epsilon}}, \mathbf{C}, \mathbf{V})$ ▷ Test statistic.
          $\mathbf{U}[j, k, c, \mathbf{v}] \leftarrow \mathsf{transform}(\mathbf{G})$ ▷ Transform to u-value.
          if $j = 1$ **then** ▷ In the first permutation (no permutation).
            $\mathbf{U}_0[k, c, \mathbf{v}] \leftarrow \mathbf{U}[1, k, c, \mathbf{v}]$ ▷ Keep the unpermuted u-value.
          end if
        end for
        $\mathbf{U}_{\mathrm{e}}[j, k, c] \leftarrow \mathsf{extremum}(\mathbf{U}[j, k, c, \cdot])$ ▷ Extremum across space.
      end for
      $c \leftarrow c + 1$ ▷ Increment counter for the number of designs and contrasts.
    end for
  end for
  $C \leftarrow c$ ▷ Keep the total number of designs and contrasts for later use.

if NPCMOD $\wedge \neg$ NPCCON **then** ▷ Combine modalities only.
  for all $c \in \{1, \ldots, C\}$ **do** ▷ For each design/contrast.
    for all $\mathbf{v}$ **do** ▷ For each image point.
      $\mathbf{T}[c, \mathbf{v}] \leftarrow \mathsf{combine}(\mathbf{U}[j, \cdot, c, \mathbf{v}])$ ▷ Combined statistic.
    end for
    $\mathbf{T}_{\mathrm{e}}[j, c] \leftarrow \mathsf{extremum}(\mathbf{T}[c, \cdot])$ ▷ Distribution of the extrema across tests.
  end for
else if NPCCON $\wedge \neg$ NPCMOD **then** ▷ Combine designs/contrasts only.
  for all $k \in \{1, \ldots, K\}$ **do** ▷ For each design/contrast.
    for all $\mathbf{v}$ **do** ▷ For each image point.
      $\mathbf{T}[k, \mathbf{v}] \leftarrow \mathsf{combine}(\mathbf{U}[j, k, \cdot, \mathbf{v}])$ ▷ Combined statistic.
    end for
    $\mathbf{T}_{\mathrm{e}}[j, k] \leftarrow \mathsf{extremum}(\mathbf{T}[k, \cdot])$ ▷ Distribution of the extrema across tests.
  end for
else if NPCMOD $\wedge$ NPCCON **then** ▷ Combine modalities & designs/contrasts.
  for all $\mathbf{v}$ **do** ▷ For each image point.
    $\mathbf{T}[\mathbf{v}] \leftarrow \mathsf{combine}(\mathbf{U}[j, \cdot, \cdot, \mathbf{v}])$ ▷ Combined statistic.
  end for
  $\mathbf{T}_{\mathrm{e}}[j] \leftarrow \mathsf{extremum}(\mathbf{T}[\cdot])$ ▷ Distribution of the extrema across tests.
end if
if $j = 1$ **then** ▷ In the first permutation (no permutation).
  $\mathbf{T}_0 \leftarrow \mathbf{T}$ ▷ Keep the unpermuted combined statistic.
end if
end for
if NPCMOD $\wedge \neg$ NPCCON **then** ▷ Combine modalities only.
  for all $c \in \{1, \ldots, C\}$ **do** ▷ For each design/contrast.
    for all $\mathbf{v}$ **do** ▷ For each image point.
      $\text{p-value}[c, \mathbf{v}] \leftarrow \mathsf{data\_pval}(\mathbf{T}_0[c, \mathbf{v}], \mathbf{T}_{\mathrm{e}}[\cdot, c])$ ▷ Combined p-value.
    end for
  end for
else if NPCCON $\wedge \neg$ NPCMOD **then** ▷ Combine designs/contrasts only.
  for all $k \in \{1, \ldots, K\}$ **do** ▷ For each design/contrast.
    for all $\mathbf{v}$ **do** ▷ For each image point.
      $\text{p-value}[k, \mathbf{v}] \leftarrow \mathsf{data\_pval}(\mathbf{T}_0[k, \mathbf{v}], \mathbf{T}_{\mathrm{e}}[\cdot, k])$ ▷ Combined p-value.
    end for
  end for
else if NPCMOD $\wedge$ NPCCON **then** ▷ Combine modalities & designs/contrasts.
  for all $\mathbf{v}$ **do** ▷ For each image point.
    $\text{p-value}[\mathbf{v}] \leftarrow \mathsf{data\_pval}(\mathbf{T}_0[\mathbf{v}], \mathbf{T}_{\mathrm{e}}[\cdot])$ ▷ Combined p-value.
  end for
end if
if FWEMOD $\wedge \neg$ FWECON **then** ▷ Correct over modalities only.
  for all $c \in \{1, \ldots, C\}$ **do** ▷ For each design/contrast.
    for all $j \in \{1, \ldots, J\}$ **do** ▷ For each shuffling.
      $\mathbf{U}'_{\mathrm{e}}[j, c] \leftarrow \mathsf{extremum}(\mathbf{U}_{\mathrm{e}}[j, \cdot, c])$ ▷ Distribution of the extrema.
    end for
    for all $k \in \{1, \ldots, K\}$ **do** ▷ For each modality.
      for all $\mathbf{v}$ **do** ▷ For each image point.
        $\tilde{\text{p}}\text{-value}[k, c, \mathbf{v}] \leftarrow \mathsf{data\_pval}(\mathbf{U}_0[k, c, \mathbf{v}], \mathbf{U}'_{\mathrm{e}}[\cdot, c])$ ▷ Adjusted p-value.

```
        end for
      end for
    end for
  else if FWECON ∧ ¬ FWEMOD then                          ▷ Correct over designs/contrasts only.
    for all k ∈ {1, . . . , K} do                                     ▷ For each modality.
      for all j ∈ {1, . . . , J} do                                    ▷ For each shuffling.
        U'_e[j, k] ← extremum(U_e[j, k, ·])                   ▷ Distribution of the extrema.
      end for
      for all c ∈ {1, . . . , C} do                             ▷ For each design/contrast.
        for all v do                                                ▷ For each image point.
          p̃-value[k, c, v] ← data_pval(U_0[k, c, v], U'_e[·, k])     ▷ Adjusted p-value.
        end for
      end for
    end for
  else if FWEMOD ∧ FWECON then                   ▷ Correct over modalities & des./contr.
    for all j ∈ {1, . . . , J} do                                      ▷ For each shuffling.
      U'_e[j] ← extremum(U_e[j, ·, ·])                        ▷ Distribution of the extrema.
    end for
    for all k ∈ {1, . . . , K} do                                     ▷ For each modality.
      for all c ∈ {1, . . . , C} do                             ▷ For each design/contrast.
        for all v do                                                ▷ For each image point.
          p̃-value[k, c, v] ← data_pval(U_0[k, c, v], U'_e[·])        ▷ Adjusted p-value.
        end for
      end for
    end for
  end if
```

Within the algorithm, the functions are:

- *sync_perms*: This function produces a set $\mathcal{P}$ of permutation and/or sign flipping matrices that can be performed sychronously to test a joint null hypotheses about the input data. The synchronisation is always necessary to allow combination/correction over modalities, and it may also be necessary across multiple designs and/or contrasts if these are to be combined/corrected as well. If synchronisation is not necessary for designs and/or contrasts, the algorithm can be modified so that $\mathcal{P}$ can be defined inside the for-loops that iterate over designs and contrasts.

- *transform*: This converts the test statistic into a u-value, thus rendering the NPC method feasible for imaging applications. If no combination is to be performed, the algorithm can be modified to skip this step and work directly with the test statistic.

- *extremum*: For statistics in which larger values are evidence against the null hypothesis, this function takes the maximum. For statistics in which smaller values are indication against the null, this takes the minimum. In

either case, it is always the most extreme towards evidence favouring the alternative. This function effectively implements a CTP using an IUT.

– *combine*: This combines the inputs (p- or u-values) into a new, combined statistic. Any of the combining functions from Table 1 (in the main text) can be considered. For the method of Tippett, *combine* and *extremum* are the same.

– *data_pval*: This function produces a p-value based on a set of empirical values for the test statistic after shuffling. This works by computing the fraction of the test statistics after shuffling that is larger or equal than the unpermuted test statistic, while taking care of ties.

The algorithm has four major parts: the first consists of the loop that begins in line 5 of the pseudocode above, and which consists of a simplified version of the `randomise` algorithm. The second begins with the conditional structure in line 27, which performs the combination and computes distribution of the extremum statistics for each case of NPC, thus also treating the MTP-I. These initial two parts are repeated for each shuffling, in the loop that begins in line 3. The third part begins with the conditional in line 51, that is, once all rearrangements have been performed; in this part, the distributions are used to compute the combined p-values. Finally, the fourth part begins with the conditional in line 68, in which the MTP-II is addressed.

As shown, the algorithm is simplified so as to emphasise the most important aspects of combination and correction. However, various modifications and improvements can be applied for particular circumstances, and for speed, including the partitioning discussed in the Appendix A of Winkler et al. (2014). An open-source working implementation, that can be executed in MATLAB (The MathWorks Inc., 2013) or Octave (Eaton et al., 2014), is available in the tool *Permutation Analysis of Linear Models* (PALM), available for download at `www.fmrib.ox.ac.uk/fsl`.

References

Benjamini, Y., Hochberg, Y., 1995. Controlling the false discovery rate: a practical and powerful approach to multiple testing. Journal of the Royal Statistical Society. Series B (Methodological) 57 (1), 289–300.

Eaton, J. W., Bateman, D., Hauberg, S., 2014. GNU Octave: A high-level interactive language for numerical computations. Network Theory Ltd.
URL http://www.gnu.org/software/octave

The MathWorks Inc., 2013. MATLAB Version 8.1 (R2013a). Natick, Massachusetts.

Winkler, A. M., Ridgway, G. R., Webster, M. A., Smith, S. M., Nichols, T. E., 2014. Permutation inference for the general linear model. NeuroImage 92, 381–97.

Winkler, A. M., Webster, M. A., Vidaurre, D., Nichols, T. E., Smith, S. M., 2015. Multilevel block permutation. NeuroImage 123, 253–68.
