# Supplementary figures and images for: Non‐parametric combination and related permutation tests for neuroimaging (part 2 of 2)
Source: Hum Brain Mapp. 2016 Feb 5;37(4):1486–511. doi: 10.1002/hbm.23115 (PMC4783210; doi:10.1002/hbm.23115)

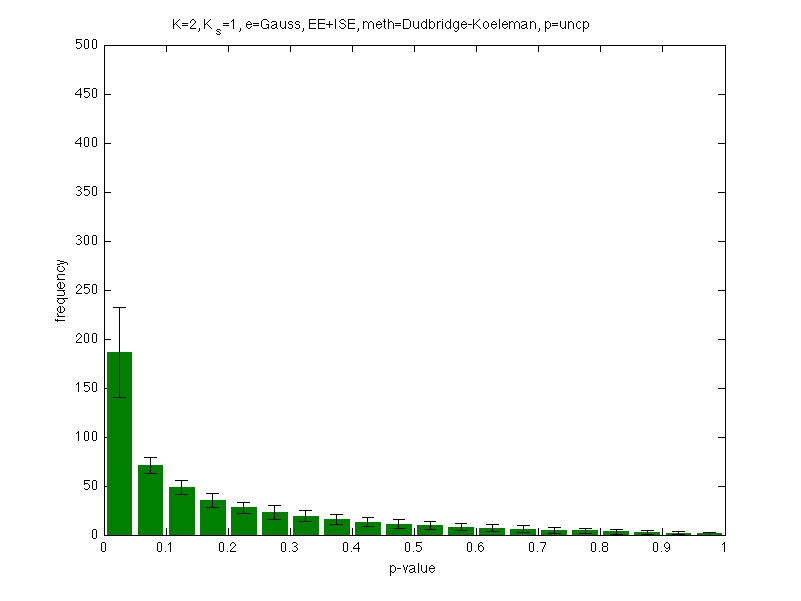

Supplement: Supplementary file 1 — Supporting Information [file HBM-37-1486-s001.zip › supporting_information/errorrates/histograms/images/hist_kset02_eGauss_ee+ise_npc_dudbridge-koeleman_uncp.png]

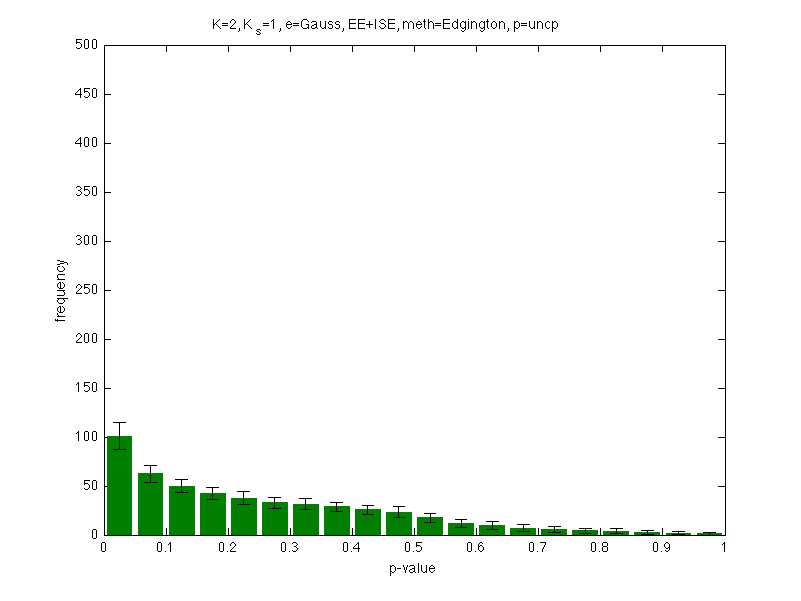

Supplement: Supplementary file 1 — Supporting Information [file HBM-37-1486-s001.zip › supporting_information/errorrates/histograms/images/hist_kset02_eGauss_ee+ise_npc_edgington_uncp.png]

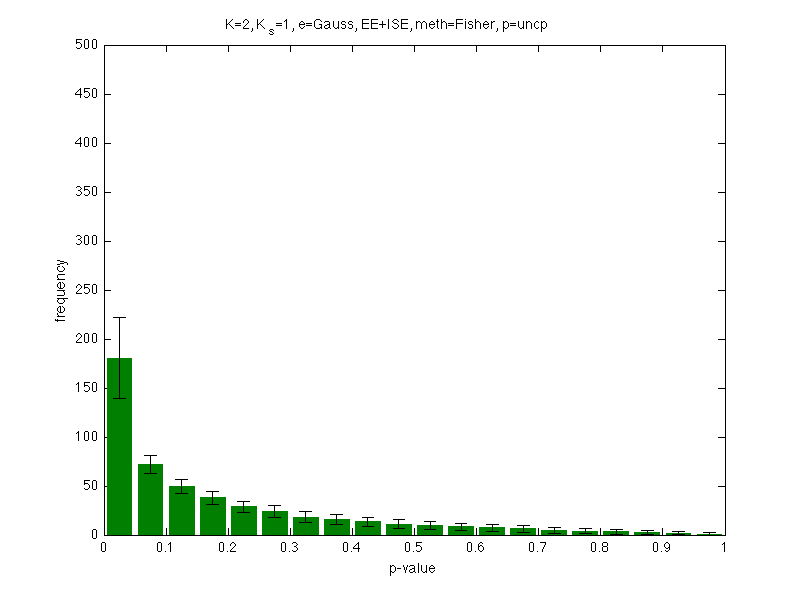

Supplement: Supplementary file 1 — Supporting Information [file HBM-37-1486-s001.zip › supporting_information/errorrates/histograms/images/hist_kset02_eGauss_ee+ise_npc_fisher_uncp.png]

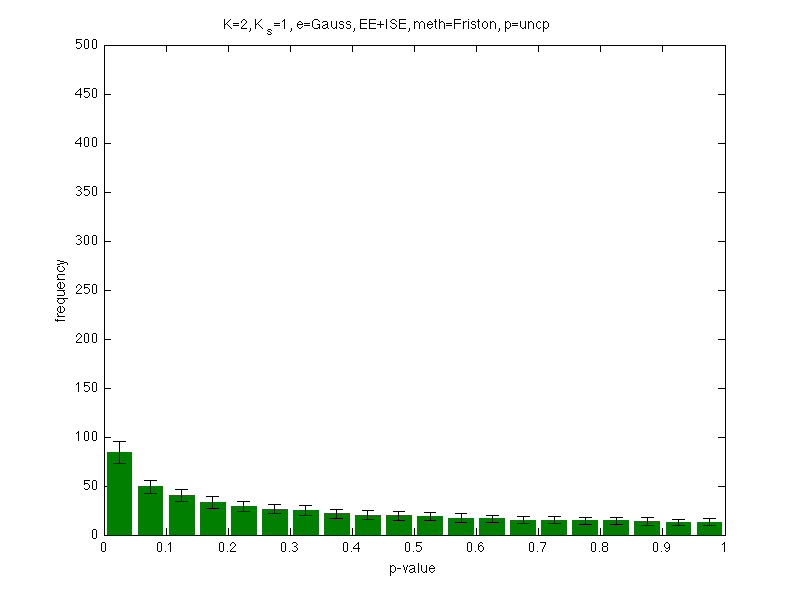

Supplement: Supplementary file 1 — Supporting Information [file HBM-37-1486-s001.zip › supporting_information/errorrates/histograms/images/hist_kset02_eGauss_ee+ise_npc_friston_uncp.png]

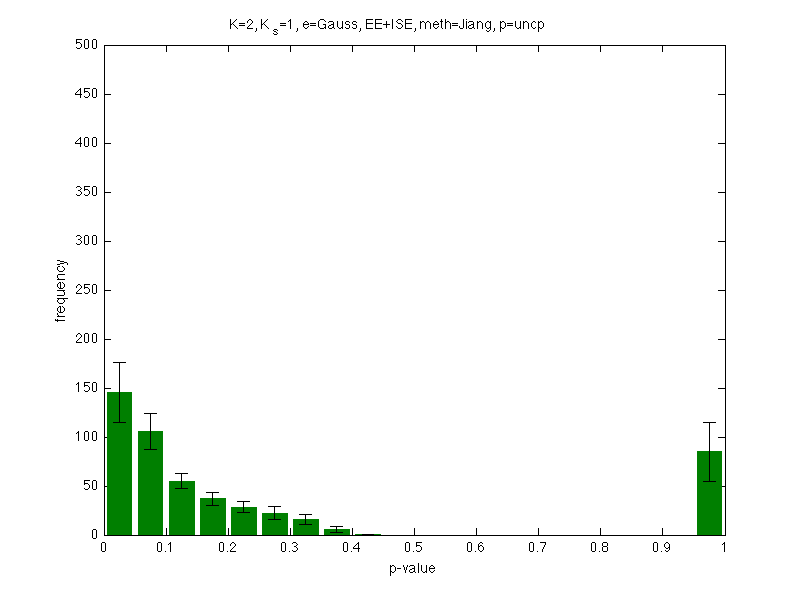

Supplement: Supplementary file 1 — Supporting Information [file HBM-37-1486-s001.zip › supporting_information/errorrates/histograms/images/hist_kset02_eGauss_ee+ise_npc_jiang_uncp.png]

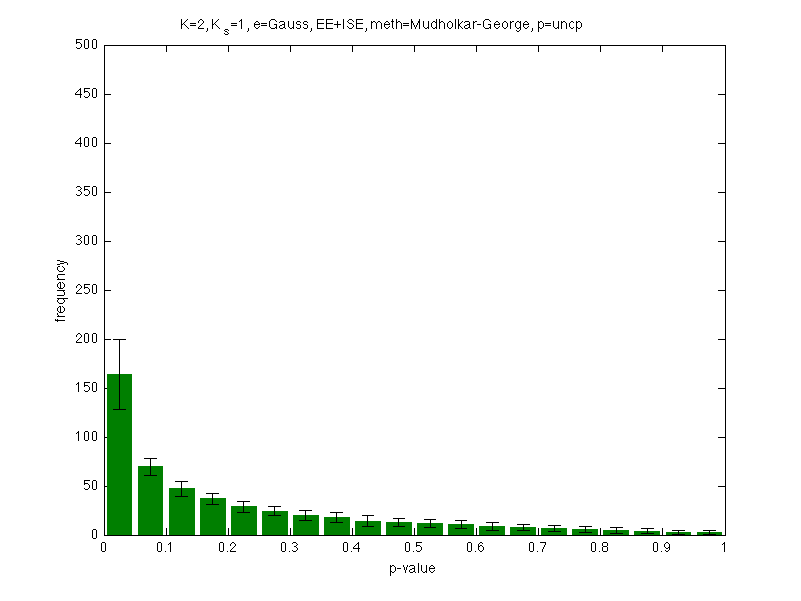

Supplement: Supplementary file 1 — Supporting Information [file HBM-37-1486-s001.zip › supporting_information/errorrates/histograms/images/hist_kset02_eGauss_ee+ise_npc_mudholkar-george_uncp.png]

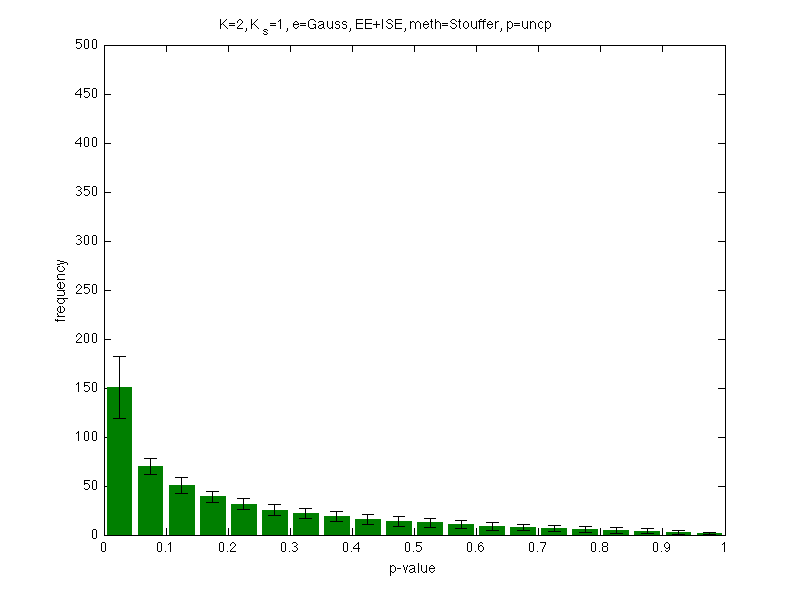

Supplement: Supplementary file 1 — Supporting Information [file HBM-37-1486-s001.zip › supporting_information/errorrates/histograms/images/hist_kset02_eGauss_ee+ise_npc_stouffer_uncp.png]

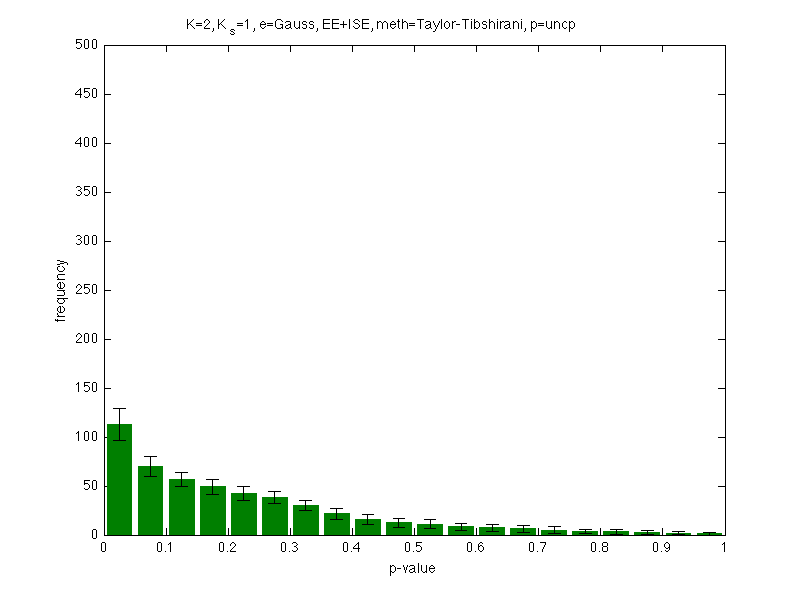

Supplement: Supplementary file 1 — Supporting Information [file HBM-37-1486-s001.zip › supporting_information/errorrates/histograms/images/hist_kset02_eGauss_ee+ise_npc_taylor-tibshirani_uncp.png]

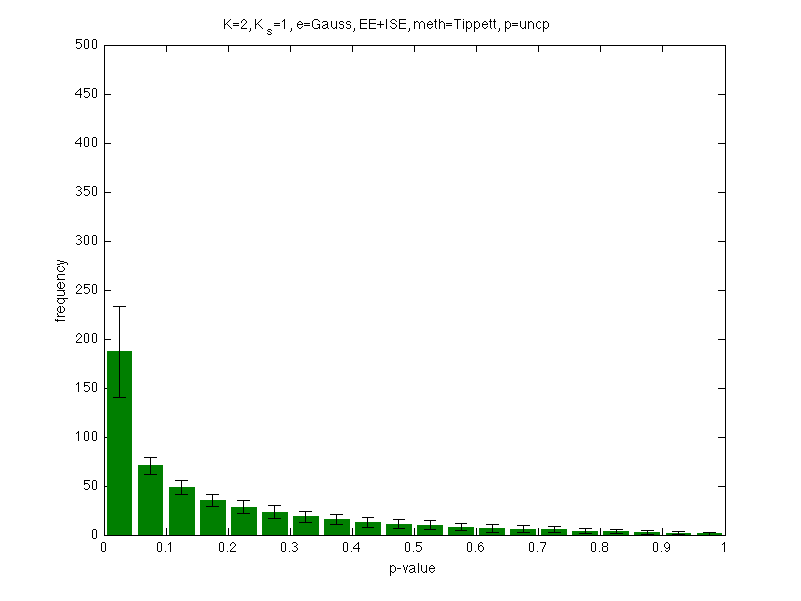

Supplement: Supplementary file 1 — Supporting Information [file HBM-37-1486-s001.zip › supporting_information/errorrates/histograms/images/hist_kset02_eGauss_ee+ise_npc_tippett_uncp.png]

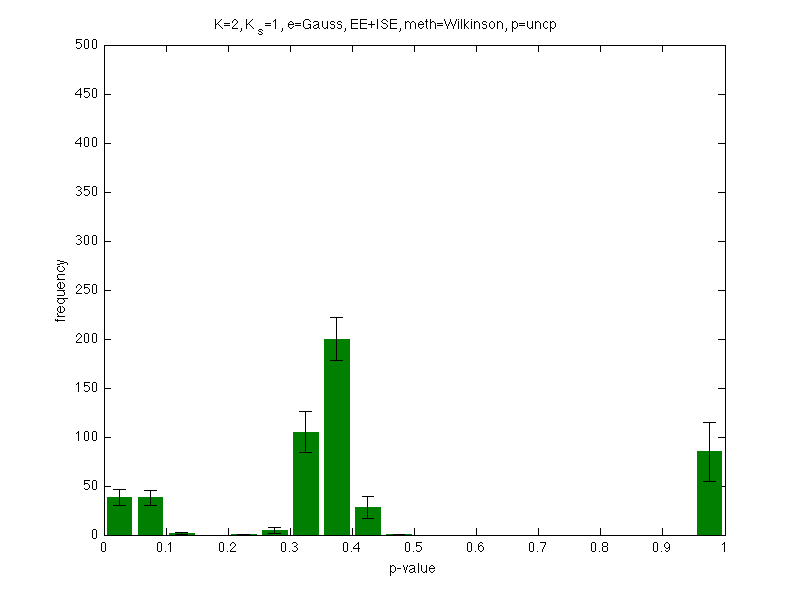

Supplement: Supplementary file 1 — Supporting Information [file HBM-37-1486-s001.zip › supporting_information/errorrates/histograms/images/hist_kset02_eGauss_ee+ise_npc_wilkinson_uncp.png]

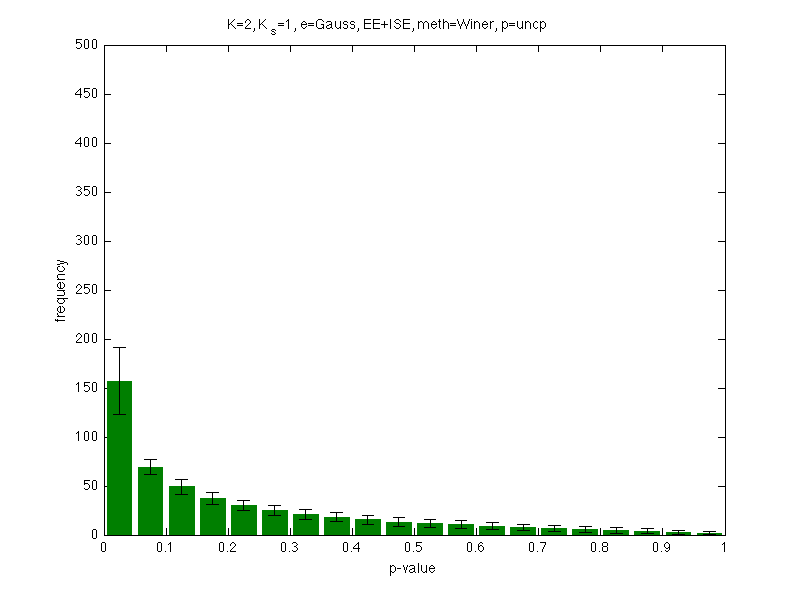

Supplement: Supplementary file 1 — Supporting Information [file HBM-37-1486-s001.zip › supporting_information/errorrates/histograms/images/hist_kset02_eGauss_ee+ise_npc_winer_uncp.png]

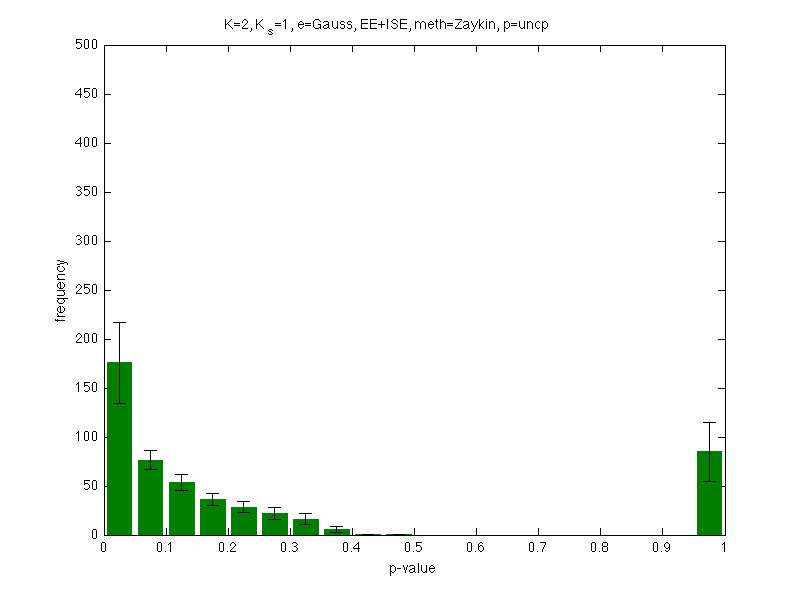

Supplement: Supplementary file 1 — Supporting Information [file HBM-37-1486-s001.zip › supporting_information/errorrates/histograms/images/hist_kset02_eGauss_ee+ise_npc_zaykin_uncp.png]

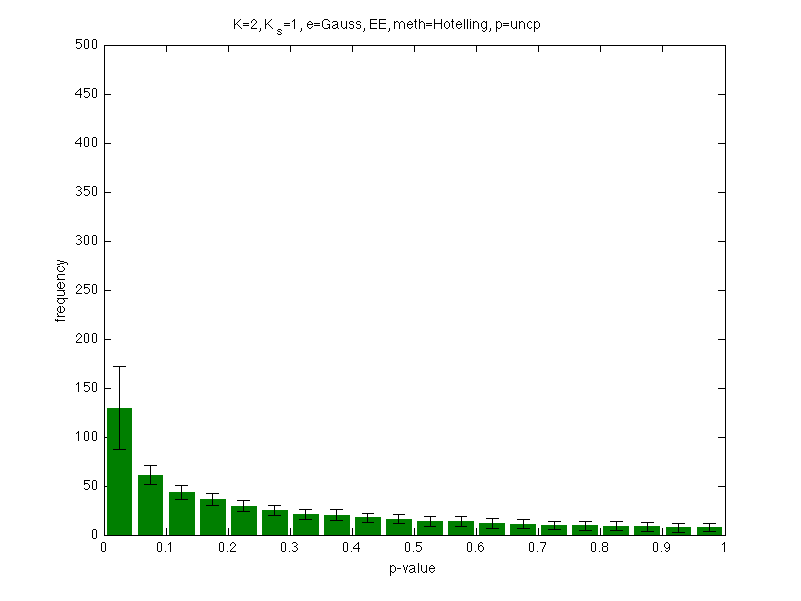

Supplement: Supplementary file 1 — Supporting Information [file HBM-37-1486-s001.zip › supporting_information/errorrates/histograms/images/hist_kset02_eGauss_ee_mv_hotellingtsq_uncp.png]

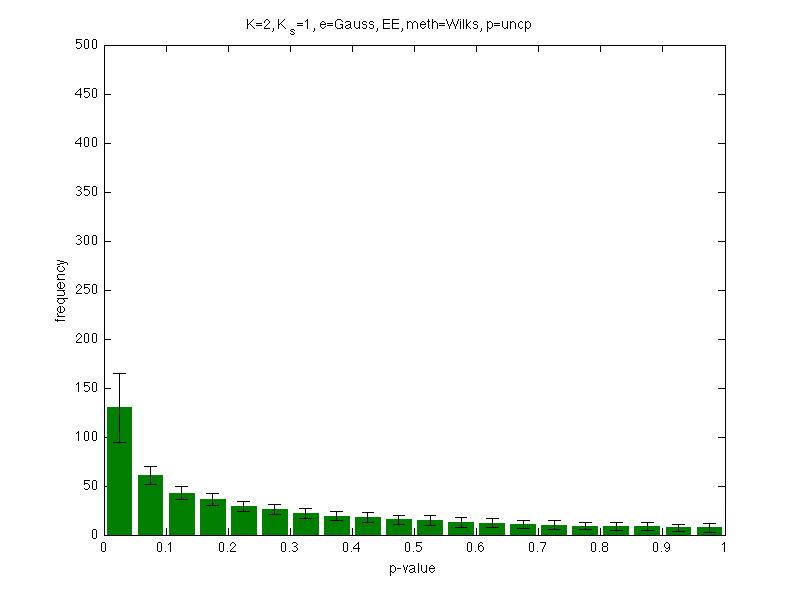

Supplement: Supplementary file 1 — Supporting Information [file HBM-37-1486-s001.zip › supporting_information/errorrates/histograms/images/hist_kset02_eGauss_ee_mv_wilks_uncp.png]

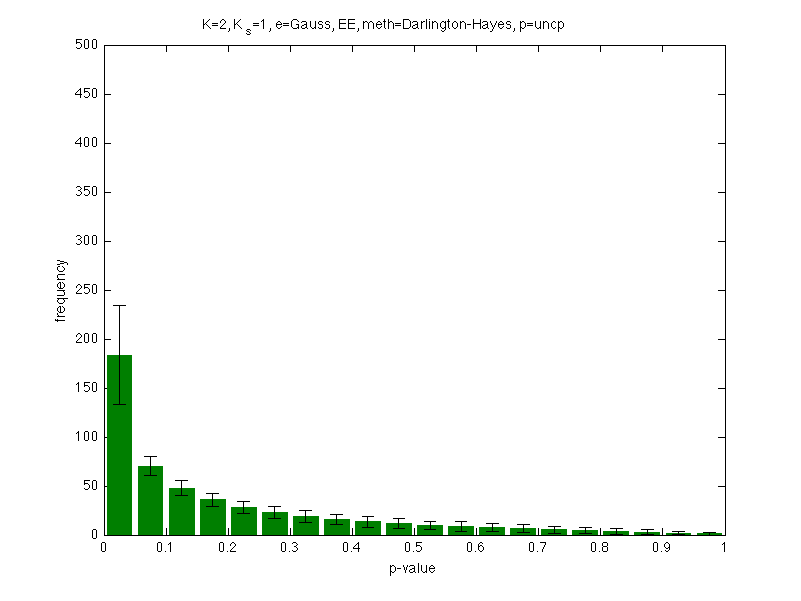

Supplement: Supplementary file 1 — Supporting Information [file HBM-37-1486-s001.zip › supporting_information/errorrates/histograms/images/hist_kset02_eGauss_ee_npc_darlington-hayes_uncp.png]

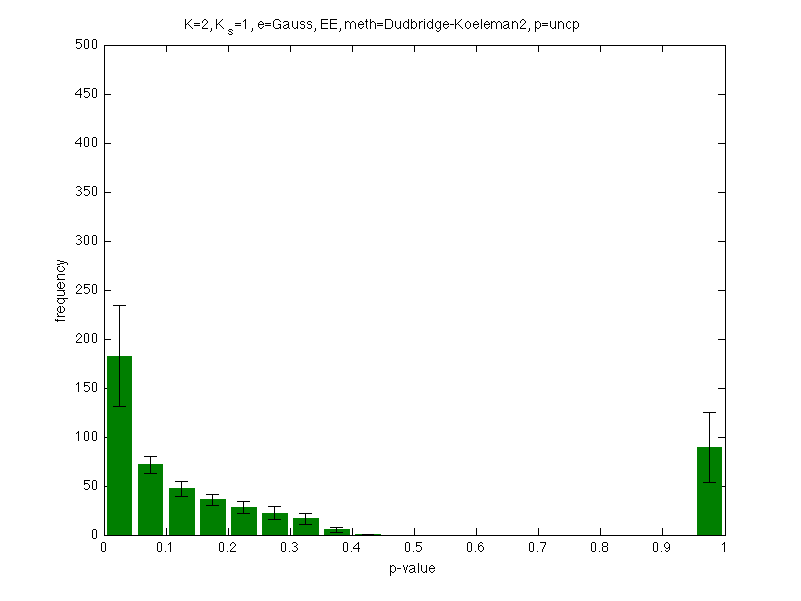

Supplement: Supplementary file 1 — Supporting Information [file HBM-37-1486-s001.zip › supporting_information/errorrates/histograms/images/hist_kset02_eGauss_ee_npc_dudbridge-koeleman2_uncp.png]

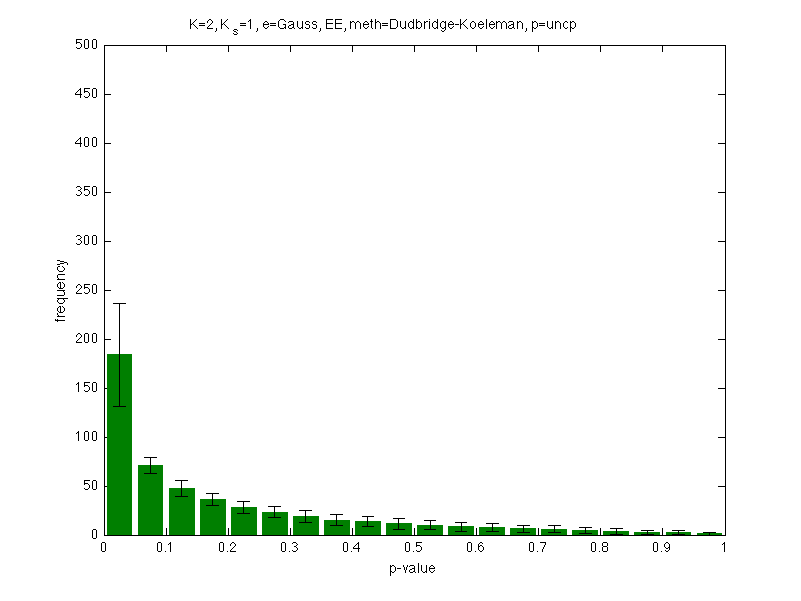

Supplement: Supplementary file 1 — Supporting Information [file HBM-37-1486-s001.zip › supporting_information/errorrates/histograms/images/hist_kset02_eGauss_ee_npc_dudbridge-koeleman_uncp.png]

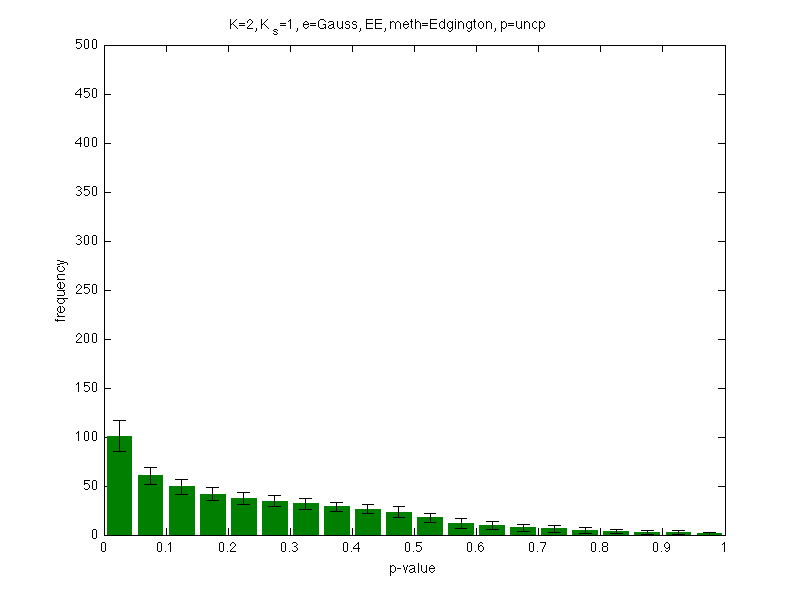

Supplement: Supplementary file 1 — Supporting Information [file HBM-37-1486-s001.zip › supporting_information/errorrates/histograms/images/hist_kset02_eGauss_ee_npc_edgington_uncp.png]

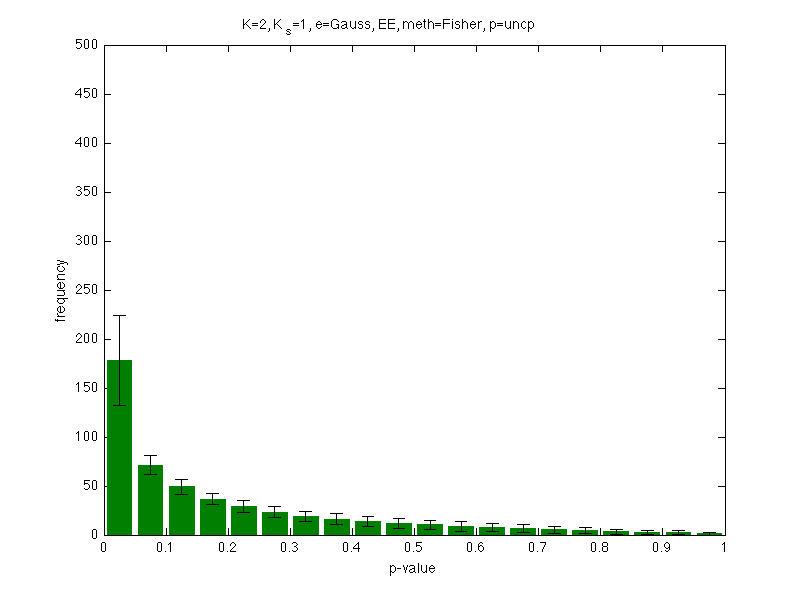

Supplement: Supplementary file 1 — Supporting Information [file HBM-37-1486-s001.zip › supporting_information/errorrates/histograms/images/hist_kset02_eGauss_ee_npc_fisher_uncp.png]

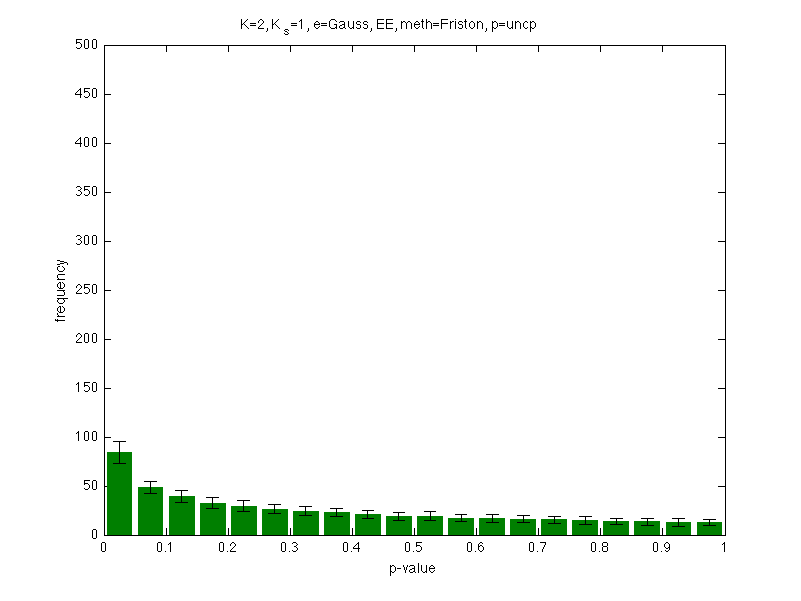

Supplement: Supplementary file 1 — Supporting Information [file HBM-37-1486-s001.zip › supporting_information/errorrates/histograms/images/hist_kset02_eGauss_ee_npc_friston_uncp.png]

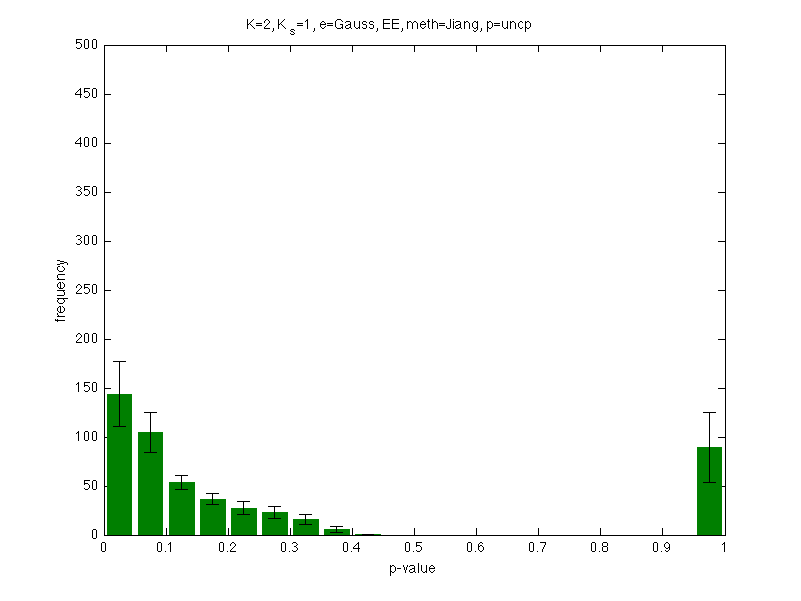

Supplement: Supplementary file 1 — Supporting Information [file HBM-37-1486-s001.zip › supporting_information/errorrates/histograms/images/hist_kset02_eGauss_ee_npc_jiang_uncp.png]

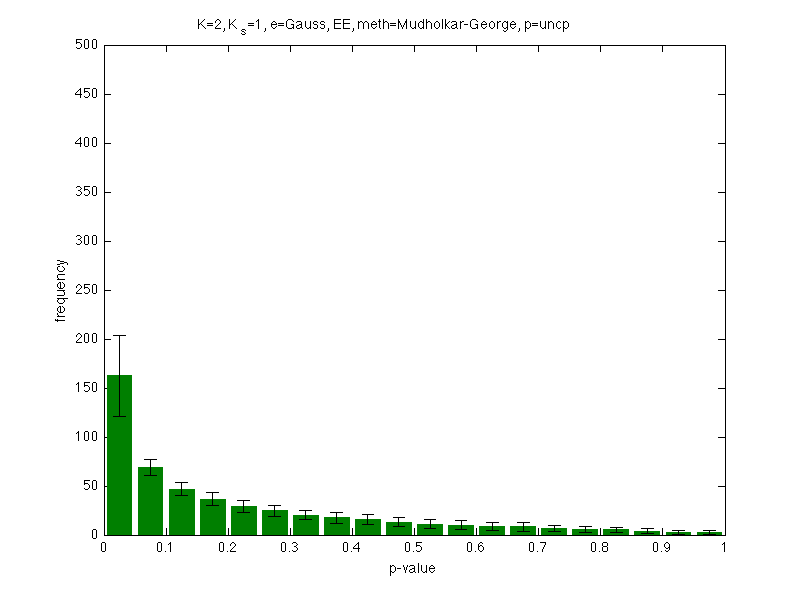

Supplement: Supplementary file 1 — Supporting Information [file HBM-37-1486-s001.zip › supporting_information/errorrates/histograms/images/hist_kset02_eGauss_ee_npc_mudholkar-george_uncp.png]

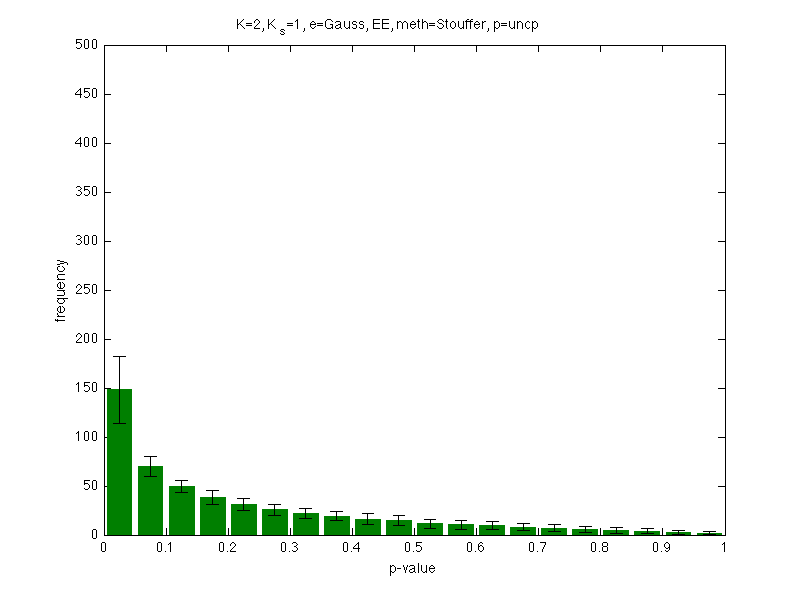

Supplement: Supplementary file 1 — Supporting Information [file HBM-37-1486-s001.zip › supporting_information/errorrates/histograms/images/hist_kset02_eGauss_ee_npc_stouffer_uncp.png]

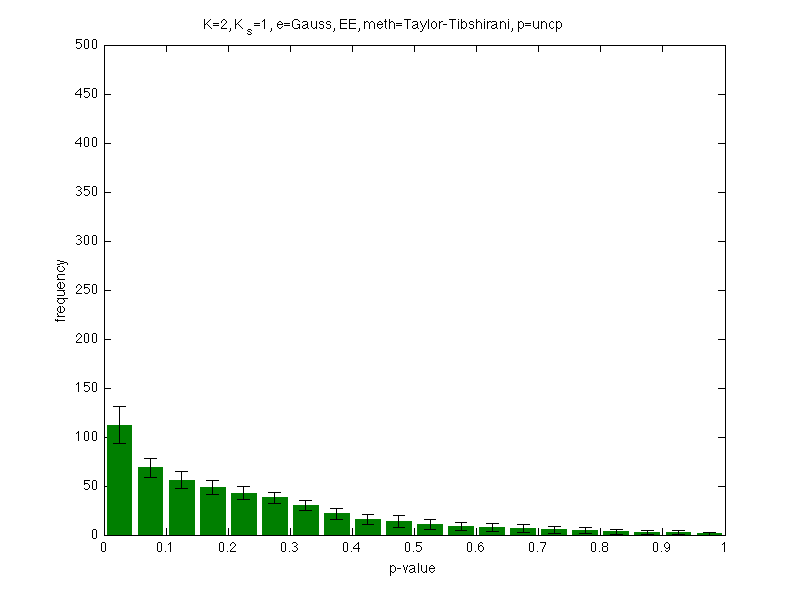

Supplement: Supplementary file 1 — Supporting Information [file HBM-37-1486-s001.zip › supporting_information/errorrates/histograms/images/hist_kset02_eGauss_ee_npc_taylor-tibshirani_uncp.png]

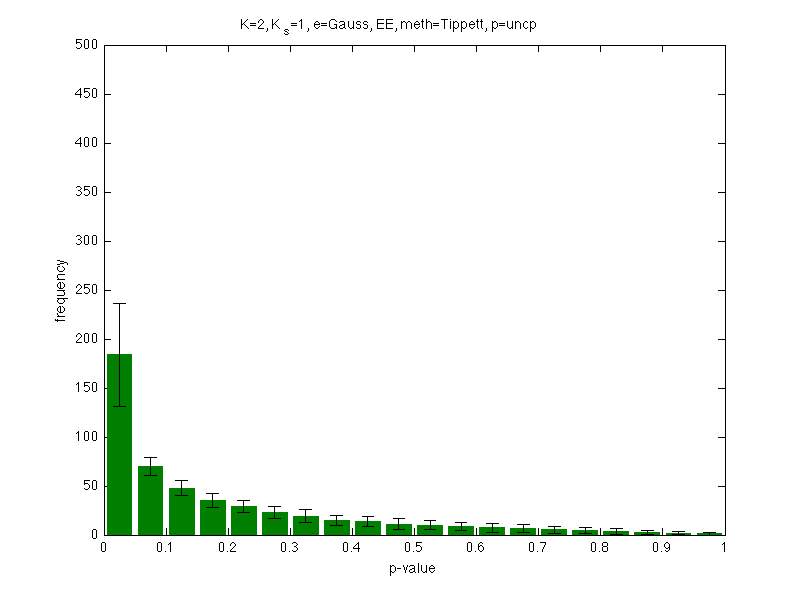

Supplement: Supplementary file 1 — Supporting Information [file HBM-37-1486-s001.zip › supporting_information/errorrates/histograms/images/hist_kset02_eGauss_ee_npc_tippett_uncp.png]

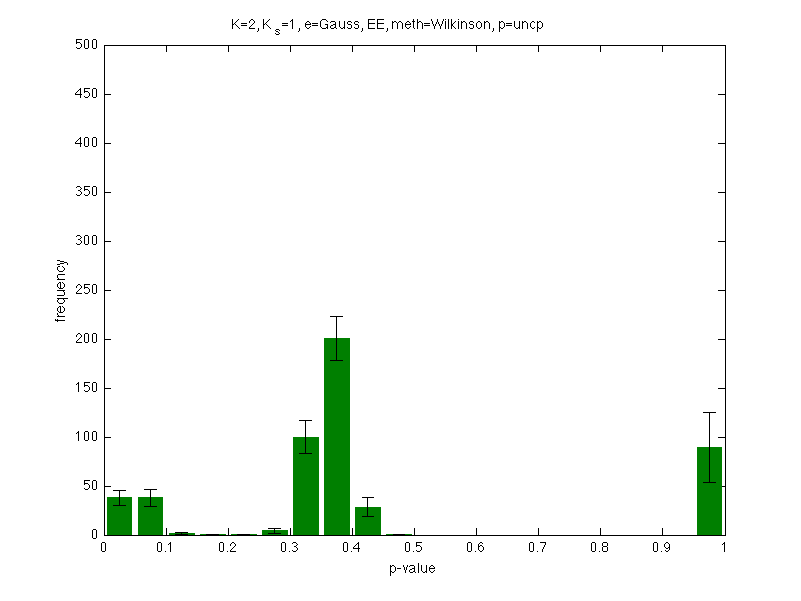

Supplement: Supplementary file 1 — Supporting Information [file HBM-37-1486-s001.zip › supporting_information/errorrates/histograms/images/hist_kset02_eGauss_ee_npc_wilkinson_uncp.png]

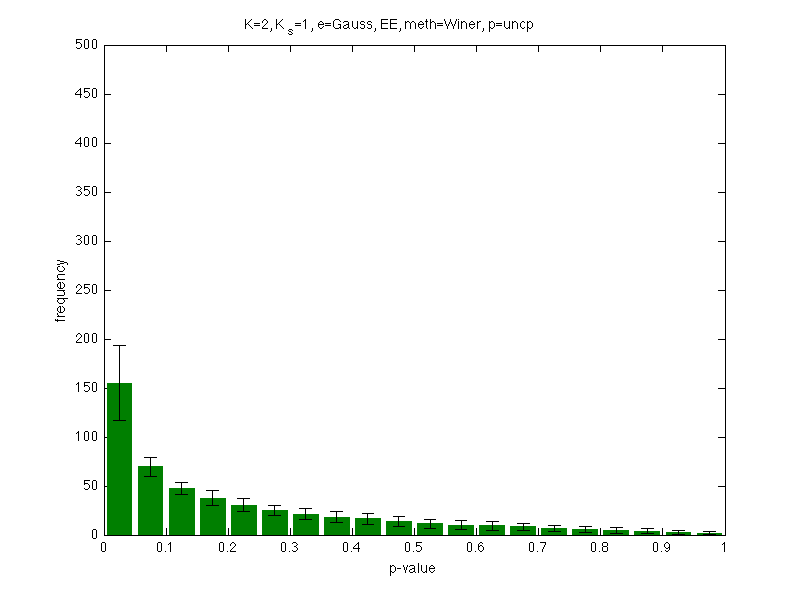

Supplement: Supplementary file 1 — Supporting Information [file HBM-37-1486-s001.zip › supporting_information/errorrates/histograms/images/hist_kset02_eGauss_ee_npc_winer_uncp.png]

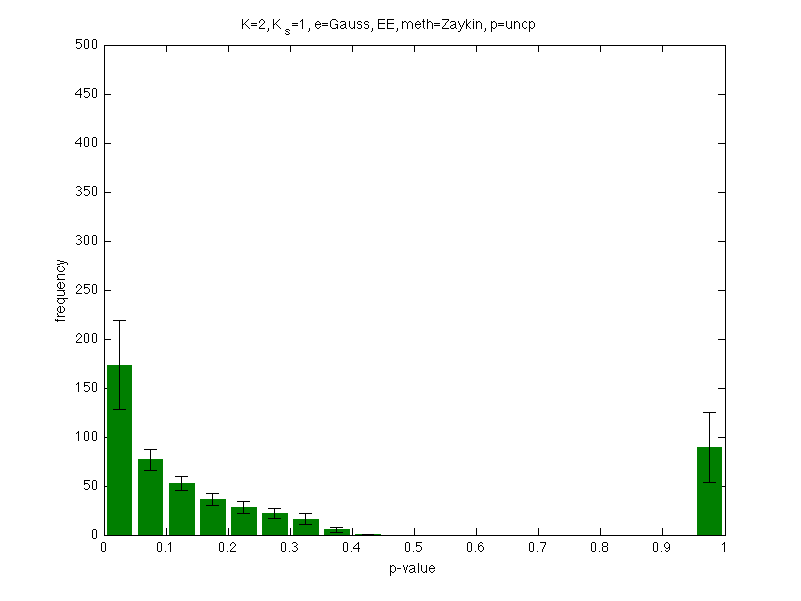

Supplement: Supplementary file 1 — Supporting Information [file HBM-37-1486-s001.zip › supporting_information/errorrates/histograms/images/hist_kset02_eGauss_ee_npc_zaykin_uncp.png]

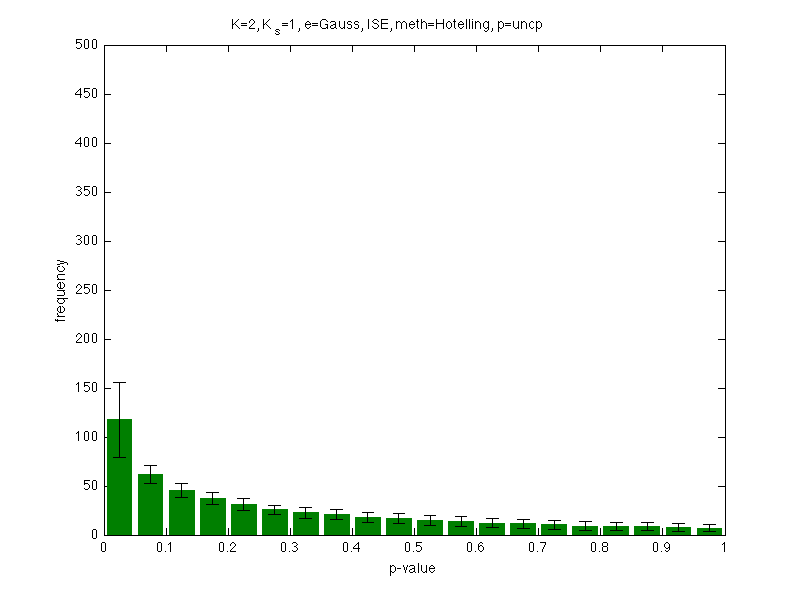

Supplement: Supplementary file 1 — Supporting Information [file HBM-37-1486-s001.zip › supporting_information/errorrates/histograms/images/hist_kset02_eGauss_ise_mv_hotellingtsq_uncp.png]

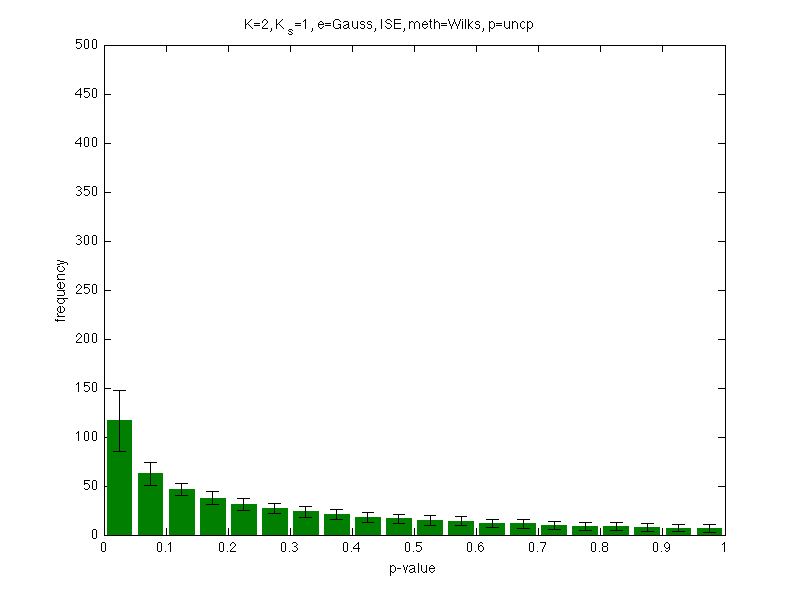

Supplement: Supplementary file 1 — Supporting Information [file HBM-37-1486-s001.zip › supporting_information/errorrates/histograms/images/hist_kset02_eGauss_ise_mv_wilks_uncp.png]

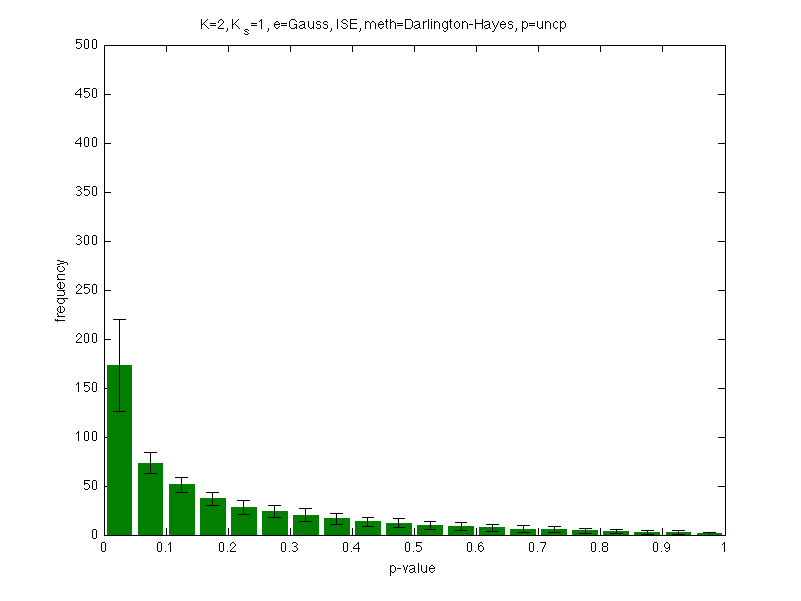

Supplement: Supplementary file 1 — Supporting Information [file HBM-37-1486-s001.zip › supporting_information/errorrates/histograms/images/hist_kset02_eGauss_ise_npc_darlington-hayes_uncp.png]

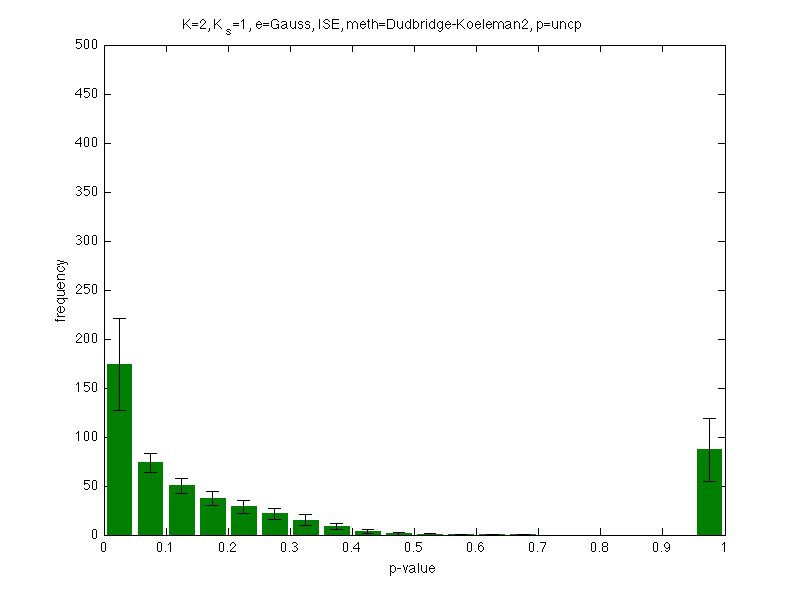

Supplement: Supplementary file 1 — Supporting Information [file HBM-37-1486-s001.zip › supporting_information/errorrates/histograms/images/hist_kset02_eGauss_ise_npc_dudbridge-koeleman2_uncp.png]

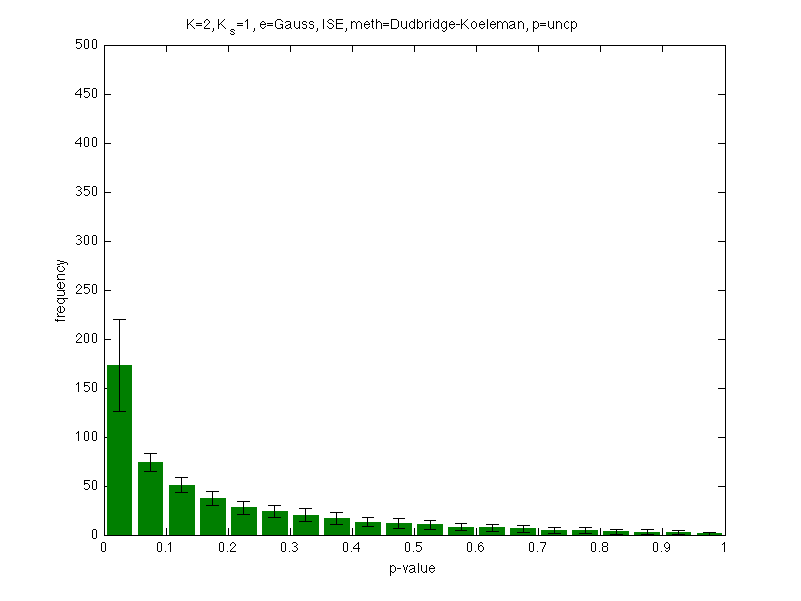

Supplement: Supplementary file 1 — Supporting Information [file HBM-37-1486-s001.zip › supporting_information/errorrates/histograms/images/hist_kset02_eGauss_ise_npc_dudbridge-koeleman_uncp.png]

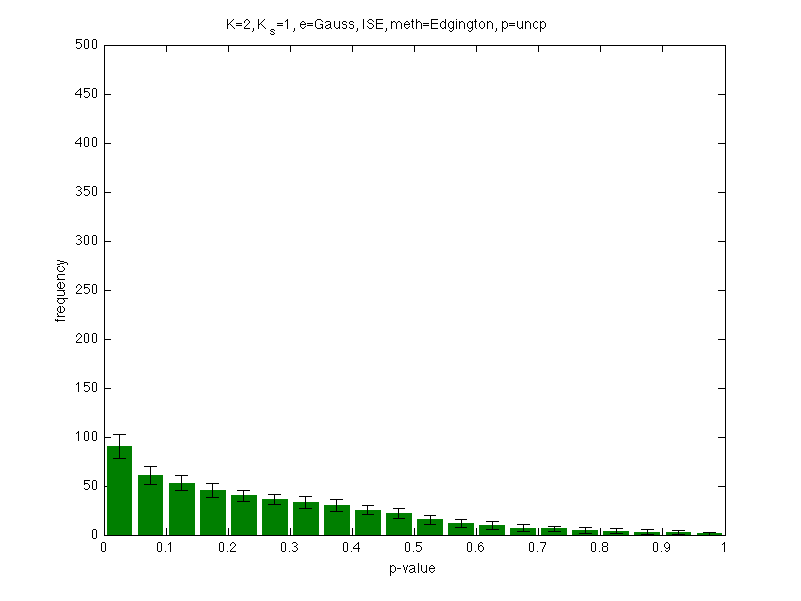

Supplement: Supplementary file 1 — Supporting Information [file HBM-37-1486-s001.zip › supporting_information/errorrates/histograms/images/hist_kset02_eGauss_ise_npc_edgington_uncp.png]

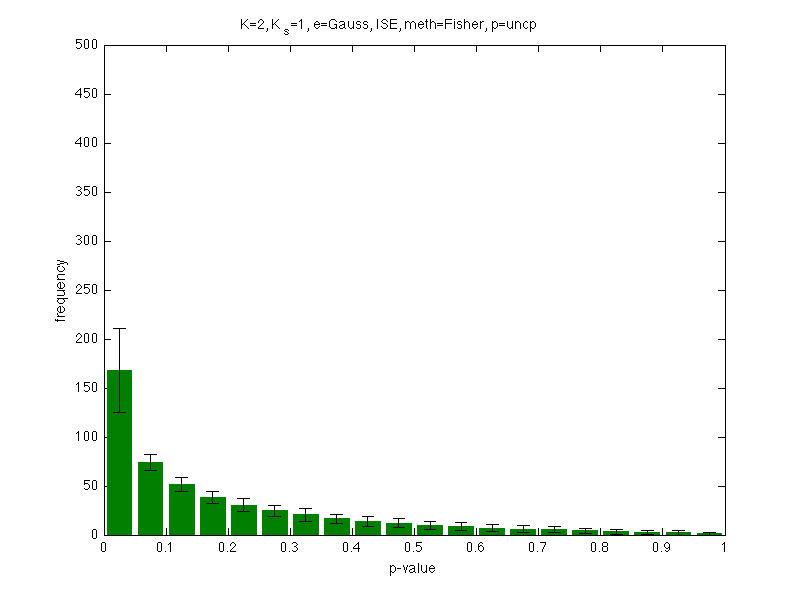

Supplement: Supplementary file 1 — Supporting Information [file HBM-37-1486-s001.zip › supporting_information/errorrates/histograms/images/hist_kset02_eGauss_ise_npc_fisher_uncp.png]

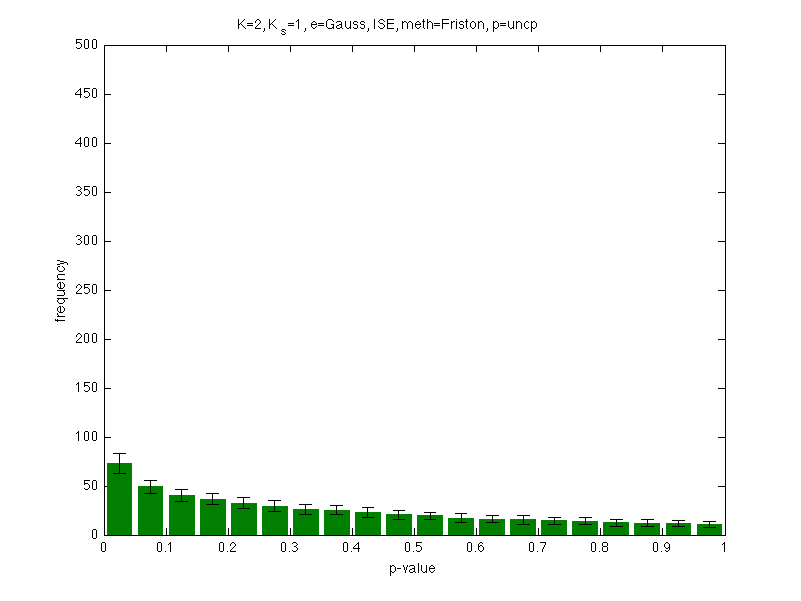

Supplement: Supplementary file 1 — Supporting Information [file HBM-37-1486-s001.zip › supporting_information/errorrates/histograms/images/hist_kset02_eGauss_ise_npc_friston_uncp.png]

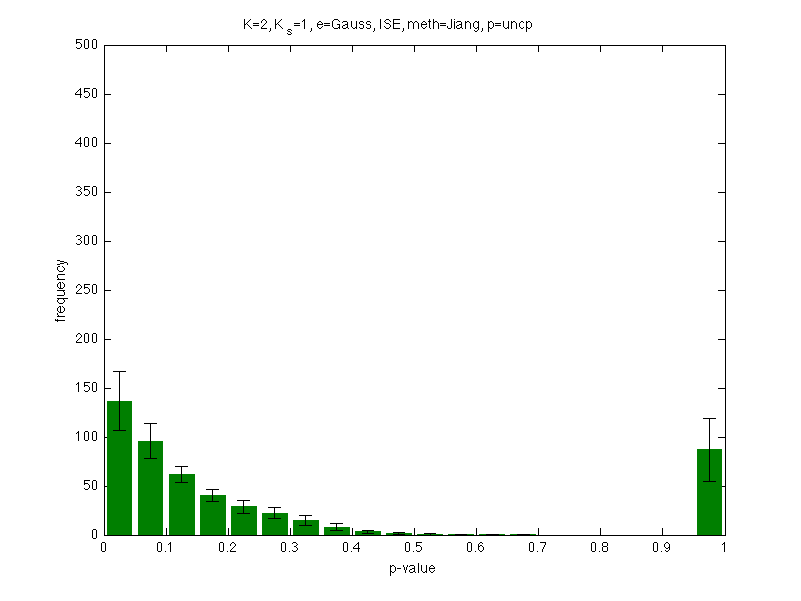

Supplement: Supplementary file 1 — Supporting Information [file HBM-37-1486-s001.zip › supporting_information/errorrates/histograms/images/hist_kset02_eGauss_ise_npc_jiang_uncp.png]

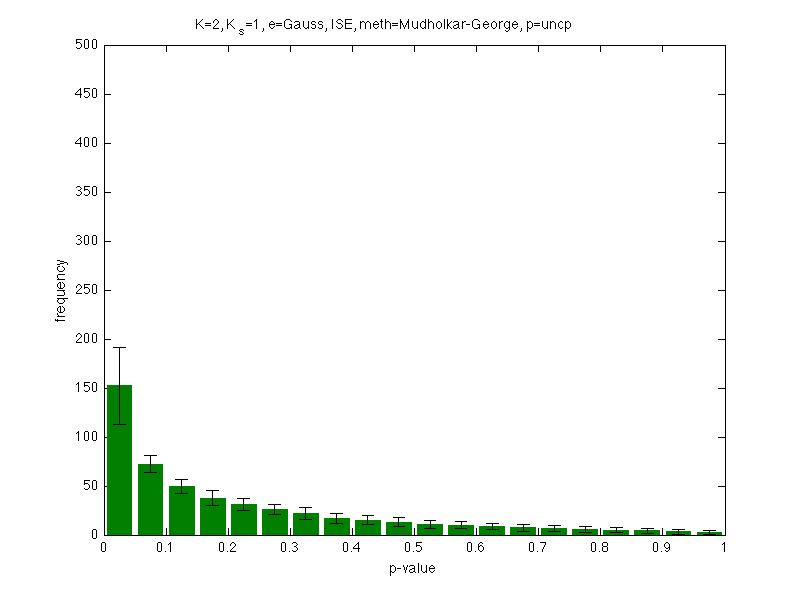

Supplement: Supplementary file 1 — Supporting Information [file HBM-37-1486-s001.zip › supporting_information/errorrates/histograms/images/hist_kset02_eGauss_ise_npc_mudholkar-george_uncp.png]

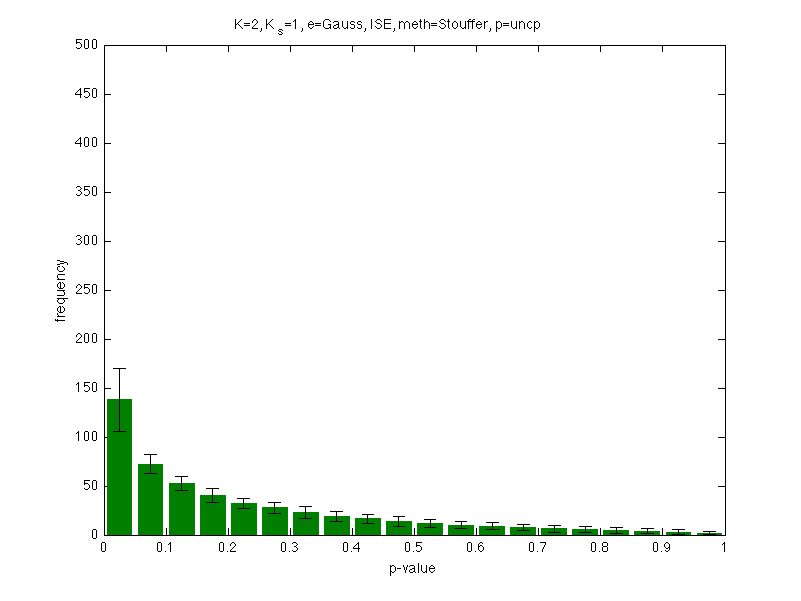

Supplement: Supplementary file 1 — Supporting Information [file HBM-37-1486-s001.zip › supporting_information/errorrates/histograms/images/hist_kset02_eGauss_ise_npc_stouffer_uncp.png]

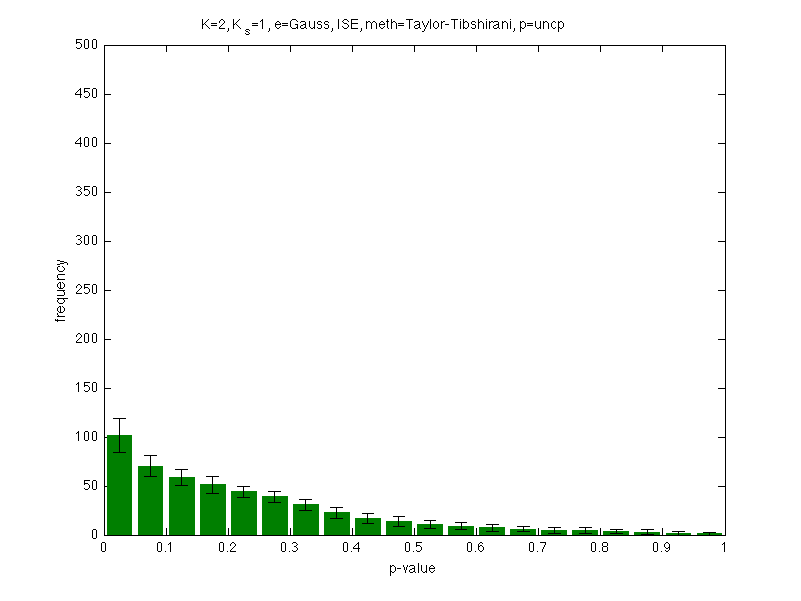

Supplement: Supplementary file 1 — Supporting Information [file HBM-37-1486-s001.zip › supporting_information/errorrates/histograms/images/hist_kset02_eGauss_ise_npc_taylor-tibshirani_uncp.png]

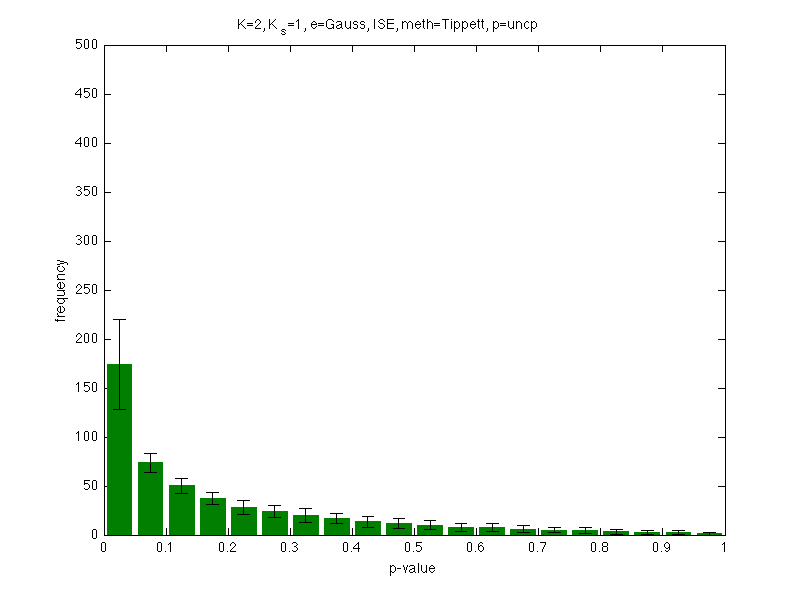

Supplement: Supplementary file 1 — Supporting Information [file HBM-37-1486-s001.zip › supporting_information/errorrates/histograms/images/hist_kset02_eGauss_ise_npc_tippett_uncp.png]

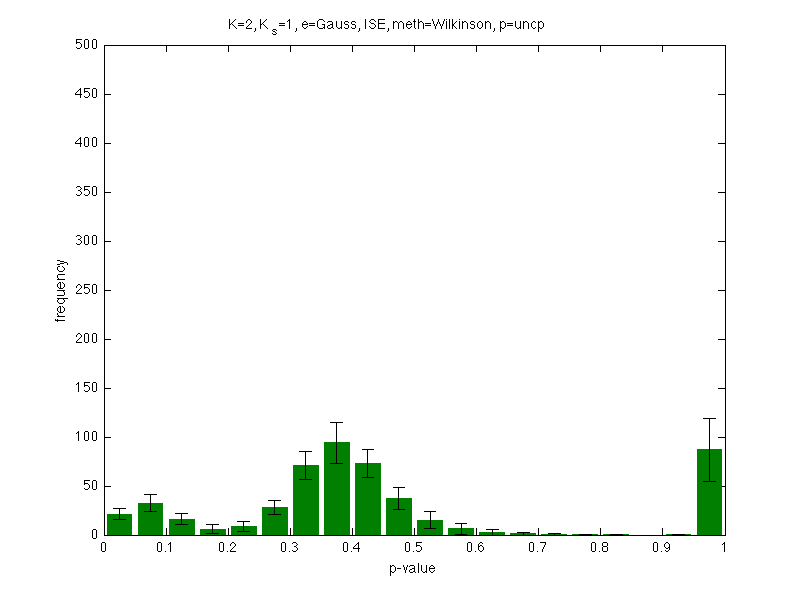

Supplement: Supplementary file 1 — Supporting Information [file HBM-37-1486-s001.zip › supporting_information/errorrates/histograms/images/hist_kset02_eGauss_ise_npc_wilkinson_uncp.png]

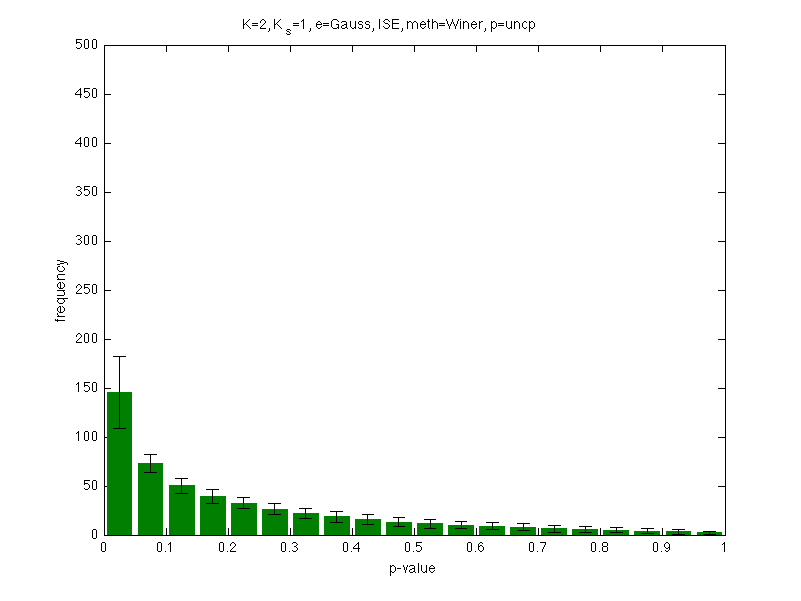

Supplement: Supplementary file 1 — Supporting Information [file HBM-37-1486-s001.zip › supporting_information/errorrates/histograms/images/hist_kset02_eGauss_ise_npc_winer_uncp.png]

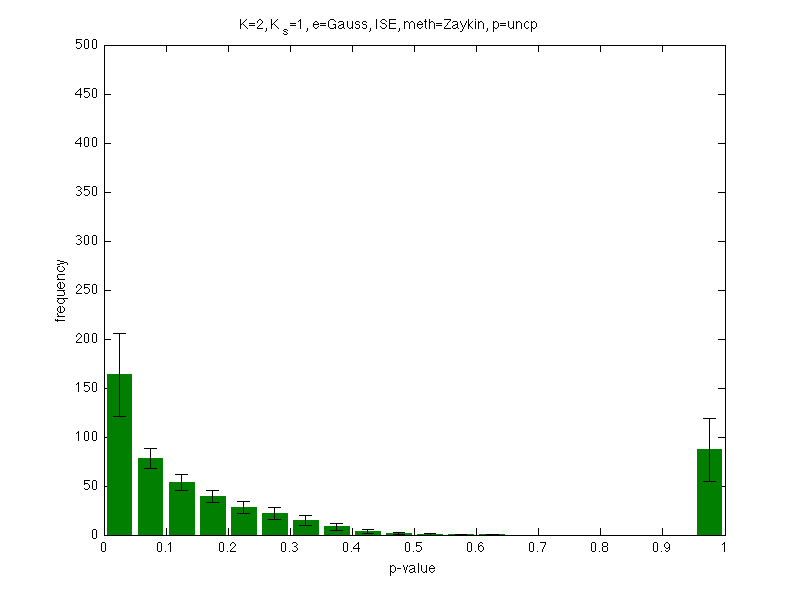

Supplement: Supplementary file 1 — Supporting Information [file HBM-37-1486-s001.zip › supporting_information/errorrates/histograms/images/hist_kset02_eGauss_ise_npc_zaykin_uncp.png]

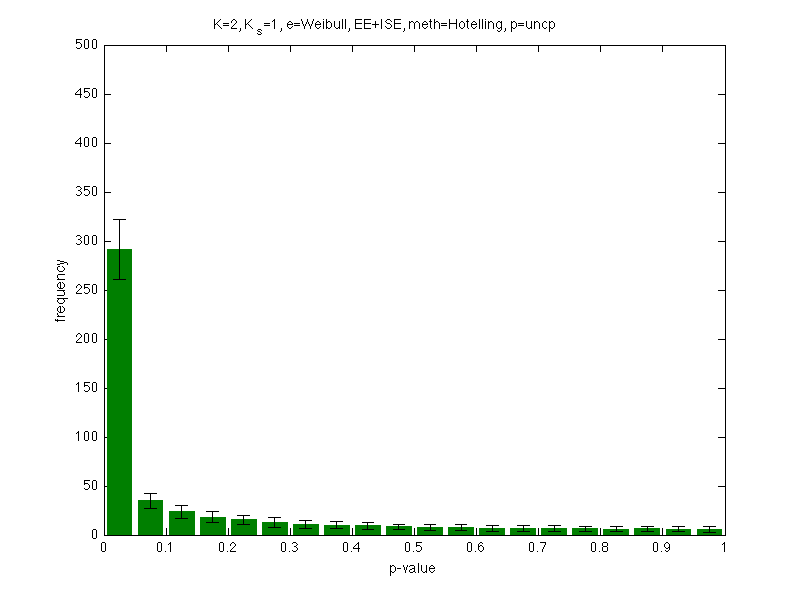

Supplement: Supplementary file 1 — Supporting Information [file HBM-37-1486-s001.zip › supporting_information/errorrates/histograms/images/hist_kset02_eWeibull_ee+ise_mv_hotellingtsq_uncp.png]

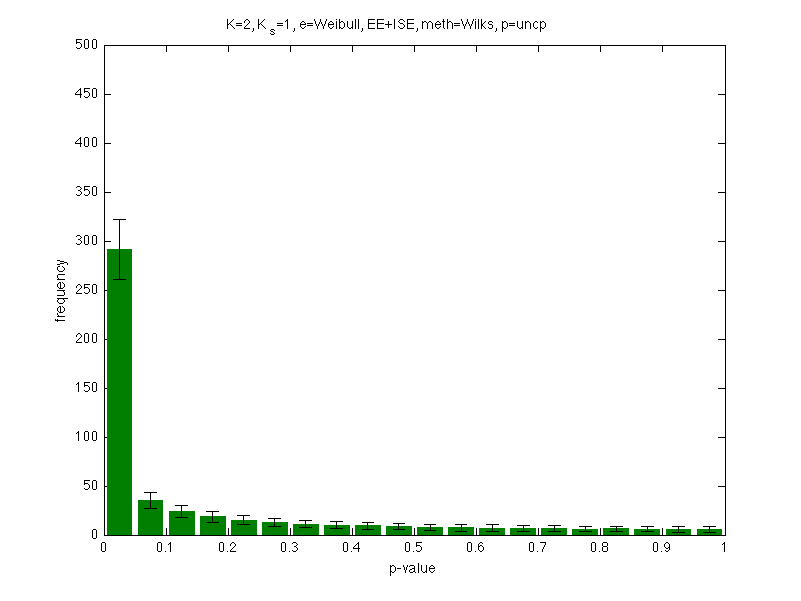

Supplement: Supplementary file 1 — Supporting Information [file HBM-37-1486-s001.zip › supporting_information/errorrates/histograms/images/hist_kset02_eWeibull_ee+ise_mv_wilks_uncp.png]

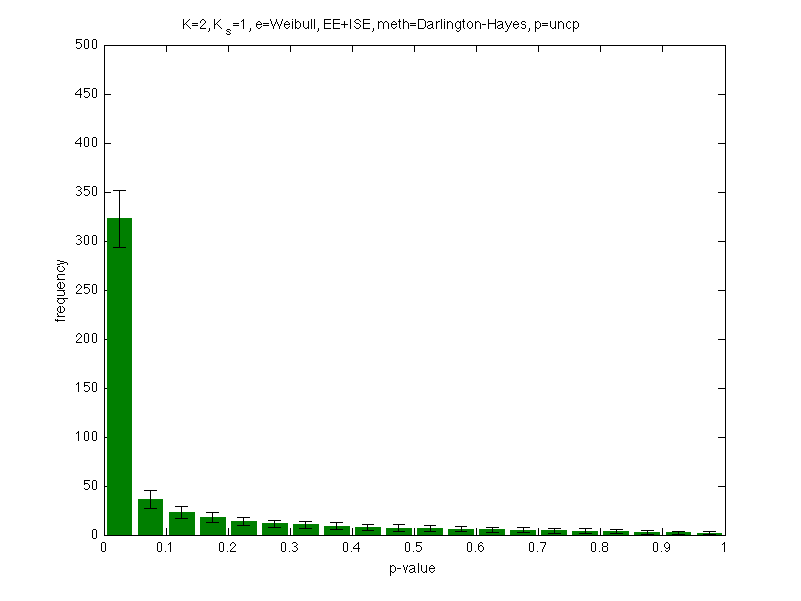

Supplement: Supplementary file 1 — Supporting Information [file HBM-37-1486-s001.zip › supporting_information/errorrates/histograms/images/hist_kset02_eWeibull_ee+ise_npc_darlington-hayes_uncp.png]

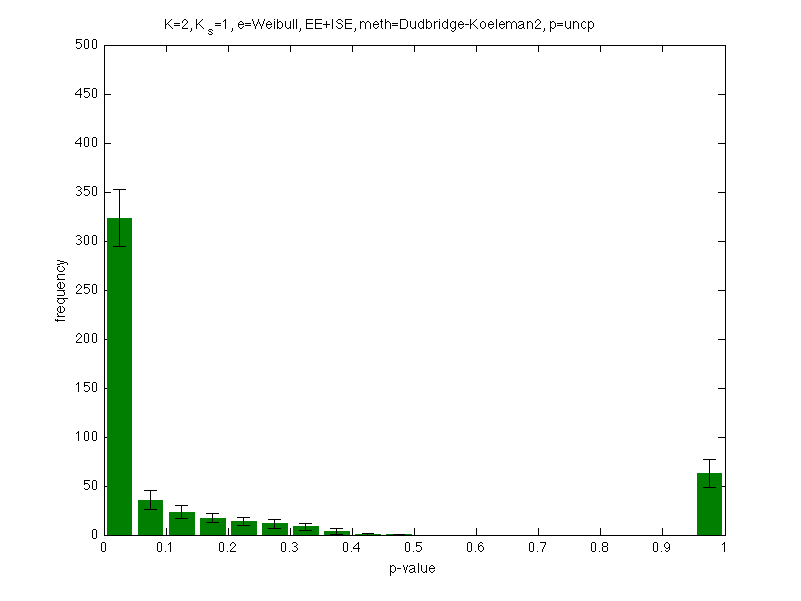

Supplement: Supplementary file 1 — Supporting Information [file HBM-37-1486-s001.zip › supporting_information/errorrates/histograms/images/hist_kset02_eWeibull_ee+ise_npc_dudbridge-koeleman2_uncp.png]

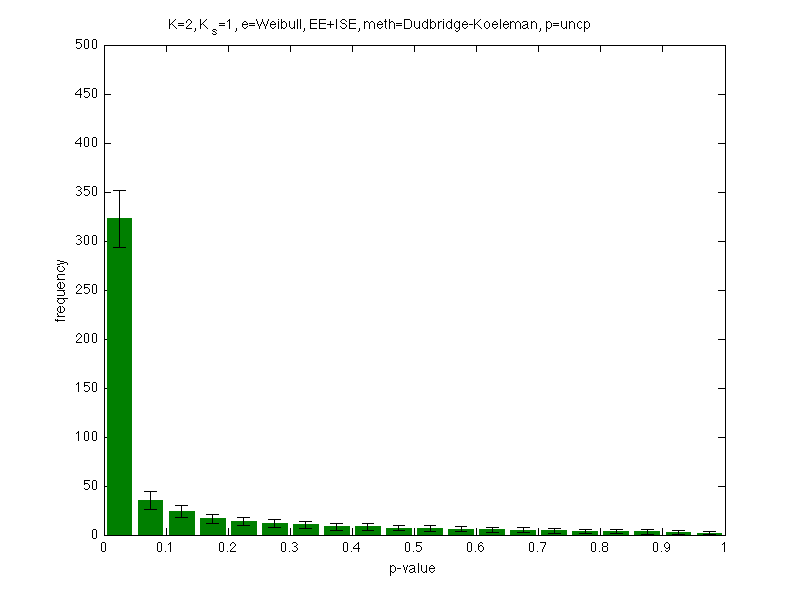

Supplement: Supplementary file 1 — Supporting Information [file HBM-37-1486-s001.zip › supporting_information/errorrates/histograms/images/hist_kset02_eWeibull_ee+ise_npc_dudbridge-koeleman_uncp.png]

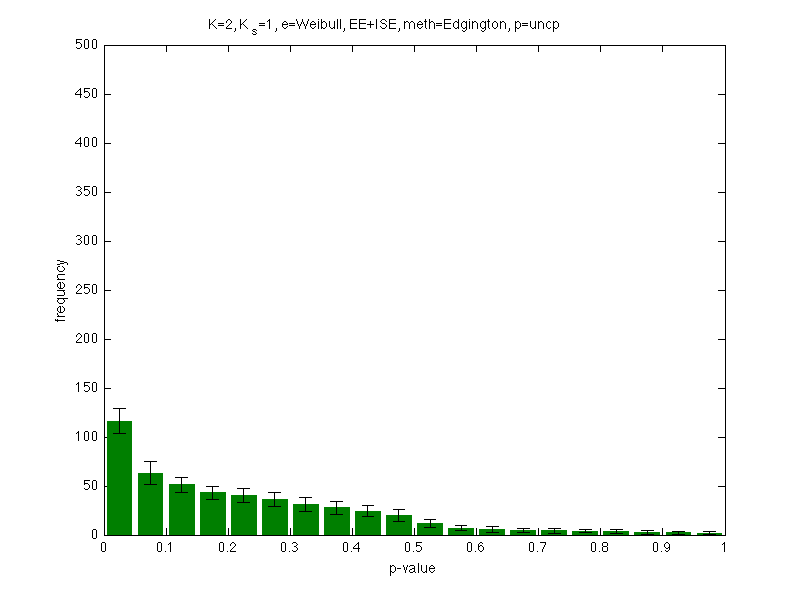

Supplement: Supplementary file 1 — Supporting Information [file HBM-37-1486-s001.zip › supporting_information/errorrates/histograms/images/hist_kset02_eWeibull_ee+ise_npc_edgington_uncp.png]

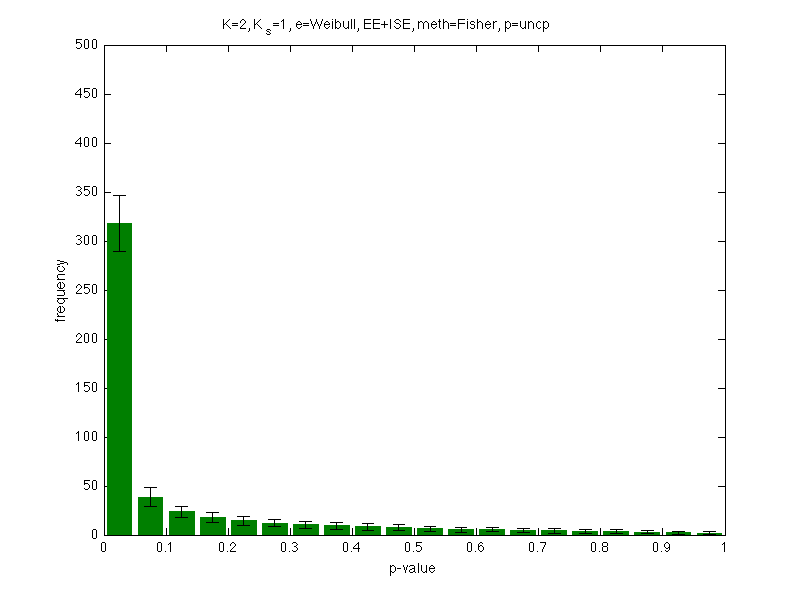

Supplement: Supplementary file 1 — Supporting Information [file HBM-37-1486-s001.zip › supporting_information/errorrates/histograms/images/hist_kset02_eWeibull_ee+ise_npc_fisher_uncp.png]

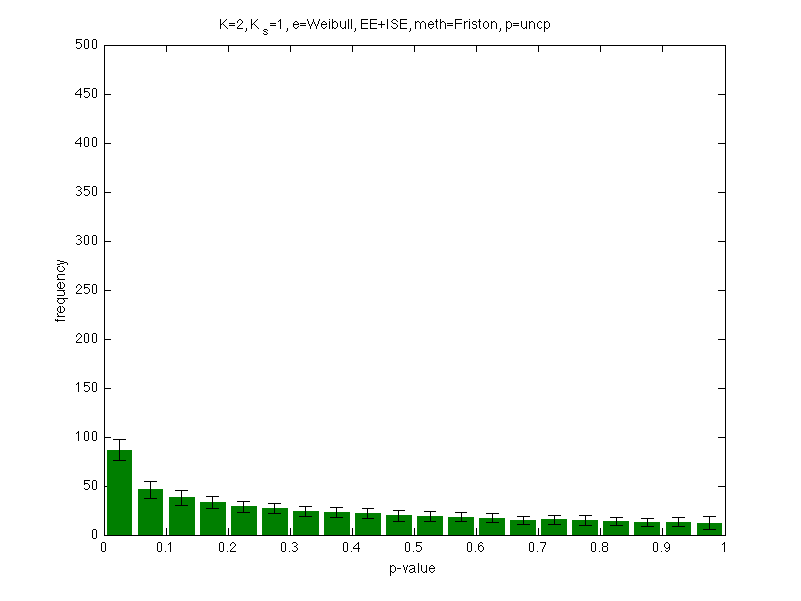

Supplement: Supplementary file 1 — Supporting Information [file HBM-37-1486-s001.zip › supporting_information/errorrates/histograms/images/hist_kset02_eWeibull_ee+ise_npc_friston_uncp.png]

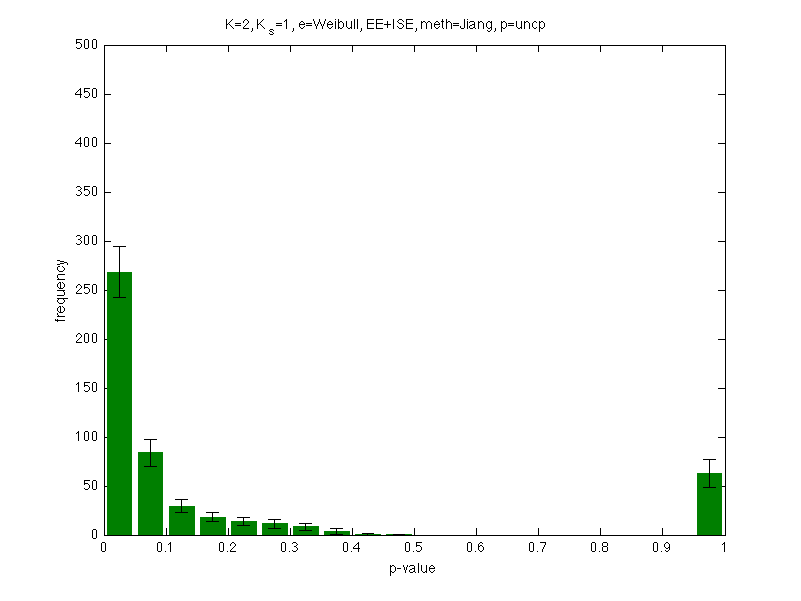

Supplement: Supplementary file 1 — Supporting Information [file HBM-37-1486-s001.zip › supporting_information/errorrates/histograms/images/hist_kset02_eWeibull_ee+ise_npc_jiang_uncp.png]

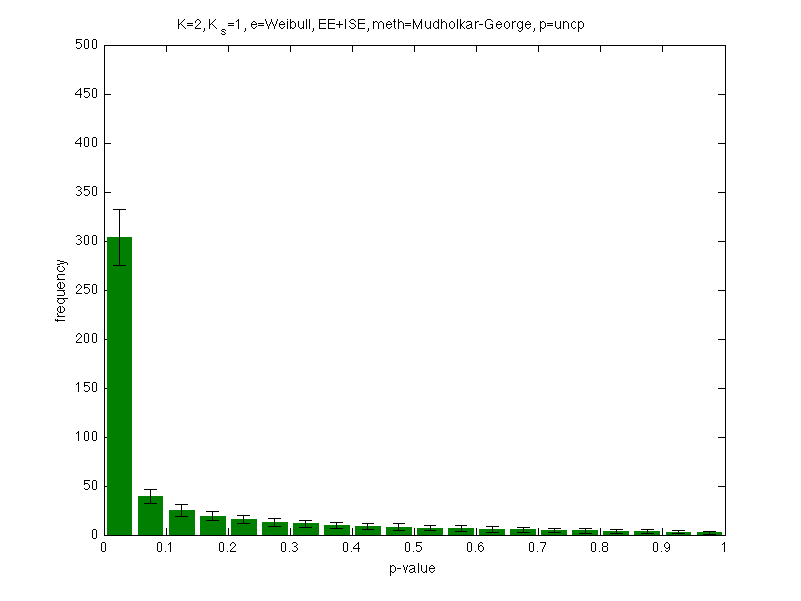

Supplement: Supplementary file 1 — Supporting Information [file HBM-37-1486-s001.zip › supporting_information/errorrates/histograms/images/hist_kset02_eWeibull_ee+ise_npc_mudholkar-george_uncp.png]

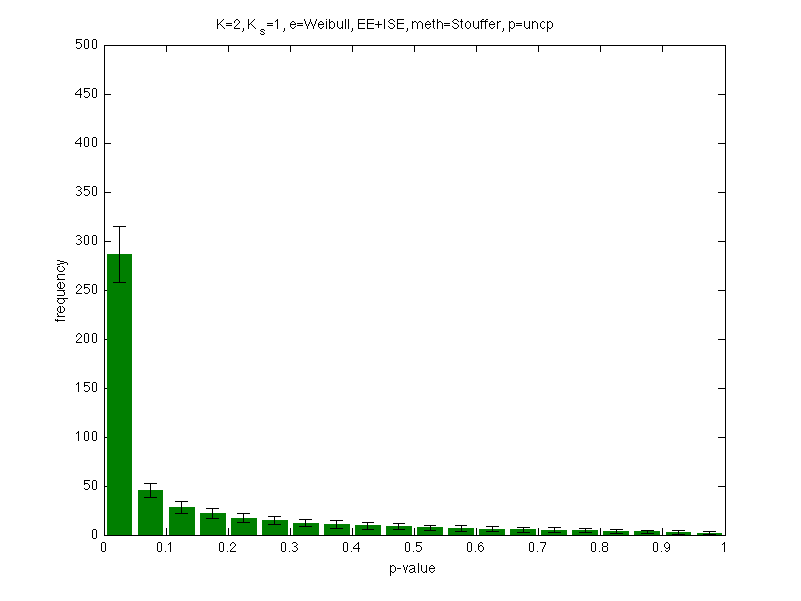

Supplement: Supplementary file 1 — Supporting Information [file HBM-37-1486-s001.zip › supporting_information/errorrates/histograms/images/hist_kset02_eWeibull_ee+ise_npc_stouffer_uncp.png]

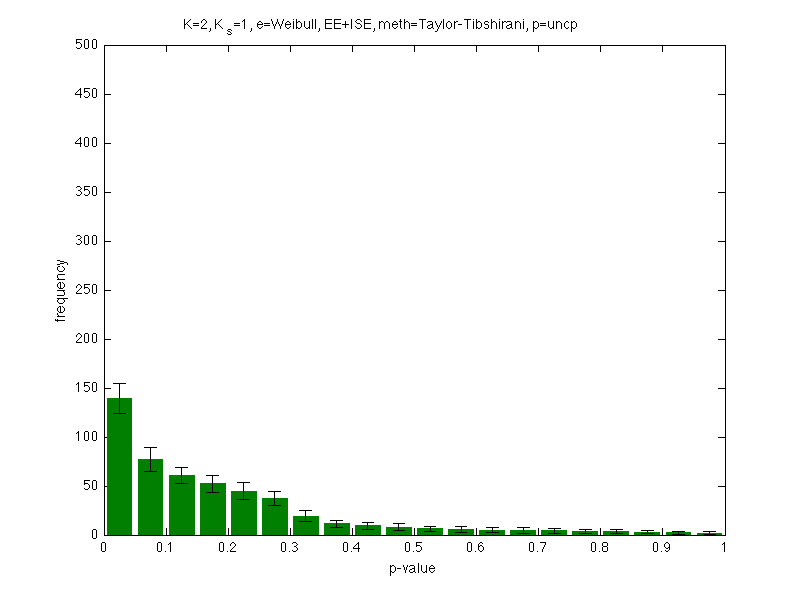

Supplement: Supplementary file 1 — Supporting Information [file HBM-37-1486-s001.zip › supporting_information/errorrates/histograms/images/hist_kset02_eWeibull_ee+ise_npc_taylor-tibshirani_uncp.png]

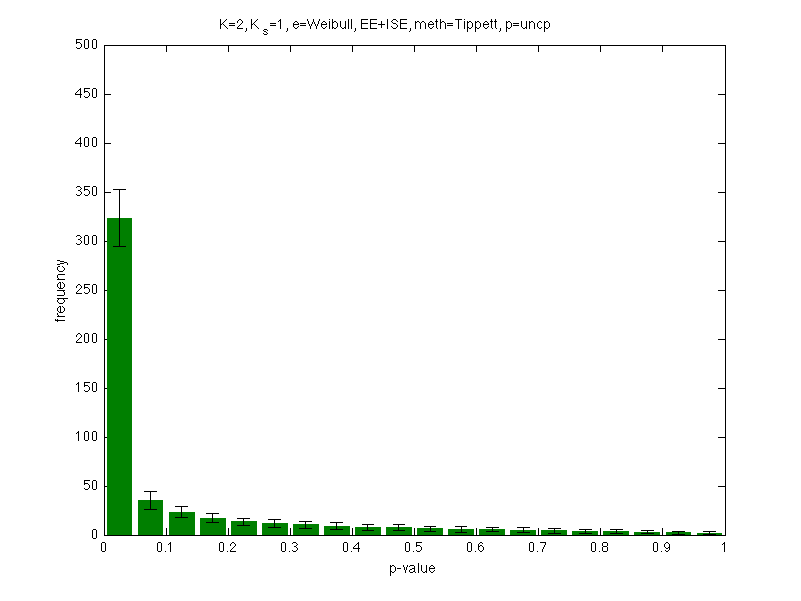

Supplement: Supplementary file 1 — Supporting Information [file HBM-37-1486-s001.zip › supporting_information/errorrates/histograms/images/hist_kset02_eWeibull_ee+ise_npc_tippett_uncp.png]

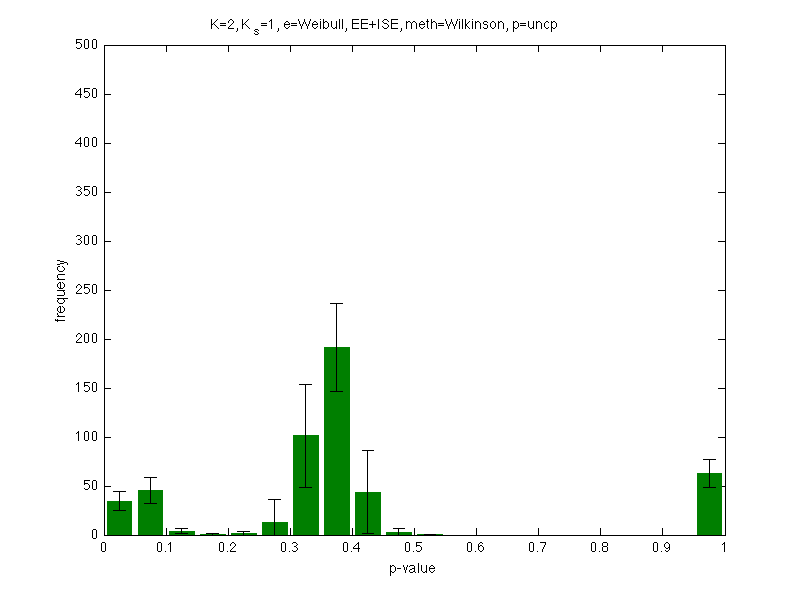

Supplement: Supplementary file 1 — Supporting Information [file HBM-37-1486-s001.zip › supporting_information/errorrates/histograms/images/hist_kset02_eWeibull_ee+ise_npc_wilkinson_uncp.png]

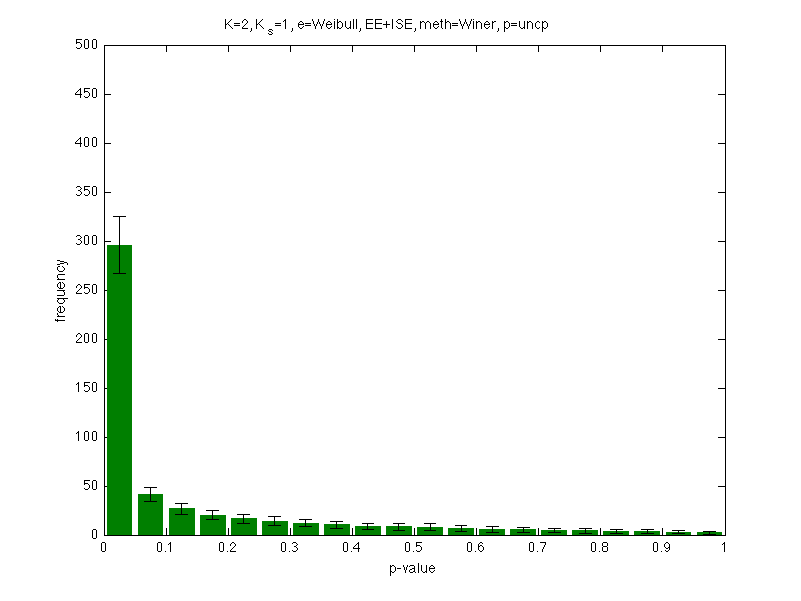

Supplement: Supplementary file 1 — Supporting Information [file HBM-37-1486-s001.zip › supporting_information/errorrates/histograms/images/hist_kset02_eWeibull_ee+ise_npc_winer_uncp.png]

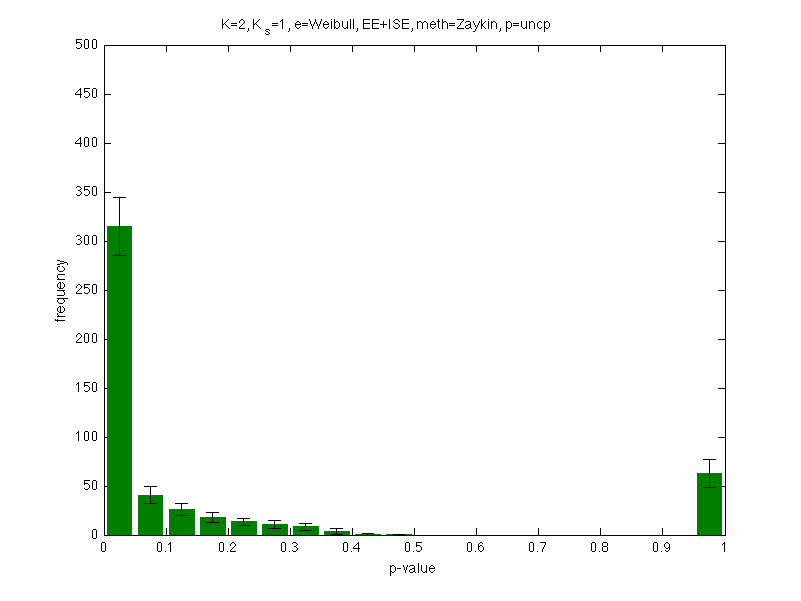

Supplement: Supplementary file 1 — Supporting Information [file HBM-37-1486-s001.zip › supporting_information/errorrates/histograms/images/hist_kset02_eWeibull_ee+ise_npc_zaykin_uncp.png]

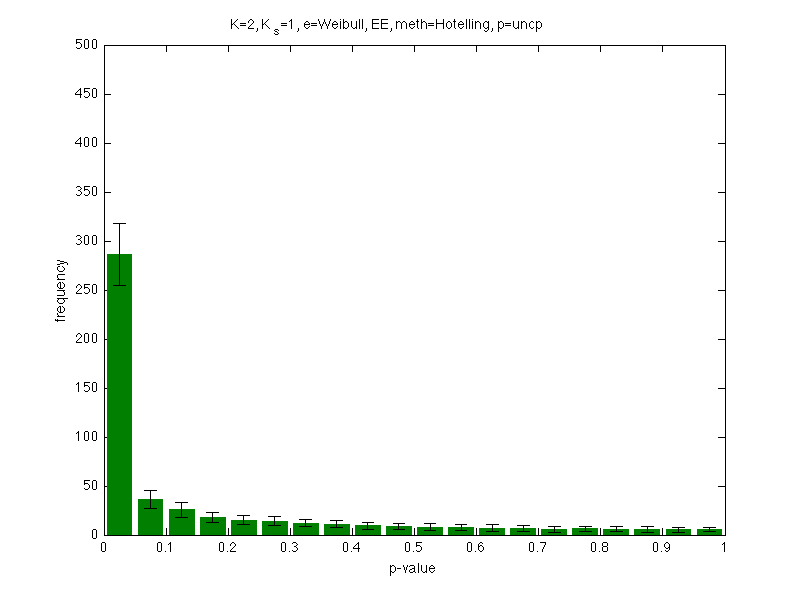

Supplement: Supplementary file 1 — Supporting Information [file HBM-37-1486-s001.zip › supporting_information/errorrates/histograms/images/hist_kset02_eWeibull_ee_mv_hotellingtsq_uncp.png]

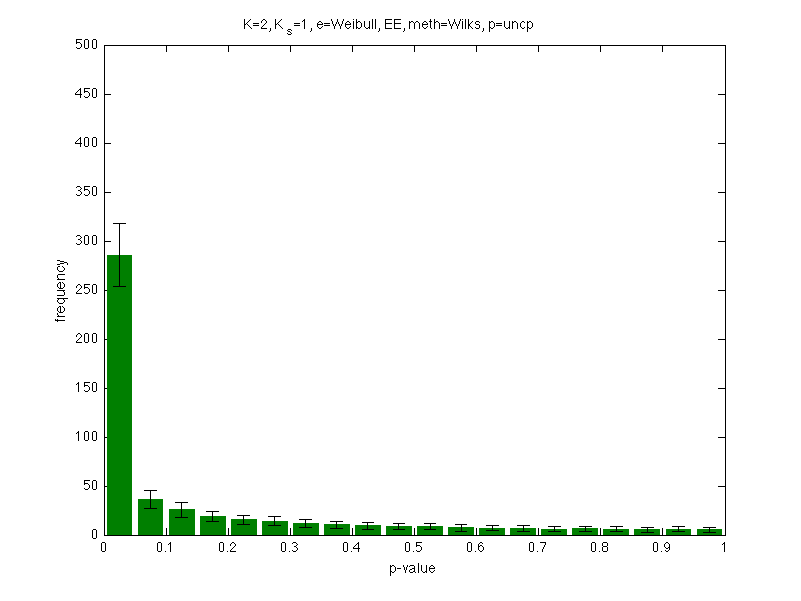

Supplement: Supplementary file 1 — Supporting Information [file HBM-37-1486-s001.zip › supporting_information/errorrates/histograms/images/hist_kset02_eWeibull_ee_mv_wilks_uncp.png]

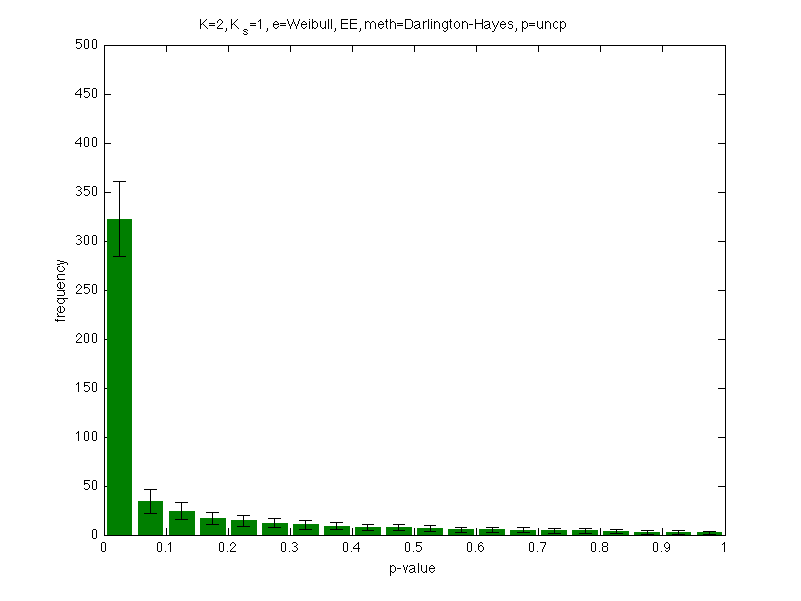

Supplement: Supplementary file 1 — Supporting Information [file HBM-37-1486-s001.zip › supporting_information/errorrates/histograms/images/hist_kset02_eWeibull_ee_npc_darlington-hayes_uncp.png]

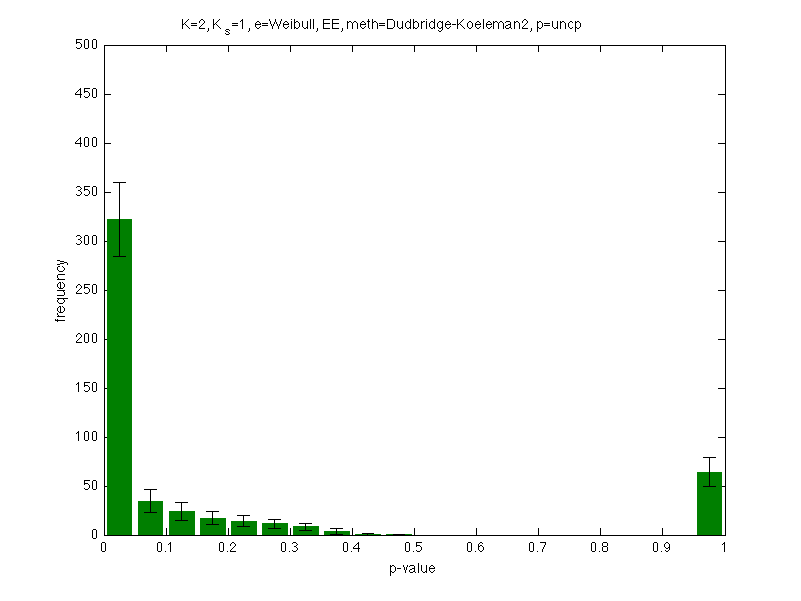

Supplement: Supplementary file 1 — Supporting Information [file HBM-37-1486-s001.zip › supporting_information/errorrates/histograms/images/hist_kset02_eWeibull_ee_npc_dudbridge-koeleman2_uncp.png]

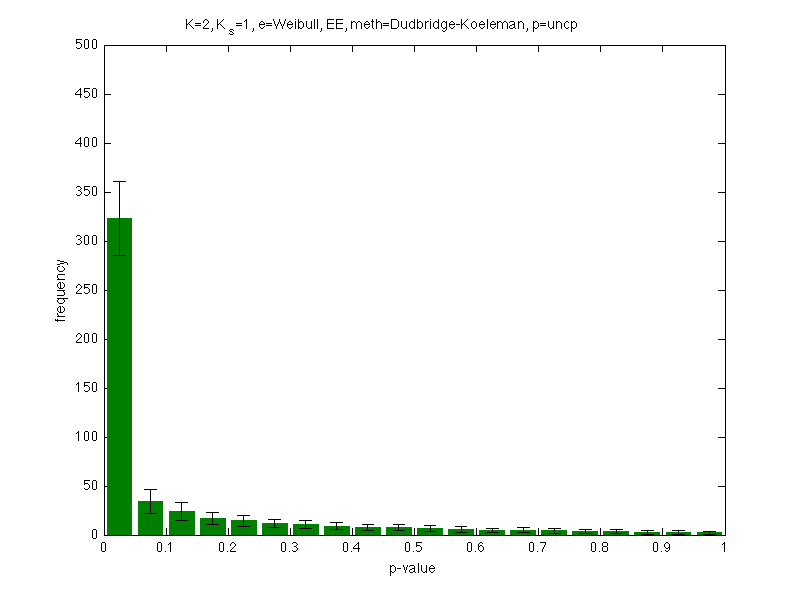

Supplement: Supplementary file 1 — Supporting Information [file HBM-37-1486-s001.zip › supporting_information/errorrates/histograms/images/hist_kset02_eWeibull_ee_npc_dudbridge-koeleman_uncp.png]

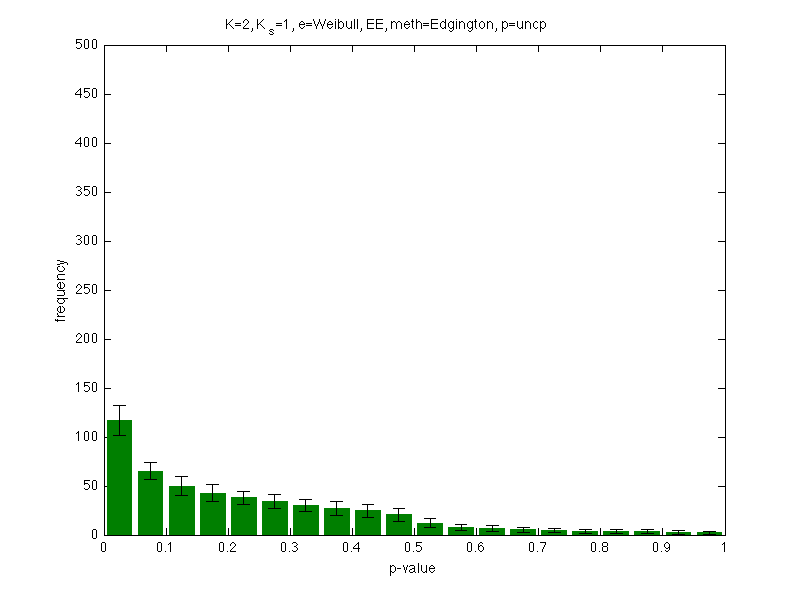

Supplement: Supplementary file 1 — Supporting Information [file HBM-37-1486-s001.zip › supporting_information/errorrates/histograms/images/hist_kset02_eWeibull_ee_npc_edgington_uncp.png]

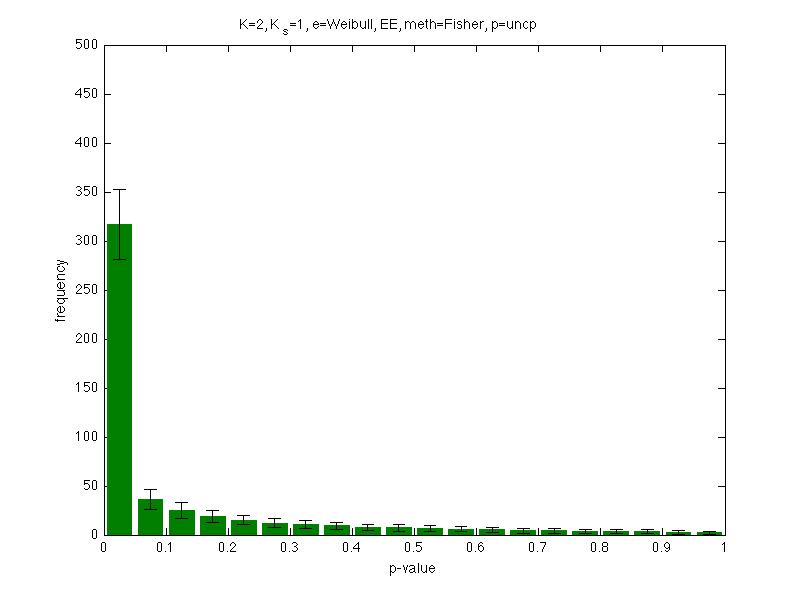

Supplement: Supplementary file 1 — Supporting Information [file HBM-37-1486-s001.zip › supporting_information/errorrates/histograms/images/hist_kset02_eWeibull_ee_npc_fisher_uncp.png]

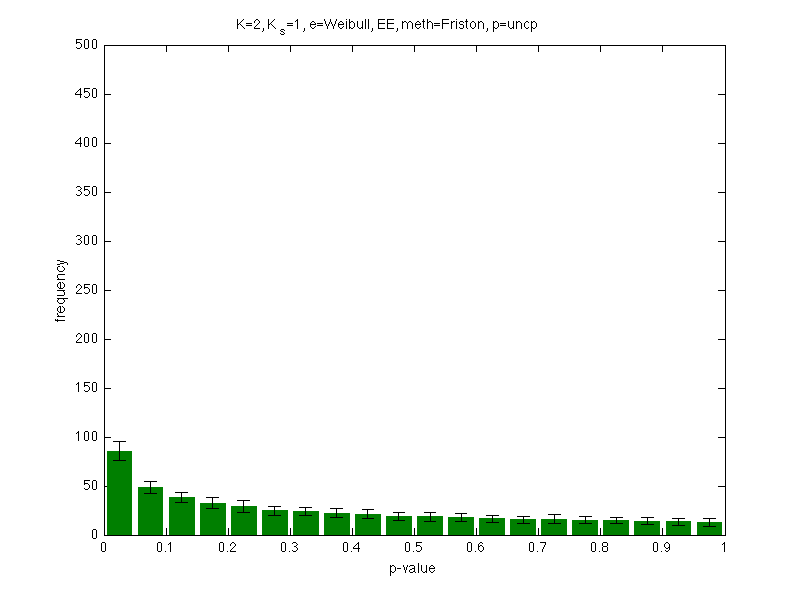

Supplement: Supplementary file 1 — Supporting Information [file HBM-37-1486-s001.zip › supporting_information/errorrates/histograms/images/hist_kset02_eWeibull_ee_npc_friston_uncp.png]

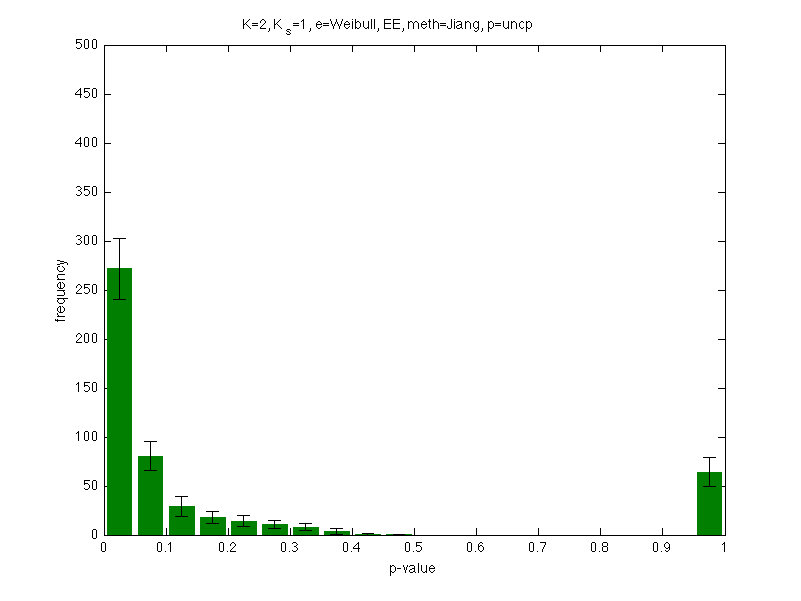

Supplement: Supplementary file 1 — Supporting Information [file HBM-37-1486-s001.zip › supporting_information/errorrates/histograms/images/hist_kset02_eWeibull_ee_npc_jiang_uncp.png]

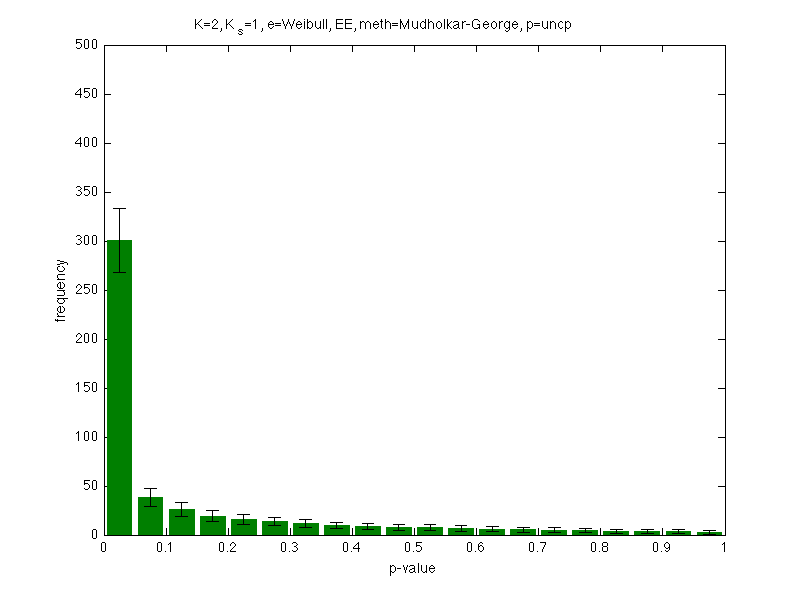

Supplement: Supplementary file 1 — Supporting Information [file HBM-37-1486-s001.zip › supporting_information/errorrates/histograms/images/hist_kset02_eWeibull_ee_npc_mudholkar-george_uncp.png]

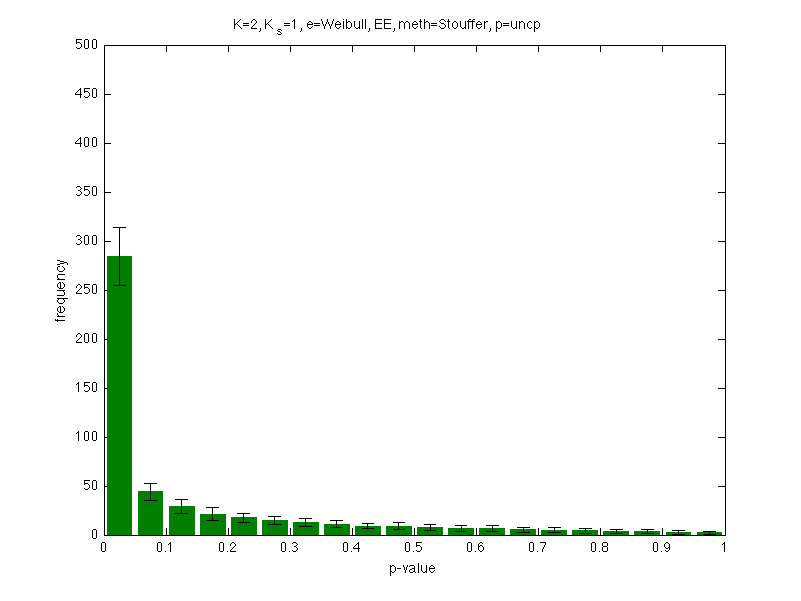

Supplement: Supplementary file 1 — Supporting Information [file HBM-37-1486-s001.zip › supporting_information/errorrates/histograms/images/hist_kset02_eWeibull_ee_npc_stouffer_uncp.png]

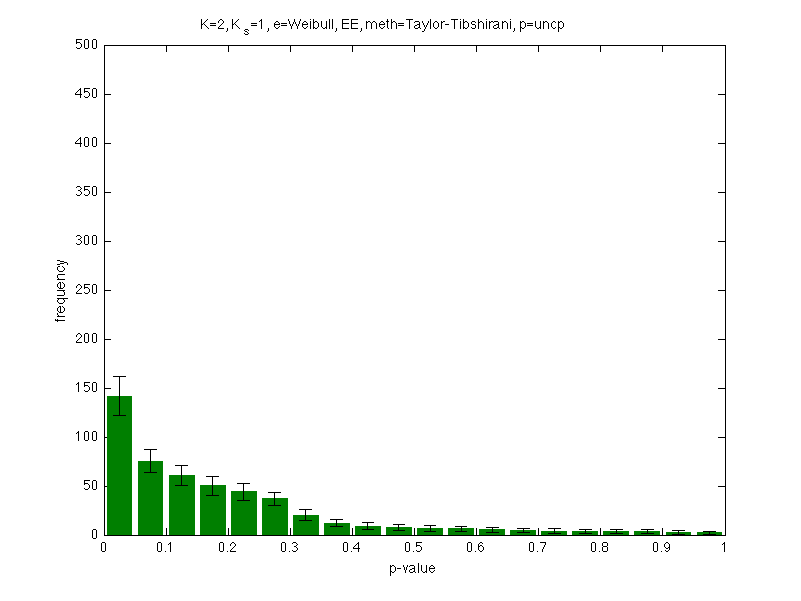

Supplement: Supplementary file 1 — Supporting Information [file HBM-37-1486-s001.zip › supporting_information/errorrates/histograms/images/hist_kset02_eWeibull_ee_npc_taylor-tibshirani_uncp.png]

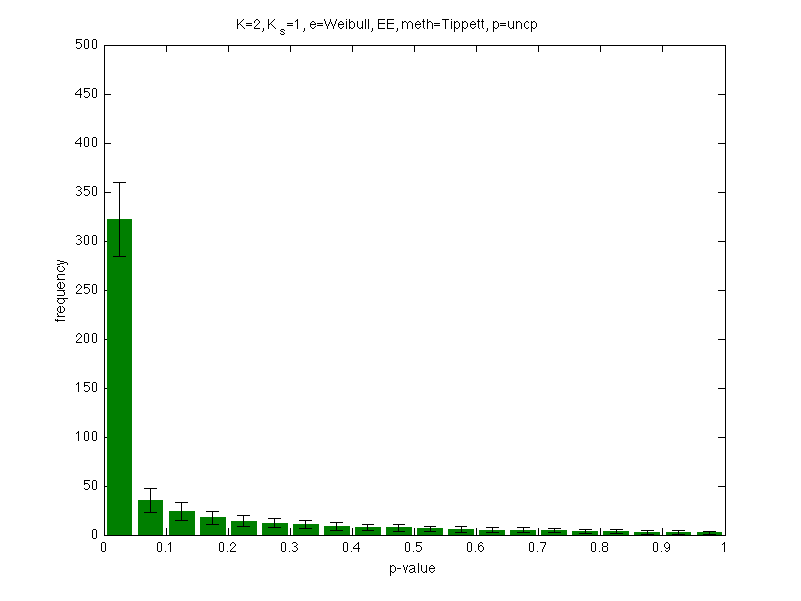

Supplement: Supplementary file 1 — Supporting Information [file HBM-37-1486-s001.zip › supporting_information/errorrates/histograms/images/hist_kset02_eWeibull_ee_npc_tippett_uncp.png]

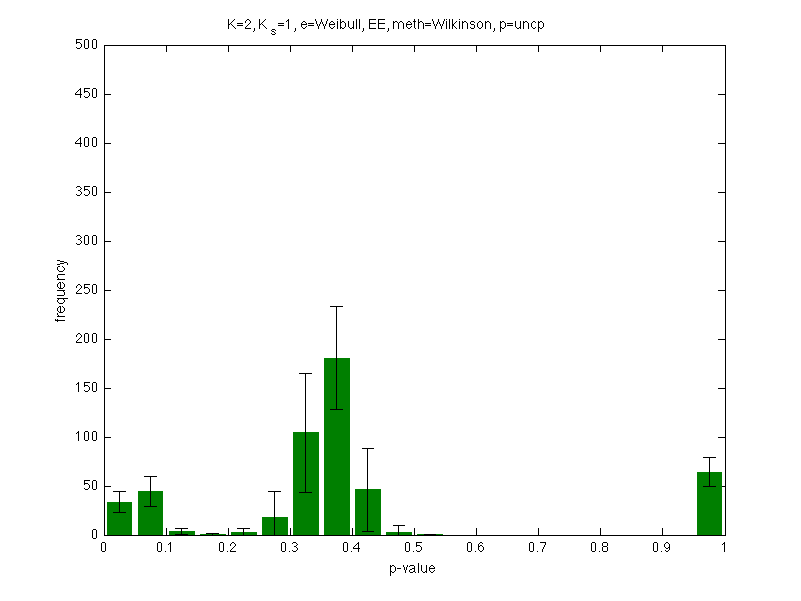

Supplement: Supplementary file 1 — Supporting Information [file HBM-37-1486-s001.zip › supporting_information/errorrates/histograms/images/hist_kset02_eWeibull_ee_npc_wilkinson_uncp.png]

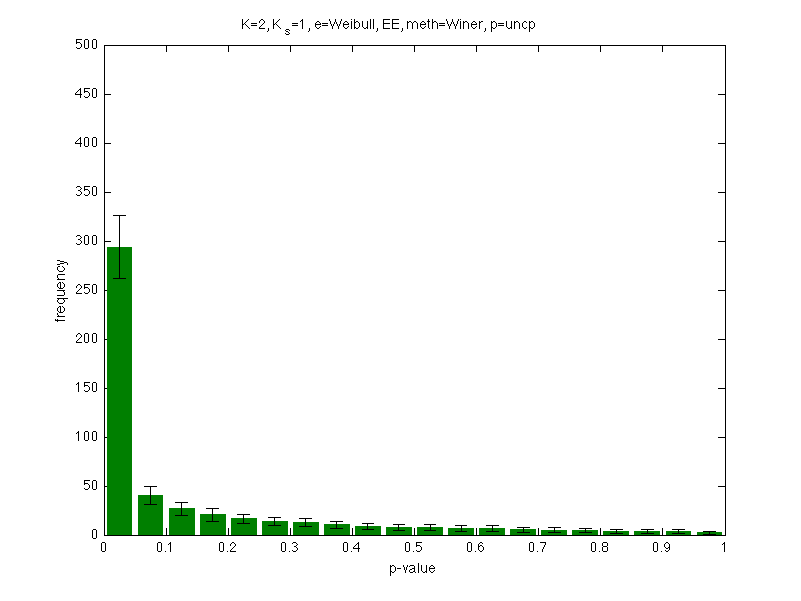

Supplement: Supplementary file 1 — Supporting Information [file HBM-37-1486-s001.zip › supporting_information/errorrates/histograms/images/hist_kset02_eWeibull_ee_npc_winer_uncp.png]

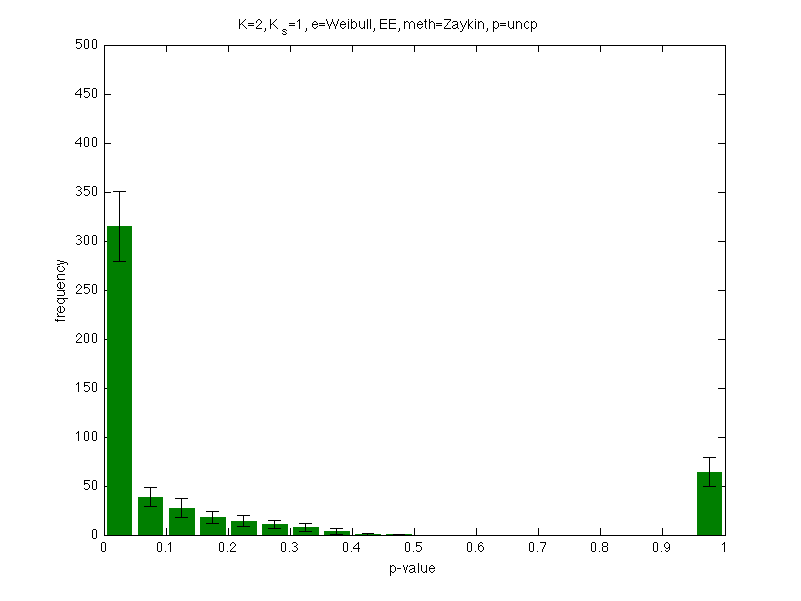

Supplement: Supplementary file 1 — Supporting Information [file HBM-37-1486-s001.zip › supporting_information/errorrates/histograms/images/hist_kset02_eWeibull_ee_npc_zaykin_uncp.png]

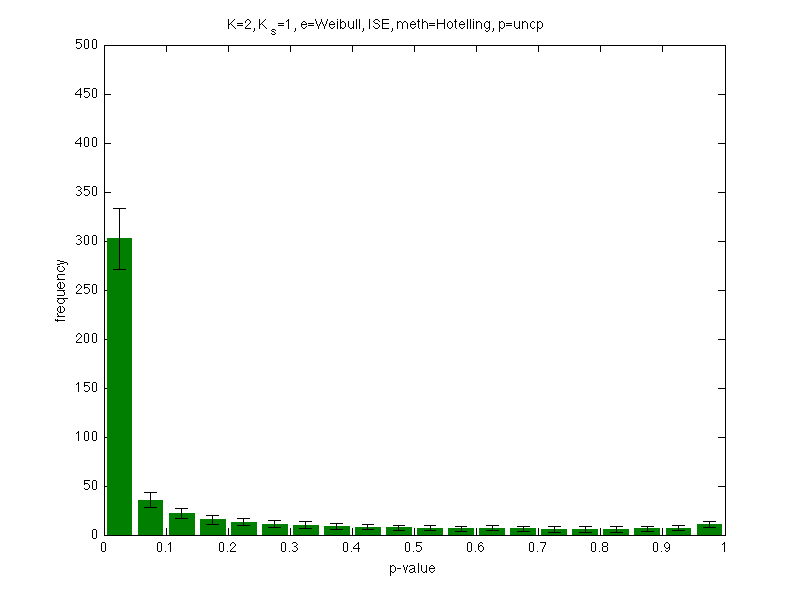

Supplement: Supplementary file 1 — Supporting Information [file HBM-37-1486-s001.zip › supporting_information/errorrates/histograms/images/hist_kset02_eWeibull_ise_mv_hotellingtsq_uncp.png]

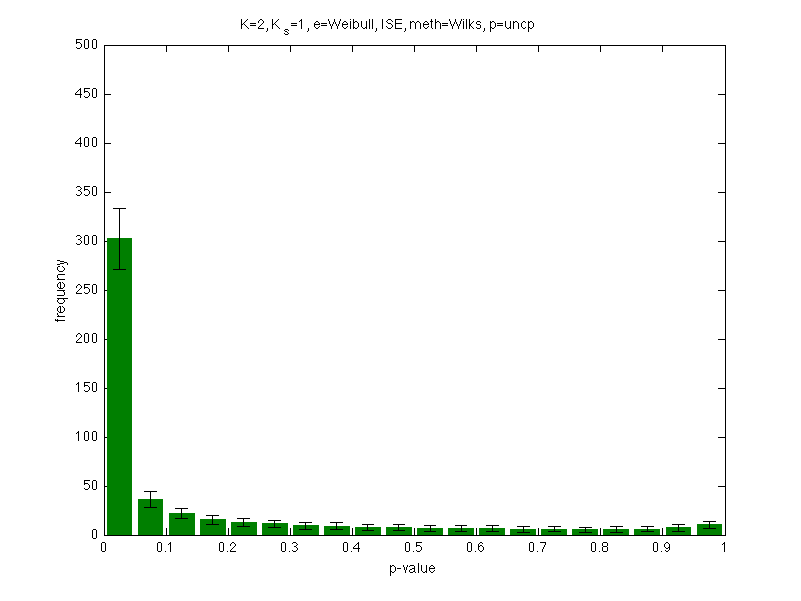

Supplement: Supplementary file 1 — Supporting Information [file HBM-37-1486-s001.zip › supporting_information/errorrates/histograms/images/hist_kset02_eWeibull_ise_mv_wilks_uncp.png]

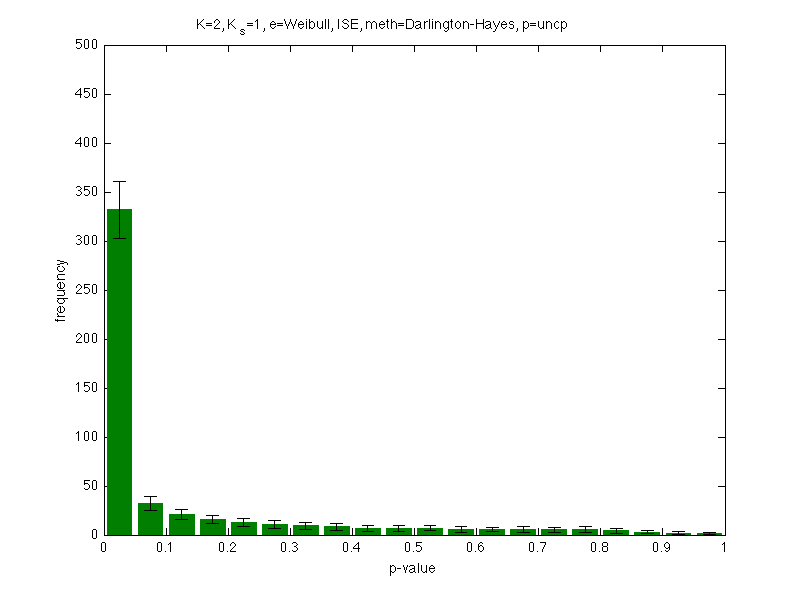

Supplement: Supplementary file 1 — Supporting Information [file HBM-37-1486-s001.zip › supporting_information/errorrates/histograms/images/hist_kset02_eWeibull_ise_npc_darlington-hayes_uncp.png]

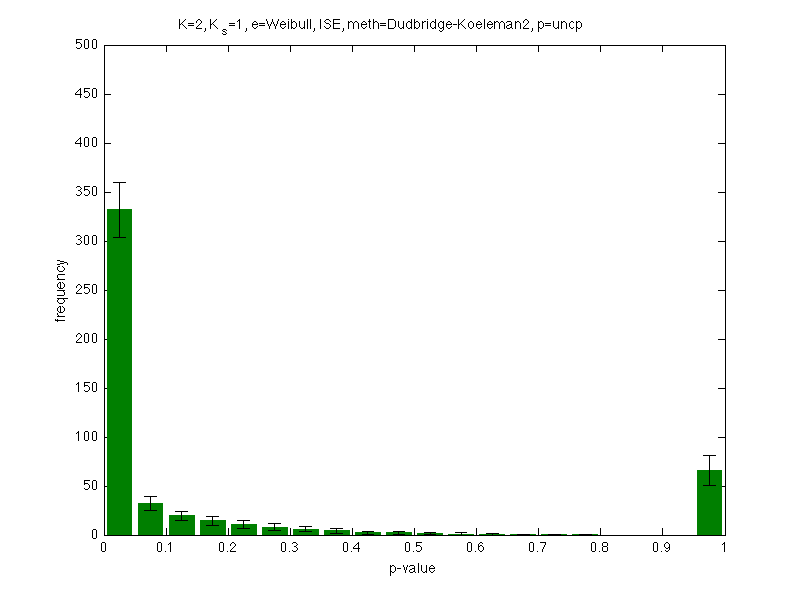

Supplement: Supplementary file 1 — Supporting Information [file HBM-37-1486-s001.zip › supporting_information/errorrates/histograms/images/hist_kset02_eWeibull_ise_npc_dudbridge-koeleman2_uncp.png]

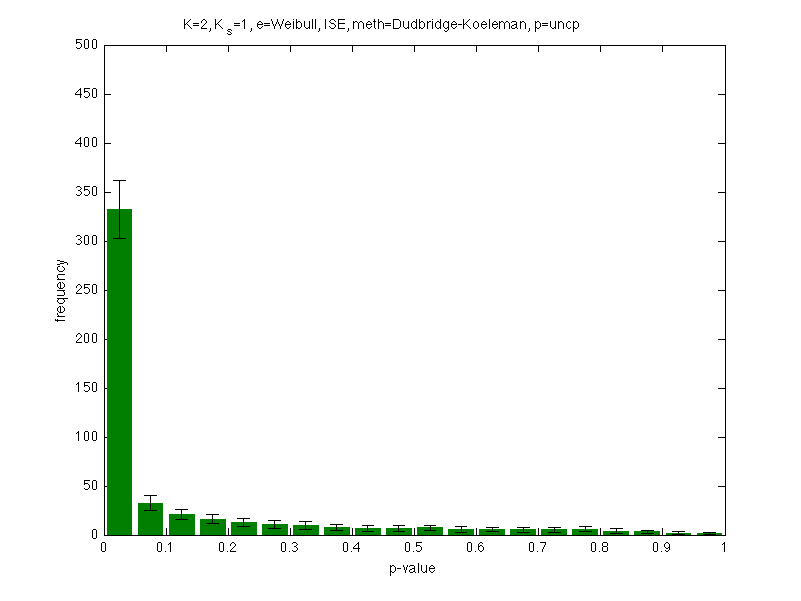

Supplement: Supplementary file 1 — Supporting Information [file HBM-37-1486-s001.zip › supporting_information/errorrates/histograms/images/hist_kset02_eWeibull_ise_npc_dudbridge-koeleman_uncp.png]

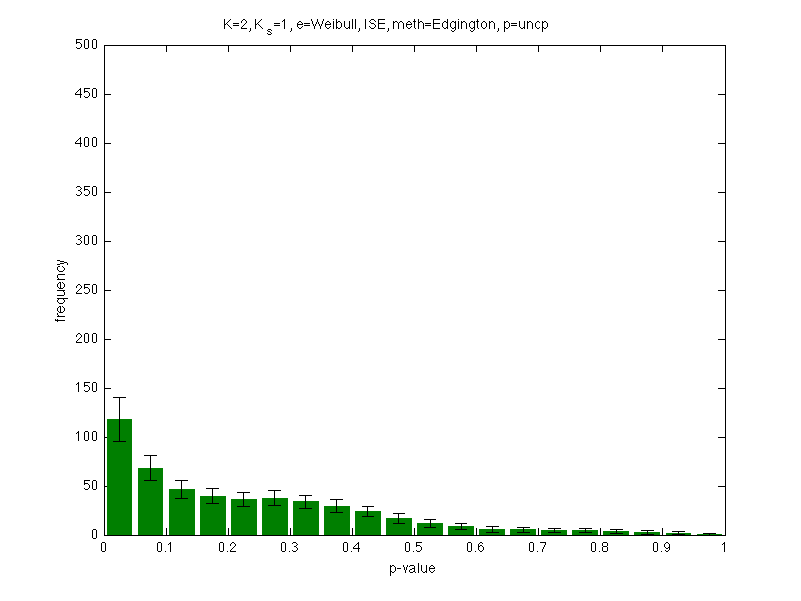

Supplement: Supplementary file 1 — Supporting Information [file HBM-37-1486-s001.zip › supporting_information/errorrates/histograms/images/hist_kset02_eWeibull_ise_npc_edgington_uncp.png]

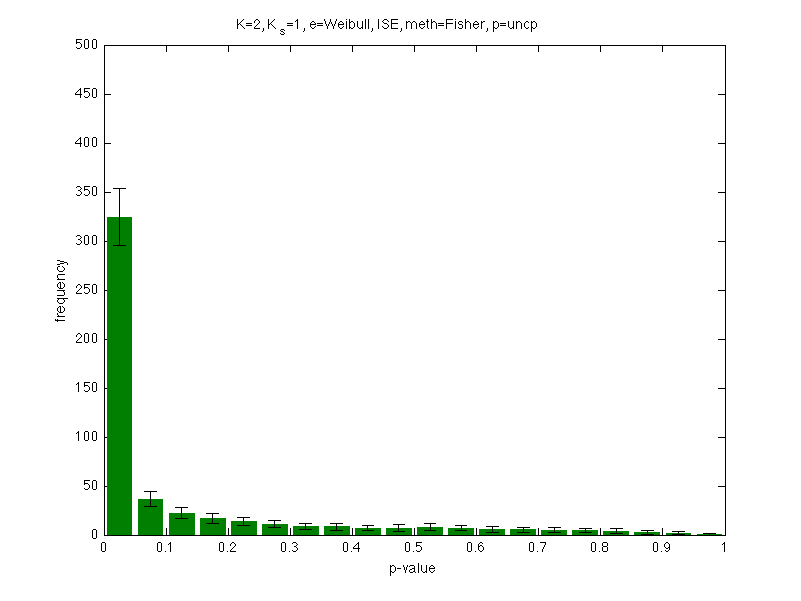

Supplement: Supplementary file 1 — Supporting Information [file HBM-37-1486-s001.zip › supporting_information/errorrates/histograms/images/hist_kset02_eWeibull_ise_npc_fisher_uncp.png]

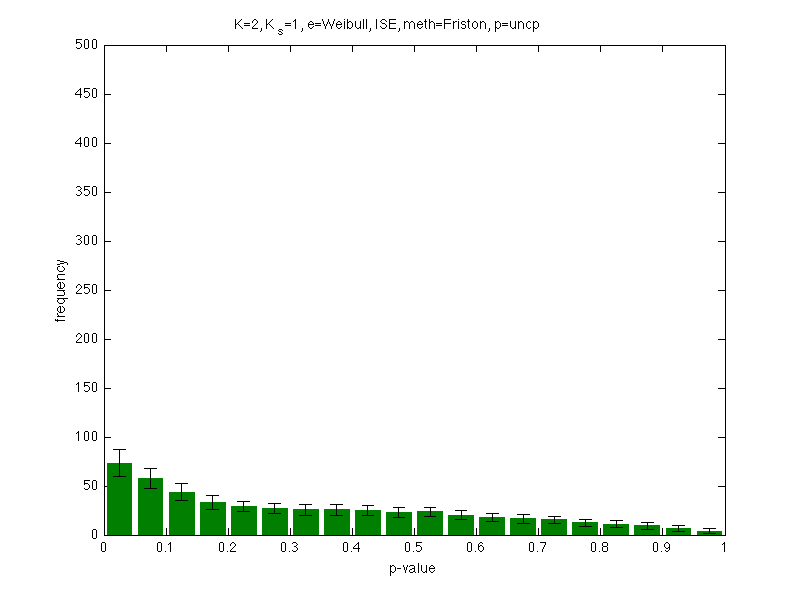

Supplement: Supplementary file 1 — Supporting Information [file HBM-37-1486-s001.zip › supporting_information/errorrates/histograms/images/hist_kset02_eWeibull_ise_npc_friston_uncp.png]

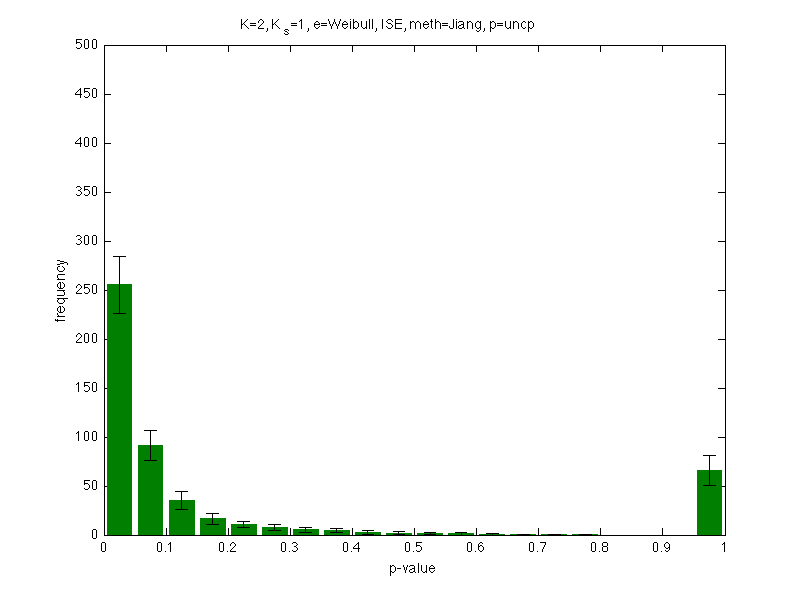

Supplement: Supplementary file 1 — Supporting Information [file HBM-37-1486-s001.zip › supporting_information/errorrates/histograms/images/hist_kset02_eWeibull_ise_npc_jiang_uncp.png]

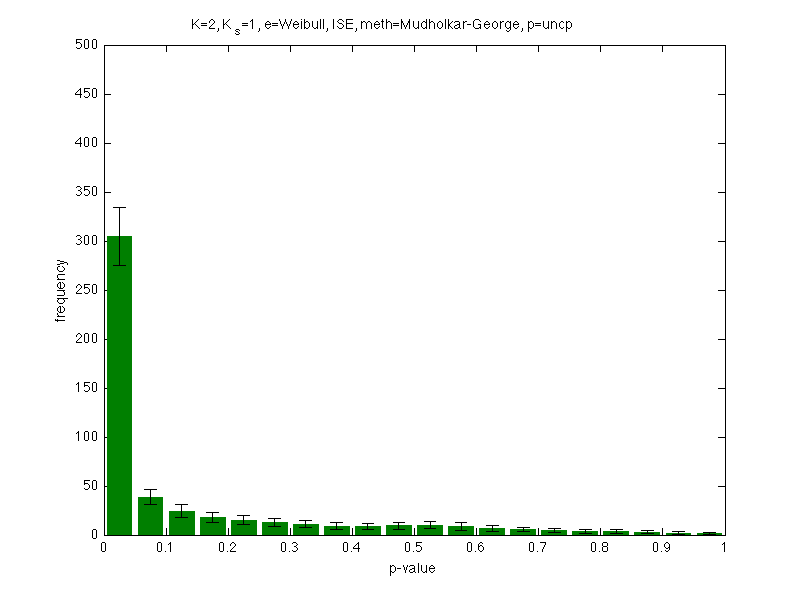

Supplement: Supplementary file 1 — Supporting Information [file HBM-37-1486-s001.zip › supporting_information/errorrates/histograms/images/hist_kset02_eWeibull_ise_npc_mudholkar-george_uncp.png]

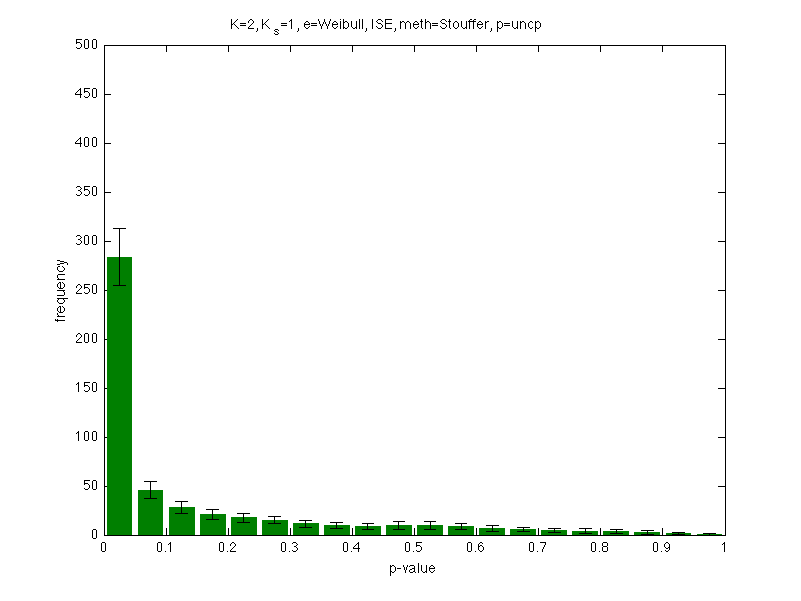

Supplement: Supplementary file 1 — Supporting Information [file HBM-37-1486-s001.zip › supporting_information/errorrates/histograms/images/hist_kset02_eWeibull_ise_npc_stouffer_uncp.png]

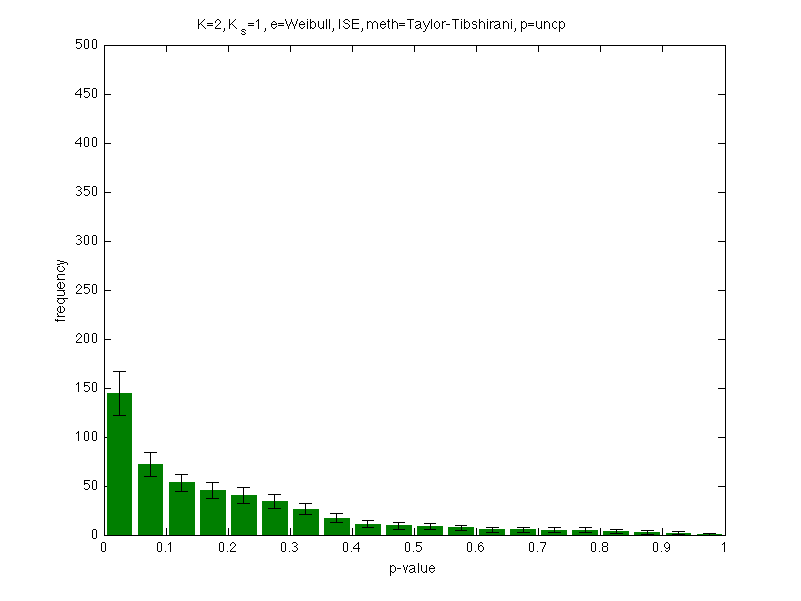

Supplement: Supplementary file 1 — Supporting Information [file HBM-37-1486-s001.zip › supporting_information/errorrates/histograms/images/hist_kset02_eWeibull_ise_npc_taylor-tibshirani_uncp.png]

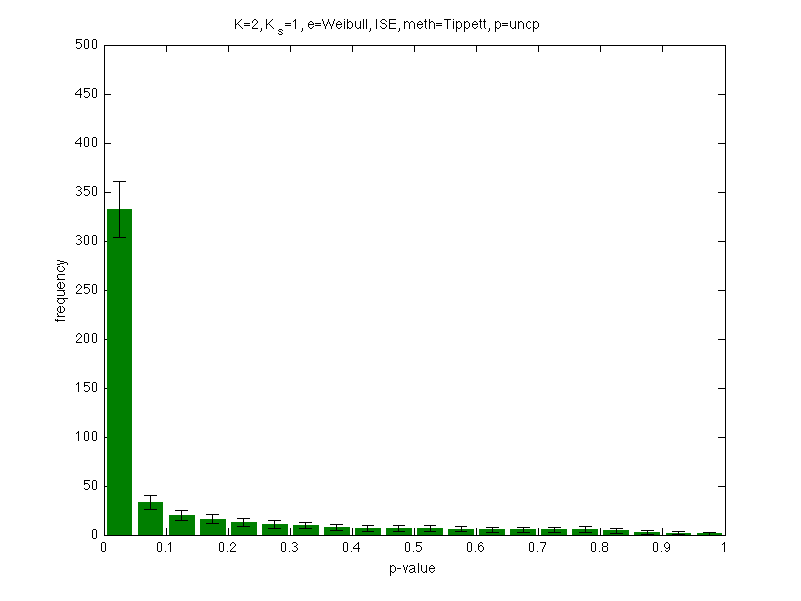

Supplement: Supplementary file 1 — Supporting Information [file HBM-37-1486-s001.zip › supporting_information/errorrates/histograms/images/hist_kset02_eWeibull_ise_npc_tippett_uncp.png]

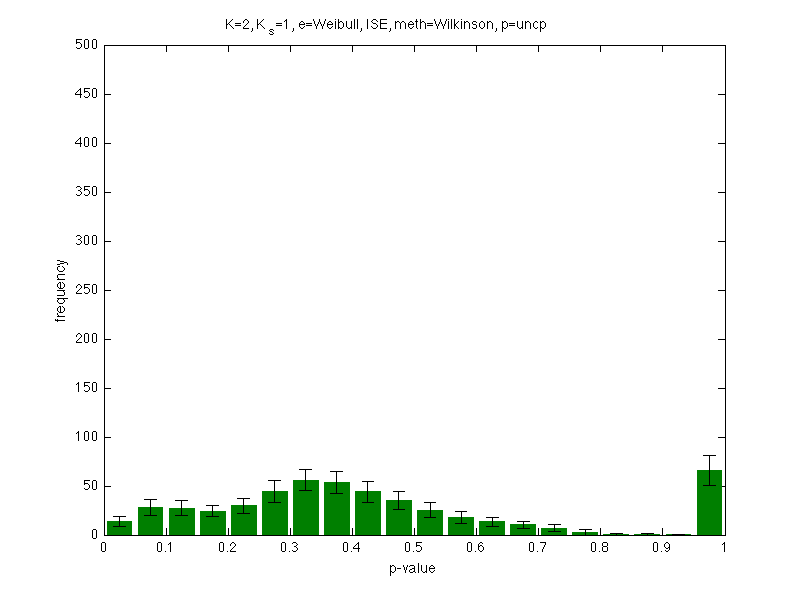

Supplement: Supplementary file 1 — Supporting Information [file HBM-37-1486-s001.zip › supporting_information/errorrates/histograms/images/hist_kset02_eWeibull_ise_npc_wilkinson_uncp.png]

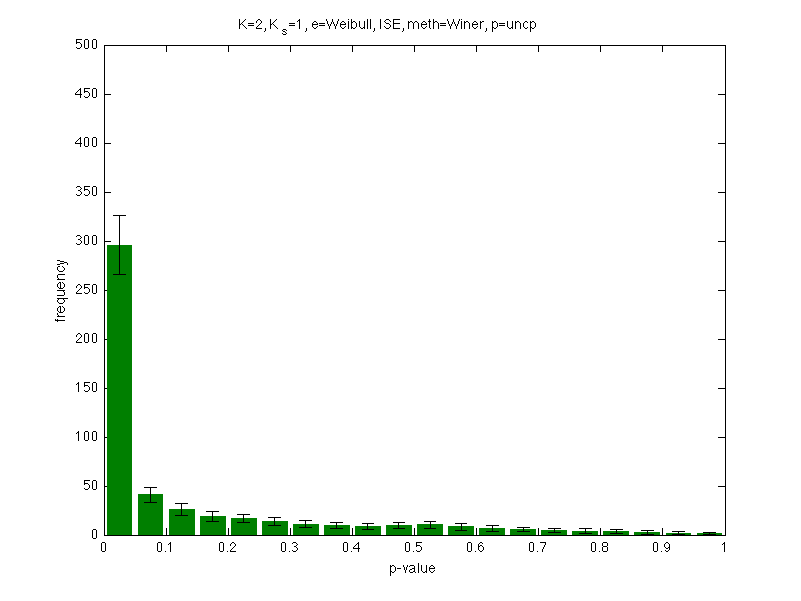

Supplement: Supplementary file 1 — Supporting Information [file HBM-37-1486-s001.zip › supporting_information/errorrates/histograms/images/hist_kset02_eWeibull_ise_npc_winer_uncp.png]

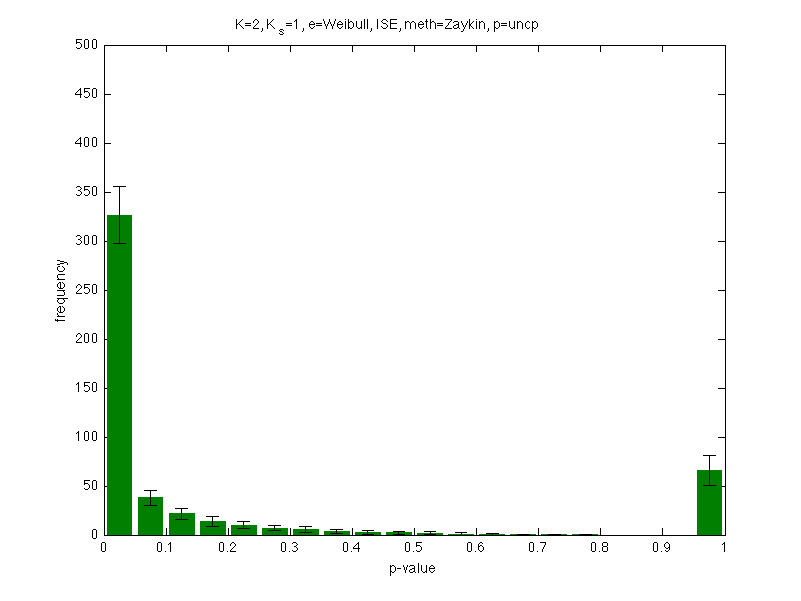

Supplement: Supplementary file 1 — Supporting Information [file HBM-37-1486-s001.zip › supporting_information/errorrates/histograms/images/hist_kset02_eWeibull_ise_npc_zaykin_uncp.png]

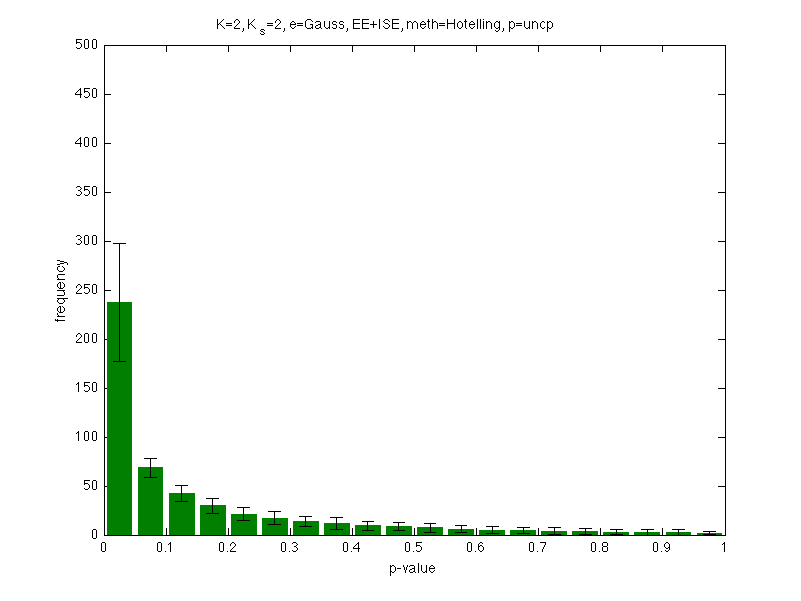

Supplement: Supplementary file 1 — Supporting Information [file HBM-37-1486-s001.zip › supporting_information/errorrates/histograms/images/hist_kset03_eGauss_ee+ise_mv_hotellingtsq_uncp.png]

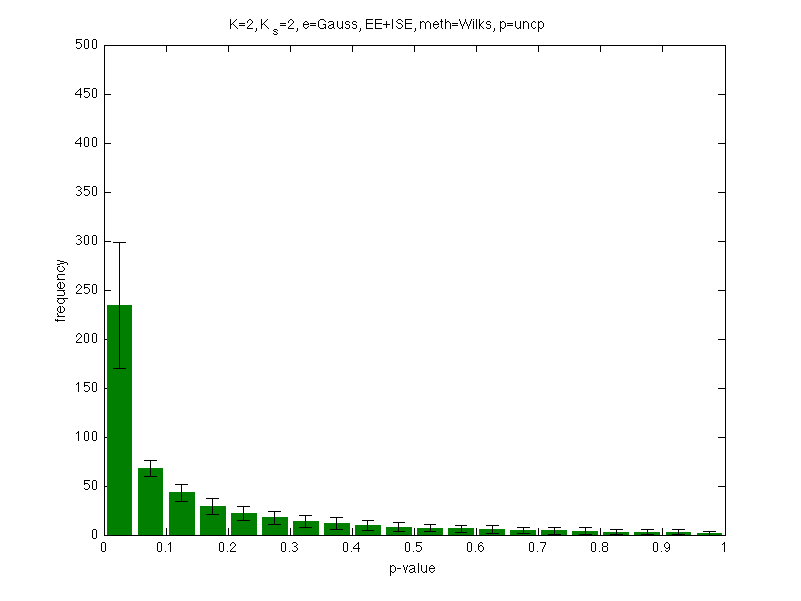

Supplement: Supplementary file 1 — Supporting Information [file HBM-37-1486-s001.zip › supporting_information/errorrates/histograms/images/hist_kset03_eGauss_ee+ise_mv_wilks_uncp.png]

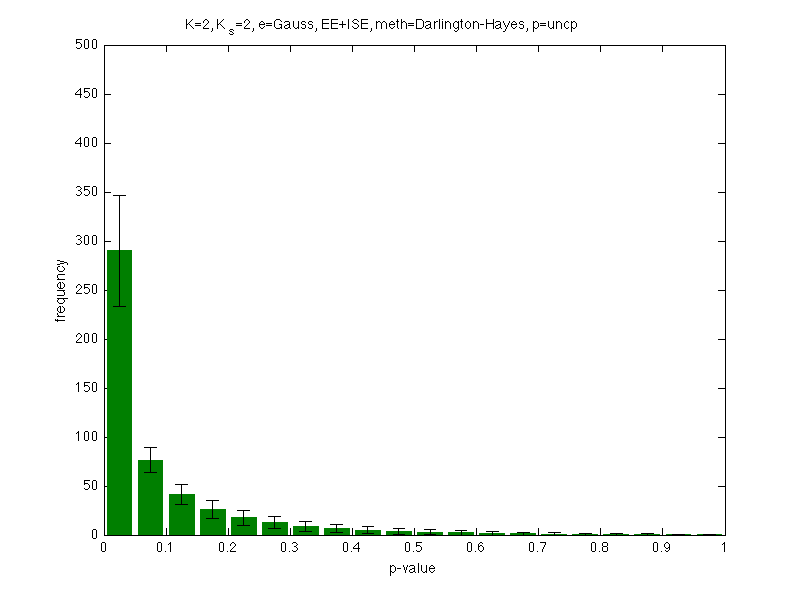

Supplement: Supplementary file 1 — Supporting Information [file HBM-37-1486-s001.zip › supporting_information/errorrates/histograms/images/hist_kset03_eGauss_ee+ise_npc_darlington-hayes_uncp.png]

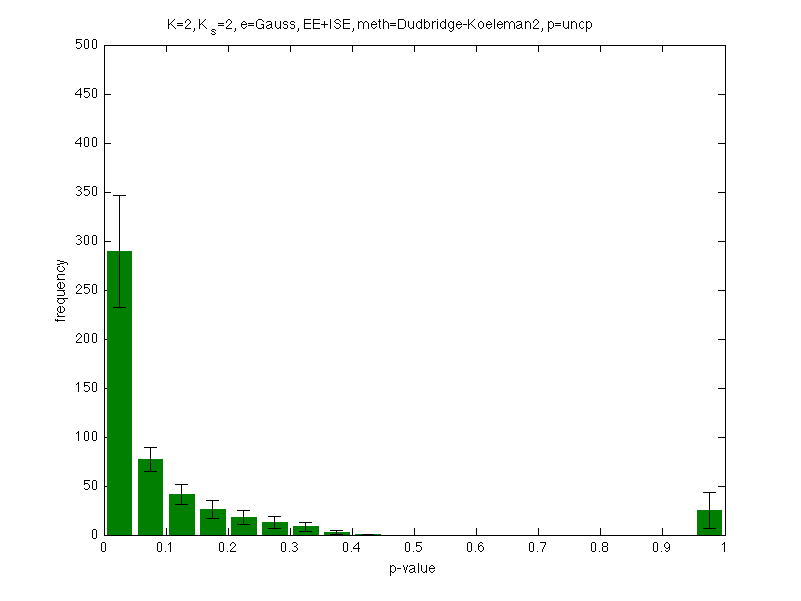

Supplement: Supplementary file 1 — Supporting Information [file HBM-37-1486-s001.zip › supporting_information/errorrates/histograms/images/hist_kset03_eGauss_ee+ise_npc_dudbridge-koeleman2_uncp.png]

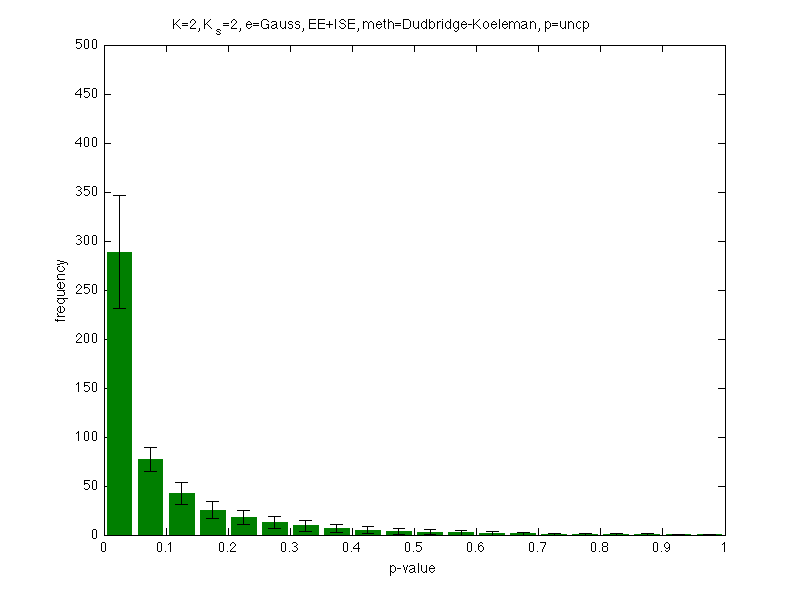

Supplement: Supplementary file 1 — Supporting Information [file HBM-37-1486-s001.zip › supporting_information/errorrates/histograms/images/hist_kset03_eGauss_ee+ise_npc_dudbridge-koeleman_uncp.png]

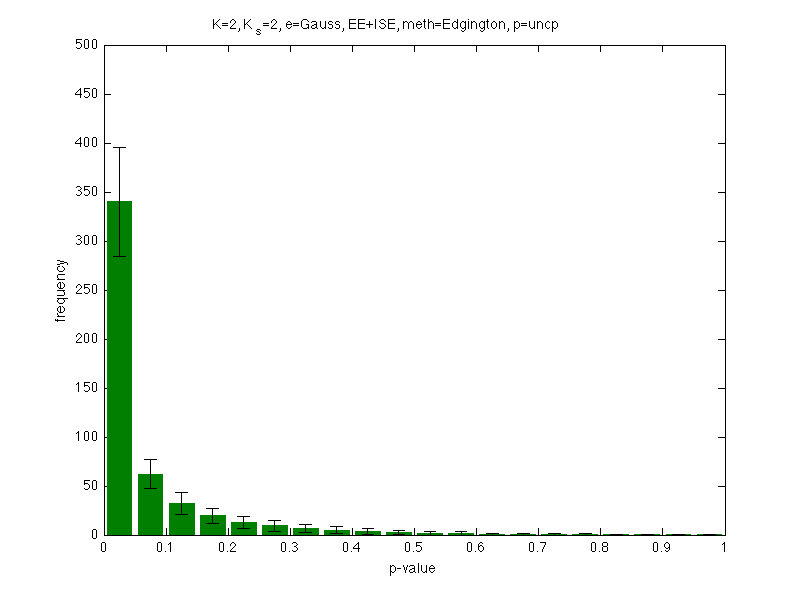

Supplement: Supplementary file 1 — Supporting Information [file HBM-37-1486-s001.zip › supporting_information/errorrates/histograms/images/hist_kset03_eGauss_ee+ise_npc_edgington_uncp.png]

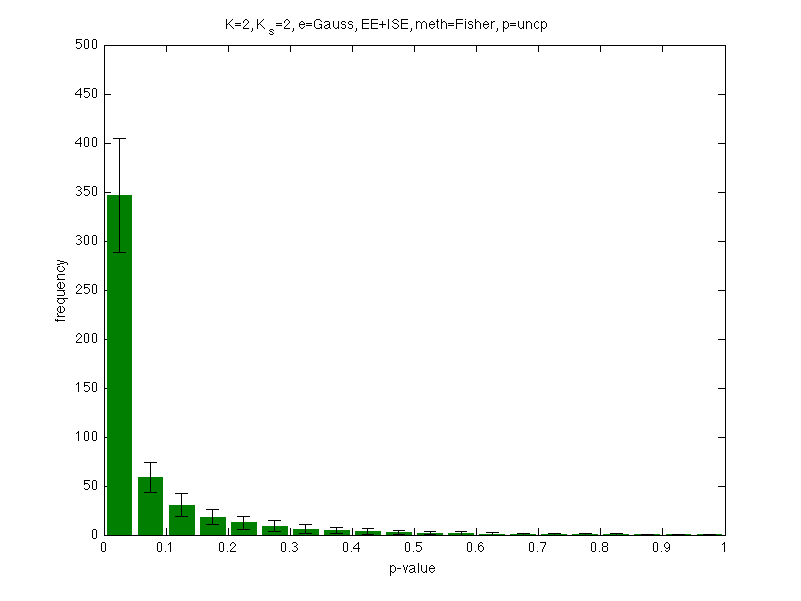

Supplement: Supplementary file 1 — Supporting Information [file HBM-37-1486-s001.zip › supporting_information/errorrates/histograms/images/hist_kset03_eGauss_ee+ise_npc_fisher_uncp.png]
