# Supplementary material for: Non‐parametric combination and related permutation tests for neuroimaging
Source: Hum Brain Mapp. 2016 Feb 5;37(4):1486–511. doi: 10.1002/hbm.23115 (PMC4783210; doi:10.1002/hbm.23115)
Supplement: Supplementary file 1 — Supporting Information [file HBM-37-1486-s001.zip › supporting_information/errorrates/histograms/simA_Gauss_ee_uncp.html]

Sim A, Gauss, EE, uncp

# Simulations A, Gauss, EE, uncp

---

| Method | ksig=0 / K=2 | ksig=0 / K=3 | ksig=0 / K=4 | ksig=0 / K=5 | ksig=0 / K=6 | ksig=0 / K=7 | ksig=0 / K=8 | ksig=0 / K=9 | ksig=0 / K=10 | ksig=0 / K=11 | ksig=0 / K=12 | ksig=0 / K=13 | ksig=0 / K=14 | ksig=0 / K=15 | ksig=0 / K=16 |
| --- | --- | --- | --- | --- | --- | --- | --- | --- | --- | --- | --- | --- | --- | --- | --- |
| Tippett | **0.0501** (0.0482-0.0520) | **0.0497** (0.0478-0.0517) | **0.0504** (0.0485-0.0523) | **0.0504** (0.0485-0.0524) | **0.0513** (0.0494-0.0532) | **0.0508** (0.0489-0.0528) | **0.0503** (0.0485-0.0523) | **0.0500** (0.0481-0.0520) | **0.0491** (0.0472-0.0510) | **0.0499** (0.0480-0.0519) | **0.0501** (0.0482-0.0520) | **0.0495** (0.0476-0.0514) | **0.0494** (0.0475-0.0513) | **0.0494** (0.0476-0.0514) | **0.0486** (0.0467-0.0505) |
| Fisher | **0.0500** (0.0481-0.0519) | **0.0496** (0.0478-0.0516) | **0.0497** (0.0478-0.0517) | **0.0506** (0.0487-0.0525) | **0.0495** (0.0477-0.0515) | **0.0497** (0.0478-0.0516) | **0.0490** (0.0471-0.0509) | **0.0486** (0.0468-0.0505) | **0.0489** (0.0470-0.0508) | **0.0498** (0.0479-0.0517) | **0.0498** (0.0479-0.0517) | **0.0502** (0.0484-0.0522) | **0.0496** (0.0478-0.0516) | **0.0507** (0.0488-0.0526) | **0.0496** (0.0477-0.0515) |
| Stouffer | **0.0496** (0.0478-0.0516) | **0.0491** (0.0472-0.0510) | **0.0494** (0.0476-0.0514) | **0.0500** (0.0481-0.0520) | **0.0488** (0.0470-0.0507) | **0.0487** (0.0468-0.0506) | **0.0482** (0.0464-0.0501) | **0.0497** (0.0478-0.0516) | **0.0484** (0.0466-0.0503) | **0.0498** (0.0479-0.0517) | **0.0489** (0.0471-0.0509) | **0.0507** (0.0488-0.0527) | **0.0508** (0.0489-0.0528) | **0.0502** (0.0483-0.0522) | **0.0501** (0.0483-0.0521) |
| Wilkinson | **0.0216** (0.0203-0.0229) | **0.0076** (0.0068-0.0084) | **0.0227** (0.0215-0.0241) | **0.0202** (0.0190-0.0215) | **0.0169** (0.0158-0.0181) | **0.0265** (0.0251-0.0279) | **0.0249** (0.0235-0.0263) | **0.0215** (0.0203-0.0228) | **0.0275** (0.0261-0.0290) | **0.0279** (0.0265-0.0294) | **0.0239** (0.0226-0.0253) | **0.0272** (0.0258-0.0287) | **0.0306** (0.0291-0.0322) | **0.0283** (0.0269-0.0298) | **0.0277** (0.0263-0.0291) |
| Winer | **0.0499** (0.0481-0.0519) | **0.0490** (0.0472-0.0510) | **0.0499** (0.0481-0.0519) | **0.0509** (0.0490-0.0529) | **0.0495** (0.0476-0.0514) | **0.0487** (0.0468-0.0506) | **0.0485** (0.0467-0.0504) | **0.0496** (0.0477-0.0515) | **0.0496** (0.0477-0.0515) | **0.0499** (0.0480-0.0519) | **0.0490** (0.0472-0.0510) | **0.0498** (0.0479-0.0518) | **0.0509** (0.0490-0.0529) | **0.0498** (0.0480-0.0518) | **0.0504** (0.0485-0.0523) |
| Edgington | **0.0484** (0.0466-0.0504) | **0.0501** (0.0482-0.0521) | **0.0502** (0.0483-0.0521) | **0.0501** (0.0482-0.0520) | **0.0495** (0.0476-0.0514) | **0.0481** (0.0463-0.0500) | **0.0481** (0.0462-0.0500) | **0.0492** (0.0473-0.0511) | **0.0486** (0.0467-0.0505) | **0.0494** (0.0475-0.0513) | **0.0486** (0.0467-0.0505) | **0.0498** (0.0479-0.0517) | **0.0508** (0.0489-0.0527) | **0.0503** (0.0485-0.0523) | **0.0504** (0.0485-0.0523) |
| Mudholkar-George | **0.0500** (0.0481-0.0519) | **0.0493** (0.0474-0.0512) | **0.0497** (0.0478-0.0516) | **0.0512** (0.0493-0.0532) | **0.0492** (0.0474-0.0512) | **0.0494** (0.0475-0.0513) | **0.0486** (0.0468-0.0505) | **0.0495** (0.0477-0.0515) | **0.0490** (0.0472-0.0510) | **0.0499** (0.0481-0.0519) | **0.0487** (0.0468-0.0506) | **0.0497** (0.0479-0.0517) | **0.0503** (0.0484-0.0523) | **0.0505** (0.0487-0.0525) | **0.0506** (0.0488-0.0526) |
| Friston | **0.0487** (0.0469-0.0506) | **0.0490** (0.0471-0.0509) | **0.0498** (0.0480-0.0518) | **0.0499** (0.0480-0.0518) | **0.0497** (0.0478-0.0517) | **0.0499** (0.0481-0.0519) | **0.0491** (0.0473-0.0510) | **0.0483** (0.0464-0.0502) | **0.0476** (0.0458-0.0495) | **0.0479** (0.0461-0.0498) | **0.0498** (0.0479-0.0518) | **0.0504** (0.0485-0.0524) | **0.0509** (0.0490-0.0528) | **0.0502** (0.0483-0.0521) | **0.0507** (0.0488-0.0526) |
| Darlington-Hayes | **0.0494** (0.0476-0.0514) | **0.0498** (0.0479-0.0517) | **0.0501** (0.0482-0.0520) | **0.0508** (0.0489-0.0528) | **0.0501** (0.0482-0.0520) | **0.0486** (0.0468-0.0506) | **0.0483** (0.0465-0.0502) | **0.0496** (0.0477-0.0515) | **0.0491** (0.0473-0.0510) | **0.0502** (0.0483-0.0521) | **0.0495** (0.0476-0.0514) | **0.0503** (0.0484-0.0523) | **0.0503** (0.0485-0.0523) | **0.0500** (0.0481-0.0519) | **0.0498** (0.0479-0.0517) |
| Zaykin | **0.0490** (0.0471-0.0509) | **0.0495** (0.0476-0.0514) | **0.0496** (0.0478-0.0516) | **0.0503** (0.0484-0.0523) | **0.0496** (0.0478-0.0516) | **0.0498** (0.0480-0.0518) | **0.0490** (0.0472-0.0509) | **0.0486** (0.0468-0.0505) | **0.0497** (0.0479-0.0517) | **0.0496** (0.0477-0.0515) | **0.0496** (0.0478-0.0516) | **0.0503** (0.0484-0.0522) | **0.0494** (0.0476-0.0514) | **0.0499** (0.0480-0.0519) | **0.0499** (0.0480-0.0518) |
| Dudbridge-Koeleman | **0.0497** (0.0478-0.0516) | **0.0493** (0.0475-0.0513) | **0.0502** (0.0484-0.0522) | **0.0503** (0.0484-0.0523) | **0.0498** (0.0480-0.0518) | **0.0490** (0.0471-0.0509) | **0.0485** (0.0466-0.0504) | **0.0493** (0.0475-0.0513) | **0.0488** (0.0470-0.0507) | **0.0500** (0.0481-0.0519) | **0.0495** (0.0476-0.0514) | **0.0505** (0.0486-0.0524) | **0.0499** (0.0480-0.0518) | **0.0506** (0.0487-0.0525) | **0.0500** (0.0481-0.0520) |
| Dudbridge-Koeleman2 | **0.0497** (0.0478-0.0516) | **0.0493** (0.0474-0.0512) | **0.0499** (0.0480-0.0518) | **0.0499** (0.0480-0.0519) | **0.0506** (0.0487-0.0526) | **0.0497** (0.0478-0.0516) | **0.0491** (0.0472-0.0510) | **0.0497** (0.0478-0.0516) | **0.0491** (0.0472-0.0510) | **0.0492** (0.0474-0.0512) | **0.0502** (0.0484-0.0522) | **0.0499** (0.0480-0.0518) | **0.0493** (0.0474-0.0512) | **0.0499** (0.0480-0.0518) | **0.0499** (0.0480-0.0518) |
| Taylor-Tibshirani | **0.0489** (0.0470-0.0508) | **0.0488** (0.0470-0.0508) | **0.0497** (0.0478-0.0516) | **0.0489** (0.0470-0.0508) | **0.0486** (0.0467-0.0505) | **0.0493** (0.0474-0.0512) | **0.0486** (0.0468-0.0506) | **0.0495** (0.0477-0.0515) | **0.0488** (0.0470-0.0507) | **0.0498** (0.0479-0.0517) | **0.0485** (0.0467-0.0505) | **0.0500** (0.0481-0.0519) | **0.0500** (0.0481-0.0519) | **0.0493** (0.0474-0.0512) | **0.0503** (0.0485-0.0523) |
| Jiang | **0.0492** (0.0474-0.0512) | **0.0487** (0.0468-0.0506) | **0.0492** (0.0474-0.0512) | **0.0499** (0.0480-0.0518) | **0.0498** (0.0479-0.0517) | **0.0493** (0.0475-0.0513) | **0.0490** (0.0471-0.0509) | **0.0491** (0.0472-0.0510) | **0.0501** (0.0482-0.0520) | **0.0498** (0.0479-0.0517) | **0.0501** (0.0482-0.0520) | **0.0508** (0.0489-0.0528) | **0.0498** (0.0480-0.0518) | **0.0487** (0.0468-0.0506) | **0.0497** (0.0478-0.0516) |
| Hotelling | **0.0502** (0.0483-0.0521) | **0.0503** (0.0484-0.0522) | **0.0503** (0.0485-0.0523) | **0.0503** (0.0485-0.0523) | **0.0505** (0.0486-0.0524) | **0.0500** (0.0481-0.0519) | **0.0496** (0.0477-0.0515) | **0.0499** (0.0480-0.0518) | **0.0506** (0.0487-0.0526) | **0.0515** (0.0496-0.0535) | **0.0517** (0.0498-0.0537) | **0.0492** (0.0473-0.0511) | **0.0504** (0.0485-0.0524) | **0.0504** (0.0485-0.0523) | **0.0504** (0.0485-0.0523) |
| Wilks | **0.0493** (0.0475-0.0513) | **0.0494** (0.0475-0.0513) | **0.0506** (0.0487-0.0525) | **0.0494** (0.0476-0.0514) | **0.0495** (0.0476-0.0514) | **0.0495** (0.0476-0.0514) | **0.0499** (0.0480-0.0518) | **0.0492** (0.0474-0.0512) | **0.0485** (0.0467-0.0504) | **0.0510** (0.0491-0.0529) | **0.0492** (0.0474-0.0512) | **0.0498** (0.0479-0.0517) | **0.0497** (0.0478-0.0516) | **0.0496** (0.0477-0.0515) | **0.0494** (0.0475-0.0513) |
