# Supplementary material for: Non‐parametric combination and related permutation tests for neuroimaging
Source: Hum Brain Mapp. 2016 Feb 5;37(4):1486–511. doi: 10.1002/hbm.23115 (PMC4783210; doi:10.1002/hbm.23115)
Supplement: Supplementary file 1 — Supporting Information [file HBM-37-1486-s001.zip › supporting_information/errorrates/histograms/simA_Gauss_ise_uncp.html]

Sim A, Gauss, ISE, uncp

# Simulations A, Gauss, ISE, uncp

---

| Method | ksig=0 / K=2 | ksig=0 / K=3 | ksig=0 / K=4 | ksig=0 / K=5 | ksig=0 / K=6 | ksig=0 / K=7 | ksig=0 / K=8 | ksig=0 / K=9 | ksig=0 / K=10 | ksig=0 / K=11 | ksig=0 / K=12 | ksig=0 / K=13 | ksig=0 / K=14 | ksig=0 / K=15 | ksig=0 / K=16 |
| --- | --- | --- | --- | --- | --- | --- | --- | --- | --- | --- | --- | --- | --- | --- | --- |
| Tippett | **0.0490** (0.0471-0.0509) | **0.0501** (0.0482-0.0520) | **0.0489** (0.0471-0.0508) | **0.0502** (0.0483-0.0521) | **0.0484** (0.0466-0.0504) | **0.0492** (0.0474-0.0512) | **0.0496** (0.0478-0.0516) | **0.0492** (0.0473-0.0511) | **0.0496** (0.0478-0.0516) | **0.0492** (0.0473-0.0511) | **0.0492** (0.0473-0.0511) | **0.0493** (0.0474-0.0512) | **0.0504** (0.0485-0.0523) | **0.0501** (0.0482-0.0520) | **0.0499** (0.0480-0.0518) |
| Fisher | **0.0483** (0.0464-0.0502) | **0.0487** (0.0469-0.0506) | **0.0481** (0.0463-0.0500) | **0.0482** (0.0464-0.0501) | **0.0480** (0.0461-0.0499) | **0.0463** (0.0445-0.0482) | **0.0461** (0.0443-0.0479) | **0.0482** (0.0464-0.0501) | **0.0489** (0.0470-0.0508) | **0.0484** (0.0465-0.0503) | **0.0489** (0.0470-0.0508) | **0.0482** (0.0463-0.0501) | **0.0484** (0.0466-0.0503) | **0.0485** (0.0467-0.0505) | **0.0474** (0.0456-0.0493) |
| Stouffer | **0.0496** (0.0478-0.0516) | **0.0490** (0.0471-0.0509) | **0.0489** (0.0470-0.0508) | **0.0487** (0.0469-0.0507) | **0.0469** (0.0451-0.0488) | **0.0471** (0.0453-0.0490) | **0.0476** (0.0457-0.0495) | **0.0490** (0.0472-0.0509) | **0.0489** (0.0471-0.0508) | **0.0485** (0.0467-0.0504) | **0.0486** (0.0468-0.0505) | **0.0505** (0.0486-0.0525) | **0.0498** (0.0479-0.0517) | **0.0493** (0.0474-0.0512) | **0.0497** (0.0478-0.0516) |
| Wilkinson | **0.0146** (0.0136-0.0157) | **0.0107** (0.0098-0.0116) | **0.0170** (0.0159-0.0182) | **0.0191** (0.0179-0.0203) | **0.0170** (0.0159-0.0182) | **0.0195** (0.0183-0.0207) | **0.0222** (0.0209-0.0235) | **0.0214** (0.0202-0.0227) | **0.0227** (0.0214-0.0240) | **0.0237** (0.0224-0.0251) | **0.0241** (0.0228-0.0255) | **0.0243** (0.0230-0.0257) | **0.0259** (0.0246-0.0274) | **0.0272** (0.0258-0.0287) | **0.0271** (0.0258-0.0286) |
| Winer | **0.0492** (0.0473-0.0511) | **0.0483** (0.0464-0.0502) | **0.0482** (0.0463-0.0501) | **0.0482** (0.0463-0.0501) | **0.0479** (0.0460-0.0498) | **0.0480** (0.0462-0.0499) | **0.0475** (0.0456-0.0494) | **0.0489** (0.0470-0.0508) | **0.0495** (0.0476-0.0514) | **0.0491** (0.0472-0.0510) | **0.0489** (0.0470-0.0508) | **0.0498** (0.0479-0.0517) | **0.0503** (0.0484-0.0523) | **0.0490** (0.0471-0.0509) | **0.0495** (0.0476-0.0514) |
| Edgington | **0.0491** (0.0473-0.0511) | **0.0482** (0.0463-0.0501) | **0.0499** (0.0480-0.0519) | **0.0492** (0.0473-0.0511) | **0.0468** (0.0450-0.0487) | **0.0481** (0.0463-0.0500) | **0.0491** (0.0472-0.0510) | **0.0488** (0.0469-0.0507) | **0.0494** (0.0476-0.0514) | **0.0479** (0.0461-0.0498) | **0.0488** (0.0469-0.0507) | **0.0502** (0.0483-0.0521) | **0.0490** (0.0472-0.0509) | **0.0484** (0.0466-0.0504) | **0.0490** (0.0472-0.0509) |
| Mudholkar-George | **0.0488** (0.0469-0.0507) | **0.0489** (0.0470-0.0508) | **0.0481** (0.0463-0.0501) | **0.0479** (0.0461-0.0498) | **0.0485** (0.0467-0.0505) | **0.0473** (0.0455-0.0492) | **0.0478** (0.0459-0.0497) | **0.0495** (0.0476-0.0514) | **0.0498** (0.0480-0.0518) | **0.0490** (0.0472-0.0510) | **0.0483** (0.0465-0.0502) | **0.0498** (0.0480-0.0518) | **0.0503** (0.0484-0.0522) | **0.0487** (0.0469-0.0507) | **0.0487** (0.0468-0.0506) |
| Friston | **0.0489** (0.0470-0.0508) | **0.0493** (0.0474-0.0512) | **0.0485** (0.0467-0.0504) | **0.0486** (0.0468-0.0505) | **0.0485** (0.0467-0.0504) | **0.0495** (0.0477-0.0515) | **0.0486** (0.0468-0.0505) | **0.0491** (0.0472-0.0510) | **0.0504** (0.0485-0.0523) | **0.0492** (0.0473-0.0511) | **0.0481** (0.0463-0.0501) | **0.0484** (0.0466-0.0504) | **0.0478** (0.0460-0.0497) | **0.0479** (0.0460-0.0498) | **0.0471** (0.0453-0.0490) |
| Darlington-Hayes | **0.0491** (0.0472-0.0510) | **0.0475** (0.0457-0.0494) | **0.0481** (0.0462-0.0500) | **0.0475** (0.0457-0.0494) | **0.0485** (0.0467-0.0504) | **0.0468** (0.0450-0.0487) | **0.0477** (0.0458-0.0496) | **0.0470** (0.0452-0.0489) | **0.0473** (0.0455-0.0492) | **0.0474** (0.0456-0.0493) | **0.0474** (0.0456-0.0493) | **0.0468** (0.0450-0.0487) | **0.0471** (0.0453-0.0490) | **0.0468** (0.0450-0.0487) | **0.0475** (0.0457-0.0494) |
| Zaykin | **0.0488** (0.0469-0.0507) | **0.0481** (0.0462-0.0500) | **0.0478** (0.0460-0.0497) | **0.0480** (0.0462-0.0499) | **0.0473** (0.0455-0.0492) | **0.0465** (0.0446-0.0483) | **0.0464** (0.0446-0.0483) | **0.0470** (0.0451-0.0488) | **0.0473** (0.0454-0.0492) | **0.0476** (0.0458-0.0495) | **0.0475** (0.0457-0.0494) | **0.0467** (0.0449-0.0486) | **0.0477** (0.0459-0.0496) | **0.0473** (0.0455-0.0492) | **0.0470** (0.0452-0.0489) |
| Dudbridge-Koeleman | **0.0489** (0.0470-0.0508) | **0.0481** (0.0463-0.0500) | **0.0479** (0.0461-0.0498) | **0.0488** (0.0470-0.0507) | **0.0484** (0.0466-0.0503) | **0.0466** (0.0448-0.0485) | **0.0477** (0.0459-0.0496) | **0.0480** (0.0462-0.0499) | **0.0477** (0.0458-0.0496) | **0.0475** (0.0457-0.0494) | **0.0478** (0.0460-0.0497) | **0.0472** (0.0454-0.0491) | **0.0477** (0.0458-0.0496) | **0.0484** (0.0465-0.0503) | **0.0464** (0.0446-0.0483) |
| Dudbridge-Koeleman2 | **0.0495** (0.0477-0.0515) | **0.0490** (0.0471-0.0509) | **0.0478** (0.0460-0.0497) | **0.0488** (0.0469-0.0507) | **0.0486** (0.0467-0.0505) | **0.0470** (0.0452-0.0489) | **0.0478** (0.0460-0.0497) | **0.0473** (0.0455-0.0492) | **0.0477** (0.0458-0.0496) | **0.0484** (0.0466-0.0503) | **0.0478** (0.0460-0.0497) | **0.0463** (0.0445-0.0482) | **0.0472** (0.0454-0.0491) | **0.0472** (0.0454-0.0491) | **0.0475** (0.0457-0.0494) |
| Taylor-Tibshirani | **0.0496** (0.0478-0.0516) | **0.0490** (0.0471-0.0509) | **0.0488** (0.0469-0.0507) | **0.0483** (0.0465-0.0503) | **0.0469** (0.0451-0.0488) | **0.0474** (0.0456-0.0493) | **0.0472** (0.0454-0.0491) | **0.0488** (0.0469-0.0507) | **0.0495** (0.0477-0.0515) | **0.0498** (0.0479-0.0517) | **0.0489** (0.0470-0.0508) | **0.0492** (0.0474-0.0512) | **0.0498** (0.0479-0.0517) | **0.0491** (0.0473-0.0510) | **0.0484** (0.0465-0.0503) |
| Jiang | **0.0486** (0.0468-0.0505) | **0.0485** (0.0467-0.0505) | **0.0481** (0.0463-0.0500) | **0.0479** (0.0461-0.0498) | **0.0472** (0.0454-0.0491) | **0.0479** (0.0460-0.0498) | **0.0476** (0.0457-0.0495) | **0.0468** (0.0450-0.0487) | **0.0473** (0.0455-0.0492) | **0.0479** (0.0460-0.0498) | **0.0472** (0.0454-0.0491) | **0.0473** (0.0454-0.0492) | **0.0474** (0.0456-0.0493) | **0.0475** (0.0457-0.0494) | **0.0476** (0.0457-0.0495) |
| Hotelling | **0.0490** (0.0472-0.0510) | **0.0496** (0.0477-0.0515) | **0.0497** (0.0478-0.0516) | **0.0490** (0.0472-0.0509) | **0.0491** (0.0472-0.0510) | **0.0485** (0.0466-0.0504) | **0.0499** (0.0480-0.0518) | **0.0486** (0.0467-0.0505) | **0.0498** (0.0479-0.0518) | **0.0493** (0.0475-0.0513) | **0.0500** (0.0481-0.0519) | **0.0508** (0.0489-0.0527) | **0.0506** (0.0487-0.0526) | **0.0502** (0.0483-0.0522) | **0.0497** (0.0478-0.0516) |
| Wilks | **0.0490** (0.0472-0.0509) | **0.0499** (0.0480-0.0518) | **0.0508** (0.0489-0.0527) | **0.0496** (0.0478-0.0516) | **0.0505** (0.0486-0.0524) | **0.0507** (0.0489-0.0527) | **0.0499** (0.0480-0.0518) | **0.0501** (0.0482-0.0520) | **0.0486** (0.0468-0.0505) | **0.0500** (0.0481-0.0519) | **0.0500** (0.0481-0.0519) | **0.0504** (0.0486-0.0524) | **0.0494** (0.0475-0.0513) | **0.0487** (0.0469-0.0507) | **0.0482** (0.0464-0.0501) |
