# Supplementary material for: Non‐parametric combination and related permutation tests for neuroimaging
Source: Hum Brain Mapp. 2016 Feb 5;37(4):1486–511. doi: 10.1002/hbm.23115 (PMC4783210; doi:10.1002/hbm.23115)
Supplement: Supplementary file 1 — Supporting Information [file HBM-37-1486-s001.zip › supporting_information/errorrates/histograms/simA_Weibull_ise_uncp.html]

Sim A, Weibull, ISE, uncp

# Simulations A, Weibull, ISE, uncp

---

| Method | ksig=0 / K=2 | ksig=0 / K=3 | ksig=0 / K=4 | ksig=0 / K=5 | ksig=0 / K=6 | ksig=0 / K=7 | ksig=0 / K=8 | ksig=0 / K=9 | ksig=0 / K=10 | ksig=0 / K=11 | ksig=0 / K=12 | ksig=0 / K=13 | ksig=0 / K=14 | ksig=0 / K=15 | ksig=0 / K=16 |
| --- | --- | --- | --- | --- | --- | --- | --- | --- | --- | --- | --- | --- | --- | --- | --- |
| Tippett | **0.0325** (0.0310-0.0341) | **0.0309** (0.0294-0.0324) | **0.0317** (0.0302-0.0333) | **0.0324** (0.0309-0.0340) | **0.0318** (0.0303-0.0334) | **0.0323** (0.0308-0.0339) | **0.0335** (0.0320-0.0351) | **0.0334** (0.0319-0.0350) | **0.0330** (0.0315-0.0346) | **0.0326** (0.0311-0.0342) | **0.0336** (0.0321-0.0353) | **0.0337** (0.0321-0.0353) | **0.0326** (0.0311-0.0342) | **0.0333** (0.0318-0.0349) | **0.0335** (0.0320-0.0352) |
| Fisher | **0.0382** (0.0366-0.0399) | **0.0417** (0.0400-0.0435) | **0.0416** (0.0399-0.0434) | **0.0422** (0.0405-0.0440) | **0.0428** (0.0410-0.0446) | **0.0437** (0.0419-0.0455) | **0.0436** (0.0418-0.0454) | **0.0441** (0.0423-0.0459) | **0.0443** (0.0426-0.0462) | **0.0438** (0.0421-0.0456) | **0.0432** (0.0414-0.0450) | **0.0436** (0.0418-0.0454) | **0.0437** (0.0419-0.0455) | **0.0429** (0.0412-0.0448) | **0.0442** (0.0425-0.0461) |
| Stouffer | **0.0434** (0.0416-0.0452) | **0.0464** (0.0446-0.0483) | **0.0471** (0.0453-0.0490) | **0.0474** (0.0456-0.0493) | **0.0479** (0.0460-0.0498) | **0.0489** (0.0470-0.0508) | **0.0482** (0.0464-0.0501) | **0.0490** (0.0471-0.0509) | **0.0504** (0.0486-0.0524) | **0.0499** (0.0480-0.0518) | **0.0492** (0.0473-0.0511) | **0.0490** (0.0471-0.0509) | **0.0493** (0.0474-0.0512) | **0.0489** (0.0470-0.0508) | **0.0489** (0.0471-0.0509) |
| Wilkinson | **0.0105** (0.0096-0.0114) | **0.0111** (0.0102-0.0121) | **0.0141** (0.0131-0.0152) | **0.0154** (0.0144-0.0165) | **0.0168** (0.0157-0.0180) | **0.0177** (0.0166-0.0189) | **0.0194** (0.0182-0.0206) | **0.0202** (0.0190-0.0215) | **0.0203** (0.0191-0.0216) | **0.0220** (0.0207-0.0233) | **0.0218** (0.0206-0.0231) | **0.0230** (0.0217-0.0244) | **0.0234** (0.0221-0.0247) | **0.0230** (0.0218-0.0244) | **0.0234** (0.0221-0.0247) |
| Winer | **0.0429** (0.0412-0.0447) | **0.0460** (0.0442-0.0479) | **0.0483** (0.0465-0.0502) | **0.0469** (0.0451-0.0488) | **0.0478** (0.0459-0.0497) | **0.0484** (0.0465-0.0503) | **0.0479** (0.0461-0.0498) | **0.0485** (0.0466-0.0504) | **0.0494** (0.0476-0.0514) | **0.0501** (0.0482-0.0521) | **0.0490** (0.0471-0.0509) | **0.0478** (0.0459-0.0497) | **0.0478** (0.0460-0.0497) | **0.0488** (0.0469-0.0507) | **0.0489** (0.0470-0.0508) |
| Edgington | **0.0469** (0.0451-0.0488) | **0.0508** (0.0489-0.0528) | **0.0496** (0.0478-0.0516) | **0.0495** (0.0477-0.0515) | **0.0516** (0.0497-0.0536) | **0.0513** (0.0494-0.0533) | **0.0509** (0.0490-0.0528) | **0.0508** (0.0489-0.0527) | **0.0511** (0.0492-0.0531) | **0.0506** (0.0487-0.0525) | **0.0515** (0.0496-0.0535) | **0.0501** (0.0482-0.0520) | **0.0515** (0.0496-0.0534) | **0.0519** (0.0500-0.0538) | **0.0513** (0.0494-0.0532) |
| Mudholkar-George | **0.0414** (0.0397-0.0432) | **0.0449** (0.0432-0.0468) | **0.0460** (0.0442-0.0479) | **0.0464** (0.0446-0.0483) | **0.0460** (0.0442-0.0479) | **0.0474** (0.0456-0.0493) | **0.0471** (0.0452-0.0490) | **0.0478** (0.0459-0.0497) | **0.0492** (0.0473-0.0511) | **0.0494** (0.0475-0.0513) | **0.0484** (0.0466-0.0504) | **0.0471** (0.0452-0.0490) | **0.0477** (0.0458-0.0496) | **0.0472** (0.0454-0.0491) | **0.0475** (0.0457-0.0494) |
| Friston | **0.0466** (0.0448-0.0485) | **0.0540** (0.0521-0.0560) | **0.0508** (0.0489-0.0527) | **0.0493** (0.0474-0.0512) | **0.0477** (0.0459-0.0496) | **0.0476** (0.0458-0.0495) | **0.0471** (0.0452-0.0490) | **0.0461** (0.0443-0.0480) | **0.0456** (0.0438-0.0475) | **0.0452** (0.0435-0.0471) | **0.0454** (0.0436-0.0473) | **0.0448** (0.0430-0.0466) | **0.0445** (0.0427-0.0463) | **0.0440** (0.0423-0.0459) | **0.0434** (0.0416-0.0452) |
| Darlington-Hayes | **0.0320** (0.0305-0.0336) | **0.0406** (0.0389-0.0423) | **0.0384** (0.0367-0.0401) | **0.0414** (0.0397-0.0432) | **0.0395** (0.0378-0.0412) | **0.0413** (0.0396-0.0431) | **0.0393** (0.0376-0.0410) | **0.0422** (0.0405-0.0440) | **0.0413** (0.0396-0.0431) | **0.0435** (0.0417-0.0453) | **0.0413** (0.0396-0.0431) | **0.0421** (0.0404-0.0439) | **0.0420** (0.0403-0.0438) | **0.0422** (0.0405-0.0440) | **0.0420** (0.0403-0.0438) |
| Zaykin | **0.0393** (0.0376-0.0410) | **0.0410** (0.0393-0.0428) | **0.0415** (0.0398-0.0433) | **0.0422** (0.0405-0.0440) | **0.0423** (0.0406-0.0441) | **0.0424** (0.0406-0.0442) | **0.0419** (0.0402-0.0437) | **0.0417** (0.0400-0.0435) | **0.0423** (0.0406-0.0441) | **0.0421** (0.0403-0.0439) | **0.0418** (0.0401-0.0436) | **0.0419** (0.0402-0.0437) | **0.0412** (0.0395-0.0429) | **0.0419** (0.0402-0.0437) | **0.0416** (0.0399-0.0434) |
| Dudbridge-Koeleman | **0.0322** (0.0307-0.0338) | **0.0377** (0.0361-0.0394) | **0.0365** (0.0349-0.0382) | **0.0373** (0.0357-0.0390) | **0.0375** (0.0359-0.0392) | **0.0388** (0.0371-0.0405) | **0.0382** (0.0366-0.0400) | **0.0402** (0.0385-0.0419) | **0.0396** (0.0380-0.0414) | **0.0402** (0.0385-0.0420) | **0.0393** (0.0376-0.0410) | **0.0396** (0.0379-0.0413) | **0.0396** (0.0379-0.0414) | **0.0389** (0.0372-0.0406) | **0.0386** (0.0369-0.0403) |
| Dudbridge-Koeleman2 | **0.0310** (0.0295-0.0325) | **0.0410** (0.0393-0.0428) | **0.0389** (0.0372-0.0406) | **0.0405** (0.0388-0.0422) | **0.0407** (0.0390-0.0425) | **0.0423** (0.0406-0.0441) | **0.0410** (0.0393-0.0428) | **0.0420** (0.0403-0.0438) | **0.0413** (0.0396-0.0431) | **0.0423** (0.0406-0.0441) | **0.0416** (0.0399-0.0434) | **0.0415** (0.0398-0.0433) | **0.0419** (0.0402-0.0437) | **0.0417** (0.0400-0.0435) | **0.0415** (0.0398-0.0433) |
| Taylor-Tibshirani | **0.0458** (0.0440-0.0476) | **0.0484** (0.0466-0.0504) | **0.0480** (0.0462-0.0499) | **0.0472** (0.0453-0.0491) | **0.0487** (0.0469-0.0506) | **0.0477** (0.0458-0.0496) | **0.0475** (0.0457-0.0494) | **0.0485** (0.0466-0.0504) | **0.0483** (0.0465-0.0502) | **0.0498** (0.0479-0.0517) | **0.0479** (0.0460-0.0498) | **0.0473** (0.0455-0.0492) | **0.0479** (0.0461-0.0498) | **0.0487** (0.0468-0.0506) | **0.0477** (0.0458-0.0496) |
| Jiang | **0.0392** (0.0375-0.0409) | **0.0423** (0.0406-0.0441) | **0.0425** (0.0408-0.0443) | **0.0436** (0.0418-0.0454) | **0.0430** (0.0412-0.0448) | **0.0443** (0.0425-0.0461) | **0.0436** (0.0419-0.0454) | **0.0441** (0.0423-0.0459) | **0.0433** (0.0416-0.0451) | **0.0440** (0.0422-0.0458) | **0.0433** (0.0415-0.0451) | **0.0430** (0.0413-0.0448) | **0.0424** (0.0406-0.0442) | **0.0423** (0.0406-0.0441) | **0.0425** (0.0408-0.0443) |
| Hotelling | **0.0239** (0.0226-0.0252) | **0.0259** (0.0246-0.0273) | **0.0279** (0.0265-0.0294) | **0.0300** (0.0286-0.0316) | **0.0314** (0.0299-0.0330) | **0.0318** (0.0303-0.0334) | **0.0333** (0.0318-0.0349) | **0.0329** (0.0314-0.0345) | **0.0326** (0.0311-0.0342) | **0.0338** (0.0322-0.0354) | **0.0337** (0.0322-0.0353) | **0.0348** (0.0332-0.0364) | **0.0354** (0.0338-0.0371) | **0.0354** (0.0338-0.0370) | **0.0365** (0.0349-0.0382) |
| Wilks | **0.0235** (0.0222-0.0249) | **0.0263** (0.0249-0.0277) | **0.0282** (0.0268-0.0297) | **0.0299** (0.0284-0.0314) | **0.0311** (0.0296-0.0327) | **0.0326** (0.0311-0.0342) | **0.0330** (0.0315-0.0346) | **0.0332** (0.0317-0.0348) | **0.0328** (0.0313-0.0344) | **0.0332** (0.0317-0.0348) | **0.0331** (0.0316-0.0347) | **0.0349** (0.0333-0.0365) | **0.0354** (0.0338-0.0371) | **0.0353** (0.0338-0.0370) | **0.0369** (0.0353-0.0386) |
