# Supplementary material for: Non‐parametric combination and related permutation tests for neuroimaging
Source: Hum Brain Mapp. 2016 Feb 5;37(4):1486–511. doi: 10.1002/hbm.23115 (PMC4783210; doi:10.1002/hbm.23115)
Supplement: Supplementary file 1 — Supporting Information [file HBM-37-1486-s001.zip › supporting_information/errorrates/histograms/simB_Gauss_ee+ise_uncp.html]

Sim B, Gauss, EE+ISE, uncp

# Simulations B, Gauss, EE+ISE, uncp

---

| Method | ksig=1 / K=2 | ksig=1 / K=3 | ksig=1 / K=4 | ksig=1 / K=5 | ksig=1 / K=6 | ksig=1 / K=7 | ksig=1 / K=8 | ksig=1 / K=9 | ksig=1 / K=10 | ksig=1 / K=11 | ksig=1 / K=12 | ksig=1 / K=13 | ksig=1 / K=14 | ksig=1 / K=15 | ksig=1 / K=16 |
| --- | --- | --- | --- | --- | --- | --- | --- | --- | --- | --- | --- | --- | --- | --- | --- |
| Tippett | **0.3735** (0.3693-0.3778) | **0.3167** (0.3127-0.3208) | **0.2813** (0.2773-0.2852) | **0.2558** (0.2520-0.2597) | **0.2369** (0.2332-0.2407) | **0.2222** (0.2186-0.2259) | **0.2084** (0.2048-0.2119) | **0.1984** (0.1950-0.2020) | **0.1898** (0.1864-0.1933) | **0.1836** (0.1803-0.1870) | **0.1782** (0.1749-0.1816) | **0.1725** (0.1692-0.1758) | **0.1666** (0.1634-0.1699) | **0.1625** (0.1593-0.1658) | **0.1587** (0.1555-0.1619) |
| Fisher | **0.3609** (0.3567-0.3651) | **0.2989** (0.2949-0.3029) | **0.2588** (0.2549-0.2626) | **0.2311** (0.2274-0.2348) | **0.2106** (0.2070-0.2142) | **0.1966** (0.1931-0.2001) | **0.1826** (0.1793-0.1861) | **0.1719** (0.1686-0.1752) | **0.1643** (0.1611-0.1676) | **0.1575** (0.1543-0.1607) | **0.1527** (0.1495-0.1558) | **0.1473** (0.1442-0.1504) | **0.1410** (0.1379-0.1440) | **0.1376** (0.1346-0.1406) | **0.1352** (0.1322-0.1382) |
| Stouffer | **0.3004** (0.2964-0.3044) | **0.2298** (0.2262-0.2335) | **0.1919** (0.1885-0.1954) | **0.1693** (0.1660-0.1726) | **0.1556** (0.1524-0.1588) | **0.1454** (0.1424-0.1486) | **0.1385** (0.1355-0.1415) | **0.1300** (0.1271-0.1330) | **0.1246** (0.1217-0.1275) | **0.1200** (0.1172-0.1229) | **0.1159** (0.1131-0.1187) | **0.1123** (0.1096-0.1151) | **0.1071** (0.1044-0.1098) | **0.1061** (0.1035-0.1089) | **0.1025** (0.0999-0.1052) |
| Wilkinson | **0.0760** (0.0737-0.0784) | **0.0318** (0.0303-0.0334) | **0.0713** (0.0691-0.0736) | **0.0527** (0.0507-0.0547) | **0.0473** (0.0455-0.0492) | **0.0651** (0.0630-0.0673) | **0.0547** (0.0527-0.0567) | **0.0476** (0.0457-0.0495) | **0.0594** (0.0573-0.0615) | **0.0568** (0.0548-0.0589) | **0.0484** (0.0465-0.0503) | **0.0528** (0.0509-0.0548) | **0.0556** (0.0536-0.0576) | **0.0509** (0.0490-0.0529) | **0.0498** (0.0479-0.0517) |
| Winer | **0.3141** (0.3100-0.3182) | **0.2449** (0.2411-0.2487) | **0.2039** (0.2004-0.2075) | **0.1802** (0.1769-0.1836) | **0.1653** (0.1620-0.1685) | **0.1534** (0.1503-0.1566) | **0.1452** (0.1421-0.1483) | **0.1365** (0.1335-0.1395) | **0.1298** (0.1269-0.1328) | **0.1250** (0.1222-0.1280) | **0.1212** (0.1184-0.1241) | **0.1175** (0.1147-0.1203) | **0.1130** (0.1102-0.1158) | **0.1107** (0.1080-0.1135) | **0.1077** (0.1050-0.1105) |
| Edgington | **0.2019** (0.1984-0.2055) | **0.1532** (0.1500-0.1564) | **0.1363** (0.1333-0.1393) | **0.1221** (0.1192-0.1250) | **0.1173** (0.1145-0.1201) | **0.1103** (0.1075-0.1130) | **0.1058** (0.1032-0.1086) | **0.1008** (0.0982-0.1034) | **0.0973** (0.0947-0.0999) | **0.0963** (0.0937-0.0989) | **0.0927** (0.0902-0.0953) | **0.0894** (0.0869-0.0919) | **0.0897** (0.0872-0.0923) | **0.0864** (0.0839-0.0889) | **0.0848** (0.0824-0.0873) |
| Mudholkar-George | **0.3282** (0.3241-0.3323) | **0.2606** (0.2567-0.2644) | **0.2181** (0.2145-0.2218) | **0.1922** (0.1888-0.1957) | **0.1761** (0.1728-0.1794) | **0.1633** (0.1601-0.1666) | **0.1530** (0.1499-0.1562) | **0.1443** (0.1412-0.1474) | **0.1379** (0.1349-0.1409) | **0.1339** (0.1310-0.1370) | **0.1272** (0.1243-0.1301) | **0.1238** (0.1209-0.1267) | **0.1186** (0.1158-0.1214) | **0.1158** (0.1130-0.1186) | **0.1130** (0.1102-0.1158) |
| Friston | **0.1683** (0.1650-0.1716) | **0.1149** (0.1122-0.1178) | **0.0924** (0.0899-0.0950) | **0.0818** (0.0794-0.0842) | **0.0762** (0.0739-0.0786) | **0.0724** (0.0701-0.0747) | **0.0691** (0.0669-0.0713) | **0.0662** (0.0640-0.0684) | **0.0634** (0.0613-0.0656) | **0.0618** (0.0597-0.0639) | **0.0605** (0.0585-0.0627) | **0.0588** (0.0568-0.0609) | **0.0568** (0.0548-0.0589) | **0.0569** (0.0549-0.0590) | **0.0567** (0.0547-0.0588) |
| Darlington-Hayes | **0.3724** (0.3682-0.3767) | **0.2758** (0.2719-0.2797) | **0.2522** (0.2485-0.2561) | **0.2118** (0.2082-0.2154) | **0.2024** (0.1989-0.2059) | **0.1795** (0.1762-0.1829) | **0.1735** (0.1702-0.1769) | **0.1596** (0.1565-0.1629) | **0.1561** (0.1529-0.1593) | **0.1446** (0.1415-0.1477) | **0.1437** (0.1407-0.1468) | **0.1354** (0.1324-0.1384) | **0.1337** (0.1307-0.1367) | **0.1274** (0.1245-0.1304) | **0.1259** (0.1230-0.1288) |
| Zaykin | **0.3511** (0.3470-0.3553) | **0.2857** (0.2817-0.2896) | **0.2470** (0.2432-0.2508) | **0.2193** (0.2157-0.2229) | **0.2014** (0.1979-0.2049) | **0.1867** (0.1833-0.1902) | **0.1748** (0.1715-0.1782) | **0.1649** (0.1617-0.1682) | **0.1556** (0.1524-0.1588) | **0.1500** (0.1469-0.1532) | **0.1456** (0.1426-0.1487) | **0.1406** (0.1376-0.1437) | **0.1355** (0.1326-0.1386) | **0.1306** (0.1277-0.1336) | **0.1263** (0.1235-0.1293) |
| Dudbridge-Koeleman | **0.3723** (0.3681-0.3765) | **0.3076** (0.3036-0.3117) | **0.2757** (0.2718-0.2797) | **0.2420** (0.2383-0.2458) | **0.2256** (0.2219-0.2293) | **0.2071** (0.2036-0.2107) | **0.1963** (0.1929-0.1998) | **0.1828** (0.1794-0.1862) | **0.1755** (0.1722-0.1789) | **0.1664** (0.1632-0.1697) | **0.1616** (0.1584-0.1648) | **0.1561** (0.1530-0.1593) | **0.1517** (0.1486-0.1549) | **0.1447** (0.1417-0.1478) | **0.1419** (0.1389-0.1450) |
| Dudbridge-Koeleman2 | **0.3724** (0.3682-0.3766) | **0.2917** (0.2878-0.2957) | **0.2599** (0.2561-0.2638) | **0.2217** (0.2181-0.2254) | **0.2089** (0.2053-0.2125) | **0.1871** (0.1837-0.1905) | **0.1762** (0.1729-0.1796) | **0.1662** (0.1630-0.1695) | **0.1576** (0.1545-0.1609) | **0.1499** (0.1468-0.1531) | **0.1454** (0.1424-0.1485) | **0.1404** (0.1374-0.1435) | **0.1352** (0.1322-0.1382) | **0.1313** (0.1283-0.1343) | **0.1276** (0.1247-0.1305) |
| Taylor-Tibshirani | **0.2264** (0.2227-0.2301) | **0.1849** (0.1815-0.1883) | **0.1660** (0.1628-0.1693) | **0.1511** (0.1480-0.1543) | **0.1422** (0.1392-0.1453) | **0.1354** (0.1325-0.1385) | **0.1298** (0.1269-0.1328) | **0.1236** (0.1207-0.1265) | **0.1203** (0.1175-0.1232) | **0.1178** (0.1150-0.1206) | **0.1135** (0.1108-0.1164) | **0.1104** (0.1076-0.1131) | **0.1074** (0.1048-0.1102) | **0.1057** (0.1030-0.1084) | **0.1047** (0.1021-0.1075) |
| Jiang | **0.2899** (0.2859-0.2939) | **0.2052** (0.2017-0.2088) | **0.1836** (0.1802-0.1870) | **0.1615** (0.1583-0.1648) | **0.1522** (0.1490-0.1553) | **0.1444** (0.1413-0.1475) | **0.1399** (0.1369-0.1430) | **0.1331** (0.1302-0.1361) | **0.1289** (0.1260-0.1318) | **0.1259** (0.1230-0.1288) | **0.1220** (0.1192-0.1249) | **0.1204** (0.1176-0.1233) | **0.1158** (0.1130-0.1186) | **0.1140** (0.1112-0.1168) | **0.1116** (0.1089-0.1144) |
| Hotelling | **0.2609** (0.2570-0.2647) | **0.2138** (0.2102-0.2174) | **0.1809** (0.1775-0.1843) | **0.1578** (0.1546-0.1610) | **0.1413** (0.1383-0.1444) | **0.1252** (0.1223-0.1281) | **0.1161** (0.1133-0.1190) | **0.1061** (0.1034-0.1088) | **0.0962** (0.0936-0.0988) | **0.0911** (0.0886-0.0937) | **0.0848** (0.0824-0.0872) | **0.0783** (0.0760-0.0807) | **0.0735** (0.0712-0.0758) | **0.0670** (0.0648-0.0692) | **0.0618** (0.0597-0.0639) |
| Wilks | **0.2591** (0.2553-0.2630) | **0.2106** (0.2071-0.2142) | **0.1782** (0.1749-0.1816) | **0.1561** (0.1529-0.1593) | **0.1376** (0.1346-0.1407) | **0.1248** (0.1219-0.1277) | **0.1125** (0.1098-0.1153) | **0.1031** (0.1005-0.1058) | **0.0949** (0.0924-0.0975) | **0.0879** (0.0855-0.0904) | **0.0807** (0.0783-0.0831) | **0.0760** (0.0737-0.0784) | **0.0709** (0.0687-0.0732) | **0.0682** (0.0660-0.0704) | **0.0629** (0.0608-0.0650) |
