# Supplementary material for: Non‐parametric combination and related permutation tests for neuroimaging
Source: Hum Brain Mapp. 2016 Feb 5;37(4):1486–511. doi: 10.1002/hbm.23115 (PMC4783210; doi:10.1002/hbm.23115)
Supplement: Supplementary file 1 — Supporting Information [file HBM-37-1486-s001.zip › supporting_information/errorrates/histograms/simB_Gauss_ee_uncp.html]

Sim B, Gauss, EE, uncp

# Simulations B, Gauss, EE, uncp

---

| Method | ksig=1 / K=2 | ksig=1 / K=3 | ksig=1 / K=4 | ksig=1 / K=5 | ksig=1 / K=6 | ksig=1 / K=7 | ksig=1 / K=8 | ksig=1 / K=9 | ksig=1 / K=10 | ksig=1 / K=11 | ksig=1 / K=12 | ksig=1 / K=13 | ksig=1 / K=14 | ksig=1 / K=15 | ksig=1 / K=16 |
| --- | --- | --- | --- | --- | --- | --- | --- | --- | --- | --- | --- | --- | --- | --- | --- |
| Tippett | **0.3674** (0.3632-0.3716) | **0.3121** (0.3081-0.3162) | **0.2792** (0.2752-0.2831) | **0.2541** (0.2503-0.2579) | **0.2360** (0.2323-0.2398) | **0.2213** (0.2177-0.2250) | **0.2084** (0.2048-0.2119) | **0.1999** (0.1965-0.2035) | **0.1897** (0.1863-0.1932) | **0.1831** (0.1798-0.1866) | **0.1773** (0.1740-0.1807) | **0.1710** (0.1677-0.1743) | **0.1656** (0.1624-0.1689) | **0.1615** (0.1583-0.1648) | **0.1581** (0.1549-0.1613) |
| Fisher | **0.3559** (0.3517-0.3601) | **0.2940** (0.2900-0.2980) | **0.2556** (0.2518-0.2594) | **0.2291** (0.2254-0.2328) | **0.2132** (0.2096-0.2168) | **0.1958** (0.1923-0.1993) | **0.1831** (0.1797-0.1865) | **0.1732** (0.1699-0.1765) | **0.1666** (0.1634-0.1699) | **0.1580** (0.1548-0.1612) | **0.1519** (0.1487-0.1550) | **0.1472** (0.1441-0.1503) | **0.1426** (0.1396-0.1457) | **0.1390** (0.1360-0.1420) | **0.1358** (0.1328-0.1388) |
| Stouffer | **0.2966** (0.2926-0.3006) | **0.2280** (0.2243-0.2317) | **0.1913** (0.1879-0.1948) | **0.1707** (0.1674-0.1740) | **0.1548** (0.1517-0.1580) | **0.1441** (0.1410-0.1472) | **0.1374** (0.1344-0.1404) | **0.1294** (0.1265-0.1324) | **0.1239** (0.1211-0.1268) | **0.1189** (0.1161-0.1217) | **0.1160** (0.1132-0.1188) | **0.1121** (0.1094-0.1149) | **0.1086** (0.1059-0.1113) | **0.1069** (0.1043-0.1097) | **0.1037** (0.1011-0.1064) |
| Wilkinson | **0.0758** (0.0735-0.0782) | **0.0312** (0.0297-0.0328) | **0.0701** (0.0679-0.0724) | **0.0532** (0.0513-0.0552) | **0.0467** (0.0449-0.0486) | **0.0660** (0.0638-0.0682) | **0.0560** (0.0540-0.0580) | **0.0484** (0.0465-0.0503) | **0.0604** (0.0583-0.0625) | **0.0581** (0.0561-0.0602) | **0.0489** (0.0470-0.0508) | **0.0556** (0.0537-0.0577) | **0.0586** (0.0565-0.0607) | **0.0523** (0.0503-0.0542) | **0.0523** (0.0503-0.0542) |
| Winer | **0.3099** (0.3059-0.3140) | **0.2421** (0.2384-0.2459) | **0.2039** (0.2004-0.2075) | **0.1805** (0.1772-0.1839) | **0.1644** (0.1612-0.1677) | **0.1516** (0.1484-0.1547) | **0.1441** (0.1411-0.1472) | **0.1363** (0.1334-0.1394) | **0.1289** (0.1260-0.1318) | **0.1252** (0.1223-0.1281) | **0.1213** (0.1184-0.1242) | **0.1174** (0.1146-0.1203) | **0.1150** (0.1122-0.1178) | **0.1113** (0.1086-0.1141) | **0.1081** (0.1054-0.1109) |
| Edgington | **0.2018** (0.1983-0.2053) | **0.1531** (0.1500-0.1563) | **0.1334** (0.1304-0.1364) | **0.1228** (0.1200-0.1257) | **0.1155** (0.1127-0.1183) | **0.1084** (0.1057-0.1111) | **0.1039** (0.1012-0.1066) | **0.0992** (0.0966-0.1019) | **0.0958** (0.0933-0.0984) | **0.0953** (0.0927-0.0979) | **0.0926** (0.0901-0.0952) | **0.0920** (0.0895-0.0945) | **0.0898** (0.0873-0.0923) | **0.0883** (0.0859-0.0908) | **0.0870** (0.0846-0.0895) |
| Mudholkar-George | **0.3251** (0.3210-0.3292) | **0.2570** (0.2532-0.2608) | **0.2187** (0.2151-0.2224) | **0.1933** (0.1899-0.1968) | **0.1750** (0.1717-0.1784) | **0.1625** (0.1593-0.1657) | **0.1526** (0.1495-0.1558) | **0.1444** (0.1413-0.1475) | **0.1391** (0.1361-0.1421) | **0.1307** (0.1278-0.1337) | **0.1274** (0.1245-0.1303) | **0.1240** (0.1211-0.1269) | **0.1207** (0.1179-0.1236) | **0.1160** (0.1133-0.1189) | **0.1133** (0.1106-0.1161) |
| Friston | **0.1684** (0.1651-0.1717) | **0.1128** (0.1101-0.1156) | **0.0934** (0.0909-0.0960) | **0.0821** (0.0797-0.0846) | **0.0756** (0.0734-0.0780) | **0.0712** (0.0690-0.0735) | **0.0674** (0.0652-0.0696) | **0.0636** (0.0615-0.0658) | **0.0618** (0.0597-0.0639) | **0.0615** (0.0595-0.0637) | **0.0610** (0.0589-0.0631) | **0.0602** (0.0582-0.0623) | **0.0604** (0.0584-0.0626) | **0.0601** (0.0580-0.0622) | **0.0596** (0.0575-0.0617) |
| Darlington-Hayes | **0.3669** (0.3627-0.3712) | **0.2719** (0.2680-0.2758) | **0.2511** (0.2474-0.2550) | **0.2126** (0.2091-0.2162) | **0.2024** (0.1989-0.2059) | **0.1800** (0.1766-0.1834) | **0.1728** (0.1695-0.1762) | **0.1576** (0.1545-0.1608) | **0.1543** (0.1512-0.1575) | **0.1431** (0.1401-0.1462) | **0.1427** (0.1397-0.1458) | **0.1357** (0.1327-0.1387) | **0.1338** (0.1308-0.1368) | **0.1267** (0.1238-0.1296) | **0.1272** (0.1243-0.1302) |
| Zaykin | **0.3463** (0.3421-0.3505) | **0.2857** (0.2818-0.2897) | **0.2455** (0.2418-0.2493) | **0.2200** (0.2164-0.2237) | **0.2008** (0.1973-0.2043) | **0.1868** (0.1834-0.1903) | **0.1763** (0.1730-0.1797) | **0.1638** (0.1606-0.1671) | **0.1572** (0.1541-0.1604) | **0.1509** (0.1478-0.1541) | **0.1456** (0.1425-0.1487) | **0.1411** (0.1381-0.1442) | **0.1372** (0.1342-0.1402) | **0.1330** (0.1301-0.1360) | **0.1298** (0.1268-0.1327) |
| Dudbridge-Koeleman | **0.3675** (0.3633-0.3717) | **0.3040** (0.3000-0.3080) | **0.2720** (0.2681-0.2759) | **0.2420** (0.2382-0.2458) | **0.2254** (0.2217-0.2290) | **0.2073** (0.2038-0.2109) | **0.1956** (0.1921-0.1991) | **0.1821** (0.1788-0.1855) | **0.1760** (0.1727-0.1794) | **0.1650** (0.1618-0.1683) | **0.1615** (0.1583-0.1647) | **0.1550** (0.1518-0.1582) | **0.1524** (0.1493-0.1556) | **0.1470** (0.1439-0.1502) | **0.1436** (0.1405-0.1467) |
| Dudbridge-Koeleman2 | **0.3652** (0.3610-0.3694) | **0.2898** (0.2859-0.2938) | **0.2581** (0.2542-0.2619) | **0.2224** (0.2188-0.2261) | **0.2072** (0.2037-0.2108) | **0.1903** (0.1869-0.1938) | **0.1777** (0.1744-0.1811) | **0.1662** (0.1629-0.1694) | **0.1581** (0.1549-0.1613) | **0.1497** (0.1466-0.1528) | **0.1464** (0.1433-0.1495) | **0.1418** (0.1387-0.1448) | **0.1379** (0.1349-0.1410) | **0.1321** (0.1292-0.1351) | **0.1286** (0.1257-0.1316) |
| Taylor-Tibshirani | **0.2241** (0.2204-0.2278) | **0.1847** (0.1813-0.1881) | **0.1635** (0.1602-0.1667) | **0.1501** (0.1470-0.1532) | **0.1413** (0.1383-0.1444) | **0.1341** (0.1311-0.1371) | **0.1279** (0.1250-0.1308) | **0.1235** (0.1207-0.1264) | **0.1190** (0.1162-0.1219) | **0.1168** (0.1140-0.1196) | **0.1143** (0.1115-0.1171) | **0.1113** (0.1086-0.1141) | **0.1097** (0.1070-0.1125) | **0.1062** (0.1035-0.1089) | **0.1043** (0.1016-0.1070) |
| Jiang | **0.2875** (0.2836-0.2915) | **0.2030** (0.1995-0.2066) | **0.1860** (0.1826-0.1895) | **0.1624** (0.1592-0.1657) | **0.1530** (0.1499-0.1562) | **0.1454** (0.1423-0.1485) | **0.1402** (0.1372-0.1433) | **0.1346** (0.1317-0.1377) | **0.1298** (0.1269-0.1328) | **0.1262** (0.1233-0.1291) | **0.1242** (0.1214-0.1272) | **0.1208** (0.1180-0.1237) | **0.1192** (0.1164-0.1221) | **0.1158** (0.1130-0.1186) | **0.1133** (0.1106-0.1161) |
| Hotelling | **0.2587** (0.2549-0.2626) | **0.2107** (0.2072-0.2143) | **0.1787** (0.1753-0.1821) | **0.1596** (0.1564-0.1628) | **0.1411** (0.1380-0.1441) | **0.1257** (0.1228-0.1286) | **0.1132** (0.1105-0.1160) | **0.1034** (0.1008-0.1061) | **0.0955** (0.0930-0.0981) | **0.0898** (0.0873-0.0923) | **0.0841** (0.0817-0.0866) | **0.0771** (0.0748-0.0795) | **0.0722** (0.0700-0.0745) | **0.0665** (0.0644-0.0687) | **0.0628** (0.0607-0.0649) |
| Wilks | **0.2598** (0.2560-0.2636) | **0.2122** (0.2086-0.2158) | **0.1828** (0.1794-0.1862) | **0.1571** (0.1539-0.1603) | **0.1409** (0.1379-0.1440) | **0.1276** (0.1247-0.1306) | **0.1142** (0.1114-0.1170) | **0.1044** (0.1018-0.1072) | **0.0960** (0.0935-0.0986) | **0.0891** (0.0866-0.0916) | **0.0835** (0.0811-0.0860) | **0.0755** (0.0732-0.0779) | **0.0722** (0.0699-0.0745) | **0.0662** (0.0640-0.0684) | **0.0605** (0.0585-0.0627) |
