# Supplementary material for: Non‐parametric combination and related permutation tests for neuroimaging
Source: Hum Brain Mapp. 2016 Feb 5;37(4):1486–511. doi: 10.1002/hbm.23115 (PMC4783210; doi:10.1002/hbm.23115)
Supplement: Supplementary file 1 — Supporting Information [file HBM-37-1486-s001.zip › supporting_information/errorrates/histograms/simB_Gauss_ise_uncp.html]

Sim B, Gauss, ISE, uncp

# Simulations B, Gauss, ISE, uncp

---

| Method | ksig=1 / K=2 | ksig=1 / K=3 | ksig=1 / K=4 | ksig=1 / K=5 | ksig=1 / K=6 | ksig=1 / K=7 | ksig=1 / K=8 | ksig=1 / K=9 | ksig=1 / K=10 | ksig=1 / K=11 | ksig=1 / K=12 | ksig=1 / K=13 | ksig=1 / K=14 | ksig=1 / K=15 | ksig=1 / K=16 |
| --- | --- | --- | --- | --- | --- | --- | --- | --- | --- | --- | --- | --- | --- | --- | --- |
| Tippett | **0.3475** (0.3433-0.3517) | **0.2986** (0.2946-0.3026) | **0.2670** (0.2631-0.2709) | **0.2460** (0.2422-0.2498) | **0.2278** (0.2241-0.2315) | **0.2152** (0.2116-0.2188) | **0.2049** (0.2014-0.2085) | **0.1960** (0.1925-0.1995) | **0.1886** (0.1852-0.1920) | **0.1813** (0.1780-0.1847) | **0.1759** (0.1725-0.1792) | **0.1702** (0.1669-0.1735) | **0.1660** (0.1628-0.1693) | **0.1621** (0.1589-0.1654) | **0.1588** (0.1557-0.1621) |
| Fisher | **0.3358** (0.3317-0.3400) | **0.2815** (0.2776-0.2855) | **0.2452** (0.2415-0.2490) | **0.2203** (0.2167-0.2240) | **0.2013** (0.1978-0.2049) | **0.1879** (0.1845-0.1913) | **0.1761** (0.1728-0.1794) | **0.1670** (0.1638-0.1703) | **0.1591** (0.1559-0.1623) | **0.1523** (0.1491-0.1554) | **0.1466** (0.1435-0.1497) | **0.1420** (0.1389-0.1450) | **0.1371** (0.1342-0.1402) | **0.1310** (0.1281-0.1340) | **0.1305** (0.1276-0.1335) |
| Stouffer | **0.2763** (0.2724-0.2802) | **0.2139** (0.2103-0.2175) | **0.1803** (0.1769-0.1837) | **0.1606** (0.1574-0.1639) | **0.1459** (0.1429-0.1491) | **0.1361** (0.1331-0.1391) | **0.1270** (0.1241-0.1299) | **0.1224** (0.1195-0.1253) | **0.1169** (0.1142-0.1198) | **0.1118** (0.1090-0.1146) | **0.1095** (0.1068-0.1123) | **0.1063** (0.1036-0.1091) | **0.1035** (0.1009-0.1062) | **0.1007** (0.0981-0.1034) | **0.0989** (0.0963-0.1015) |
| Wilkinson | **0.0425** (0.0408-0.0443) | **0.0301** (0.0286-0.0316) | **0.0443** (0.0425-0.0461) | **0.0450** (0.0432-0.0468) | **0.0387** (0.0371-0.0404) | **0.0455** (0.0437-0.0474) | **0.0467** (0.0449-0.0486) | **0.0423** (0.0405-0.0441) | **0.0448** (0.0430-0.0467) | **0.0468** (0.0450-0.0487) | **0.0454** (0.0436-0.0472) | **0.0453** (0.0435-0.0472) | **0.0468** (0.0450-0.0487) | **0.0482** (0.0464-0.0502) | **0.0473** (0.0454-0.0492) |
| Winer | **0.2909** (0.2869-0.2949) | **0.2272** (0.2235-0.2309) | **0.1944** (0.1910-0.1979) | **0.1717** (0.1684-0.1750) | **0.1550** (0.1519-0.1582) | **0.1461** (0.1430-0.1492) | **0.1369** (0.1339-0.1400) | **0.1300** (0.1271-0.1330) | **0.1243** (0.1215-0.1272) | **0.1213** (0.1184-0.1242) | **0.1163** (0.1135-0.1192) | **0.1127** (0.1100-0.1155) | **0.1094** (0.1067-0.1122) | **0.1057** (0.1030-0.1084) | **0.1038** (0.1012-0.1065) |
| Edgington | **0.1802** (0.1769-0.1836) | **0.1377** (0.1347-0.1407) | **0.1201** (0.1173-0.1230) | **0.1104** (0.1077-0.1132) | **0.1039** (0.1012-0.1066) | **0.0993** (0.0967-0.1020) | **0.0966** (0.0940-0.0992) | **0.0942** (0.0917-0.0968) | **0.0915** (0.0890-0.0941) | **0.0890** (0.0865-0.0915) | **0.0865** (0.0840-0.0890) | **0.0863** (0.0839-0.0888) | **0.0841** (0.0817-0.0865) | **0.0816** (0.0793-0.0841) | **0.0794** (0.0771-0.0818) |
| Mudholkar-George | **0.3042** (0.3002-0.3082) | **0.2438** (0.2401-0.2476) | **0.2087** (0.2052-0.2123) | **0.1867** (0.1833-0.1901) | **0.1687** (0.1654-0.1720) | **0.1583** (0.1551-0.1615) | **0.1483** (0.1452-0.1514) | **0.1400** (0.1369-0.1430) | **0.1329** (0.1300-0.1359) | **0.1262** (0.1233-0.1291) | **0.1241** (0.1212-0.1270) | **0.1188** (0.1160-0.1217) | **0.1149** (0.1121-0.1177) | **0.1112** (0.1085-0.1140) | **0.1092** (0.1065-0.1120) |
| Friston | **0.1465** (0.1434-0.1496) | **0.1002** (0.0976-0.1028) | **0.0840** (0.0816-0.0864) | **0.0747** (0.0724-0.0770) | **0.0716** (0.0694-0.0739) | **0.0687** (0.0666-0.0710) | **0.0659** (0.0638-0.0681) | **0.0641** (0.0619-0.0662) | **0.0629** (0.0608-0.0651) | **0.0613** (0.0592-0.0634) | **0.0592** (0.0572-0.0613) | **0.0585** (0.0565-0.0606) | **0.0573** (0.0553-0.0594) | **0.0571** (0.0551-0.0591) | **0.0557** (0.0538-0.0578) |
| Darlington-Hayes | **0.3462** (0.3420-0.3504) | **0.2548** (0.2510-0.2586) | **0.2357** (0.2320-0.2395) | **0.1986** (0.1952-0.2022) | **0.1884** (0.1850-0.1919) | **0.1687** (0.1654-0.1720) | **0.1634** (0.1601-0.1666) | **0.1513** (0.1482-0.1545) | **0.1467** (0.1437-0.1499) | **0.1360** (0.1330-0.1391) | **0.1363** (0.1334-0.1394) | **0.1287** (0.1258-0.1316) | **0.1270** (0.1241-0.1299) | **0.1206** (0.1178-0.1235) | **0.1175** (0.1147-0.1204) |
| Zaykin | **0.3265** (0.3224-0.3307) | **0.2672** (0.2634-0.2711) | **0.2324** (0.2287-0.2361) | **0.2068** (0.2033-0.2104) | **0.1879** (0.1845-0.1913) | **0.1760** (0.1727-0.1794) | **0.1649** (0.1617-0.1682) | **0.1541** (0.1510-0.1573) | **0.1472** (0.1441-0.1503) | **0.1415** (0.1385-0.1446) | **0.1370** (0.1341-0.1401) | **0.1319** (0.1290-0.1349) | **0.1288** (0.1259-0.1317) | **0.1246** (0.1217-0.1275) | **0.1224** (0.1195-0.1253) |
| Dudbridge-Koeleman | **0.3457** (0.3416-0.3499) | **0.2892** (0.2852-0.2931) | **0.2599** (0.2560-0.2637) | **0.2302** (0.2265-0.2339) | **0.2146** (0.2110-0.2182) | **0.1961** (0.1926-0.1996) | **0.1874** (0.1840-0.1908) | **0.1738** (0.1705-0.1771) | **0.1678** (0.1646-0.1711) | **0.1586** (0.1554-0.1618) | **0.1559** (0.1527-0.1591) | **0.1483** (0.1452-0.1515) | **0.1453** (0.1422-0.1484) | **0.1390** (0.1360-0.1420) | **0.1355** (0.1325-0.1385) |
| Dudbridge-Koeleman2 | **0.3473** (0.3431-0.3515) | **0.2724** (0.2685-0.2763) | **0.2441** (0.2403-0.2479) | **0.2121** (0.2085-0.2157) | **0.1957** (0.1922-0.1992) | **0.1763** (0.1730-0.1797) | **0.1685** (0.1653-0.1718) | **0.1555** (0.1523-0.1587) | **0.1500** (0.1469-0.1531) | **0.1422** (0.1391-0.1452) | **0.1377** (0.1347-0.1408) | **0.1316** (0.1286-0.1346) | **0.1286** (0.1257-0.1316) | **0.1244** (0.1216-0.1274) | **0.1222** (0.1193-0.1251) |
| Taylor-Tibshirani | **0.2023** (0.1988-0.2058) | **0.1687** (0.1654-0.1720) | **0.1485** (0.1454-0.1516) | **0.1383** (0.1353-0.1414) | **0.1279** (0.1250-0.1309) | **0.1247** (0.1218-0.1276) | **0.1191** (0.1163-0.1220) | **0.1171** (0.1144-0.1200) | **0.1131** (0.1104-0.1159) | **0.1078** (0.1051-0.1106) | **0.1071** (0.1045-0.1099) | **0.1046** (0.1019-0.1073) | **0.1028** (0.1002-0.1055) | **0.0991** (0.0965-0.1018) | **0.0991** (0.0965-0.1018) |
| Jiang | **0.2729** (0.2691-0.2769) | **0.1815** (0.1781-0.1849) | **0.1636** (0.1604-0.1669) | **0.1489** (0.1458-0.1521) | **0.1366** (0.1336-0.1396) | **0.1326** (0.1297-0.1356) | **0.1303** (0.1273-0.1332) | **0.1244** (0.1215-0.1273) | **0.1212** (0.1184-0.1241) | **0.1161** (0.1133-0.1190) | **0.1150** (0.1123-0.1178) | **0.1128** (0.1101-0.1156) | **0.1086** (0.1059-0.1114) | **0.1071** (0.1044-0.1098) | **0.1055** (0.1029-0.1083) |
| Hotelling | **0.2352** (0.2315-0.2390) | **0.1947** (0.1912-0.1982) | **0.1664** (0.1631-0.1696) | **0.1482** (0.1451-0.1513) | **0.1314** (0.1284-0.1343) | **0.1185** (0.1157-0.1214) | **0.1088** (0.1061-0.1116) | **0.1013** (0.0987-0.1040) | **0.0945** (0.0920-0.0971) | **0.0886** (0.0861-0.0911) | **0.0807** (0.0783-0.0831) | **0.0772** (0.0749-0.0795) | **0.0722** (0.0700-0.0745) | **0.0666** (0.0645-0.0689) | **0.0618** (0.0597-0.0639) |
| Wilks | **0.2332** (0.2296-0.2370) | **0.1948** (0.1914-0.1983) | **0.1678** (0.1646-0.1711) | **0.1489** (0.1458-0.1520) | **0.1323** (0.1293-0.1353) | **0.1174** (0.1146-0.1203) | **0.1094** (0.1067-0.1121) | **0.1027** (0.1001-0.1054) | **0.0924** (0.0899-0.0949) | **0.0872** (0.0848-0.0897) | **0.0816** (0.0793-0.0841) | **0.0765** (0.0742-0.0788) | **0.0709** (0.0687-0.0732) | **0.0664** (0.0642-0.0686) | **0.0610** (0.0589-0.0631) |
