# Supplementary material for: Non‐parametric combination and related permutation tests for neuroimaging
Source: Hum Brain Mapp. 2016 Feb 5;37(4):1486–511. doi: 10.1002/hbm.23115 (PMC4783210; doi:10.1002/hbm.23115)
Supplement: Supplementary file 1 — Supporting Information [file HBM-37-1486-s001.zip › supporting_information/errorrates/histograms/simB_Weibull_ee+ise_uncp.html]

Sim B, Weibull, EE+ISE, uncp

# Simulations B, Weibull, EE+ISE, uncp

---

| Method | ksig=1 / K=2 | ksig=1 / K=3 | ksig=1 / K=4 | ksig=1 / K=5 | ksig=1 / K=6 | ksig=1 / K=7 | ksig=1 / K=8 | ksig=1 / K=9 | ksig=1 / K=10 | ksig=1 / K=11 | ksig=1 / K=12 | ksig=1 / K=13 | ksig=1 / K=14 | ksig=1 / K=15 | ksig=1 / K=16 |
| --- | --- | --- | --- | --- | --- | --- | --- | --- | --- | --- | --- | --- | --- | --- | --- |
| Tippett | **0.6463** (0.6421-0.6505) | **0.6158** (0.6115-0.6200) | **0.5953** (0.5910-0.5996) | **0.5813** (0.5769-0.5856) | **0.5711** (0.5668-0.5754) | **0.5624** (0.5581-0.5668) | **0.5543** (0.5499-0.5587) | **0.5486** (0.5443-0.5530) | **0.5429** (0.5386-0.5473) | **0.5380** (0.5336-0.5424) | **0.5342** (0.5298-0.5386) | **0.5307** (0.5263-0.5351) | **0.5256** (0.5212-0.5299) | **0.5226** (0.5182-0.5270) | **0.5196** (0.5152-0.5240) |
| Fisher | **0.6355** (0.6313-0.6397) | **0.5946** (0.5903-0.5989) | **0.5668** (0.5624-0.5711) | **0.5424** (0.5381-0.5468) | **0.5216** (0.5172-0.5260) | **0.5035** (0.4991-0.5078) | **0.4872** (0.4828-0.4916) | **0.4740** (0.4696-0.4784) | **0.4603** (0.4560-0.4647) | **0.4506** (0.4463-0.4550) | **0.4383** (0.4340-0.4427) | **0.4292** (0.4249-0.4335) | **0.4199** (0.4156-0.4242) | **0.4112** (0.4069-0.4155) | **0.4045** (0.4002-0.4088) |
| Stouffer | **0.5722** (0.5678-0.5765) | **0.4876** (0.4833-0.4920) | **0.4288** (0.4245-0.4332) | **0.3824** (0.3781-0.3866) | **0.3442** (0.3400-0.3483) | **0.3169** (0.3129-0.3210) | **0.2947** (0.2907-0.2987) | **0.2743** (0.2704-0.2782) | **0.2576** (0.2538-0.2614) | **0.2452** (0.2415-0.2490) | **0.2340** (0.2303-0.2377) | **0.2220** (0.2184-0.2257) | **0.2142** (0.2106-0.2178) | **0.2054** (0.2018-0.2089) | **0.1990** (0.1955-0.2025) |
| Wilkinson | **0.0693** (0.0671-0.0715) | **0.0323** (0.0308-0.0339) | **0.0672** (0.0650-0.0694) | **0.0520** (0.0500-0.0539) | **0.0501** (0.0482-0.0520) | **0.0623** (0.0602-0.0644) | **0.0530** (0.0510-0.0550) | **0.0510** (0.0491-0.0530) | **0.0572** (0.0552-0.0593) | **0.0553** (0.0533-0.0573) | **0.0534** (0.0514-0.0554) | **0.0542** (0.0523-0.0562) | **0.0551** (0.0532-0.0572) | **0.0530** (0.0510-0.0550) | **0.0536** (0.0516-0.0556) |
| Winer | **0.5913** (0.5870-0.5956) | **0.5251** (0.5207-0.5295) | **0.4757** (0.4713-0.4801) | **0.4424** (0.4381-0.4468) | **0.4136** (0.4093-0.4179) | **0.3901** (0.3859-0.3944) | **0.3677** (0.3635-0.3719) | **0.3515** (0.3473-0.3557) | **0.3366** (0.3325-0.3408) | **0.3249** (0.3208-0.3291) | **0.3132** (0.3091-0.3172) | **0.3041** (0.3001-0.3082) | **0.2954** (0.2915-0.2995) | **0.2852** (0.2813-0.2892) | **0.2787** (0.2748-0.2827) |
| Edgington | **0.2326** (0.2289-0.2363) | **0.1642** (0.1610-0.1675) | **0.1406** (0.1376-0.1437) | **0.1270** (0.1241-0.1300) | **0.1177** (0.1149-0.1206) | **0.1108** (0.1081-0.1136) | **0.1053** (0.1026-0.1080) | **0.1006** (0.0980-0.1033) | **0.0984** (0.0958-0.1010) | **0.0943** (0.0918-0.0969) | **0.0935** (0.0910-0.0961) | **0.0904** (0.0879-0.0930) | **0.0893** (0.0869-0.0919) | **0.0878** (0.0853-0.0903) | **0.0860** (0.0836-0.0885) |
| Mudholkar-George | **0.6071** (0.6028-0.6113) | **0.5488** (0.5445-0.5532) | **0.5068** (0.5024-0.5111) | **0.4748** (0.4705-0.4792) | **0.4483** (0.4439-0.4526) | **0.4252** (0.4209-0.4295) | **0.4058** (0.4015-0.4101) | **0.3869** (0.3826-0.3912) | **0.3713** (0.3671-0.3756) | **0.3610** (0.3568-0.3652) | **0.3471** (0.3430-0.3513) | **0.3376** (0.3335-0.3418) | **0.3281** (0.3240-0.3322) | **0.3196** (0.3155-0.3237) | **0.3101** (0.3060-0.3141) |
| Friston | **0.1733** (0.1700-0.1766) | **0.1056** (0.1030-0.1084) | **0.0847** (0.0823-0.0872) | **0.0741** (0.0718-0.0764) | **0.0713** (0.0690-0.0735) | **0.0672** (0.0650-0.0694) | **0.0641** (0.0620-0.0663) | **0.0618** (0.0597-0.0639) | **0.0617** (0.0596-0.0638) | **0.0602** (0.0581-0.0623) | **0.0601** (0.0580-0.0622) | **0.0594** (0.0574-0.0615) | **0.0591** (0.0571-0.0612) | **0.0580** (0.0560-0.0601) | **0.0586** (0.0565-0.0607) |
| Darlington-Hayes | **0.6455** (0.6413-0.6496) | **0.5642** (0.5599-0.5686) | **0.5532** (0.5488-0.5575) | **0.4902** (0.4858-0.4946) | **0.4855** (0.4811-0.4898) | **0.4354** (0.4310-0.4397) | **0.4340** (0.4296-0.4383) | **0.3929** (0.3886-0.3972) | **0.3923** (0.3880-0.3966) | **0.3619** (0.3577-0.3661) | **0.3619** (0.3577-0.3661) | **0.3338** (0.3296-0.3379) | **0.3327** (0.3286-0.3369) | **0.3116** (0.3075-0.3156) | **0.3122** (0.3082-0.3163) |
| Zaykin | **0.6300** (0.6257-0.6342) | **0.5858** (0.5815-0.5901) | **0.5524** (0.5481-0.5568) | **0.5281** (0.5237-0.5325) | **0.5057** (0.5013-0.5101) | **0.4857** (0.4813-0.4901) | **0.4692** (0.4648-0.4735) | **0.4535** (0.4491-0.4578) | **0.4411** (0.4367-0.4454) | **0.4279** (0.4236-0.4323) | **0.4180** (0.4137-0.4223) | **0.4073** (0.4030-0.4116) | **0.3986** (0.3943-0.4029) | **0.3887** (0.3845-0.3930) | **0.3818** (0.3776-0.3861) |
| Dudbridge-Koeleman | **0.6451** (0.6409-0.6493) | **0.6034** (0.5991-0.6077) | **0.5867** (0.5824-0.5910) | **0.5581** (0.5537-0.5624) | **0.5465** (0.5421-0.5509) | **0.5226** (0.5182-0.5270) | **0.5123** (0.5080-0.5167) | **0.4934** (0.4891-0.4978) | **0.4874** (0.4830-0.4918) | **0.4726** (0.4682-0.4770) | **0.4646** (0.4603-0.4690) | **0.4512** (0.4468-0.4555) | **0.4469** (0.4426-0.4513) | **0.4345** (0.4302-0.4389) | **0.4305** (0.4261-0.4348) |
| Dudbridge-Koeleman2 | **0.6466** (0.6424-0.6507) | **0.5909** (0.5866-0.5952) | **0.5680** (0.5636-0.5723) | **0.5319** (0.5275-0.5363) | **0.5147** (0.5103-0.5191) | **0.4866** (0.4822-0.4910) | **0.4744** (0.4700-0.4788) | **0.4564** (0.4520-0.4607) | **0.4417** (0.4374-0.4461) | **0.4287** (0.4244-0.4331) | **0.4205** (0.4162-0.4248) | **0.4078** (0.4035-0.4121) | **0.3989** (0.3946-0.4032) | **0.3896** (0.3854-0.3939) | **0.3816** (0.3774-0.3859) |
| Taylor-Tibshirani | **0.2780** (0.2741-0.2819) | **0.2270** (0.2233-0.2307) | **0.1984** (0.1950-0.2020) | **0.1831** (0.1798-0.1865) | **0.1693** (0.1660-0.1726) | **0.1635** (0.1603-0.1668) | **0.1565** (0.1533-0.1597) | **0.1524** (0.1493-0.1556) | **0.1452** (0.1422-0.1484) | **0.1444** (0.1414-0.1475) | **0.1400** (0.1370-0.1430) | **0.1359** (0.1329-0.1389) | **0.1355** (0.1326-0.1386) | **0.1325** (0.1295-0.1355) | **0.1301** (0.1271-0.1330) |
| Jiang | **0.5364** (0.5321-0.5408) | **0.2652** (0.2614-0.2691) | **0.2584** (0.2545-0.2622) | **0.2171** (0.2135-0.2208) | **0.1984** (0.1949-0.2019) | **0.1914** (0.1880-0.1949) | **0.1870** (0.1836-0.1905) | **0.1800** (0.1767-0.1834) | **0.1760** (0.1727-0.1793) | **0.1701** (0.1668-0.1734) | **0.1650** (0.1618-0.1683) | **0.1609** (0.1577-0.1642) | **0.1582** (0.1550-0.1614) | **0.1571** (0.1539-0.1603) | **0.1543** (0.1512-0.1575) |
| Hotelling | **0.5828** (0.5785-0.5872) | **0.5505** (0.5461-0.5549) | **0.5219** (0.5175-0.5262) | **0.4997** (0.4953-0.5040) | **0.4782** (0.4738-0.4826) | **0.4572** (0.4528-0.4616) | **0.4382** (0.4338-0.4425) | **0.4203** (0.4160-0.4246) | **0.4009** (0.3967-0.4052) | **0.3798** (0.3755-0.3840) | **0.3593** (0.3551-0.3635) | **0.3328** (0.3287-0.3369) | **0.3069** (0.3028-0.3109) | **0.2737** (0.2698-0.2776) | **0.2290** (0.2253-0.2327) |
| Wilks | **0.5823** (0.5780-0.5866) | **0.5504** (0.5460-0.5547) | **0.5214** (0.5171-0.5258) | **0.4999** (0.4955-0.5043) | **0.4775** (0.4731-0.4819) | **0.4575** (0.4532-0.4619) | **0.4397** (0.4354-0.4441) | **0.4191** (0.4148-0.4234) | **0.4010** (0.3967-0.4053) | **0.3802** (0.3759-0.3844) | **0.3590** (0.3548-0.3632) | **0.3335** (0.3294-0.3376) | **0.3057** (0.3017-0.3098) | **0.2720** (0.2681-0.2759) | **0.2285** (0.2249-0.2322) |
