# Supplementary material for: Non‐parametric combination and related permutation tests for neuroimaging
Source: Hum Brain Mapp. 2016 Feb 5;37(4):1486–511. doi: 10.1002/hbm.23115 (PMC4783210; doi:10.1002/hbm.23115)
Supplement: Supplementary file 1 — Supporting Information [file HBM-37-1486-s001.zip › supporting_information/errorrates/histograms/simB_Weibull_ee_uncp.html]

Sim B, Weibull, EE, uncp

# Simulations B, Weibull, EE, uncp

---

| Method | ksig=1 / K=2 | ksig=1 / K=3 | ksig=1 / K=4 | ksig=1 / K=5 | ksig=1 / K=6 | ksig=1 / K=7 | ksig=1 / K=8 | ksig=1 / K=9 | ksig=1 / K=10 | ksig=1 / K=11 | ksig=1 / K=12 | ksig=1 / K=13 | ksig=1 / K=14 | ksig=1 / K=15 | ksig=1 / K=16 |
| --- | --- | --- | --- | --- | --- | --- | --- | --- | --- | --- | --- | --- | --- | --- | --- |
| Tippett | **0.6440** (0.6398-0.6481) | **0.6207** (0.6164-0.6249) | **0.6055** (0.6012-0.6098) | **0.5949** (0.5905-0.5992) | **0.5861** (0.5818-0.5904) | **0.5785** (0.5742-0.5828) | **0.5713** (0.5670-0.5757) | **0.5661** (0.5618-0.5705) | **0.5628** (0.5584-0.5671) | **0.5580** (0.5536-0.5623) | **0.5538** (0.5495-0.5582) | **0.5504** (0.5460-0.5548) | **0.5469** (0.5425-0.5513) | **0.5447** (0.5403-0.5490) | **0.5409** (0.5365-0.5453) |
| Fisher | **0.6333** (0.6291-0.6376) | **0.5965** (0.5922-0.6008) | **0.5669** (0.5626-0.5713) | **0.5445** (0.5402-0.5489) | **0.5239** (0.5195-0.5282) | **0.5075** (0.5031-0.5119) | **0.4920** (0.4876-0.4964) | **0.4778** (0.4735-0.4822) | **0.4659** (0.4615-0.4703) | **0.4547** (0.4503-0.4591) | **0.4444** (0.4401-0.4488) | **0.4335** (0.4291-0.4378) | **0.4253** (0.4209-0.4296) | **0.4159** (0.4116-0.4203) | **0.4091** (0.4048-0.4134) |
| Stouffer | **0.5681** (0.5637-0.5724) | **0.4822** (0.4778-0.4865) | **0.4239** (0.4196-0.4282) | **0.3779** (0.3736-0.3821) | **0.3411** (0.3369-0.3452) | **0.3163** (0.3122-0.3204) | **0.2910** (0.2870-0.2950) | **0.2719** (0.2680-0.2758) | **0.2545** (0.2507-0.2583) | **0.2395** (0.2358-0.2433) | **0.2296** (0.2259-0.2333) | **0.2171** (0.2135-0.2207) | **0.2087** (0.2051-0.2122) | **0.2017** (0.1982-0.2053) | **0.1942** (0.1908-0.1977) |
| Wilkinson | **0.0672** (0.0651-0.0695) | **0.0324** (0.0309-0.0340) | **0.0654** (0.0633-0.0676) | **0.0543** (0.0524-0.0563) | **0.0501** (0.0482-0.0520) | **0.0617** (0.0596-0.0638) | **0.0566** (0.0546-0.0586) | **0.0533** (0.0514-0.0553) | **0.0568** (0.0548-0.0589) | **0.0566** (0.0546-0.0587) | **0.0542** (0.0522-0.0562) | **0.0549** (0.0529-0.0569) | **0.0567** (0.0547-0.0588) | **0.0547** (0.0527-0.0567) | **0.0560** (0.0540-0.0580) |
| Winer | **0.5873** (0.5829-0.5916) | **0.5176** (0.5132-0.5220) | **0.4741** (0.4697-0.4784) | **0.4376** (0.4333-0.4420) | **0.4094** (0.4051-0.4138) | **0.3856** (0.3813-0.3898) | **0.3648** (0.3606-0.3691) | **0.3477** (0.3435-0.3518) | **0.3336** (0.3295-0.3378) | **0.3186** (0.3145-0.3227) | **0.3070** (0.3030-0.3111) | **0.2963** (0.2923-0.3003) | **0.2864** (0.2824-0.2904) | **0.2792** (0.2753-0.2831) | **0.2707** (0.2668-0.2746) |
| Edgington | **0.2338** (0.2301-0.2375) | **0.1654** (0.1621-0.1687) | **0.1415** (0.1385-0.1446) | **0.1285** (0.1256-0.1315) | **0.1200** (0.1172-0.1229) | **0.1141** (0.1113-0.1169) | **0.1074** (0.1047-0.1101) | **0.1034** (0.1007-0.1061) | **0.1009** (0.0983-0.1036) | **0.0986** (0.0960-0.1012) | **0.0946** (0.0920-0.0972) | **0.0909** (0.0884-0.0935) | **0.0902** (0.0877-0.0927) | **0.0892** (0.0868-0.0918) | **0.0873** (0.0848-0.0898) |
| Mudholkar-George | **0.6013** (0.5970-0.6056) | **0.5433** (0.5389-0.5476) | **0.5033** (0.4989-0.5076) | **0.4702** (0.4658-0.4746) | **0.4446** (0.4402-0.4489) | **0.4213** (0.4170-0.4256) | **0.4012** (0.3969-0.4055) | **0.3853** (0.3810-0.3896) | **0.3679** (0.3636-0.3721) | **0.3542** (0.3501-0.3584) | **0.3427** (0.3385-0.3469) | **0.3307** (0.3266-0.3349) | **0.3203** (0.3162-0.3244) | **0.3125** (0.3084-0.3166) | **0.3032** (0.2992-0.3073) |
| Friston | **0.1711** (0.1678-0.1744) | **0.1083** (0.1056-0.1111) | **0.0888** (0.0864-0.0913) | **0.0783** (0.0760-0.0807) | **0.0729** (0.0706-0.0752) | **0.0679** (0.0657-0.0701) | **0.0659** (0.0637-0.0681) | **0.0628** (0.0607-0.0650) | **0.0622** (0.0601-0.0643) | **0.0609** (0.0589-0.0631) | **0.0590** (0.0570-0.0611) | **0.0575** (0.0555-0.0595) | **0.0572** (0.0552-0.0592) | **0.0563** (0.0543-0.0583) | **0.0548** (0.0528-0.0568) |
| Darlington-Hayes | **0.6449** (0.6407-0.6491) | **0.5634** (0.5591-0.5678) | **0.5564** (0.5520-0.5607) | **0.4909** (0.4865-0.4952) | **0.4902** (0.4858-0.4946) | **0.4378** (0.4335-0.4422) | **0.4406** (0.4362-0.4449) | **0.4013** (0.3970-0.4056) | **0.3998** (0.3955-0.4041) | **0.3664** (0.3622-0.3707) | **0.3684** (0.3642-0.3727) | **0.3416** (0.3375-0.3458) | **0.3422** (0.3381-0.3464) | **0.3199** (0.3159-0.3240) | **0.3209** (0.3168-0.3250) |
| Zaykin | **0.6294** (0.6252-0.6337) | **0.5862** (0.5819-0.5905) | **0.5535** (0.5491-0.5578) | **0.5282** (0.5238-0.5326) | **0.5087** (0.5043-0.5131) | **0.4894** (0.4851-0.4938) | **0.4742** (0.4698-0.4786) | **0.4592** (0.4548-0.4635) | **0.4449** (0.4405-0.4493) | **0.4341** (0.4297-0.4384) | **0.4231** (0.4188-0.4275) | **0.4125** (0.4082-0.4169) | **0.4027** (0.3984-0.4070) | **0.3948** (0.3905-0.3991) | **0.3861** (0.3818-0.3903) |
| Dudbridge-Koeleman | **0.6454** (0.6412-0.6496) | **0.6073** (0.6030-0.6116) | **0.5912** (0.5868-0.5955) | **0.5613** (0.5569-0.5656) | **0.5530** (0.5487-0.5574) | **0.5297** (0.5253-0.5341) | **0.5225** (0.5181-0.5268) | **0.5033** (0.4990-0.5077) | **0.4969** (0.4925-0.5012) | **0.4822** (0.4778-0.4866) | **0.4770** (0.4726-0.4814) | **0.4634** (0.4590-0.4677) | **0.4592** (0.4549-0.4636) | **0.4463** (0.4420-0.4507) | **0.4440** (0.4396-0.4483) |
| Dudbridge-Koeleman2 | **0.6448** (0.6406-0.6490) | **0.5890** (0.5847-0.5933) | **0.5682** (0.5639-0.5725) | **0.5310** (0.5266-0.5354) | **0.5174** (0.5130-0.5217) | **0.4896** (0.4852-0.4940) | **0.4783** (0.4739-0.4827) | **0.4589** (0.4545-0.4632) | **0.4462** (0.4419-0.4506) | **0.4338** (0.4294-0.4381) | **0.4253** (0.4210-0.4297) | **0.4138** (0.4095-0.4181) | **0.4037** (0.3994-0.4080) | **0.3941** (0.3898-0.3984) | **0.3865** (0.3822-0.3908) |
| Taylor-Tibshirani | **0.2836** (0.2796-0.2875) | **0.2233** (0.2196-0.2269) | **0.2030** (0.1995-0.2065) | **0.1872** (0.1838-0.1906) | **0.1748** (0.1715-0.1782) | **0.1675** (0.1643-0.1708) | **0.1599** (0.1567-0.1631) | **0.1538** (0.1506-0.1569) | **0.1500** (0.1469-0.1532) | **0.1444** (0.1414-0.1475) | **0.1406** (0.1376-0.1437) | **0.1391** (0.1361-0.1422) | **0.1366** (0.1336-0.1396) | **0.1342** (0.1313-0.1373) | **0.1307** (0.1278-0.1337) |
| Jiang | **0.5429** (0.5386-0.5473) | **0.2678** (0.2639-0.2717) | **0.2593** (0.2555-0.2632) | **0.2188** (0.2152-0.2225) | **0.2048** (0.2013-0.2084) | **0.1977** (0.1942-0.2012) | **0.1907** (0.1873-0.1942) | **0.1850** (0.1816-0.1884) | **0.1831** (0.1798-0.1865) | **0.1775** (0.1741-0.1808) | **0.1724** (0.1691-0.1757) | **0.1696** (0.1663-0.1729) | **0.1689** (0.1657-0.1722) | **0.1650** (0.1617-0.1682) | **0.1632** (0.1600-0.1665) |
| Hotelling | **0.5721** (0.5677-0.5764) | **0.5374** (0.5330-0.5417) | **0.5104** (0.5061-0.5148) | **0.4877** (0.4833-0.4920) | **0.4700** (0.4656-0.4744) | **0.4492** (0.4448-0.4536) | **0.4327** (0.4283-0.4370) | **0.4132** (0.4089-0.4175) | **0.3935** (0.3892-0.3977) | **0.3749** (0.3707-0.3792) | **0.3528** (0.3486-0.3570) | **0.3271** (0.3230-0.3312) | **0.2985** (0.2945-0.3025) | **0.2632** (0.2593-0.2671) | **0.2233** (0.2197-0.2270) |
| Wilks | **0.5711** (0.5668-0.5755) | **0.5368** (0.5325-0.5412) | **0.5110** (0.5066-0.5154) | **0.4886** (0.4842-0.4929) | **0.4691** (0.4647-0.4735) | **0.4495** (0.4452-0.4539) | **0.4323** (0.4280-0.4366) | **0.4136** (0.4093-0.4179) | **0.3941** (0.3898-0.3984) | **0.3751** (0.3708-0.3793) | **0.3525** (0.3483-0.3567) | **0.3273** (0.3232-0.3314) | **0.2982** (0.2942-0.3022) | **0.2630** (0.2592-0.2669) | **0.2232** (0.2196-0.2269) |
