# Supplementary material for: Non‐parametric combination and related permutation tests for neuroimaging
Source: Hum Brain Mapp. 2016 Feb 5;37(4):1486–511. doi: 10.1002/hbm.23115 (PMC4783210; doi:10.1002/hbm.23115)
Supplement: Supplementary file 1 — Supporting Information [file HBM-37-1486-s001.zip › supporting_information/errorrates/histograms/simB_Weibull_ise_uncp.html]

Sim B, Weibull, ISE, uncp

# Simulations B, Weibull, ISE, uncp

---

| Method | ksig=1 / K=2 | ksig=1 / K=3 | ksig=1 / K=4 | ksig=1 / K=5 | ksig=1 / K=6 | ksig=1 / K=7 | ksig=1 / K=8 | ksig=1 / K=9 | ksig=1 / K=10 | ksig=1 / K=11 | ksig=1 / K=12 | ksig=1 / K=13 | ksig=1 / K=14 | ksig=1 / K=15 | ksig=1 / K=16 |
| --- | --- | --- | --- | --- | --- | --- | --- | --- | --- | --- | --- | --- | --- | --- | --- |
| Tippett | **0.6634** (0.6593-0.6676) | **0.6306** (0.6264-0.6348) | **0.6079** (0.6036-0.6122) | **0.5912** (0.5869-0.5955) | **0.5758** (0.5715-0.5801) | **0.5645** (0.5601-0.5688) | **0.5555** (0.5511-0.5599) | **0.5485** (0.5441-0.5528) | **0.5405** (0.5361-0.5448) | **0.5345** (0.5301-0.5389) | **0.5271** (0.5227-0.5315) | **0.5223** (0.5179-0.5266) | **0.5175** (0.5131-0.5219) | **0.5131** (0.5087-0.5175) | **0.5086** (0.5042-0.5129) |
| Fisher | **0.6485** (0.6443-0.6527) | **0.6017** (0.5974-0.6060) | **0.5688** (0.5645-0.5731) | **0.5428** (0.5384-0.5472) | **0.5226** (0.5182-0.5269) | **0.5042** (0.4998-0.5085) | **0.4876** (0.4832-0.4920) | **0.4755** (0.4711-0.4799) | **0.4631** (0.4587-0.4675) | **0.4510** (0.4466-0.4553) | **0.4402** (0.4359-0.4446) | **0.4321** (0.4278-0.4365) | **0.4223** (0.4180-0.4266) | **0.4145** (0.4102-0.4188) | **0.4083** (0.4040-0.4126) |
| Stouffer | **0.5667** (0.5623-0.5710) | **0.4735** (0.4691-0.4779) | **0.4120** (0.4077-0.4163) | **0.3664** (0.3622-0.3706) | **0.3319** (0.3277-0.3360) | **0.3055** (0.3015-0.3096) | **0.2825** (0.2786-0.2864) | **0.2634** (0.2595-0.2672) | **0.2474** (0.2436-0.2512) | **0.2357** (0.2320-0.2394) | **0.2233** (0.2197-0.2270) | **0.2151** (0.2115-0.2187) | **0.2064** (0.2029-0.2100) | **0.1957** (0.1922-0.1992) | **0.1891** (0.1857-0.1926) |
| Wilkinson | **0.0275** (0.0261-0.0289) | **0.0302** (0.0288-0.0318) | **0.0356** (0.0341-0.0373) | **0.0377** (0.0361-0.0394) | **0.0392** (0.0375-0.0409) | **0.0412** (0.0395-0.0430) | **0.0409** (0.0392-0.0427) | **0.0416** (0.0399-0.0434) | **0.0404** (0.0387-0.0421) | **0.0428** (0.0411-0.0446) | **0.0414** (0.0397-0.0432) | **0.0427** (0.0410-0.0445) | **0.0424** (0.0407-0.0442) | **0.0430** (0.0413-0.0448) | **0.0427** (0.0410-0.0445) |
| Winer | **0.5914** (0.5870-0.5957) | **0.5145** (0.5101-0.5189) | **0.4648** (0.4605-0.4692) | **0.4284** (0.4240-0.4327) | **0.4007** (0.3964-0.4050) | **0.3769** (0.3727-0.3812) | **0.3586** (0.3544-0.3628) | **0.3396** (0.3354-0.3437) | **0.3261** (0.3220-0.3302) | **0.3148** (0.3107-0.3189) | **0.3044** (0.3004-0.3085) | **0.2962** (0.2922-0.3002) | **0.2876** (0.2836-0.2916) | **0.2789** (0.2750-0.2828) | **0.2698** (0.2659-0.2737) |
| Edgington | **0.2360** (0.2323-0.2397) | **0.1546** (0.1514-0.1578) | **0.1227** (0.1199-0.1256) | **0.1127** (0.1099-0.1155) | **0.1053** (0.1027-0.1080) | **0.0982** (0.0956-0.1009) | **0.0953** (0.0928-0.0979) | **0.0928** (0.0903-0.0954) | **0.0916** (0.0891-0.0942) | **0.0884** (0.0859-0.0909) | **0.0856** (0.0831-0.0880) | **0.0858** (0.0834-0.0883) | **0.0851** (0.0827-0.0876) | **0.0829** (0.0805-0.0854) | **0.0819** (0.0795-0.0844) |
| Mudholkar-George | **0.6086** (0.6043-0.6128) | **0.5430** (0.5386-0.5473) | **0.4964** (0.4920-0.5008) | **0.4619** (0.4576-0.4663) | **0.4361** (0.4317-0.4404) | **0.4133** (0.4090-0.4176) | **0.3960** (0.3917-0.4003) | **0.3771** (0.3729-0.3814) | **0.3636** (0.3594-0.3678) | **0.3517** (0.3475-0.3559) | **0.3412** (0.3371-0.3454) | **0.3317** (0.3276-0.3358) | **0.3202** (0.3161-0.3243) | **0.3112** (0.3072-0.3153) | **0.3031** (0.2991-0.3071) |
| Friston | **0.1465** (0.1434-0.1496) | **0.1001** (0.0975-0.1027) | **0.0800** (0.0776-0.0824) | **0.0711** (0.0689-0.0734) | **0.0661** (0.0639-0.0683) | **0.0624** (0.0604-0.0646) | **0.0606** (0.0585-0.0627) | **0.0571** (0.0551-0.0592) | **0.0553** (0.0534-0.0574) | **0.0540** (0.0521-0.0561) | **0.0528** (0.0509-0.0548) | **0.0526** (0.0507-0.0546) | **0.0520** (0.0501-0.0540) | **0.0509** (0.0490-0.0529) | **0.0502** (0.0484-0.0522) |
| Darlington-Hayes | **0.6634** (0.6592-0.6675) | **0.5779** (0.5736-0.5823) | **0.5682** (0.5639-0.5726) | **0.5012** (0.4968-0.5056) | **0.4989** (0.4945-0.5033) | **0.4476** (0.4433-0.4520) | **0.4482** (0.4439-0.4526) | **0.4062** (0.4019-0.4105) | **0.4083** (0.4040-0.4127) | **0.3732** (0.3690-0.3775) | **0.3774** (0.3732-0.3817) | **0.3507** (0.3465-0.3549) | **0.3504** (0.3463-0.3546) | **0.3240** (0.3199-0.3281) | **0.3256** (0.3215-0.3297) |
| Zaykin | **0.6525** (0.6483-0.6566) | **0.6020** (0.5977-0.6063) | **0.5649** (0.5605-0.5692) | **0.5364** (0.5321-0.5408) | **0.5147** (0.5103-0.5191) | **0.4953** (0.4910-0.4997) | **0.4780** (0.4737-0.4824) | **0.4634** (0.4591-0.4678) | **0.4495** (0.4452-0.4539) | **0.4363** (0.4320-0.4407) | **0.4264** (0.4221-0.4308) | **0.4154** (0.4111-0.4197) | **0.4046** (0.4003-0.4089) | **0.3973** (0.3930-0.4016) | **0.3877** (0.3834-0.3919) |
| Dudbridge-Koeleman | **0.6641** (0.6599-0.6682) | **0.6209** (0.6166-0.6251) | **0.6007** (0.5964-0.6050) | **0.5675** (0.5632-0.5719) | **0.5546** (0.5502-0.5589) | **0.5315** (0.5271-0.5359) | **0.5236** (0.5192-0.5279) | **0.5043** (0.4999-0.5086) | **0.4981** (0.4938-0.5025) | **0.4822** (0.4778-0.4865) | **0.4764** (0.4720-0.4808) | **0.4631** (0.4588-0.4675) | **0.4564** (0.4521-0.4608) | **0.4437** (0.4393-0.4481) | **0.4409** (0.4365-0.4452) |
| Dudbridge-Koeleman2 | **0.6635** (0.6593-0.6676) | **0.6062** (0.6019-0.6105) | **0.5790** (0.5747-0.5834) | **0.5383** (0.5339-0.5426) | **0.5220** (0.5176-0.5264) | **0.4980** (0.4936-0.5023) | **0.4829** (0.4785-0.4873) | **0.4648** (0.4604-0.4691) | **0.4536** (0.4492-0.4580) | **0.4387** (0.4343-0.4430) | **0.4282** (0.4239-0.4326) | **0.4170** (0.4127-0.4213) | **0.4059** (0.4016-0.4102) | **0.3973** (0.3930-0.4016) | **0.3895** (0.3852-0.3937) |
| Taylor-Tibshirani | **0.2893** (0.2853-0.2933) | **0.2056** (0.2021-0.2092) | **0.1749** (0.1716-0.1782) | **0.1613** (0.1581-0.1645) | **0.1525** (0.1494-0.1557) | **0.1455** (0.1424-0.1486) | **0.1374** (0.1344-0.1404) | **0.1342** (0.1312-0.1372) | **0.1328** (0.1298-0.1358) | **0.1287** (0.1258-0.1317) | **0.1277** (0.1248-0.1306) | **0.1254** (0.1225-0.1283) | **0.1223** (0.1195-0.1252) | **0.1203** (0.1174-0.1231) | **0.1178** (0.1150-0.1206) |
| Jiang | **0.5110** (0.5066-0.5154) | **0.3274** (0.3233-0.3315) | **0.2525** (0.2487-0.2563) | **0.2156** (0.2120-0.2192) | **0.1951** (0.1916-0.1986) | **0.1834** (0.1800-0.1868) | **0.1754** (0.1721-0.1787) | **0.1650** (0.1618-0.1683) | **0.1632** (0.1600-0.1665) | **0.1628** (0.1596-0.1661) | **0.1557** (0.1526-0.1589) | **0.1536** (0.1504-0.1568) | **0.1490** (0.1459-0.1521) | **0.1472** (0.1441-0.1504) | **0.1432** (0.1401-0.1463) |
| Hotelling | **0.6049** (0.6006-0.6092) | **0.5633** (0.5589-0.5676) | **0.5303** (0.5259-0.5347) | **0.5045** (0.5001-0.5088) | **0.4762** (0.4718-0.4806) | **0.4547** (0.4503-0.4591) | **0.4323** (0.4280-0.4366) | **0.4101** (0.4058-0.4144) | **0.3858** (0.3816-0.3901) | **0.3621** (0.3579-0.3663) | **0.3332** (0.3291-0.3374) | **0.3040** (0.3000-0.3080) | **0.2723** (0.2685-0.2763) | **0.2362** (0.2325-0.2399) | **0.1929** (0.1895-0.1964) |
| Wilks | **0.6047** (0.6004-0.6090) | **0.5631** (0.5587-0.5674) | **0.5292** (0.5248-0.5336) | **0.5029** (0.4985-0.5073) | **0.4764** (0.4720-0.4807) | **0.4539** (0.4496-0.4583) | **0.4312** (0.4269-0.4355) | **0.4096** (0.4053-0.4139) | **0.3847** (0.3805-0.3890) | **0.3618** (0.3576-0.3660) | **0.3330** (0.3289-0.3371) | **0.3049** (0.3009-0.3090) | **0.2722** (0.2683-0.2761) | **0.2355** (0.2318-0.2392) | **0.1924** (0.1890-0.1959) |
