# Supplementary material for: Non‐parametric combination and related permutation tests for neuroimaging
Source: Hum Brain Mapp. 2016 Feb 5;37(4):1486–511. doi: 10.1002/hbm.23115 (PMC4783210; doi:10.1002/hbm.23115)
Supplement: Supplementary file 1 — Supporting Information [file HBM-37-1486-s001.zip › supporting_information/errorrates/histograms/simC_Gauss_ee+ise_uncp.html]

Sim C, Gauss, EE+ISE, uncp

# Simulations C, Gauss, EE+ISE, uncp

---

| Method | ksig=1 / K=16 | ksig=2 / K=16 | ksig=3 / K=16 | ksig=4 / K=16 | ksig=5 / K=16 | ksig=6 / K=16 | ksig=7 / K=16 | ksig=8 / K=16 | ksig=9 / K=16 | ksig=10 / K=16 | ksig=11 / K=16 | ksig=12 / K=16 | ksig=13 / K=16 | ksig=14 / K=16 | ksig=15 / K=16 | ksig=16 / K=16 |
| --- | --- | --- | --- | --- | --- | --- | --- | --- | --- | --- | --- | --- | --- | --- | --- | --- |
| Tippett | **0.1587** (0.1555-0.1619) | **0.2532** (0.2494-0.2571) | **0.3365** (0.3323-0.3406) | **0.4052** (0.4009-0.4096) | **0.4670** (0.4626-0.4713) | **0.5255** (0.5211-0.5299) | **0.5754** (0.5710-0.5797) | **0.6177** (0.6134-0.6220) | **0.6553** (0.6511-0.6595) | **0.6868** (0.6827-0.6908) | **0.7170** (0.7131-0.7210) | **0.7423** (0.7384-0.7461) | **0.7676** (0.7639-0.7713) | **0.7874** (0.7838-0.7910) | **0.8065** (0.8030-0.8099) | **0.8273** (0.8240-0.8306) |
| Fisher | **0.1352** (0.1322-0.1382) | **0.2745** (0.2706-0.2784) | **0.4438** (0.4394-0.4481) | **0.6087** (0.6044-0.6130) | **0.7435** (0.7396-0.7473) | **0.8391** (0.8358-0.8423) | **0.9023** (0.8996-0.9048) | **0.9418** (0.9397-0.9438) | **0.9664** (0.9648-0.9679) | **0.9802** (0.9790-0.9814) | **0.9884** (0.9874-0.9893) | **0.9926** (0.9919-0.9934) | **0.9954** (0.9948-0.9960) | **0.9972** (0.9967-0.9976) | **0.9980** (0.9976-0.9984) | **0.9989** (0.9986-0.9992) |
| Stouffer | **0.1025** (0.0999-0.1052) | **0.1920** (0.1886-0.1955) | **0.3198** (0.3157-0.3239) | **0.4677** (0.4633-0.4721) | **0.6164** (0.6122-0.6207) | **0.7470** (0.7432-0.7508) | **0.8452** (0.8420-0.8484) | **0.9135** (0.9110-0.9159) | **0.9520** (0.9501-0.9539) | **0.9750** (0.9736-0.9763) | **0.9872** (0.9861-0.9881) | **0.9932** (0.9925-0.9939) | **0.9964** (0.9958-0.9969) | **0.9980** (0.9975-0.9983) | **0.9989** (0.9986-0.9992) | **0.9996** (0.9994-0.9997) |
| Wilkinson | **0.0498** (0.0479-0.0517) | **0.0924** (0.0899-0.0949) | **0.1599** (0.1567-0.1631) | **0.2526** (0.2488-0.2564) | **0.3776** (0.3733-0.3818) | **0.5141** (0.5097-0.5185) | **0.6458** (0.6416-0.6500) | **0.7530** (0.7492-0.7568) | **0.8360** (0.8327-0.8392) | **0.8927** (0.8899-0.8953) | **0.9319** (0.9297-0.9341) | **0.9548** (0.9529-0.9565) | **0.9701** (0.9686-0.9716) | **0.9807** (0.9794-0.9818) | **0.9872** (0.9862-0.9882) | **0.9921** (0.9913-0.9929) |
| Winer | **0.1077** (0.1050-0.1105) | **0.2033** (0.1998-0.2069) | **0.3363** (0.3322-0.3404) | **0.4875** (0.4831-0.4919) | **0.6357** (0.6315-0.6399) | **0.7596** (0.7558-0.7633) | **0.8535** (0.8503-0.8565) | **0.9167** (0.9142-0.9191) | **0.9545** (0.9527-0.9563) | **0.9761** (0.9747-0.9774) | **0.9875** (0.9864-0.9884) | **0.9933** (0.9925-0.9939) | **0.9963** (0.9957-0.9968) | **0.9981** (0.9977-0.9984) | **0.9989** (0.9986-0.9992) | **0.9995** (0.9993-0.9997) |
| Edgington | **0.0848** (0.0824-0.0873) | **0.1421** (0.1391-0.1452) | **0.2187** (0.2151-0.2223) | **0.3254** (0.3213-0.3296) | **0.4528** (0.4484-0.4571) | **0.5858** (0.5815-0.5901) | **0.7130** (0.7090-0.7169) | **0.8255** (0.8222-0.8288) | **0.9034** (0.9008-0.9059) | **0.9512** (0.9493-0.9530) | **0.9764** (0.9750-0.9777) | **0.9890** (0.9881-0.9899) | **0.9948** (0.9942-0.9954) | **0.9974** (0.9969-0.9978) | **0.9985** (0.9981-0.9988) | **0.9992** (0.9989-0.9994) |
| Mudholkar-George | **0.1130** (0.1102-0.1158) | **0.2178** (0.2142-0.2214) | **0.3579** (0.3537-0.3621) | **0.5118** (0.5075-0.5162) | **0.6561** (0.6519-0.6602) | **0.7754** (0.7717-0.7790) | **0.8617** (0.8586-0.8647) | **0.9212** (0.9188-0.9235) | **0.9563** (0.9544-0.9580) | **0.9768** (0.9754-0.9781) | **0.9878** (0.9868-0.9887) | **0.9931** (0.9924-0.9938) | **0.9963** (0.9957-0.9968) | **0.9979** (0.9975-0.9983) | **0.9988** (0.9985-0.9991) | **0.9996** (0.9994-0.9997) |
| Friston | **0.0567** (0.0547-0.0588) | **0.0670** (0.0648-0.0692) | **0.0784** (0.0760-0.0807) | **0.0924** (0.0899-0.0949) | **0.1073** (0.1046-0.1101) | **0.1250** (0.1221-0.1279) | **0.1460** (0.1430-0.1491) | **0.1704** (0.1671-0.1737) | **0.2022** (0.1987-0.2057) | **0.2394** (0.2357-0.2431) | **0.2875** (0.2835-0.2914) | **0.3489** (0.3448-0.3531) | **0.4276** (0.4233-0.4319) | **0.5313** (0.5269-0.5356) | **0.6714** (0.6673-0.6755) | **0.8601** (0.8570-0.8631) |
| Darlington-Hayes | **0.1259** (0.1230-0.1288) | **0.2582** (0.2544-0.2620) | **0.4302** (0.4258-0.4345) | **0.6009** (0.5966-0.6052) | **0.7392** (0.7353-0.7430) | **0.8364** (0.8331-0.8396) | **0.8996** (0.8969-0.9022) | **0.9365** (0.9343-0.9386) | **0.9610** (0.9593-0.9627) | **0.9754** (0.9740-0.9767) | **0.9840** (0.9828-0.9850) | **0.9895** (0.9886-0.9904) | **0.9926** (0.9919-0.9934) | **0.9950** (0.9944-0.9956) | **0.9964** (0.9959-0.9969) | **0.9976** (0.9971-0.9980) |
| Zaykin | **0.1263** (0.1235-0.1293) | **0.2648** (0.2609-0.2687) | **0.4298** (0.4255-0.4341) | **0.5933** (0.5889-0.5976) | **0.7269** (0.7230-0.7308) | **0.8238** (0.8204-0.8271) | **0.8877** (0.8849-0.8905) | **0.9284** (0.9261-0.9306) | **0.9556** (0.9537-0.9573) | **0.9730** (0.9715-0.9744) | **0.9820** (0.9808-0.9831) | **0.9888** (0.9878-0.9897) | **0.9925** (0.9917-0.9932) | **0.9945** (0.9938-0.9951) | **0.9963** (0.9958-0.9968) | **0.9973** (0.9968-0.9977) |
| Dudbridge-Koeleman | **0.1419** (0.1389-0.1450) | **0.2905** (0.2866-0.2945) | **0.4631** (0.4588-0.4675) | **0.6188** (0.6146-0.6231) | **0.7436** (0.7397-0.7474) | **0.8332** (0.8299-0.8364) | **0.8897** (0.8869-0.8924) | **0.9281** (0.9258-0.9303) | **0.9530** (0.9511-0.9548) | **0.9690** (0.9674-0.9705) | **0.9792** (0.9779-0.9804) | **0.9857** (0.9846-0.9867) | **0.9899** (0.9889-0.9907) | **0.9927** (0.9919-0.9934) | **0.9948** (0.9942-0.9954) | **0.9962** (0.9956-0.9967) |
| Dudbridge-Koeleman2 | **0.1276** (0.1247-0.1305) | **0.2635** (0.2597-0.2674) | **0.4326** (0.4283-0.4370) | **0.5933** (0.5890-0.5976) | **0.7271** (0.7232-0.7310) | **0.8236** (0.8203-0.8269) | **0.8887** (0.8859-0.8914) | **0.9292** (0.9269-0.9314) | **0.9563** (0.9545-0.9581) | **0.9722** (0.9707-0.9736) | **0.9825** (0.9813-0.9836) | **0.9889** (0.9880-0.9898) | **0.9925** (0.9917-0.9932) | **0.9947** (0.9940-0.9953) | **0.9962** (0.9956-0.9967) | **0.9971** (0.9966-0.9975) |
| Taylor-Tibshirani | **0.1047** (0.1021-0.1075) | **0.1976** (0.1942-0.2012) | **0.3282** (0.3241-0.3323) | **0.4857** (0.4814-0.4901) | **0.6396** (0.6354-0.6438) | **0.7734** (0.7697-0.7770) | **0.8694** (0.8664-0.8723) | **0.9296** (0.9273-0.9318) | **0.9626** (0.9609-0.9643) | **0.9801** (0.9789-0.9813) | **0.9896** (0.9887-0.9905) | **0.9943** (0.9936-0.9949) | **0.9966** (0.9960-0.9970) | **0.9981** (0.9976-0.9984) | **0.9988** (0.9985-0.9991) | **0.9994** (0.9992-0.9996) |
| Jiang | **0.1116** (0.1089-0.1144) | **0.2260** (0.2223-0.2296) | **0.3842** (0.3799-0.3885) | **0.5549** (0.5506-0.5593) | **0.7002** (0.6962-0.7042) | **0.8078** (0.8043-0.8112) | **0.8792** (0.8763-0.8820) | **0.9246** (0.9222-0.9269) | **0.9535** (0.9516-0.9553) | **0.9714** (0.9699-0.9728) | **0.9819** (0.9807-0.9830) | **0.9885** (0.9875-0.9894) | **0.9924** (0.9916-0.9931) | **0.9948** (0.9941-0.9954) | **0.9961** (0.9955-0.9966) | **0.9973** (0.9968-0.9977) |
| Hotelling | **0.0618** (0.0597-0.0639) | **0.0755** (0.0733-0.0779) | **0.0900** (0.0875-0.0925) | **0.1058** (0.1032-0.1086) | **0.1183** (0.1155-0.1211) | **0.1317** (0.1288-0.1347) | **0.1476** (0.1445-0.1507) | **0.1608** (0.1576-0.1641) | **0.1784** (0.1751-0.1818) | **0.1913** (0.1878-0.1947) | **0.2065** (0.2030-0.2101) | **0.2223** (0.2187-0.2260) | **0.2356** (0.2319-0.2393) | **0.2479** (0.2441-0.2517) | **0.2634** (0.2595-0.2673) | **0.2748** (0.2709-0.2788) |
| Wilks | **0.0629** (0.0608-0.0650) | **0.0776** (0.0753-0.0800) | **0.0895** (0.0870-0.0920) | **0.1033** (0.1007-0.1060) | **0.1168** (0.1141-0.1197) | **0.1314** (0.1285-0.1344) | **0.1466** (0.1435-0.1497) | **0.1617** (0.1585-0.1650) | **0.1758** (0.1725-0.1792) | **0.1876** (0.1842-0.1910) | **0.2038** (0.2003-0.2073) | **0.2179** (0.2143-0.2215) | **0.2326** (0.2290-0.2364) | **0.2466** (0.2428-0.2504) | **0.2594** (0.2555-0.2632) | **0.2715** (0.2677-0.2755) |
