# Supplementary material for: Non‐parametric combination and related permutation tests for neuroimaging
Source: Hum Brain Mapp. 2016 Feb 5;37(4):1486–511. doi: 10.1002/hbm.23115 (PMC4783210; doi:10.1002/hbm.23115)
Supplement: Supplementary file 1 — Supporting Information [file HBM-37-1486-s001.zip › supporting_information/errorrates/histograms/simC_Gauss_ee_uncp.html]

Sim C, Gauss, EE, uncp

# Simulations C, Gauss, EE, uncp

---

| Method | ksig=1 / K=16 | ksig=2 / K=16 | ksig=3 / K=16 | ksig=4 / K=16 | ksig=5 / K=16 | ksig=6 / K=16 | ksig=7 / K=16 | ksig=8 / K=16 | ksig=9 / K=16 | ksig=10 / K=16 | ksig=11 / K=16 | ksig=12 / K=16 | ksig=13 / K=16 | ksig=14 / K=16 | ksig=15 / K=16 | ksig=16 / K=16 |
| --- | --- | --- | --- | --- | --- | --- | --- | --- | --- | --- | --- | --- | --- | --- | --- | --- |
| Tippett | **0.1581** (0.1549-0.1613) | **0.2485** (0.2447-0.2523) | **0.3299** (0.3258-0.3340) | **0.3997** (0.3955-0.4040) | **0.4588** (0.4544-0.4632) | **0.5146** (0.5102-0.5189) | **0.5605** (0.5561-0.5648) | **0.6014** (0.5971-0.6057) | **0.6379** (0.6337-0.6421) | **0.6692** (0.6651-0.6733) | **0.6982** (0.6941-0.7022) | **0.7258** (0.7218-0.7297) | **0.7508** (0.7469-0.7545) | **0.7692** (0.7655-0.7729) | **0.7924** (0.7888-0.7959) | **0.8088** (0.8054-0.8123) |
| Fisher | **0.1358** (0.1328-0.1388) | **0.2731** (0.2692-0.2770) | **0.4390** (0.4346-0.4433) | **0.5986** (0.5943-0.6029) | **0.7302** (0.7263-0.7341) | **0.8256** (0.8222-0.8289) | **0.8913** (0.8885-0.8940) | **0.9325** (0.9302-0.9346) | **0.9579** (0.9561-0.9596) | **0.9736** (0.9722-0.9750) | **0.9836** (0.9825-0.9847) | **0.9899** (0.9890-0.9907) | **0.9942** (0.9935-0.9948) | **0.9964** (0.9959-0.9969) | **0.9978** (0.9973-0.9982) | **0.9988** (0.9984-0.9990) |
| Stouffer | **0.1037** (0.1011-0.1064) | **0.1906** (0.1871-0.1940) | **0.3118** (0.3078-0.3159) | **0.4561** (0.4518-0.4605) | **0.6032** (0.5989-0.6075) | **0.7343** (0.7305-0.7382) | **0.8334** (0.8301-0.8367) | **0.9004** (0.8977-0.9030) | **0.9433** (0.9412-0.9453) | **0.9691** (0.9676-0.9706) | **0.9829** (0.9817-0.9840) | **0.9911** (0.9902-0.9919) | **0.9956** (0.9950-0.9961) | **0.9980** (0.9975-0.9983) | **0.9991** (0.9988-0.9994) | **0.9997** (0.9995-0.9998) |
| Wilkinson | **0.0523** (0.0503-0.0542) | **0.0925** (0.0900-0.0950) | **0.1537** (0.1506-0.1569) | **0.2469** (0.2432-0.2507) | **0.3660** (0.3617-0.3702) | **0.5010** (0.4967-0.5054) | **0.6312** (0.6269-0.6354) | **0.7417** (0.7378-0.7455) | **0.8232** (0.8199-0.8266) | **0.8807** (0.8778-0.8835) | **0.9194** (0.9170-0.9217) | **0.9459** (0.9439-0.9478) | **0.9642** (0.9625-0.9658) | **0.9752** (0.9738-0.9765) | **0.9835** (0.9824-0.9846) | **0.9892** (0.9882-0.9900) |
| Winer | **0.1081** (0.1054-0.1109) | **0.2027** (0.1992-0.2063) | **0.3298** (0.3257-0.3339) | **0.4788** (0.4744-0.4832) | **0.6231** (0.6188-0.6273) | **0.7467** (0.7429-0.7505) | **0.8418** (0.8386-0.8450) | **0.9055** (0.9029-0.9081) | **0.9453** (0.9432-0.9472) | **0.9693** (0.9678-0.9708) | **0.9828** (0.9816-0.9839) | **0.9911** (0.9902-0.9919) | **0.9955** (0.9949-0.9960) | **0.9979** (0.9975-0.9983) | **0.9990** (0.9987-0.9993) | **0.9996** (0.9994-0.9998) |
| Edgington | **0.0870** (0.0846-0.0895) | **0.1405** (0.1375-0.1436) | **0.2195** (0.2159-0.2232) | **0.3216** (0.3175-0.3257) | **0.4442** (0.4398-0.4485) | **0.5752** (0.5709-0.5795) | **0.7071** (0.7031-0.7111) | **0.8151** (0.8117-0.8185) | **0.8934** (0.8907-0.8961) | **0.9430** (0.9410-0.9450) | **0.9716** (0.9701-0.9730) | **0.9850** (0.9839-0.9860) | **0.9927** (0.9919-0.9934) | **0.9967** (0.9961-0.9971) | **0.9983** (0.9979-0.9986) | **0.9994** (0.9991-0.9995) |
| Mudholkar-George | **0.1133** (0.1106-0.1161) | **0.2160** (0.2124-0.2196) | **0.3503** (0.3462-0.3545) | **0.5025** (0.4982-0.5069) | **0.6437** (0.6395-0.6478) | **0.7627** (0.7590-0.7664) | **0.8525** (0.8494-0.8556) | **0.9099** (0.9074-0.9124) | **0.9472** (0.9452-0.9492) | **0.9708** (0.9693-0.9722) | **0.9832** (0.9820-0.9843) | **0.9906** (0.9897-0.9914) | **0.9952** (0.9946-0.9958) | **0.9978** (0.9973-0.9982) | **0.9989** (0.9985-0.9991) | **0.9996** (0.9994-0.9997) |
| Friston | **0.0596** (0.0575-0.0617) | **0.0692** (0.0670-0.0714) | **0.0812** (0.0789-0.0837) | **0.0936** (0.0911-0.0962) | **0.1080** (0.1053-0.1108) | **0.1270** (0.1241-0.1299) | **0.1460** (0.1430-0.1491) | **0.1737** (0.1704-0.1771) | **0.2036** (0.2001-0.2072) | **0.2419** (0.2381-0.2457) | **0.2887** (0.2848-0.2927) | **0.3474** (0.3433-0.3516) | **0.4244** (0.4201-0.4287) | **0.5256** (0.5213-0.5300) | **0.6643** (0.6601-0.6684) | **0.8491** (0.8459-0.8522) |
| Darlington-Hayes | **0.1272** (0.1243-0.1302) | **0.2572** (0.2533-0.2610) | **0.4269** (0.4226-0.4312) | **0.5924** (0.5880-0.5967) | **0.7257** (0.7218-0.7296) | **0.8229** (0.8195-0.8262) | **0.8874** (0.8846-0.8901) | **0.9263** (0.9240-0.9286) | **0.9519** (0.9500-0.9537) | **0.9687** (0.9671-0.9702) | **0.9788** (0.9775-0.9800) | **0.9857** (0.9846-0.9867) | **0.9905** (0.9896-0.9913) | **0.9932** (0.9924-0.9938) | **0.9950** (0.9943-0.9956) | **0.9966** (0.9961-0.9971) |
| Zaykin | **0.1298** (0.1268-0.1327) | **0.2624** (0.2585-0.2662) | **0.4274** (0.4231-0.4318) | **0.5850** (0.5807-0.5893) | **0.7139** (0.7099-0.7179) | **0.8099** (0.8065-0.8134) | **0.8760** (0.8731-0.8789) | **0.9179** (0.9154-0.9203) | **0.9451** (0.9430-0.9470) | **0.9642** (0.9626-0.9658) | **0.9759** (0.9745-0.9772) | **0.9846** (0.9835-0.9857) | **0.9895** (0.9886-0.9904) | **0.9928** (0.9920-0.9935) | **0.9954** (0.9948-0.9960) | **0.9971** (0.9966-0.9975) |
| Dudbridge-Koeleman | **0.1436** (0.1405-0.1467) | **0.2898** (0.2859-0.2938) | **0.4591** (0.4548-0.4635) | **0.6107** (0.6064-0.6149) | **0.7309** (0.7270-0.7347) | **0.8166** (0.8132-0.8200) | **0.8774** (0.8745-0.8802) | **0.9173** (0.9149-0.9197) | **0.9436** (0.9416-0.9456) | **0.9611** (0.9594-0.9628) | **0.9719** (0.9704-0.9733) | **0.9807** (0.9794-0.9818) | **0.9859** (0.9848-0.9869) | **0.9896** (0.9887-0.9905) | **0.9924** (0.9916-0.9931) | **0.9943** (0.9936-0.9950) |
| Dudbridge-Koeleman2 | **0.1286** (0.1257-0.1316) | **0.2622** (0.2583-0.2661) | **0.4279** (0.4236-0.4322) | **0.5848** (0.5805-0.5891) | **0.7155** (0.7116-0.7195) | **0.8093** (0.8059-0.8127) | **0.8763** (0.8733-0.8791) | **0.9171** (0.9147-0.9195) | **0.9454** (0.9434-0.9474) | **0.9637** (0.9621-0.9653) | **0.9762** (0.9748-0.9775) | **0.9841** (0.9830-0.9852) | **0.9898** (0.9888-0.9906) | **0.9928** (0.9921-0.9935) | **0.9952** (0.9946-0.9958) | **0.9967** (0.9962-0.9972) |
| Taylor-Tibshirani | **0.1043** (0.1016-0.1070) | **0.1961** (0.1927-0.1996) | **0.3234** (0.3193-0.3275) | **0.4739** (0.4695-0.4783) | **0.6296** (0.6254-0.6338) | **0.7613** (0.7575-0.7650) | **0.8588** (0.8557-0.8618) | **0.9183** (0.9159-0.9207) | **0.9545** (0.9527-0.9563) | **0.9748** (0.9734-0.9761) | **0.9853** (0.9842-0.9864) | **0.9919** (0.9910-0.9926) | **0.9959** (0.9953-0.9964) | **0.9977** (0.9973-0.9981) | **0.9987** (0.9984-0.9990) | **0.9995** (0.9992-0.9996) |
| Jiang | **0.1133** (0.1106-0.1161) | **0.2253** (0.2216-0.2289) | **0.3801** (0.3759-0.3844) | **0.5453** (0.5409-0.5497) | **0.6887** (0.6846-0.6927) | **0.7931** (0.7895-0.7966) | **0.8669** (0.8639-0.8698) | **0.9128** (0.9103-0.9152) | **0.9429** (0.9408-0.9449) | **0.9628** (0.9611-0.9644) | **0.9760** (0.9746-0.9773) | **0.9844** (0.9833-0.9854) | **0.9899** (0.9890-0.9907) | **0.9936** (0.9928-0.9942) | **0.9954** (0.9947-0.9959) | **0.9969** (0.9964-0.9974) |
| Hotelling | **0.0628** (0.0607-0.0649) | **0.0762** (0.0739-0.0785) | **0.0888** (0.0864-0.0914) | **0.1020** (0.0994-0.1047) | **0.1170** (0.1143-0.1199) | **0.1305** (0.1275-0.1334) | **0.1456** (0.1425-0.1487) | **0.1610** (0.1578-0.1643) | **0.1736** (0.1703-0.1769) | **0.1885** (0.1851-0.1920) | **0.2030** (0.1995-0.2065) | **0.2161** (0.2125-0.2197) | **0.2299** (0.2262-0.2336) | **0.2436** (0.2398-0.2474) | **0.2560** (0.2522-0.2599) | **0.2711** (0.2672-0.2750) |
| Wilks | **0.0605** (0.0585-0.0627) | **0.0734** (0.0712-0.0757) | **0.0870** (0.0846-0.0895) | **0.0992** (0.0966-0.1019) | **0.1138** (0.1110-0.1166) | **0.1277** (0.1248-0.1306) | **0.1417** (0.1386-0.1447) | **0.1556** (0.1524-0.1588) | **0.1708** (0.1675-0.1741) | **0.1841** (0.1808-0.1876) | **0.1982** (0.1947-0.2017) | **0.2115** (0.2079-0.2151) | **0.2285** (0.2248-0.2322) | **0.2432** (0.2395-0.2470) | **0.2566** (0.2528-0.2604) | **0.2685** (0.2647-0.2724) |
