# Supplementary material for: Non‐parametric combination and related permutation tests for neuroimaging
Source: Hum Brain Mapp. 2016 Feb 5;37(4):1486–511. doi: 10.1002/hbm.23115 (PMC4783210; doi:10.1002/hbm.23115)
Supplement: Supplementary file 1 — Supporting Information [file HBM-37-1486-s001.zip › supporting_information/errorrates/histograms/simC_Gauss_ise_uncp.html]

Sim C, Gauss, ISE, uncp

# Simulations C, Gauss, ISE, uncp

---

| Method | ksig=1 / K=16 | ksig=2 / K=16 | ksig=3 / K=16 | ksig=4 / K=16 | ksig=5 / K=16 | ksig=6 / K=16 | ksig=7 / K=16 | ksig=8 / K=16 | ksig=9 / K=16 | ksig=10 / K=16 | ksig=11 / K=16 | ksig=12 / K=16 | ksig=13 / K=16 | ksig=14 / K=16 | ksig=15 / K=16 | ksig=16 / K=16 |
| --- | --- | --- | --- | --- | --- | --- | --- | --- | --- | --- | --- | --- | --- | --- | --- | --- |
| Tippett | **0.1588** (0.1557-0.1621) | **0.2412** (0.2375-0.2450) | **0.3134** (0.3094-0.3175) | **0.3721** (0.3679-0.3763) | **0.4259** (0.4216-0.4303) | **0.4688** (0.4644-0.4732) | **0.5080** (0.5037-0.5124) | **0.5406** (0.5362-0.5450) | **0.5709** (0.5666-0.5753) | **0.5962** (0.5919-0.6005) | **0.6228** (0.6186-0.6271) | **0.6421** (0.6379-0.6463) | **0.6643** (0.6601-0.6684) | **0.6747** (0.6706-0.6788) | **0.6926** (0.6886-0.6966) | **0.7078** (0.7038-0.7118) |
| Fisher | **0.1305** (0.1276-0.1335) | **0.2580** (0.2542-0.2619) | **0.4136** (0.4093-0.4179) | **0.5606** (0.5562-0.5649) | **0.6852** (0.6811-0.6893) | **0.7850** (0.7813-0.7885) | **0.8538** (0.8507-0.8569) | **0.9038** (0.9011-0.9063) | **0.9364** (0.9342-0.9385) | **0.9584** (0.9567-0.9602) | **0.9709** (0.9693-0.9723) | **0.9816** (0.9804-0.9828) | **0.9881** (0.9871-0.9890) | **0.9911** (0.9902-0.9918) | **0.9938** (0.9931-0.9945) | **0.9956** (0.9950-0.9962) |
| Stouffer | **0.0989** (0.0963-0.1015) | **0.1802** (0.1768-0.1836) | **0.2906** (0.2867-0.2946) | **0.4198** (0.4154-0.4241) | **0.5556** (0.5512-0.5599) | **0.6830** (0.6789-0.6870) | **0.7839** (0.7803-0.7875) | **0.8607** (0.8576-0.8637) | **0.9121** (0.9096-0.9145) | **0.9483** (0.9463-0.9502) | **0.9694** (0.9678-0.9709) | **0.9825** (0.9814-0.9837) | **0.9889** (0.9879-0.9897) | **0.9936** (0.9929-0.9943) | **0.9966** (0.9961-0.9971) | **0.9980** (0.9975-0.9983) |
| Wilkinson | **0.0473** (0.0454-0.0492) | **0.0798** (0.0774-0.0822) | **0.1276** (0.1247-0.1306) | **0.1943** (0.1909-0.1978) | **0.2755** (0.2716-0.2795) | **0.3708** (0.3666-0.3750) | **0.4774** (0.4731-0.4818) | **0.5708** (0.5665-0.5751) | **0.6572** (0.6530-0.6614) | **0.7375** (0.7337-0.7414) | **0.7963** (0.7927-0.7998) | **0.8453** (0.8421-0.8485) | **0.8813** (0.8784-0.8841) | **0.9079** (0.9054-0.9104) | **0.9333** (0.9311-0.9355) | **0.9471** (0.9451-0.9490) |
| Winer | **0.1038** (0.1012-0.1065) | **0.1879** (0.1845-0.1913) | **0.3063** (0.3022-0.3103) | **0.4440** (0.4397-0.4484) | **0.5790** (0.5747-0.5833) | **0.7010** (0.6970-0.7050) | **0.7990** (0.7955-0.8025) | **0.8691** (0.8661-0.8720) | **0.9186** (0.9162-0.9210) | **0.9504** (0.9484-0.9522) | **0.9703** (0.9687-0.9717) | **0.9831** (0.9820-0.9842) | **0.9899** (0.9890-0.9907) | **0.9935** (0.9928-0.9942) | **0.9968** (0.9963-0.9973) | **0.9977** (0.9973-0.9981) |
| Edgington | **0.0794** (0.0771-0.0818) | **0.1277** (0.1248-0.1307) | **0.1917** (0.1882-0.1951) | **0.2741** (0.2702-0.2781) | **0.3789** (0.3746-0.3831) | **0.4917** (0.4873-0.4961) | **0.6046** (0.6003-0.6089) | **0.7122** (0.7082-0.7162) | **0.8071** (0.8036-0.8105) | **0.8769** (0.8740-0.8797) | **0.9254** (0.9231-0.9277) | **0.9574** (0.9556-0.9592) | **0.9753** (0.9739-0.9766) | **0.9873** (0.9863-0.9882) | **0.9931** (0.9923-0.9938) | **0.9963** (0.9957-0.9968) |
| Mudholkar-George | **0.1092** (0.1065-0.1120) | **0.2060** (0.2025-0.2096) | **0.3296** (0.3255-0.3337) | **0.4684** (0.4640-0.4728) | **0.6025** (0.5982-0.6068) | **0.7198** (0.7159-0.7238) | **0.8106** (0.8071-0.8140) | **0.8771** (0.8742-0.8799) | **0.9216** (0.9193-0.9240) | **0.9508** (0.9488-0.9526) | **0.9707** (0.9692-0.9722) | **0.9821** (0.9809-0.9832) | **0.9895** (0.9886-0.9904) | **0.9934** (0.9926-0.9940) | **0.9963** (0.9958-0.9968) | **0.9979** (0.9974-0.9982) |
| Friston | **0.0557** (0.0538-0.0578) | **0.0651** (0.0630-0.0673) | **0.0759** (0.0737-0.0783) | **0.0861** (0.0837-0.0886) | **0.1010** (0.0984-0.1037) | **0.1138** (0.1110-0.1166) | **0.1293** (0.1264-0.1322) | **0.1488** (0.1457-0.1519) | **0.1710** (0.1677-0.1743) | **0.1997** (0.1962-0.2032) | **0.2358** (0.2321-0.2395) | **0.2840** (0.2801-0.2880) | **0.3443** (0.3402-0.3485) | **0.4363** (0.4319-0.4406) | **0.5680** (0.5637-0.5724) | **0.7714** (0.7677-0.7750) |
| Darlington-Hayes | **0.1175** (0.1147-0.1204) | **0.2376** (0.2339-0.2414) | **0.3946** (0.3903-0.3989) | **0.5493** (0.5449-0.5537) | **0.6824** (0.6783-0.6865) | **0.7832** (0.7796-0.7868) | **0.8498** (0.8466-0.8529) | **0.8976** (0.8949-0.9002) | **0.9262** (0.9238-0.9284) | **0.9501** (0.9482-0.9520) | **0.9618** (0.9601-0.9634) | **0.9719** (0.9705-0.9734) | **0.9781** (0.9768-0.9794) | **0.9821** (0.9809-0.9832) | **0.9859** (0.9849-0.9869) | **0.9877** (0.9867-0.9886) |
| Zaykin | **0.1224** (0.1195-0.1253) | **0.2440** (0.2402-0.2478) | **0.3934** (0.3891-0.3977) | **0.5417** (0.5373-0.5460) | **0.6668** (0.6626-0.6709) | **0.7620** (0.7582-0.7657) | **0.8343** (0.8310-0.8375) | **0.8846** (0.8817-0.8873) | **0.9204** (0.9180-0.9228) | **0.9447** (0.9426-0.9466) | **0.9612** (0.9595-0.9629) | **0.9730** (0.9716-0.9744) | **0.9807** (0.9794-0.9819) | **0.9866** (0.9856-0.9876) | **0.9898** (0.9889-0.9907) | **0.9928** (0.9920-0.9935) |
| Dudbridge-Koeleman | **0.1355** (0.1325-0.1385) | **0.2746** (0.2707-0.2786) | **0.4292** (0.4249-0.4335) | **0.5699** (0.5656-0.5742) | **0.6880** (0.6839-0.6920) | **0.7766** (0.7729-0.7802) | **0.8392** (0.8359-0.8424) | **0.8838** (0.8810-0.8866) | **0.9143** (0.9119-0.9168) | **0.9365** (0.9343-0.9386) | **0.9504** (0.9485-0.9523) | **0.9616** (0.9599-0.9633) | **0.9695** (0.9679-0.9709) | **0.9758** (0.9744-0.9771) | **0.9798** (0.9786-0.9810) | **0.9833** (0.9822-0.9844) |
| Dudbridge-Koeleman2 | **0.1222** (0.1193-0.1251) | **0.2433** (0.2396-0.2471) | **0.3949** (0.3906-0.3992) | **0.5413** (0.5370-0.5457) | **0.6684** (0.6643-0.6726) | **0.7660** (0.7623-0.7697) | **0.8331** (0.8298-0.8363) | **0.8857** (0.8829-0.8885) | **0.9197** (0.9173-0.9220) | **0.9444** (0.9424-0.9464) | **0.9603** (0.9585-0.9619) | **0.9699** (0.9683-0.9713) | **0.9764** (0.9750-0.9777) | **0.9809** (0.9797-0.9821) | **0.9836** (0.9824-0.9846) | **0.9873** (0.9863-0.9882) |
| Taylor-Tibshirani | **0.0991** (0.0965-0.1018) | **0.1791** (0.1757-0.1825) | **0.2912** (0.2872-0.2952) | **0.4245** (0.4202-0.4289) | **0.5612** (0.5569-0.5656) | **0.6885** (0.6845-0.6926) | **0.7938** (0.7903-0.7974) | **0.8681** (0.8651-0.8710) | **0.9177** (0.9153-0.9201) | **0.9511** (0.9492-0.9530) | **0.9699** (0.9684-0.9714) | **0.9831** (0.9819-0.9842) | **0.9896** (0.9887-0.9904) | **0.9942** (0.9935-0.9948) | **0.9967** (0.9962-0.9972) | **0.9976** (0.9971-0.9980) |
| Jiang | **0.1055** (0.1029-0.1083) | **0.2035** (0.2000-0.2071) | **0.3354** (0.3313-0.3396) | **0.4845** (0.4801-0.4889) | **0.6153** (0.6110-0.6196) | **0.7221** (0.7182-0.7260) | **0.8070** (0.8035-0.8104) | **0.8611** (0.8580-0.8641) | **0.9042** (0.9016-0.9068) | **0.9318** (0.9296-0.9340) | **0.9530** (0.9511-0.9548) | **0.9664** (0.9648-0.9679) | **0.9758** (0.9744-0.9771) | **0.9822** (0.9810-0.9834) | **0.9879** (0.9869-0.9888) | **0.9908** (0.9899-0.9916) |
| Hotelling | **0.0618** (0.0597-0.0639) | **0.0744** (0.0722-0.0768) | **0.0871** (0.0846-0.0896) | **0.0999** (0.0973-0.1026) | **0.1136** (0.1108-0.1164) | **0.1244** (0.1215-0.1273) | **0.1399** (0.1369-0.1429) | **0.1545** (0.1514-0.1577) | **0.1665** (0.1633-0.1698) | **0.1821** (0.1787-0.1855) | **0.1962** (0.1928-0.1997) | **0.2090** (0.2055-0.2126) | **0.2217** (0.2181-0.2253) | **0.2354** (0.2317-0.2391) | **0.2484** (0.2446-0.2522) | **0.2644** (0.2605-0.2683) |
| Wilks | **0.0610** (0.0589-0.0631) | **0.0736** (0.0713-0.0759) | **0.0875** (0.0851-0.0900) | **0.1025** (0.0999-0.1052) | **0.1149** (0.1121-0.1177) | **0.1278** (0.1249-0.1308) | **0.1433** (0.1403-0.1464) | **0.1587** (0.1555-0.1619) | **0.1704** (0.1671-0.1737) | **0.1845** (0.1812-0.1880) | **0.1993** (0.1958-0.2028) | **0.2142** (0.2107-0.2179) | **0.2296** (0.2260-0.2333) | **0.2449** (0.2412-0.2487) | **0.2548** (0.2510-0.2586) | **0.2698** (0.2659-0.2737) |
