# Supplementary material for: Non‐parametric combination and related permutation tests for neuroimaging
Source: Hum Brain Mapp. 2016 Feb 5;37(4):1486–511. doi: 10.1002/hbm.23115 (PMC4783210; doi:10.1002/hbm.23115)
Supplement: Supplementary file 1 — Supporting Information [file HBM-37-1486-s001.zip › supporting_information/errorrates/histograms/simC_Weibull_ee+ise_uncp.html]

Sim C, Weibull, EE+ISE, uncp

# Simulations C, Weibull, EE+ISE, uncp

---

| Method | ksig=1 / K=16 | ksig=2 / K=16 | ksig=3 / K=16 | ksig=4 / K=16 | ksig=5 / K=16 | ksig=6 / K=16 | ksig=7 / K=16 | ksig=8 / K=16 | ksig=9 / K=16 | ksig=10 / K=16 | ksig=11 / K=16 | ksig=12 / K=16 | ksig=13 / K=16 | ksig=14 / K=16 | ksig=15 / K=16 | ksig=16 / K=16 |
| --- | --- | --- | --- | --- | --- | --- | --- | --- | --- | --- | --- | --- | --- | --- | --- | --- |
| Tippett | **0.5196** (0.5152-0.5240) | **0.7519** (0.7481-0.7557) | **0.8687** (0.8658-0.8717) | **0.9299** (0.9276-0.9321) | **0.9613** (0.9596-0.9630) | **0.9781** (0.9768-0.9794) | **0.9873** (0.9863-0.9882) | **0.9927** (0.9920-0.9934) | **0.9953** (0.9947-0.9959) | **0.9975** (0.9970-0.9979) | **0.9983** (0.9979-0.9986) | **0.9990** (0.9987-0.9992) | **0.9994** (0.9991-0.9995) | **0.9996** (0.9994-0.9998) | **0.9998** (0.9997-0.9999) | **0.9998** (0.9997-0.9999) |
| Fisher | **0.4045** (0.4002-0.4088) | **0.6813** (0.6772-0.6853) | **0.8482** (0.8450-0.8513) | **0.9343** (0.9321-0.9364) | **0.9721** (0.9706-0.9735) | **0.9888** (0.9879-0.9897) | **0.9959** (0.9953-0.9964) | **0.9983** (0.9979-0.9987) | **0.9993** (0.9990-0.9995) | **0.9998** (0.9997-0.9999) | **0.9999** (0.9998-1.0000) | **1.0000** (0.9999-1.0000) | **1.0000** (0.9999-1.0000) | **1.0000** (0.9999-1.0000) | **1.0000** (0.9999-1.0000) | **1.0000** (0.9999-1.0000) |
| Stouffer | **0.1990** (0.1955-0.2025) | **0.4219** (0.4176-0.4263) | **0.6392** (0.6350-0.6434) | **0.8081** (0.8046-0.8115) | **0.9082** (0.9056-0.9107) | **0.9603** (0.9586-0.9620) | **0.9842** (0.9831-0.9853) | **0.9937** (0.9930-0.9944) | **0.9977** (0.9972-0.9981) | **0.9993** (0.9991-0.9995) | **0.9997** (0.9995-0.9998) | **0.9999** (0.9998-1.0000) | **1.0000** (0.9999-1.0000) | **1.0000** (0.9999-1.0000) | **1.0000** (0.9999-1.0000) | **1.0000** (0.9999-1.0000) |
| Wilkinson | **0.0536** (0.0516-0.0556) | **0.0977** (0.0951-0.1003) | **0.1636** (0.1604-0.1669) | **0.2530** (0.2492-0.2569) | **0.3645** (0.3603-0.3687) | **0.4902** (0.4859-0.4946) | **0.6106** (0.6064-0.6149) | **0.7262** (0.7223-0.7301) | **0.8200** (0.8166-0.8233) | **0.8827** (0.8798-0.8855) | **0.9328** (0.9306-0.9350) | **0.9619** (0.9602-0.9636) | **0.9775** (0.9762-0.9788) | **0.9873** (0.9862-0.9882) | **0.9931** (0.9923-0.9938) | **0.9965** (0.9959-0.9969) |
| Winer | **0.2787** (0.2748-0.2827) | **0.5276** (0.5233-0.5320) | **0.7295** (0.7256-0.7334) | **0.8634** (0.8603-0.8663) | **0.9366** (0.9345-0.9387) | **0.9727** (0.9713-0.9741) | **0.9893** (0.9883-0.9901) | **0.9960** (0.9954-0.9965) | **0.9985** (0.9981-0.9988) | **0.9996** (0.9994-0.9997) | **0.9999** (0.9997-0.9999) | **0.9999** (0.9998-1.0000) | **1.0000** (0.9999-1.0000) | **1.0000** (0.9999-1.0000) | **1.0000** (0.9999-1.0000) | **1.0000** (0.9999-1.0000) |
| Edgington | **0.0860** (0.0836-0.0885) | **0.1413** (0.1383-0.1444) | **0.2182** (0.2146-0.2219) | **0.3161** (0.3121-0.3202) | **0.4353** (0.4310-0.4397) | **0.5634** (0.5591-0.5678) | **0.6929** (0.6888-0.6969) | **0.8034** (0.7999-0.8069) | **0.8874** (0.8846-0.8902) | **0.9437** (0.9417-0.9457) | **0.9754** (0.9740-0.9767) | **0.9902** (0.9893-0.9910) | **0.9960** (0.9954-0.9965) | **0.9986** (0.9982-0.9989) | **0.9995** (0.9992-0.9996) | **0.9998** (0.9996-0.9999) |
| Mudholkar-George | **0.3101** (0.3060-0.3141) | **0.5709** (0.5666-0.5753) | **0.7669** (0.7631-0.7705) | **0.8863** (0.8835-0.8891) | **0.9484** (0.9464-0.9503) | **0.9783** (0.9770-0.9795) | **0.9915** (0.9906-0.9922) | **0.9969** (0.9964-0.9974) | **0.9988** (0.9984-0.9990) | **0.9997** (0.9995-0.9998) | **0.9999** (0.9998-1.0000) | **0.9999** (0.9998-1.0000) | **1.0000** (0.9999-1.0000) | **1.0000** (0.9999-1.0000) | **1.0000** (0.9999-1.0000) | **1.0000** (0.9999-1.0000) |
| Friston | **0.0586** (0.0565-0.0607) | **0.0673** (0.0652-0.0696) | **0.0745** (0.0722-0.0768) | **0.0837** (0.0813-0.0862) | **0.0951** (0.0926-0.0977) | **0.1068** (0.1041-0.1095) | **0.1201** (0.1173-0.1230) | **0.1351** (0.1321-0.1381) | **0.1557** (0.1526-0.1589) | **0.1799** (0.1766-0.1833) | **0.2113** (0.2078-0.2149) | **0.2504** (0.2466-0.2542) | **0.3054** (0.3014-0.3095) | **0.3808** (0.3765-0.3850) | **0.4965** (0.4921-0.5008) | **0.6716** (0.6675-0.6757) |
| Darlington-Hayes | **0.3122** (0.3082-0.3163) | **0.6007** (0.5964-0.6050) | **0.8040** (0.8005-0.8074) | **0.9132** (0.9107-0.9157) | **0.9642** (0.9626-0.9658) | **0.9859** (0.9848-0.9869) | **0.9945** (0.9938-0.9951) | **0.9979** (0.9974-0.9982) | **0.9991** (0.9988-0.9993) | **0.9997** (0.9995-0.9998) | **0.9999** (0.9997-0.9999) | **1.0000** (0.9999-1.0000) | **1.0000** (0.9999-1.0000) | **1.0000** (0.9999-1.0000) | **1.0000** (0.9999-1.0000) | **1.0000** (0.9999-1.0000) |
| Zaykin | **0.3818** (0.3776-0.3861) | **0.6595** (0.6553-0.6636) | **0.8335** (0.8302-0.8367) | **0.9252** (0.9228-0.9275) | **0.9686** (0.9670-0.9701) | **0.9863** (0.9852-0.9872) | **0.9943** (0.9936-0.9949) | **0.9978** (0.9974-0.9982) | **0.9991** (0.9988-0.9993) | **0.9997** (0.9996-0.9998) | **0.9999** (0.9998-1.0000) | **0.9999** (0.9998-1.0000) | **1.0000** (0.9999-1.0000) | **1.0000** (0.9999-1.0000) | **1.0000** (0.9999-1.0000) | **1.0000** (0.9999-1.0000) |
| Dudbridge-Koeleman | **0.4305** (0.4261-0.4348) | **0.7071** (0.7031-0.7111) | **0.8630** (0.8599-0.8660) | **0.9412** (0.9391-0.9433) | **0.9750** (0.9736-0.9763) | **0.9896** (0.9887-0.9905) | **0.9958** (0.9952-0.9963) | **0.9984** (0.9980-0.9987) | **0.9993** (0.9990-0.9995) | **0.9997** (0.9996-0.9998) | **0.9999** (0.9998-1.0000) | **1.0000** (0.9999-1.0000) | **1.0000** (0.9999-1.0000) | **1.0000** (0.9999-1.0000) | **1.0000** (0.9999-1.0000) | **1.0000** (0.9999-1.0000) |
| Dudbridge-Koeleman2 | **0.3816** (0.3774-0.3859) | **0.6594** (0.6553-0.6636) | **0.8332** (0.8299-0.8364) | **0.9254** (0.9231-0.9277) | **0.9678** (0.9662-0.9693) | **0.9864** (0.9854-0.9874) | **0.9944** (0.9937-0.9950) | **0.9979** (0.9974-0.9982) | **0.9992** (0.9989-0.9994) | **0.9997** (0.9996-0.9998) | **0.9999** (0.9998-1.0000) | **1.0000** (0.9999-1.0000) | **1.0000** (0.9999-1.0000) | **1.0000** (0.9999-1.0000) | **1.0000** (0.9999-1.0000) | **1.0000** (0.9999-1.0000) |
| Taylor-Tibshirani | **0.1301** (0.1271-0.1330) | **0.2549** (0.2511-0.2587) | **0.4220** (0.4177-0.4264) | **0.5997** (0.5954-0.6039) | **0.7587** (0.7549-0.7624) | **0.8686** (0.8656-0.8715) | **0.9367** (0.9345-0.9388) | **0.9739** (0.9725-0.9753) | **0.9905** (0.9896-0.9913) | **0.9967** (0.9962-0.9972) | **0.9986** (0.9982-0.9989) | **0.9996** (0.9994-0.9997) | **0.9999** (0.9998-1.0000) | **1.0000** (0.9999-1.0000) | **1.0000** (0.9999-1.0000) | **1.0000** (0.9999-1.0000) |
| Jiang | **0.1543** (0.1512-0.1575) | **0.3320** (0.3279-0.3361) | **0.5524** (0.5481-0.5568) | **0.7458** (0.7420-0.7496) | **0.8716** (0.8687-0.8745) | **0.9417** (0.9396-0.9437) | **0.9744** (0.9730-0.9758) | **0.9898** (0.9889-0.9906) | **0.9958** (0.9952-0.9963) | **0.9984** (0.9980-0.9987) | **0.9992** (0.9989-0.9994) | **0.9997** (0.9995-0.9998) | **0.9999** (0.9998-1.0000) | **1.0000** (0.9999-1.0000) | **1.0000** (0.9999-1.0000) | **1.0000** (0.9999-1.0000) |
| Hotelling | **0.2290** (0.2253-0.2327) | **0.3732** (0.3689-0.3774) | **0.4860** (0.4816-0.4904) | **0.5794** (0.5751-0.5837) | **0.6571** (0.6529-0.6612) | **0.7188** (0.7148-0.7227) | **0.7604** (0.7567-0.7641) | **0.8023** (0.7988-0.8057) | **0.8307** (0.8274-0.8340) | **0.8582** (0.8551-0.8612) | **0.8771** (0.8742-0.8800) | **0.8946** (0.8919-0.8972) | **0.9100** (0.9075-0.9125) | **0.9223** (0.9200-0.9247) | **0.9330** (0.9308-0.9352) | **0.9397** (0.9376-0.9418) |
| Wilks | **0.2285** (0.2249-0.2322) | **0.3717** (0.3675-0.3759) | **0.4866** (0.4822-0.4910) | **0.5824** (0.5781-0.5868) | **0.6587** (0.6545-0.6628) | **0.7177** (0.7137-0.7216) | **0.7634** (0.7596-0.7671) | **0.8006** (0.7971-0.8041) | **0.8332** (0.8299-0.8364) | **0.8578** (0.8548-0.8609) | **0.8793** (0.8764-0.8821) | **0.8953** (0.8925-0.8979) | **0.9091** (0.9065-0.9116) | **0.9229** (0.9206-0.9252) | **0.9327** (0.9305-0.9348) | **0.9398** (0.9377-0.9419) |
