# Supplementary material for: Non‐parametric combination and related permutation tests for neuroimaging
Source: Hum Brain Mapp. 2016 Feb 5;37(4):1486–511. doi: 10.1002/hbm.23115 (PMC4783210; doi:10.1002/hbm.23115)
Supplement: Supplementary file 1 — Supporting Information [file HBM-37-1486-s001.zip › supporting_information/errorrates/histograms/simC_Weibull_ee_uncp.html]

Sim C, Weibull, EE, uncp

# Simulations C, Weibull, EE, uncp

---

| Method | ksig=1 / K=16 | ksig=2 / K=16 | ksig=3 / K=16 | ksig=4 / K=16 | ksig=5 / K=16 | ksig=6 / K=16 | ksig=7 / K=16 | ksig=8 / K=16 | ksig=9 / K=16 | ksig=10 / K=16 | ksig=11 / K=16 | ksig=12 / K=16 | ksig=13 / K=16 | ksig=14 / K=16 | ksig=15 / K=16 | ksig=16 / K=16 |
| --- | --- | --- | --- | --- | --- | --- | --- | --- | --- | --- | --- | --- | --- | --- | --- | --- |
| Tippett | **0.5409** (0.5365-0.5453) | **0.7678** (0.7641-0.7715) | **0.8794** (0.8765-0.8822) | **0.9345** (0.9323-0.9367) | **0.9642** (0.9625-0.9658) | **0.9797** (0.9784-0.9809) | **0.9878** (0.9868-0.9888) | **0.9927** (0.9919-0.9934) | **0.9955** (0.9949-0.9961) | **0.9977** (0.9972-0.9981) | **0.9984** (0.9980-0.9987) | **0.9989** (0.9986-0.9992) | **0.9993** (0.9991-0.9995) | **0.9996** (0.9994-0.9997) | **0.9997** (0.9995-0.9998) | **0.9998** (0.9997-0.9999) |
| Fisher | **0.4091** (0.4048-0.4134) | **0.6857** (0.6816-0.6898) | **0.8494** (0.8462-0.8525) | **0.9333** (0.9311-0.9355) | **0.9720** (0.9705-0.9734) | **0.9884** (0.9874-0.9893) | **0.9949** (0.9942-0.9954) | **0.9982** (0.9978-0.9986) | **0.9993** (0.9990-0.9995) | **0.9997** (0.9995-0.9998) | **0.9999** (0.9998-1.0000) | **1.0000** (0.9999-1.0000) | **1.0000** (0.9999-1.0000) | **1.0000** (0.9999-1.0000) | **1.0000** (0.9999-1.0000) | **1.0000** (0.9999-1.0000) |
| Stouffer | **0.1942** (0.1908-0.1977) | **0.4149** (0.4106-0.4192) | **0.6364** (0.6322-0.6406) | **0.8036** (0.8001-0.8070) | **0.9062** (0.9036-0.9087) | **0.9580** (0.9562-0.9597) | **0.9835** (0.9824-0.9846) | **0.9937** (0.9929-0.9943) | **0.9975** (0.9970-0.9979) | **0.9993** (0.9990-0.9995) | **0.9997** (0.9996-0.9998) | **0.9999** (0.9998-1.0000) | **1.0000** (0.9999-1.0000) | **1.0000** (0.9999-1.0000) | **1.0000** (0.9999-1.0000) | **1.0000** (0.9999-1.0000) |
| Wilkinson | **0.0560** (0.0540-0.0580) | **0.1012** (0.0986-0.1039) | **0.1678** (0.1646-0.1711) | **0.2557** (0.2519-0.2595) | **0.3702** (0.3660-0.3744) | **0.4930** (0.4887-0.4974) | **0.6136** (0.6093-0.6179) | **0.7269** (0.7230-0.7308) | **0.8117** (0.8083-0.8151) | **0.8844** (0.8816-0.8872) | **0.9295** (0.9272-0.9317) | **0.9599** (0.9581-0.9616) | **0.9776** (0.9763-0.9789) | **0.9864** (0.9853-0.9873) | **0.9933** (0.9926-0.9940) | **0.9966** (0.9960-0.9971) |
| Winer | **0.2707** (0.2668-0.2746) | **0.5188** (0.5144-0.5232) | **0.7251** (0.7211-0.7290) | **0.8596** (0.8565-0.8626) | **0.9341** (0.9319-0.9362) | **0.9715** (0.9700-0.9729) | **0.9883** (0.9874-0.9892) | **0.9955** (0.9949-0.9961) | **0.9984** (0.9980-0.9987) | **0.9994** (0.9991-0.9996) | **0.9998** (0.9997-0.9999) | **0.9999** (0.9998-1.0000) | **1.0000** (0.9999-1.0000) | **1.0000** (0.9999-1.0000) | **1.0000** (0.9999-1.0000) | **1.0000** (0.9999-1.0000) |
| Edgington | **0.0873** (0.0848-0.0898) | **0.1439** (0.1409-0.1470) | **0.2223** (0.2187-0.2260) | **0.3248** (0.3207-0.3289) | **0.4418** (0.4374-0.4461) | **0.5709** (0.5665-0.5752) | **0.6946** (0.6905-0.6986) | **0.8049** (0.8014-0.8084) | **0.8872** (0.8844-0.8900) | **0.9421** (0.9401-0.9442) | **0.9754** (0.9740-0.9768) | **0.9895** (0.9886-0.9904) | **0.9962** (0.9956-0.9967) | **0.9987** (0.9984-0.9990) | **0.9995** (0.9992-0.9996) | **0.9998** (0.9997-0.9999) |
| Mudholkar-George | **0.3032** (0.2992-0.3073) | **0.5627** (0.5583-0.5670) | **0.7619** (0.7582-0.7657) | **0.8818** (0.8790-0.8846) | **0.9472** (0.9452-0.9491) | **0.9769** (0.9756-0.9782) | **0.9905** (0.9897-0.9914) | **0.9964** (0.9959-0.9969) | **0.9987** (0.9983-0.9989) | **0.9996** (0.9994-0.9997) | **0.9998** (0.9997-0.9999) | **1.0000** (0.9999-1.0000) | **1.0000** (0.9999-1.0000) | **1.0000** (0.9999-1.0000) | **1.0000** (0.9999-1.0000) | **1.0000** (0.9999-1.0000) |
| Friston | **0.0548** (0.0528-0.0568) | **0.0642** (0.0621-0.0664) | **0.0728** (0.0705-0.0751) | **0.0817** (0.0794-0.0842) | **0.0927** (0.0902-0.0953) | **0.1060** (0.1033-0.1087) | **0.1203** (0.1174-0.1231) | **0.1377** (0.1347-0.1407) | **0.1559** (0.1528-0.1591) | **0.1779** (0.1746-0.1813) | **0.2098** (0.2062-0.2134) | **0.2491** (0.2454-0.2530) | **0.3038** (0.2998-0.3079) | **0.3778** (0.3736-0.3821) | **0.4856** (0.4812-0.4900) | **0.6572** (0.6530-0.6613) |
| Darlington-Hayes | **0.3209** (0.3168-0.3250) | **0.6084** (0.6042-0.6127) | **0.8072** (0.8037-0.8106) | **0.9141** (0.9116-0.9165) | **0.9646** (0.9630-0.9662) | **0.9852** (0.9841-0.9863) | **0.9938** (0.9931-0.9945) | **0.9979** (0.9975-0.9983) | **0.9992** (0.9989-0.9994) | **0.9996** (0.9994-0.9997) | **0.9999** (0.9997-0.9999) | **0.9999** (0.9998-1.0000) | **1.0000** (0.9999-1.0000) | **1.0000** (0.9999-1.0000) | **1.0000** (0.9999-1.0000) | **1.0000** (0.9999-1.0000) |
| Zaykin | **0.3861** (0.3818-0.3903) | **0.6650** (0.6609-0.6692) | **0.8366** (0.8333-0.8398) | **0.9249** (0.9226-0.9272) | **0.9680** (0.9665-0.9695) | **0.9858** (0.9847-0.9868) | **0.9939** (0.9932-0.9945) | **0.9975** (0.9970-0.9979) | **0.9991** (0.9988-0.9993) | **0.9997** (0.9995-0.9998) | **0.9999** (0.9997-0.9999) | **1.0000** (0.9999-1.0000) | **1.0000** (0.9999-1.0000) | **1.0000** (0.9999-1.0000) | **1.0000** (0.9999-1.0000) | **1.0000** (0.9999-1.0000) |
| Dudbridge-Koeleman | **0.4440** (0.4396-0.4483) | **0.7153** (0.7113-0.7192) | **0.8676** (0.8646-0.8706) | **0.9409** (0.9388-0.9430) | **0.9749** (0.9735-0.9762) | **0.9890** (0.9880-0.9898) | **0.9947** (0.9940-0.9953) | **0.9981** (0.9976-0.9984) | **0.9992** (0.9989-0.9994) | **0.9997** (0.9995-0.9998) | **0.9998** (0.9997-0.9999) | **1.0000** (0.9999-1.0000) | **1.0000** (0.9999-1.0000) | **1.0000** (0.9999-1.0000) | **1.0000** (0.9999-1.0000) | **1.0000** (0.9999-1.0000) |
| Dudbridge-Koeleman2 | **0.3865** (0.3822-0.3908) | **0.6646** (0.6604-0.6687) | **0.8358** (0.8325-0.8390) | **0.9257** (0.9233-0.9279) | **0.9684** (0.9668-0.9699) | **0.9860** (0.9849-0.9870) | **0.9938** (0.9931-0.9945) | **0.9976** (0.9972-0.9980) | **0.9991** (0.9988-0.9993) | **0.9996** (0.9994-0.9998) | **0.9999** (0.9997-0.9999) | **1.0000** (0.9999-1.0000) | **1.0000** (0.9999-1.0000) | **1.0000** (0.9999-1.0000) | **1.0000** (0.9999-1.0000) | **1.0000** (0.9999-1.0000) |
| Taylor-Tibshirani | **0.1307** (0.1278-0.1337) | **0.2639** (0.2600-0.2677) | **0.4308** (0.4265-0.4352) | **0.6099** (0.6056-0.6142) | **0.7629** (0.7591-0.7666) | **0.8750** (0.8721-0.8779) | **0.9418** (0.9397-0.9438) | **0.9770** (0.9756-0.9783) | **0.9911** (0.9902-0.9918) | **0.9968** (0.9963-0.9973) | **0.9989** (0.9985-0.9991) | **0.9996** (0.9994-0.9998) | **0.9999** (0.9998-1.0000) | **1.0000** (0.9999-1.0000) | **1.0000** (0.9999-1.0000) | **1.0000** (0.9999-1.0000) |
| Jiang | **0.1632** (0.1600-0.1665) | **0.3608** (0.3566-0.3651) | **0.5778** (0.5734-0.5821) | **0.7634** (0.7596-0.7671) | **0.8821** (0.8792-0.8849) | **0.9445** (0.9425-0.9465) | **0.9748** (0.9734-0.9761) | **0.9890** (0.9881-0.9899) | **0.9955** (0.9948-0.9960) | **0.9983** (0.9979-0.9986) | **0.9993** (0.9990-0.9995) | **0.9997** (0.9995-0.9998) | **1.0000** (0.9999-1.0000) | **0.9999** (0.9998-1.0000) | **1.0000** (0.9999-1.0000) | **1.0000** (0.9999-1.0000) |
| Hotelling | **0.2233** (0.2197-0.2270) | **0.3644** (0.3602-0.3686) | **0.4760** (0.4716-0.4804) | **0.5712** (0.5669-0.5755) | **0.6444** (0.6402-0.6486) | **0.7055** (0.7015-0.7095) | **0.7542** (0.7504-0.7580) | **0.7934** (0.7899-0.7970) | **0.8231** (0.8198-0.8265) | **0.8504** (0.8472-0.8535) | **0.8711** (0.8681-0.8740) | **0.8902** (0.8874-0.8929) | **0.9057** (0.9031-0.9082) | **0.9167** (0.9142-0.9191) | **0.9253** (0.9230-0.9276) | **0.9342** (0.9320-0.9363) |
| Wilks | **0.2232** (0.2196-0.2269) | **0.3630** (0.3588-0.3672) | **0.4765** (0.4722-0.4809) | **0.5700** (0.5656-0.5743) | **0.6446** (0.6404-0.6488) | **0.7062** (0.7022-0.7101) | **0.7534** (0.7496-0.7572) | **0.7928** (0.7892-0.7963) | **0.8251** (0.8217-0.8284) | **0.8500** (0.8468-0.8531) | **0.8723** (0.8694-0.8752) | **0.8898** (0.8870-0.8925) | **0.9054** (0.9028-0.9079) | **0.9171** (0.9147-0.9195) | **0.9258** (0.9234-0.9280) | **0.9345** (0.9323-0.9366) |
