# Supplementary material for: Non‐parametric combination and related permutation tests for neuroimaging
Source: Hum Brain Mapp. 2016 Feb 5;37(4):1486–511. doi: 10.1002/hbm.23115 (PMC4783210; doi:10.1002/hbm.23115)
Supplement: Supplementary file 1 — Supporting Information [file HBM-37-1486-s001.zip › supporting_information/errorrates/histograms/simC_Weibull_ise_uncp.html]

Sim C, Weibull, ISE, uncp

# Simulations C, Weibull, ISE, uncp

---

| Method | ksig=1 / K=16 | ksig=2 / K=16 | ksig=3 / K=16 | ksig=4 / K=16 | ksig=5 / K=16 | ksig=6 / K=16 | ksig=7 / K=16 | ksig=8 / K=16 | ksig=9 / K=16 | ksig=10 / K=16 | ksig=11 / K=16 | ksig=12 / K=16 | ksig=13 / K=16 | ksig=14 / K=16 | ksig=15 / K=16 | ksig=16 / K=16 |
| --- | --- | --- | --- | --- | --- | --- | --- | --- | --- | --- | --- | --- | --- | --- | --- | --- |
| Tippett | **0.5086** (0.5042-0.5129) | **0.7402** (0.7364-0.7441) | **0.8518** (0.8486-0.8549) | **0.9133** (0.9108-0.9157) | **0.9478** (0.9458-0.9497) | **0.9659** (0.9643-0.9675) | **0.9789** (0.9776-0.9801) | **0.9853** (0.9842-0.9863) | **0.9905** (0.9896-0.9913) | **0.9936** (0.9928-0.9942) | **0.9953** (0.9946-0.9958) | **0.9965** (0.9959-0.9969) | **0.9977** (0.9972-0.9981) | **0.9982** (0.9978-0.9986) | **0.9986** (0.9983-0.9989) | **0.9988** (0.9985-0.9991) |
| Fisher | **0.4083** (0.4040-0.4126) | **0.6809** (0.6768-0.6850) | **0.8411** (0.8378-0.8443) | **0.9253** (0.9230-0.9276) | **0.9646** (0.9629-0.9661) | **0.9833** (0.9821-0.9844) | **0.9923** (0.9915-0.9930) | **0.9965** (0.9959-0.9969) | **0.9984** (0.9980-0.9987) | **0.9993** (0.9990-0.9995) | **0.9996** (0.9994-0.9997) | **0.9998** (0.9996-0.9999) | **0.9999** (0.9997-0.9999) | **0.9999** (0.9998-1.0000) | **0.9999** (0.9998-1.0000) | **1.0000** (0.9999-1.0000) |
| Stouffer | **0.1891** (0.1857-0.1926) | **0.4006** (0.3963-0.4049) | **0.6100** (0.6057-0.6143) | **0.7736** (0.7699-0.7772) | **0.8808** (0.8779-0.8836) | **0.9390** (0.9368-0.9410) | **0.9702** (0.9686-0.9716) | **0.9859** (0.9849-0.9869) | **0.9933** (0.9925-0.9940) | **0.9969** (0.9964-0.9974) | **0.9986** (0.9982-0.9989) | **0.9995** (0.9993-0.9997) | **0.9997** (0.9995-0.9998) | **0.9999** (0.9998-1.0000) | **0.9999** (0.9998-1.0000) | **0.9999** (0.9998-1.0000) |
| Wilkinson | **0.0427** (0.0410-0.0445) | **0.0707** (0.0685-0.0730) | **0.1134** (0.1107-0.1162) | **0.1637** (0.1604-0.1669) | **0.2216** (0.2180-0.2253) | **0.2865** (0.2826-0.2905) | **0.3506** (0.3464-0.3548) | **0.4221** (0.4178-0.4265) | **0.4908** (0.4864-0.4952) | **0.5499** (0.5455-0.5543) | **0.6121** (0.6079-0.6164) | **0.6520** (0.6478-0.6561) | **0.7133** (0.7093-0.7173) | **0.7524** (0.7486-0.7562) | **0.7962** (0.7927-0.7997) | **0.8363** (0.8331-0.8395) |
| Winer | **0.2698** (0.2659-0.2737) | **0.5119** (0.5075-0.5163) | **0.7071** (0.7031-0.7111) | **0.8422** (0.8390-0.8454) | **0.9194** (0.9170-0.9217) | **0.9595** (0.9577-0.9612) | **0.9807** (0.9794-0.9819) | **0.9911** (0.9902-0.9918) | **0.9959** (0.9953-0.9964) | **0.9981** (0.9977-0.9985) | **0.9992** (0.9989-0.9994) | **0.9996** (0.9994-0.9998) | **0.9998** (0.9996-0.9999) | **0.9999** (0.9998-1.0000) | **0.9999** (0.9998-1.0000) | **1.0000** (0.9999-1.0000) |
| Edgington | **0.0819** (0.0795-0.0844) | **0.1221** (0.1193-0.1250) | **0.1783** (0.1750-0.1817) | **0.2459** (0.2421-0.2497) | **0.3233** (0.3192-0.3274) | **0.4071** (0.4028-0.4115) | **0.5003** (0.4959-0.5047) | **0.5916** (0.5873-0.5959) | **0.6815** (0.6774-0.6855) | **0.7601** (0.7564-0.7639) | **0.8275** (0.8241-0.8307) | **0.8821** (0.8793-0.8849) | **0.9264** (0.9241-0.9287) | **0.9556** (0.9538-0.9574) | **0.9737** (0.9723-0.9751) | **0.9875** (0.9865-0.9885) |
| Mudholkar-George | **0.3031** (0.2991-0.3071) | **0.5598** (0.5554-0.5641) | **0.7497** (0.7458-0.7534) | **0.8696** (0.8667-0.8726) | **0.9352** (0.9330-0.9374) | **0.9681** (0.9665-0.9696) | **0.9850** (0.9839-0.9860) | **0.9933** (0.9925-0.9940) | **0.9970** (0.9965-0.9974) | **0.9985** (0.9982-0.9988) | **0.9993** (0.9991-0.9995) | **0.9997** (0.9995-0.9998) | **0.9999** (0.9997-0.9999) | **0.9999** (0.9998-1.0000) | **0.9999** (0.9998-1.0000) | **1.0000** (0.9999-1.0000) |
| Friston | **0.0502** (0.0484-0.0522) | **0.0555** (0.0535-0.0576) | **0.0629** (0.0608-0.0650) | **0.0701** (0.0679-0.0723) | **0.0784** (0.0761-0.0808) | **0.0870** (0.0845-0.0895) | **0.0984** (0.0958-0.1011) | **0.1113** (0.1086-0.1141) | **0.1267** (0.1238-0.1296) | **0.1456** (0.1425-0.1487) | **0.1714** (0.1681-0.1747) | **0.2071** (0.2036-0.2107) | **0.2531** (0.2493-0.2569) | **0.3204** (0.3163-0.3245) | **0.4266** (0.4223-0.4310) | **0.6010** (0.5967-0.6053) |
| Darlington-Hayes | **0.3256** (0.3215-0.3297) | **0.6120** (0.6077-0.6163) | **0.8052** (0.8017-0.8086) | **0.9091** (0.9065-0.9116) | **0.9586** (0.9568-0.9603) | **0.9808** (0.9795-0.9819) | **0.9917** (0.9908-0.9924) | **0.9961** (0.9955-0.9966) | **0.9981** (0.9977-0.9985) | **0.9992** (0.9989-0.9994) | **0.9996** (0.9993-0.9997) | **0.9998** (0.9996-0.9999) | **0.9999** (0.9997-0.9999) | **0.9999** (0.9998-1.0000) | **0.9999** (0.9998-1.0000) | **1.0000** (0.9999-1.0000) |
| Zaykin | **0.3877** (0.3834-0.3919) | **0.6648** (0.6606-0.6689) | **0.8304** (0.8270-0.8336) | **0.9205** (0.9181-0.9229) | **0.9620** (0.9603-0.9637) | **0.9819** (0.9807-0.9831) | **0.9919** (0.9911-0.9927) | **0.9957** (0.9951-0.9962) | **0.9982** (0.9978-0.9985) | **0.9992** (0.9989-0.9994) | **0.9995** (0.9992-0.9996) | **0.9998** (0.9997-0.9999) | **0.9999** (0.9998-1.0000) | **0.9999** (0.9998-1.0000) | **1.0000** (0.9999-1.0000) | **1.0000** (0.9999-1.0000) |
| Dudbridge-Koeleman | **0.4409** (0.4365-0.4452) | **0.7147** (0.7107-0.7186) | **0.8629** (0.8599-0.8659) | **0.9367** (0.9345-0.9388) | **0.9696** (0.9681-0.9711) | **0.9853** (0.9842-0.9863) | **0.9931** (0.9923-0.9938) | **0.9963** (0.9957-0.9968) | **0.9984** (0.9980-0.9987) | **0.9992** (0.9989-0.9994) | **0.9995** (0.9992-0.9996) | **0.9997** (0.9996-0.9998) | **0.9999** (0.9997-0.9999) | **0.9999** (0.9998-1.0000) | **0.9999** (0.9998-1.0000) | **0.9999** (0.9998-1.0000) |
| Dudbridge-Koeleman2 | **0.3895** (0.3852-0.3937) | **0.6659** (0.6617-0.6700) | **0.8329** (0.8296-0.8361) | **0.9211** (0.9187-0.9234) | **0.9621** (0.9603-0.9637) | **0.9819** (0.9807-0.9831) | **0.9918** (0.9910-0.9926) | **0.9957** (0.9951-0.9962) | **0.9983** (0.9979-0.9987) | **0.9992** (0.9989-0.9994) | **0.9994** (0.9992-0.9996) | **0.9997** (0.9996-0.9998) | **0.9998** (0.9997-0.9999) | **0.9999** (0.9998-1.0000) | **0.9999** (0.9998-1.0000) | **1.0000** (0.9999-1.0000) |
| Taylor-Tibshirani | **0.1178** (0.1150-0.1206) | **0.2204** (0.2168-0.2240) | **0.3480** (0.3438-0.3522) | **0.4725** (0.4682-0.4769) | **0.5820** (0.5777-0.5863) | **0.6853** (0.6812-0.6893) | **0.7698** (0.7660-0.7734) | **0.8328** (0.8295-0.8360) | **0.8785** (0.8756-0.8814) | **0.9162** (0.9138-0.9186) | **0.9452** (0.9432-0.9472) | **0.9621** (0.9604-0.9637) | **0.9773** (0.9760-0.9786) | **0.9856** (0.9845-0.9866) | **0.9923** (0.9915-0.9930) | **0.9954** (0.9948-0.9960) |
| Jiang | **0.1432** (0.1401-0.1463) | **0.3100** (0.3059-0.3140) | **0.4883** (0.4839-0.4927) | **0.6346** (0.6303-0.6388) | **0.7453** (0.7415-0.7491) | **0.8119** (0.8085-0.8153) | **0.8609** (0.8578-0.8639) | **0.8961** (0.8934-0.8987) | **0.9183** (0.9158-0.9206) | **0.9408** (0.9387-0.9428) | **0.9550** (0.9532-0.9568) | **0.9625** (0.9608-0.9641) | **0.9719** (0.9704-0.9733) | **0.9791** (0.9779-0.9804) | **0.9818** (0.9806-0.9830) | **0.9863** (0.9853-0.9873) |
| Hotelling | **0.1929** (0.1895-0.1964) | **0.3211** (0.3170-0.3252) | **0.4271** (0.4228-0.4315) | **0.5161** (0.5118-0.5205) | **0.5867** (0.5824-0.5910) | **0.6447** (0.6405-0.6489) | **0.6902** (0.6861-0.6942) | **0.7300** (0.7261-0.7339) | **0.7627** (0.7589-0.7664) | **0.7901** (0.7865-0.7937) | **0.8122** (0.8088-0.8156) | **0.8301** (0.8268-0.8333) | **0.8462** (0.8431-0.8494) | **0.8578** (0.8548-0.8609) | **0.8708** (0.8678-0.8737) | **0.8816** (0.8787-0.8844) |
| Wilks | **0.1924** (0.1890-0.1959) | **0.3223** (0.3182-0.3264) | **0.4277** (0.4234-0.4320) | **0.5171** (0.5127-0.5215) | **0.5843** (0.5800-0.5886) | **0.6429** (0.6387-0.6471) | **0.6934** (0.6893-0.6974) | **0.7314** (0.7275-0.7352) | **0.7641** (0.7604-0.7678) | **0.7890** (0.7854-0.7925) | **0.8111** (0.8077-0.8145) | **0.8293** (0.8260-0.8326) | **0.8460** (0.8428-0.8491) | **0.8596** (0.8565-0.8626) | **0.8702** (0.8672-0.8731) | **0.8816** (0.8788-0.8844) |
