# Supplementary material for: Non‐parametric combination and related permutation tests for neuroimaging
Source: Hum Brain Mapp. 2016 Feb 5;37(4):1486–511. doi: 10.1002/hbm.23115 (PMC4783210; doi:10.1002/hbm.23115)
Supplement: Supplementary file 1 — Supporting Information [file HBM-37-1486-s001.zip › supporting_information/errorrates/histograms/simD_Gauss_ee+ise_uncp.html]

Sim D, Gauss, EE+ISE, uncp

# Simulations D, Gauss, EE+ISE, uncp

---

| Method | ksig=2 / K=2 | ksig=3 / K=3 | ksig=4 / K=4 | ksig=5 / K=5 | ksig=6 / K=6 | ksig=7 / K=7 | ksig=8 / K=8 | ksig=9 / K=9 | ksig=10 / K=10 | ksig=11 / K=11 | ksig=12 / K=12 | ksig=13 / K=13 | ksig=14 / K=14 | ksig=15 / K=15 | ksig=16 / K=16 |
| --- | --- | --- | --- | --- | --- | --- | --- | --- | --- | --- | --- | --- | --- | --- | --- |
| Tippett | **0.5758** (0.5714-0.5801) | **0.6335** (0.6293-0.6377) | **0.6722** (0.6681-0.6763) | **0.7008** (0.6968-0.7048) | **0.7237** (0.7198-0.7276) | **0.7435** (0.7397-0.7473) | **0.7559** (0.7522-0.7597) | **0.7721** (0.7684-0.7758) | **0.7809** (0.7773-0.7845) | **0.7912** (0.7876-0.7948) | **0.7980** (0.7945-0.8015) | **0.8054** (0.8019-0.8088) | **0.8142** (0.8107-0.8175) | **0.8207** (0.8174-0.8241) | **0.8273** (0.8240-0.8306) |
| Fisher | **0.6931** (0.6891-0.6972) | **0.8147** (0.8113-0.8181) | **0.8920** (0.8892-0.8947) | **0.9341** (0.9319-0.9363) | **0.9605** (0.9588-0.9622) | **0.9751** (0.9737-0.9764) | **0.9843** (0.9831-0.9853) | **0.9893** (0.9883-0.9901) | **0.9931** (0.9923-0.9938) | **0.9950** (0.9944-0.9956) | **0.9967** (0.9962-0.9972) | **0.9971** (0.9966-0.9975) | **0.9980** (0.9975-0.9983) | **0.9985** (0.9981-0.9988) | **0.9989** (0.9986-0.9992) |
| Stouffer | **0.7170** (0.7130-0.7209) | **0.8437** (0.8405-0.8469) | **0.9152** (0.9128-0.9176) | **0.9527** (0.9508-0.9545) | **0.9741** (0.9727-0.9755) | **0.9846** (0.9835-0.9856) | **0.9903** (0.9894-0.9911) | **0.9935** (0.9927-0.9941) | **0.9963** (0.9957-0.9968) | **0.9973** (0.9968-0.9977) | **0.9981** (0.9977-0.9984) | **0.9985** (0.9981-0.9988) | **0.9988** (0.9985-0.9991) | **0.9992** (0.9990-0.9994) | **0.9996** (0.9994-0.9997) |
| Wilkinson | **0.2506** (0.2468-0.2544) | **0.4896** (0.4852-0.4939) | **0.6659** (0.6617-0.6700) | **0.7243** (0.7203-0.7282) | **0.8393** (0.8361-0.8425) | **0.8727** (0.8697-0.8756) | **0.9104** (0.9079-0.9129) | **0.9415** (0.9394-0.9435) | **0.9545** (0.9527-0.9563) | **0.9653** (0.9637-0.9669) | **0.9760** (0.9746-0.9773) | **0.9808** (0.9796-0.9820) | **0.9857** (0.9846-0.9867) | **0.9896** (0.9887-0.9905) | **0.9921** (0.9913-0.9929) |
| Winer | **0.7161** (0.7122-0.7201) | **0.8430** (0.8398-0.8461) | **0.9140** (0.9115-0.9164) | **0.9519** (0.9500-0.9537) | **0.9732** (0.9718-0.9746) | **0.9842** (0.9831-0.9853) | **0.9900** (0.9891-0.9908) | **0.9935** (0.9928-0.9942) | **0.9959** (0.9953-0.9964) | **0.9974** (0.9969-0.9978) | **0.9980** (0.9975-0.9983) | **0.9982** (0.9978-0.9986) | **0.9989** (0.9986-0.9992) | **0.9992** (0.9990-0.9994) | **0.9995** (0.9993-0.9997) |
| Edgington | **0.6803** (0.6762-0.6844) | **0.8104** (0.8069-0.8138) | **0.8911** (0.8883-0.8938) | **0.9384** (0.9362-0.9404) | **0.9649** (0.9633-0.9665) | **0.9789** (0.9776-0.9801) | **0.9865** (0.9855-0.9875) | **0.9916** (0.9908-0.9924) | **0.9946** (0.9939-0.9952) | **0.9965** (0.9959-0.9970) | **0.9975** (0.9970-0.9979) | **0.9980** (0.9976-0.9984) | **0.9987** (0.9984-0.9990) | **0.9990** (0.9987-0.9992) | **0.9992** (0.9989-0.9994) |
| Mudholkar-George | **0.7119** (0.7079-0.7159) | **0.8391** (0.8359-0.8423) | **0.9125** (0.9100-0.9149) | **0.9505** (0.9485-0.9523) | **0.9728** (0.9713-0.9742) | **0.9833** (0.9821-0.9844) | **0.9897** (0.9888-0.9905) | **0.9931** (0.9924-0.9938) | **0.9959** (0.9953-0.9964) | **0.9971** (0.9966-0.9975) | **0.9977** (0.9973-0.9981) | **0.9985** (0.9982-0.9988) | **0.9989** (0.9985-0.9991) | **0.9992** (0.9990-0.9994) | **0.9996** (0.9994-0.9997) |
| Friston | **0.6354** (0.6312-0.6396) | **0.7032** (0.6991-0.7071) | **0.7443** (0.7404-0.7481) | **0.7739** (0.7702-0.7775) | **0.7936** (0.7900-0.7971) | **0.8083** (0.8048-0.8117) | **0.8184** (0.8150-0.8218) | **0.8273** (0.8239-0.8306) | **0.8334** (0.8301-0.8367) | **0.8382** (0.8350-0.8414) | **0.8448** (0.8416-0.8479) | **0.8485** (0.8453-0.8516) | **0.8529** (0.8498-0.8560) | **0.8563** (0.8532-0.8593) | **0.8601** (0.8570-0.8631) |
| Darlington-Hayes | **0.5798** (0.5754-0.5841) | **0.7857** (0.7821-0.7893) | **0.8264** (0.8231-0.8297) | **0.9122** (0.9097-0.9146) | **0.9269** (0.9246-0.9291) | **0.9614** (0.9596-0.9630) | **0.9669** (0.9653-0.9684) | **0.9820** (0.9808-0.9831) | **0.9847** (0.9835-0.9857) | **0.9911** (0.9902-0.9919) | **0.9920** (0.9912-0.9928) | **0.9949** (0.9942-0.9954) | **0.9958** (0.9952-0.9963) | **0.9970** (0.9965-0.9975) | **0.9976** (0.9971-0.9980) |
| Zaykin | **0.6838** (0.6797-0.6879) | **0.7997** (0.7962-0.8032) | **0.8725** (0.8695-0.8754) | **0.9185** (0.9160-0.9208) | **0.9461** (0.9441-0.9481) | **0.9633** (0.9616-0.9649) | **0.9750** (0.9736-0.9764) | **0.9818** (0.9805-0.9829) | **0.9873** (0.9863-0.9883) | **0.9914** (0.9906-0.9922) | **0.9930** (0.9923-0.9937) | **0.9944** (0.9937-0.9951) | **0.9958** (0.9952-0.9964) | **0.9967** (0.9962-0.9972) | **0.9973** (0.9968-0.9977) |
| Dudbridge-Koeleman | **0.5773** (0.5730-0.5816) | **0.7635** (0.7598-0.7672) | **0.8054** (0.8019-0.8088) | **0.8912** (0.8884-0.8939) | **0.9113** (0.9088-0.9138) | **0.9485** (0.9465-0.9504) | **0.9560** (0.9542-0.9578) | **0.9737** (0.9723-0.9751) | **0.9782** (0.9769-0.9795) | **0.9867** (0.9857-0.9877) | **0.9878** (0.9868-0.9888) | **0.9917** (0.9909-0.9925) | **0.9934** (0.9927-0.9941) | **0.9954** (0.9948-0.9960) | **0.9962** (0.9956-0.9967) |
| Dudbridge-Koeleman2 | **0.5785** (0.5742-0.5828) | **0.7788** (0.7751-0.7824) | **0.8202** (0.8168-0.8236) | **0.9074** (0.9048-0.9099) | **0.9262** (0.9239-0.9284) | **0.9603** (0.9585-0.9620) | **0.9675** (0.9659-0.9690) | **0.9813** (0.9801-0.9825) | **0.9847** (0.9836-0.9858) | **0.9909** (0.9900-0.9917) | **0.9922** (0.9914-0.9930) | **0.9945** (0.9939-0.9951) | **0.9959** (0.9953-0.9964) | **0.9965** (0.9959-0.9969) | **0.9971** (0.9966-0.9975) |
| Taylor-Tibshirani | **0.6997** (0.6956-0.7037) | **0.8362** (0.8329-0.8394) | **0.9107** (0.9081-0.9131) | **0.9498** (0.9479-0.9517) | **0.9723** (0.9708-0.9737) | **0.9823** (0.9811-0.9834) | **0.9892** (0.9883-0.9901) | **0.9935** (0.9927-0.9941) | **0.9958** (0.9952-0.9963) | **0.9971** (0.9965-0.9975) | **0.9976** (0.9971-0.9980) | **0.9984** (0.9980-0.9987) | **0.9988** (0.9984-0.9990) | **0.9992** (0.9989-0.9994) | **0.9994** (0.9992-0.9996) |
| Jiang | **0.6652** (0.6610-0.6693) | **0.7970** (0.7934-0.8005) | **0.8619** (0.8588-0.8649) | **0.9117** (0.9091-0.9141) | **0.9437** (0.9416-0.9456) | **0.9623** (0.9606-0.9639) | **0.9741** (0.9727-0.9754) | **0.9819** (0.9807-0.9831) | **0.9877** (0.9867-0.9886) | **0.9907** (0.9899-0.9915) | **0.9933** (0.9925-0.9939) | **0.9947** (0.9941-0.9953) | **0.9958** (0.9952-0.9964) | **0.9964** (0.9958-0.9969) | **0.9973** (0.9968-0.9977) |
| Hotelling | **0.4735** (0.4691-0.4779) | **0.5535** (0.5492-0.5579) | **0.6057** (0.6014-0.6100) | **0.6408** (0.6366-0.6450) | **0.6649** (0.6608-0.6691) | **0.6733** (0.6692-0.6774) | **0.6738** (0.6696-0.6779) | **0.6642** (0.6601-0.6684) | **0.6454** (0.6412-0.6496) | **0.6185** (0.6143-0.6228) | **0.5784** (0.5740-0.5827) | **0.5240** (0.5196-0.5284) | **0.4554** (0.4511-0.4598) | **0.3719** (0.3677-0.3762) | **0.2748** (0.2709-0.2788) |
| Wilks | **0.4688** (0.4644-0.4731) | **0.5465** (0.5421-0.5508) | **0.5987** (0.5944-0.6030) | **0.6347** (0.6305-0.6389) | **0.6529** (0.6488-0.6571) | **0.6614** (0.6572-0.6655) | **0.6618** (0.6577-0.6660) | **0.6528** (0.6486-0.6569) | **0.6367** (0.6325-0.6409) | **0.6090** (0.6047-0.6133) | **0.5681** (0.5638-0.5725) | **0.5187** (0.5143-0.5231) | **0.4498** (0.4454-0.4541) | **0.3662** (0.3620-0.3704) | **0.2715** (0.2677-0.2755) |
