# Supplementary material for: Non‐parametric combination and related permutation tests for neuroimaging
Source: Hum Brain Mapp. 2016 Feb 5;37(4):1486–511. doi: 10.1002/hbm.23115 (PMC4783210; doi:10.1002/hbm.23115)
Supplement: Supplementary file 1 — Supporting Information [file HBM-37-1486-s001.zip › supporting_information/errorrates/histograms/simD_Gauss_ee_uncp.html]

Sim D, Gauss, EE, uncp

# Simulations D, Gauss, EE, uncp

---

| Method | ksig=2 / K=2 | ksig=3 / K=3 | ksig=4 / K=4 | ksig=5 / K=5 | ksig=6 / K=6 | ksig=7 / K=7 | ksig=8 / K=8 | ksig=9 / K=9 | ksig=10 / K=10 | ksig=11 / K=11 | ksig=12 / K=12 | ksig=13 / K=13 | ksig=14 / K=14 | ksig=15 / K=15 | ksig=16 / K=16 |
| --- | --- | --- | --- | --- | --- | --- | --- | --- | --- | --- | --- | --- | --- | --- | --- |
| Tippett | **0.5687** (0.5644-0.5731) | **0.6216** (0.6173-0.6258) | **0.6638** (0.6597-0.6679) | **0.6890** (0.6849-0.6930) | **0.7081** (0.7041-0.7121) | **0.7273** (0.7234-0.7312) | **0.7401** (0.7363-0.7439) | **0.7556** (0.7518-0.7594) | **0.7643** (0.7606-0.7680) | **0.7737** (0.7700-0.7773) | **0.7812** (0.7776-0.7848) | **0.7884** (0.7848-0.7919) | **0.7973** (0.7938-0.8008) | **0.8040** (0.8005-0.8074) | **0.8088** (0.8054-0.8123) |
| Fisher | **0.6751** (0.6710-0.6792) | **0.8033** (0.7998-0.8067) | **0.8773** (0.8744-0.8801) | **0.9238** (0.9214-0.9261) | **0.9510** (0.9491-0.9529) | **0.9670** (0.9654-0.9685) | **0.9781** (0.9768-0.9793) | **0.9849** (0.9838-0.9859) | **0.9892** (0.9883-0.9901) | **0.9931** (0.9924-0.9938) | **0.9953** (0.9946-0.9958) | **0.9967** (0.9961-0.9971) | **0.9976** (0.9971-0.9980) | **0.9982** (0.9978-0.9986) | **0.9988** (0.9984-0.9990) |
| Stouffer | **0.7018** (0.6978-0.7058) | **0.8309** (0.8276-0.8342) | **0.9035** (0.9009-0.9061) | **0.9416** (0.9395-0.9436) | **0.9660** (0.9644-0.9675) | **0.9782** (0.9768-0.9794) | **0.9869** (0.9859-0.9879) | **0.9910** (0.9902-0.9918) | **0.9943** (0.9936-0.9950) | **0.9964** (0.9959-0.9969) | **0.9979** (0.9975-0.9983) | **0.9986** (0.9982-0.9989) | **0.9989** (0.9985-0.9991) | **0.9994** (0.9992-0.9996) | **0.9997** (0.9995-0.9998) |
| Wilkinson | **0.2386** (0.2349-0.2424) | **0.4734** (0.4690-0.4778) | **0.6451** (0.6409-0.6492) | **0.7071** (0.7031-0.7111) | **0.8223** (0.8189-0.8256) | **0.8589** (0.8558-0.8619) | **0.8942** (0.8915-0.8969) | **0.9286** (0.9263-0.9308) | **0.9441** (0.9421-0.9461) | **0.9576** (0.9558-0.9593) | **0.9684** (0.9669-0.9699) | **0.9759** (0.9745-0.9772) | **0.9817** (0.9805-0.9828) | **0.9863** (0.9852-0.9873) | **0.9892** (0.9882-0.9900) |
| Winer | **0.6990** (0.6950-0.7030) | **0.8298** (0.8265-0.8331) | **0.9024** (0.8998-0.9050) | **0.9412** (0.9391-0.9432) | **0.9653** (0.9636-0.9668) | **0.9780** (0.9767-0.9793) | **0.9860** (0.9850-0.9870) | **0.9908** (0.9899-0.9916) | **0.9939** (0.9932-0.9946) | **0.9964** (0.9958-0.9969) | **0.9977** (0.9972-0.9980) | **0.9985** (0.9982-0.9988) | **0.9990** (0.9987-0.9993) | **0.9992** (0.9990-0.9994) | **0.9996** (0.9994-0.9998) |
| Edgington | **0.6685** (0.6643-0.6726) | **0.7928** (0.7892-0.7963) | **0.8779** (0.8750-0.8808) | **0.9262** (0.9238-0.9284) | **0.9551** (0.9533-0.9569) | **0.9710** (0.9695-0.9724) | **0.9813** (0.9801-0.9824) | **0.9883** (0.9873-0.9892) | **0.9923** (0.9915-0.9930) | **0.9952** (0.9946-0.9958) | **0.9969** (0.9963-0.9973) | **0.9978** (0.9973-0.9982) | **0.9985** (0.9982-0.9988) | **0.9989** (0.9986-0.9992) | **0.9994** (0.9991-0.9995) |
| Mudholkar-George | **0.6956** (0.6915-0.6996) | **0.8273** (0.8240-0.8306) | **0.8987** (0.8960-0.9013) | **0.9391** (0.9370-0.9412) | **0.9632** (0.9615-0.9648) | **0.9765** (0.9751-0.9778) | **0.9856** (0.9845-0.9866) | **0.9904** (0.9895-0.9912) | **0.9940** (0.9933-0.9946) | **0.9961** (0.9955-0.9966) | **0.9976** (0.9972-0.9980) | **0.9984** (0.9980-0.9987) | **0.9988** (0.9984-0.9991) | **0.9992** (0.9989-0.9994) | **0.9996** (0.9994-0.9997) |
| Friston | **0.6212** (0.6169-0.6254) | **0.6924** (0.6884-0.6965) | **0.7315** (0.7276-0.7353) | **0.7604** (0.7566-0.7641) | **0.7767** (0.7730-0.7803) | **0.7914** (0.7878-0.7950) | **0.8026** (0.7991-0.8061) | **0.8115** (0.8081-0.8149) | **0.8199** (0.8165-0.8232) | **0.8270** (0.8237-0.8303) | **0.8342** (0.8310-0.8375) | **0.8382** (0.8350-0.8414) | **0.8424** (0.8392-0.8456) | **0.8463** (0.8431-0.8494) | **0.8491** (0.8459-0.8522) |
| Darlington-Hayes | **0.5702** (0.5659-0.5746) | **0.7721** (0.7684-0.7758) | **0.8154** (0.8119-0.8187) | **0.8989** (0.8962-0.9015) | **0.9153** (0.9128-0.9177) | **0.9518** (0.9499-0.9536) | **0.9586** (0.9569-0.9604) | **0.9759** (0.9745-0.9772) | **0.9788** (0.9775-0.9800) | **0.9877** (0.9867-0.9886) | **0.9890** (0.9880-0.9899) | **0.9937** (0.9930-0.9944) | **0.9941** (0.9933-0.9947) | **0.9962** (0.9956-0.9967) | **0.9966** (0.9961-0.9971) |
| Zaykin | **0.6702** (0.6661-0.6743) | **0.7834** (0.7798-0.7870) | **0.8586** (0.8555-0.8616) | **0.9057** (0.9031-0.9082) | **0.9338** (0.9316-0.9360) | **0.9535** (0.9516-0.9553) | **0.9665** (0.9649-0.9680) | **0.9760** (0.9746-0.9773) | **0.9819** (0.9807-0.9830) | **0.9875** (0.9865-0.9884) | **0.9903** (0.9894-0.9911) | **0.9935** (0.9927-0.9941) | **0.9946** (0.9939-0.9952) | **0.9962** (0.9957-0.9967) | **0.9971** (0.9966-0.9975) |
| Dudbridge-Koeleman | **0.5690** (0.5647-0.5733) | **0.7513** (0.7475-0.7551) | **0.7937** (0.7902-0.7973) | **0.8796** (0.8767-0.8824) | **0.8989** (0.8962-0.9015) | **0.9374** (0.9353-0.9395) | **0.9472** (0.9452-0.9491) | **0.9665** (0.9649-0.9680) | **0.9711** (0.9696-0.9725) | **0.9818** (0.9806-0.9830) | **0.9839** (0.9828-0.9850) | **0.9898** (0.9889-0.9906) | **0.9907** (0.9899-0.9915) | **0.9939** (0.9932-0.9945) | **0.9943** (0.9936-0.9950) |
| Dudbridge-Koeleman2 | **0.5675** (0.5632-0.5719) | **0.7649** (0.7611-0.7686) | **0.8076** (0.8041-0.8110) | **0.8948** (0.8920-0.8974) | **0.9134** (0.9109-0.9158) | **0.9498** (0.9479-0.9517) | **0.9588** (0.9570-0.9605) | **0.9748** (0.9734-0.9761) | **0.9790** (0.9777-0.9802) | **0.9866** (0.9856-0.9876) | **0.9892** (0.9883-0.9901) | **0.9930** (0.9922-0.9937) | **0.9942** (0.9935-0.9948) | **0.9959** (0.9953-0.9965) | **0.9967** (0.9962-0.9972) |
| Taylor-Tibshirani | **0.6861** (0.6820-0.6902) | **0.8214** (0.8180-0.8248) | **0.8985** (0.8958-0.9011) | **0.9389** (0.9367-0.9409) | **0.9631** (0.9614-0.9647) | **0.9770** (0.9757-0.9783) | **0.9854** (0.9844-0.9865) | **0.9901** (0.9892-0.9909) | **0.9936** (0.9929-0.9943) | **0.9958** (0.9952-0.9963) | **0.9971** (0.9966-0.9975) | **0.9981** (0.9977-0.9984) | **0.9986** (0.9982-0.9989) | **0.9989** (0.9986-0.9992) | **0.9995** (0.9992-0.9996) |
| Jiang | **0.6502** (0.6460-0.6544) | **0.7810** (0.7773-0.7846) | **0.8486** (0.8455-0.8517) | **0.8989** (0.8962-0.9015) | **0.9330** (0.9308-0.9351) | **0.9531** (0.9512-0.9549) | **0.9659** (0.9642-0.9674) | **0.9757** (0.9743-0.9770) | **0.9819** (0.9807-0.9831) | **0.9869** (0.9858-0.9878) | **0.9904** (0.9895-0.9912) | **0.9934** (0.9927-0.9941) | **0.9950** (0.9943-0.9956) | **0.9962** (0.9956-0.9967) | **0.9969** (0.9964-0.9974) |
| Hotelling | **0.4655** (0.4611-0.4699) | **0.5458** (0.5414-0.5501) | **0.5954** (0.5911-0.5997) | **0.6276** (0.6234-0.6318) | **0.6534** (0.6492-0.6575) | **0.6606** (0.6564-0.6647) | **0.6597** (0.6556-0.6639) | **0.6502** (0.6460-0.6544) | **0.6320** (0.6278-0.6362) | **0.6007** (0.5964-0.6050) | **0.5623** (0.5579-0.5666) | **0.5133** (0.5089-0.5177) | **0.4459** (0.4415-0.4502) | **0.3649** (0.3607-0.3691) | **0.2711** (0.2672-0.2750) |
| Wilks | **0.4659** (0.4616-0.4703) | **0.5456** (0.5413-0.5500) | **0.5972** (0.5929-0.6015) | **0.6289** (0.6246-0.6331) | **0.6504** (0.6462-0.6546) | **0.6613** (0.6571-0.6654) | **0.6606** (0.6564-0.6647) | **0.6485** (0.6443-0.6527) | **0.6316** (0.6273-0.6358) | **0.6046** (0.6003-0.6088) | **0.5640** (0.5596-0.5683) | **0.5122** (0.5078-0.5166) | **0.4454** (0.4410-0.4498) | **0.3635** (0.3593-0.3678) | **0.2685** (0.2647-0.2724) |
