# Supplementary material for: Non‐parametric combination and related permutation tests for neuroimaging
Source: Hum Brain Mapp. 2016 Feb 5;37(4):1486–511. doi: 10.1002/hbm.23115 (PMC4783210; doi:10.1002/hbm.23115)
Supplement: Supplementary file 1 — Supporting Information [file HBM-37-1486-s001.zip › supporting_information/errorrates/histograms/simD_Gauss_ise_uncp.html]

Sim D, Gauss, ISE, uncp

# Simulations D, Gauss, ISE, uncp

---

| Method | ksig=2 / K=2 | ksig=3 / K=3 | ksig=4 / K=4 | ksig=5 / K=5 | ksig=6 / K=6 | ksig=7 / K=7 | ksig=8 / K=8 | ksig=9 / K=9 | ksig=10 / K=10 | ksig=11 / K=11 | ksig=12 / K=12 | ksig=13 / K=13 | ksig=14 / K=14 | ksig=15 / K=15 | ksig=16 / K=16 |
| --- | --- | --- | --- | --- | --- | --- | --- | --- | --- | --- | --- | --- | --- | --- | --- |
| Tippett | **0.5197** (0.5153-0.5241) | **0.5591** (0.5548-0.5635) | **0.5908** (0.5864-0.5951) | **0.6081** (0.6038-0.6124) | **0.6291** (0.6249-0.6333) | **0.6466** (0.6424-0.6508) | **0.6516** (0.6474-0.6557) | **0.6652** (0.6611-0.6693) | **0.6715** (0.6673-0.6756) | **0.6823** (0.6782-0.6864) | **0.6855** (0.6815-0.6896) | **0.6974** (0.6934-0.7014) | **0.7017** (0.6977-0.7057) | **0.7037** (0.6996-0.7076) | **0.7078** (0.7038-0.7118) |
| Fisher | **0.6284** (0.6242-0.6326) | **0.7516** (0.7478-0.7554) | **0.8306** (0.8273-0.8339) | **0.8865** (0.8836-0.8892) | **0.9197** (0.9173-0.9220) | **0.9438** (0.9417-0.9457) | **0.9581** (0.9563-0.9598) | **0.9717** (0.9702-0.9731) | **0.9787** (0.9774-0.9799) | **0.9850** (0.9839-0.9861) | **0.9890** (0.9881-0.9899) | **0.9914** (0.9906-0.9922) | **0.9936** (0.9928-0.9942) | **0.9947** (0.9940-0.9953) | **0.9956** (0.9950-0.9962) |
| Stouffer | **0.6523** (0.6481-0.6564) | **0.7810** (0.7774-0.7846) | **0.8635** (0.8605-0.8665) | **0.9115** (0.9090-0.9140) | **0.9407** (0.9386-0.9428) | **0.9631** (0.9614-0.9647) | **0.9739** (0.9725-0.9753) | **0.9836** (0.9824-0.9847) | **0.9876** (0.9866-0.9885) | **0.9915** (0.9907-0.9923) | **0.9938** (0.9930-0.9944) | **0.9957** (0.9951-0.9963) | **0.9969** (0.9963-0.9973) | **0.9975** (0.9971-0.9979) | **0.9980** (0.9975-0.9983) |
| Wilkinson | **0.1095** (0.1068-0.1122) | **0.2813** (0.2774-0.2853) | **0.4128** (0.4085-0.4172) | **0.5236** (0.5192-0.5280) | **0.6229** (0.6186-0.6271) | **0.6985** (0.6945-0.7025) | **0.7528** (0.7490-0.7565) | **0.8001** (0.7966-0.8036) | **0.8385** (0.8353-0.8417) | **0.8684** (0.8654-0.8713) | **0.8873** (0.8845-0.8901) | **0.9093** (0.9068-0.9118) | **0.9227** (0.9203-0.9250) | **0.9389** (0.9367-0.9409) | **0.9471** (0.9451-0.9490) |
| Winer | **0.6508** (0.6466-0.6550) | **0.7782** (0.7745-0.7818) | **0.8630** (0.8600-0.8660) | **0.9111** (0.9086-0.9135) | **0.9407** (0.9386-0.9428) | **0.9609** (0.9592-0.9626) | **0.9734** (0.9720-0.9748) | **0.9820** (0.9808-0.9831) | **0.9873** (0.9862-0.9882) | **0.9913** (0.9904-0.9920) | **0.9936** (0.9929-0.9943) | **0.9954** (0.9948-0.9960) | **0.9961** (0.9956-0.9966) | **0.9972** (0.9967-0.9976) | **0.9977** (0.9973-0.9981) |
| Edgington | **0.6124** (0.6081-0.6166) | **0.7301** (0.7262-0.7340) | **0.8171** (0.8136-0.8204) | **0.8767** (0.8738-0.8796) | **0.9154** (0.9129-0.9178) | **0.9423** (0.9402-0.9443) | **0.9576** (0.9558-0.9594) | **0.9725** (0.9711-0.9739) | **0.9789** (0.9776-0.9802) | **0.9850** (0.9839-0.9860) | **0.9892** (0.9883-0.9901) | **0.9919** (0.9911-0.9926) | **0.9938** (0.9931-0.9945) | **0.9953** (0.9946-0.9958) | **0.9963** (0.9957-0.9968) |
| Mudholkar-George | **0.6460** (0.6418-0.6502) | **0.7729** (0.7692-0.7765) | **0.8558** (0.8527-0.8588) | **0.9053** (0.9027-0.9078) | **0.9365** (0.9343-0.9386) | **0.9581** (0.9563-0.9598) | **0.9700** (0.9684-0.9714) | **0.9810** (0.9797-0.9821) | **0.9864** (0.9853-0.9874) | **0.9905** (0.9896-0.9913) | **0.9930** (0.9922-0.9937) | **0.9950** (0.9943-0.9955) | **0.9960** (0.9954-0.9965) | **0.9968** (0.9963-0.9973) | **0.9979** (0.9974-0.9982) |
| Friston | **0.5648** (0.5604-0.5691) | **0.6239** (0.6197-0.6282) | **0.6613** (0.6572-0.6655) | **0.6824** (0.6783-0.6865) | **0.7030** (0.6989-0.7069) | **0.7122** (0.7082-0.7162) | **0.7270** (0.7231-0.7309) | **0.7325** (0.7286-0.7363) | **0.7388** (0.7349-0.7426) | **0.7458** (0.7420-0.7496) | **0.7522** (0.7484-0.7559) | **0.7566** (0.7528-0.7603) | **0.7644** (0.7607-0.7681) | **0.7686** (0.7649-0.7723) | **0.7714** (0.7677-0.7750) |
| Darlington-Hayes | **0.5221** (0.5177-0.5265) | **0.7109** (0.7069-0.7149) | **0.7443** (0.7405-0.7481) | **0.8471** (0.8439-0.8502) | **0.8594** (0.8564-0.8625) | **0.9131** (0.9106-0.9156) | **0.9217** (0.9193-0.9240) | **0.9492** (0.9472-0.9511) | **0.9534** (0.9516-0.9553) | **0.9695** (0.9680-0.9710) | **0.9726** (0.9711-0.9740) | **0.9818** (0.9806-0.9830) | **0.9811** (0.9798-0.9822) | **0.9877** (0.9867-0.9886) | **0.9877** (0.9867-0.9886) |
| Zaykin | **0.6221** (0.6179-0.6264) | **0.7391** (0.7353-0.7430) | **0.8147** (0.8113-0.8181) | **0.8670** (0.8640-0.8700) | **0.9017** (0.8990-0.9043) | **0.9280** (0.9257-0.9302) | **0.9475** (0.9456-0.9495) | **0.9605** (0.9588-0.9622) | **0.9699** (0.9683-0.9713) | **0.9762** (0.9748-0.9775) | **0.9827** (0.9815-0.9838) | **0.9858** (0.9847-0.9868) | **0.9888** (0.9879-0.9897) | **0.9909** (0.9900-0.9917) | **0.9928** (0.9920-0.9935) |
| Dudbridge-Koeleman | **0.5154** (0.5110-0.5198) | **0.6893** (0.6852-0.6934) | **0.7243** (0.7204-0.7282) | **0.8209** (0.8175-0.8242) | **0.8404** (0.8371-0.8436) | **0.8950** (0.8923-0.8977) | **0.9010** (0.8983-0.9035) | **0.9329** (0.9307-0.9351) | **0.9401** (0.9379-0.9421) | **0.9571** (0.9553-0.9589) | **0.9630** (0.9613-0.9646) | **0.9742** (0.9728-0.9756) | **0.9752** (0.9738-0.9765) | **0.9807** (0.9795-0.9819) | **0.9833** (0.9822-0.9844) |
| Dudbridge-Koeleman2 | **0.5150** (0.5106-0.5194) | **0.7031** (0.6990-0.7070) | **0.7374** (0.7335-0.7412) | **0.8379** (0.8346-0.8411) | **0.8521** (0.8490-0.8552) | **0.9069** (0.9043-0.9094) | **0.9158** (0.9134-0.9182) | **0.9465** (0.9445-0.9484) | **0.9507** (0.9488-0.9526) | **0.9679** (0.9663-0.9694) | **0.9708** (0.9692-0.9722) | **0.9809** (0.9796-0.9820) | **0.9807** (0.9794-0.9819) | **0.9864** (0.9853-0.9874) | **0.9873** (0.9863-0.9882) |
| Taylor-Tibshirani | **0.6306** (0.6264-0.6349) | **0.7619** (0.7582-0.7657) | **0.8466** (0.8434-0.8497) | **0.9017** (0.8991-0.9043) | **0.9359** (0.9338-0.9381) | **0.9575** (0.9557-0.9593) | **0.9706** (0.9691-0.9720) | **0.9800** (0.9787-0.9812) | **0.9853** (0.9842-0.9863) | **0.9902** (0.9893-0.9910) | **0.9926** (0.9918-0.9933) | **0.9949** (0.9943-0.9955) | **0.9960** (0.9954-0.9965) | **0.9968** (0.9963-0.9973) | **0.9976** (0.9971-0.9980) |
| Jiang | **0.6036** (0.5993-0.6079) | **0.7120** (0.7080-0.7160) | **0.7880** (0.7844-0.7916) | **0.8464** (0.8432-0.8495) | **0.8846** (0.8818-0.8874) | **0.9122** (0.9097-0.9146) | **0.9347** (0.9325-0.9368) | **0.9498** (0.9479-0.9517) | **0.9617** (0.9599-0.9633) | **0.9702** (0.9687-0.9717) | **0.9769** (0.9755-0.9782) | **0.9820** (0.9808-0.9832) | **0.9855** (0.9844-0.9865) | **0.9881** (0.9871-0.9890) | **0.9908** (0.9899-0.9916) |
| Hotelling | **0.4158** (0.4115-0.4201) | **0.4917** (0.4873-0.4960) | **0.5414** (0.5370-0.5458) | **0.5769** (0.5725-0.5812) | **0.5980** (0.5937-0.6022) | **0.6114** (0.6071-0.6156) | **0.6140** (0.6097-0.6183) | **0.6048** (0.6005-0.6090) | **0.5907** (0.5864-0.5950) | **0.5641** (0.5598-0.5685) | **0.5289** (0.5245-0.5333) | **0.4869** (0.4825-0.4912) | **0.4239** (0.4196-0.4282) | **0.3476** (0.3434-0.3517) | **0.2644** (0.2605-0.2683) |
| Wilks | **0.4172** (0.4129-0.4216) | **0.4983** (0.4939-0.5026) | **0.5527** (0.5483-0.5570) | **0.5869** (0.5826-0.5912) | **0.6139** (0.6096-0.6181) | **0.6252** (0.6209-0.6294) | **0.6280** (0.6238-0.6322) | **0.6198** (0.6155-0.6240) | **0.6026** (0.5983-0.6069) | **0.5801** (0.5758-0.5844) | **0.5427** (0.5383-0.5470) | **0.4939** (0.4895-0.4983) | **0.4293** (0.4249-0.4336) | **0.3556** (0.3514-0.3598) | **0.2698** (0.2659-0.2737) |
