# Supplementary material for: Non‐parametric combination and related permutation tests for neuroimaging
Source: Hum Brain Mapp. 2016 Feb 5;37(4):1486–511. doi: 10.1002/hbm.23115 (PMC4783210; doi:10.1002/hbm.23115)
Supplement: Supplementary file 1 — Supporting Information [file HBM-37-1486-s001.zip › supporting_information/errorrates/histograms/simD_Weibull_ee+ise_uncp.html]

Sim D, Weibull, EE+ISE, uncp

# Simulations D, Weibull, EE+ISE, uncp

---

| Method | ksig=2 / K=2 | ksig=3 / K=3 | ksig=4 / K=4 | ksig=5 / K=5 | ksig=6 / K=6 | ksig=7 / K=7 | ksig=8 / K=8 | ksig=9 / K=9 | ksig=10 / K=10 | ksig=11 / K=11 | ksig=12 / K=12 | ksig=13 / K=13 | ksig=14 / K=14 | ksig=15 / K=15 | ksig=16 / K=16 |
| --- | --- | --- | --- | --- | --- | --- | --- | --- | --- | --- | --- | --- | --- | --- | --- |
| Tippett | **0.8638** (0.8607-0.8668) | **0.9314** (0.9292-0.9336) | **0.9645** (0.9628-0.9660) | **0.9793** (0.9781-0.9806) | **0.9881** (0.9871-0.9890) | **0.9931** (0.9923-0.9938) | **0.9958** (0.9952-0.9963) | **0.9972** (0.9967-0.9976) | **0.9983** (0.9979-0.9987) | **0.9988** (0.9984-0.9991) | **0.9992** (0.9989-0.9994) | **0.9995** (0.9993-0.9997) | **0.9997** (0.9995-0.9998) | **0.9998** (0.9997-0.9999) | **0.9998** (0.9997-0.9999) |
| Fisher | **0.8854** (0.8826-0.8881) | **0.9561** (0.9542-0.9578) | **0.9837** (0.9825-0.9847) | **0.9928** (0.9920-0.9935) | **0.9969** (0.9964-0.9974) | **0.9988** (0.9985-0.9991) | **0.9995** (0.9993-0.9997) | **0.9999** (0.9997-0.9999) | **1.0000** (0.9999-1.0000) | **1.0000** (0.9999-1.0000) | **1.0000** (0.9999-1.0000) | **1.0000** (0.9999-1.0000) | **1.0000** (0.9999-1.0000) | **1.0000** (0.9999-1.0000) | **1.0000** (0.9999-1.0000) |
| Stouffer | **0.8814** (0.8785-0.8842) | **0.9532** (0.9513-0.9550) | **0.9820** (0.9808-0.9832) | **0.9918** (0.9909-0.9925) | **0.9964** (0.9958-0.9969) | **0.9987** (0.9984-0.9990) | **0.9994** (0.9991-0.9995) | **0.9998** (0.9996-0.9999) | **1.0000** (0.9999-1.0000) | **1.0000** (0.9999-1.0000) | **1.0000** (0.9999-1.0000) | **1.0000** (0.9999-1.0000) | **1.0000** (0.9999-1.0000) | **1.0000** (0.9999-1.0000) | **1.0000** (0.9999-1.0000) |
| Wilkinson | **0.1315** (0.1286-0.1345) | **0.4184** (0.4141-0.4227) | **0.5705** (0.5661-0.5748) | **0.7183** (0.7143-0.7222) | **0.8060** (0.8025-0.8094) | **0.8698** (0.8669-0.8728) | **0.9168** (0.9144-0.9192) | **0.9428** (0.9408-0.9448) | **0.9615** (0.9597-0.9631) | **0.9765** (0.9751-0.9778) | **0.9827** (0.9815-0.9838) | **0.9895** (0.9886-0.9904) | **0.9929** (0.9921-0.9936) | **0.9949** (0.9943-0.9955) | **0.9965** (0.9959-0.9969) |
| Winer | **0.8838** (0.8809-0.8866) | **0.9558** (0.9539-0.9575) | **0.9831** (0.9820-0.9842) | **0.9928** (0.9920-0.9935) | **0.9968** (0.9962-0.9972) | **0.9988** (0.9985-0.9991) | **0.9995** (0.9992-0.9996) | **0.9998** (0.9996-0.9999) | **1.0000** (0.9999-1.0000) | **1.0000** (0.9999-1.0000) | **1.0000** (0.9999-1.0000) | **1.0000** (0.9999-1.0000) | **1.0000** (0.9999-1.0000) | **1.0000** (0.9999-1.0000) | **1.0000** (0.9999-1.0000) |
| Edgington | **0.7606** (0.7568-0.7643) | **0.8282** (0.8248-0.8315) | **0.9023** (0.8997-0.9049) | **0.9487** (0.9467-0.9506) | **0.9686** (0.9671-0.9701) | **0.9826** (0.9814-0.9837) | **0.9886** (0.9876-0.9895) | **0.9943** (0.9936-0.9949) | **0.9964** (0.9958-0.9969) | **0.9979** (0.9975-0.9983) | **0.9989** (0.9986-0.9992) | **0.9994** (0.9991-0.9996) | **0.9996** (0.9994-0.9997) | **0.9998** (0.9996-0.9999) | **0.9998** (0.9996-0.9999) |
| Mudholkar-George | **0.8861** (0.8832-0.8888) | **0.9567** (0.9549-0.9584) | **0.9837** (0.9825-0.9848) | **0.9930** (0.9923-0.9937) | **0.9969** (0.9964-0.9974) | **0.9989** (0.9986-0.9992) | **0.9995** (0.9992-0.9996) | **0.9998** (0.9996-0.9999) | **1.0000** (0.9999-1.0000) | **1.0000** (0.9999-1.0000) | **1.0000** (0.9999-1.0000) | **1.0000** (0.9999-1.0000) | **1.0000** (0.9999-1.0000) | **1.0000** (0.9999-1.0000) | **1.0000** (0.9999-1.0000) |
| Friston | **0.7064** (0.7024-0.7104) | **0.6990** (0.6950-0.7030) | **0.6923** (0.6883-0.6964) | **0.6891** (0.6850-0.6931) | **0.6851** (0.6810-0.6891) | **0.6807** (0.6766-0.6848) | **0.6793** (0.6752-0.6834) | **0.6770** (0.6729-0.6811) | **0.6733** (0.6692-0.6774) | **0.6731** (0.6690-0.6772) | **0.6747** (0.6706-0.6788) | **0.6746** (0.6705-0.6787) | **0.6723** (0.6682-0.6764) | **0.6738** (0.6697-0.6779) | **0.6716** (0.6675-0.6757) |
| Darlington-Hayes | **0.8636** (0.8606-0.8666) | **0.9512** (0.9493-0.9530) | **0.9785** (0.9772-0.9797) | **0.9916** (0.9908-0.9924) | **0.9959** (0.9953-0.9965) | **0.9985** (0.9982-0.9988) | **0.9994** (0.9991-0.9996) | **0.9997** (0.9996-0.9998) | **0.9999** (0.9998-1.0000) | **0.9999** (0.9998-1.0000) | **1.0000** (0.9999-1.0000) | **1.0000** (0.9999-1.0000) | **1.0000** (0.9999-1.0000) | **1.0000** (0.9999-1.0000) | **1.0000** (0.9999-1.0000) |
| Zaykin | **0.8808** (0.8779-0.8836) | **0.9513** (0.9494-0.9532) | **0.9804** (0.9792-0.9816) | **0.9910** (0.9902-0.9918) | **0.9959** (0.9953-0.9964) | **0.9983** (0.9979-0.9986) | **0.9993** (0.9990-0.9995) | **0.9996** (0.9994-0.9998) | **0.9999** (0.9997-0.9999) | **1.0000** (0.9999-1.0000) | **1.0000** (0.9999-1.0000) | **1.0000** (0.9999-1.0000) | **1.0000** (0.9999-1.0000) | **1.0000** (0.9999-1.0000) | **1.0000** (0.9999-1.0000) |
| Dudbridge-Koeleman | **0.8628** (0.8597-0.8658) | **0.9500** (0.9480-0.9519) | **0.9767** (0.9754-0.9780) | **0.9910** (0.9902-0.9918) | **0.9956** (0.9949-0.9961) | **0.9983** (0.9979-0.9986) | **0.9991** (0.9988-0.9993) | **0.9997** (0.9995-0.9998) | **0.9999** (0.9998-1.0000) | **1.0000** (0.9999-1.0000) | **1.0000** (0.9999-1.0000) | **1.0000** (0.9999-1.0000) | **1.0000** (0.9999-1.0000) | **1.0000** (0.9999-1.0000) | **1.0000** (0.9999-1.0000) |
| Dudbridge-Koeleman2 | **0.8635** (0.8605-0.8665) | **0.9499** (0.9480-0.9518) | **0.9776** (0.9762-0.9788) | **0.9908** (0.9899-0.9916) | **0.9958** (0.9952-0.9963) | **0.9982** (0.9978-0.9986) | **0.9992** (0.9989-0.9994) | **0.9997** (0.9995-0.9998) | **0.9999** (0.9998-1.0000) | **1.0000** (0.9999-1.0000) | **1.0000** (0.9999-1.0000) | **1.0000** (0.9999-1.0000) | **1.0000** (0.9999-1.0000) | **1.0000** (0.9999-1.0000) | **1.0000** (0.9999-1.0000) |
| Taylor-Tibshirani | **0.7910** (0.7874-0.7946) | **0.8954** (0.8927-0.8981) | **0.9553** (0.9534-0.9570) | **0.9795** (0.9782-0.9807) | **0.9900** (0.9891-0.9909) | **0.9955** (0.9948-0.9960) | **0.9979** (0.9975-0.9983) | **0.9990** (0.9987-0.9993) | **0.9996** (0.9994-0.9997) | **0.9999** (0.9997-0.9999) | **0.9999** (0.9998-1.0000) | **1.0000** (0.9999-1.0000) | **0.9999** (0.9998-1.0000) | **1.0000** (0.9999-1.0000) | **1.0000** (0.9999-1.0000) |
| Jiang | **0.8288** (0.8255-0.8321) | **0.9078** (0.9052-0.9103) | **0.9490** (0.9470-0.9509) | **0.9719** (0.9704-0.9733) | **0.9869** (0.9858-0.9878) | **0.9939** (0.9932-0.9945) | **0.9971** (0.9965-0.9975) | **0.9987** (0.9984-0.9990) | **0.9996** (0.9993-0.9997) | **0.9998** (0.9996-0.9999) | **0.9999** (0.9997-0.9999) | **1.0000** (0.9999-1.0000) | **1.0000** (0.9999-1.0000) | **1.0000** (0.9999-1.0000) | **1.0000** (0.9999-1.0000) |
| Hotelling | **0.8246** (0.8213-0.8279) | **0.9099** (0.9074-0.9124) | **0.9512** (0.9492-0.9530) | **0.9719** (0.9704-0.9733) | **0.9820** (0.9808-0.9831) | **0.9878** (0.9868-0.9887) | **0.9914** (0.9905-0.9922) | **0.9935** (0.9927-0.9941) | **0.9949** (0.9942-0.9954) | **0.9949** (0.9942-0.9954) | **0.9946** (0.9939-0.9952) | **0.9933** (0.9926-0.9940) | **0.9889** (0.9879-0.9898) | **0.9772** (0.9759-0.9785) | **0.9397** (0.9376-0.9418) |
| Wilks | **0.8251** (0.8218-0.8284) | **0.9095** (0.9069-0.9120) | **0.9510** (0.9491-0.9529) | **0.9716** (0.9701-0.9730) | **0.9823** (0.9811-0.9834) | **0.9880** (0.9870-0.9889) | **0.9918** (0.9909-0.9925) | **0.9933** (0.9925-0.9940) | **0.9947** (0.9941-0.9953) | **0.9948** (0.9942-0.9954) | **0.9945** (0.9938-0.9951) | **0.9932** (0.9925-0.9939) | **0.9888** (0.9878-0.9896) | **0.9779** (0.9766-0.9792) | **0.9398** (0.9377-0.9419) |
