# Supplementary material for: Non‐parametric combination and related permutation tests for neuroimaging
Source: Hum Brain Mapp. 2016 Feb 5;37(4):1486–511. doi: 10.1002/hbm.23115 (PMC4783210; doi:10.1002/hbm.23115)
Supplement: Supplementary file 1 — Supporting Information [file HBM-37-1486-s001.zip › supporting_information/errorrates/histograms/simD_Weibull_ee_uncp.html]

Sim D, Weibull, EE, uncp

# Simulations D, Weibull, EE, uncp

---

| Method | ksig=2 / K=2 | ksig=3 / K=3 | ksig=4 / K=4 | ksig=5 / K=5 | ksig=6 / K=6 | ksig=7 / K=7 | ksig=8 / K=8 | ksig=9 / K=9 | ksig=10 / K=10 | ksig=11 / K=11 | ksig=12 / K=12 | ksig=13 / K=13 | ksig=14 / K=14 | ksig=15 / K=15 | ksig=16 / K=16 |
| --- | --- | --- | --- | --- | --- | --- | --- | --- | --- | --- | --- | --- | --- | --- | --- |
| Tippett | **0.8608** (0.8577-0.8638) | **0.9310** (0.9288-0.9332) | **0.9642** (0.9625-0.9658) | **0.9801** (0.9789-0.9813) | **0.9881** (0.9871-0.9890) | **0.9927** (0.9919-0.9934) | **0.9959** (0.9953-0.9964) | **0.9972** (0.9967-0.9976) | **0.9985** (0.9981-0.9988) | **0.9990** (0.9986-0.9992) | **0.9992** (0.9989-0.9994) | **0.9995** (0.9992-0.9996) | **0.9997** (0.9995-0.9998) | **0.9998** (0.9996-0.9999) | **0.9998** (0.9997-0.9999) |
| Fisher | **0.8816** (0.8788-0.8844) | **0.9542** (0.9524-0.9560) | **0.9828** (0.9816-0.9839) | **0.9935** (0.9927-0.9941) | **0.9968** (0.9962-0.9972) | **0.9988** (0.9984-0.9991) | **0.9995** (0.9993-0.9997) | **0.9999** (0.9997-0.9999) | **1.0000** (0.9999-1.0000) | **1.0000** (0.9999-1.0000) | **1.0000** (0.9999-1.0000) | **1.0000** (0.9999-1.0000) | **1.0000** (0.9999-1.0000) | **1.0000** (0.9999-1.0000) | **1.0000** (0.9999-1.0000) |
| Stouffer | **0.8774** (0.8745-0.8803) | **0.9514** (0.9495-0.9533) | **0.9803** (0.9790-0.9815) | **0.9924** (0.9916-0.9931) | **0.9971** (0.9966-0.9975) | **0.9987** (0.9983-0.9990) | **0.9994** (0.9992-0.9996) | **0.9998** (0.9997-0.9999) | **0.9999** (0.9997-0.9999) | **1.0000** (0.9999-1.0000) | **1.0000** (0.9999-1.0000) | **1.0000** (0.9999-1.0000) | **1.0000** (0.9999-1.0000) | **1.0000** (0.9999-1.0000) | **1.0000** (0.9999-1.0000) |
| Wilkinson | **0.1285** (0.1256-0.1314) | **0.4035** (0.3992-0.4078) | **0.5706** (0.5663-0.5750) | **0.7127** (0.7087-0.7167) | **0.8069** (0.8034-0.8103) | **0.8649** (0.8619-0.8679) | **0.9129** (0.9104-0.9154) | **0.9419** (0.9398-0.9439) | **0.9611** (0.9594-0.9628) | **0.9751** (0.9737-0.9764) | **0.9825** (0.9813-0.9836) | **0.9891** (0.9881-0.9900) | **0.9921** (0.9913-0.9929) | **0.9946** (0.9939-0.9952) | **0.9966** (0.9960-0.9971) |
| Winer | **0.8797** (0.8768-0.8825) | **0.9535** (0.9517-0.9554) | **0.9821** (0.9809-0.9832) | **0.9932** (0.9925-0.9939) | **0.9973** (0.9968-0.9977) | **0.9988** (0.9985-0.9991) | **0.9995** (0.9993-0.9997) | **0.9998** (0.9997-0.9999) | **0.9999** (0.9998-1.0000) | **1.0000** (0.9999-1.0000) | **1.0000** (0.9999-1.0000) | **1.0000** (0.9999-1.0000) | **1.0000** (0.9999-1.0000) | **1.0000** (0.9999-1.0000) | **1.0000** (0.9999-1.0000) |
| Edgington | **0.7591** (0.7553-0.7628) | **0.8287** (0.8254-0.8320) | **0.8972** (0.8945-0.8998) | **0.9444** (0.9424-0.9464) | **0.9657** (0.9641-0.9673) | **0.9812** (0.9800-0.9824) | **0.9888** (0.9879-0.9897) | **0.9934** (0.9926-0.9940) | **0.9963** (0.9958-0.9968) | **0.9978** (0.9973-0.9981) | **0.9985** (0.9981-0.9988) | **0.9990** (0.9987-0.9993) | **0.9995** (0.9993-0.9997) | **0.9998** (0.9996-0.9999) | **0.9998** (0.9997-0.9999) |
| Mudholkar-George | **0.8818** (0.8789-0.8846) | **0.9550** (0.9532-0.9568) | **0.9824** (0.9812-0.9835) | **0.9933** (0.9925-0.9940) | **0.9974** (0.9969-0.9978) | **0.9988** (0.9985-0.9991) | **0.9996** (0.9993-0.9997) | **0.9999** (0.9997-0.9999) | **0.9999** (0.9998-1.0000) | **1.0000** (0.9999-1.0000) | **1.0000** (0.9999-1.0000) | **1.0000** (0.9999-1.0000) | **1.0000** (0.9999-1.0000) | **1.0000** (0.9999-1.0000) | **1.0000** (0.9999-1.0000) |
| Friston | **0.7089** (0.7049-0.7129) | **0.6996** (0.6955-0.7036) | **0.6947** (0.6906-0.6987) | **0.6891** (0.6851-0.6932) | **0.6822** (0.6781-0.6863) | **0.6761** (0.6720-0.6802) | **0.6729** (0.6688-0.6770) | **0.6698** (0.6657-0.6739) | **0.6675** (0.6634-0.6716) | **0.6664** (0.6623-0.6706) | **0.6671** (0.6629-0.6712) | **0.6609** (0.6568-0.6651) | **0.6591** (0.6550-0.6633) | **0.6564** (0.6522-0.6605) | **0.6572** (0.6530-0.6613) |
| Darlington-Hayes | **0.8610** (0.8579-0.8640) | **0.9498** (0.9478-0.9517) | **0.9775** (0.9762-0.9788) | **0.9920** (0.9912-0.9928) | **0.9955** (0.9949-0.9961) | **0.9984** (0.9980-0.9987) | **0.9994** (0.9991-0.9995) | **0.9998** (0.9996-0.9999) | **0.9999** (0.9998-1.0000) | **1.0000** (0.9999-1.0000) | **1.0000** (0.9999-1.0000) | **1.0000** (0.9999-1.0000) | **1.0000** (0.9999-1.0000) | **1.0000** (0.9999-1.0000) | **1.0000** (0.9999-1.0000) |
| Zaykin | **0.8779** (0.8750-0.8808) | **0.9501** (0.9481-0.9519) | **0.9796** (0.9783-0.9808) | **0.9915** (0.9907-0.9923) | **0.9963** (0.9957-0.9968) | **0.9983** (0.9979-0.9986) | **0.9993** (0.9990-0.9995) | **0.9998** (0.9996-0.9999) | **0.9999** (0.9998-1.0000) | **1.0000** (0.9999-1.0000) | **1.0000** (0.9999-1.0000) | **1.0000** (0.9999-1.0000) | **1.0000** (0.9999-1.0000) | **1.0000** (0.9999-1.0000) | **1.0000** (0.9999-1.0000) |
| Dudbridge-Koeleman | **0.8606** (0.8576-0.8636) | **0.9486** (0.9466-0.9505) | **0.9768** (0.9754-0.9781) | **0.9911** (0.9902-0.9918) | **0.9953** (0.9947-0.9959) | **0.9981** (0.9977-0.9985) | **0.9993** (0.9990-0.9995) | **0.9997** (0.9995-0.9998) | **0.9999** (0.9997-0.9999) | **1.0000** (0.9999-1.0000) | **1.0000** (0.9999-1.0000) | **1.0000** (0.9999-1.0000) | **1.0000** (0.9999-1.0000) | **1.0000** (0.9999-1.0000) | **1.0000** (0.9999-1.0000) |
| Dudbridge-Koeleman2 | **0.8608** (0.8578-0.8638) | **0.9484** (0.9464-0.9503) | **0.9765** (0.9751-0.9778) | **0.9912** (0.9903-0.9919) | **0.9957** (0.9951-0.9962) | **0.9982** (0.9978-0.9986) | **0.9991** (0.9988-0.9993) | **0.9997** (0.9996-0.9998) | **0.9999** (0.9998-1.0000) | **1.0000** (0.9999-1.0000) | **1.0000** (0.9999-1.0000) | **1.0000** (0.9999-1.0000) | **1.0000** (0.9999-1.0000) | **1.0000** (0.9999-1.0000) | **1.0000** (0.9999-1.0000) |
| Taylor-Tibshirani | **0.7888** (0.7852-0.7923) | **0.8913** (0.8886-0.8940) | **0.9542** (0.9523-0.9560) | **0.9790** (0.9777-0.9802) | **0.9908** (0.9899-0.9916) | **0.9959** (0.9953-0.9964) | **0.9979** (0.9975-0.9983) | **0.9992** (0.9989-0.9994) | **0.9998** (0.9996-0.9999) | **0.9998** (0.9997-0.9999) | **0.9999** (0.9997-0.9999) | **1.0000** (0.9999-1.0000) | **0.9999** (0.9998-1.0000) | **1.0000** (0.9999-1.0000) | **1.0000** (0.9999-1.0000) |
| Jiang | **0.8293** (0.8260-0.8326) | **0.9086** (0.9061-0.9111) | **0.9487** (0.9467-0.9506) | **0.9733** (0.9718-0.9747) | **0.9876** (0.9866-0.9885) | **0.9942** (0.9935-0.9949) | **0.9971** (0.9966-0.9975) | **0.9988** (0.9985-0.9991) | **0.9995** (0.9992-0.9996) | **0.9998** (0.9996-0.9999) | **0.9999** (0.9997-0.9999) | **1.0000** (0.9999-1.0000) | **1.0000** (0.9999-1.0000) | **1.0000** (0.9999-1.0000) | **1.0000** (0.9999-1.0000) |
| Hotelling | **0.8160** (0.8126-0.8194) | **0.9047** (0.9021-0.9072) | **0.9484** (0.9465-0.9503) | **0.9703** (0.9687-0.9717) | **0.9810** (0.9797-0.9821) | **0.9877** (0.9867-0.9886) | **0.9906** (0.9897-0.9914) | **0.9930** (0.9922-0.9937) | **0.9942** (0.9935-0.9949) | **0.9944** (0.9937-0.9950) | **0.9941** (0.9934-0.9947) | **0.9918** (0.9910-0.9926) | **0.9879** (0.9869-0.9888) | **0.9755** (0.9741-0.9768) | **0.9342** (0.9320-0.9363) |
| Wilks | **0.8163** (0.8129-0.8197) | **0.9055** (0.9029-0.9081) | **0.9486** (0.9466-0.9505) | **0.9701** (0.9686-0.9715) | **0.9811** (0.9798-0.9822) | **0.9873** (0.9863-0.9882) | **0.9909** (0.9901-0.9917) | **0.9931** (0.9923-0.9938) | **0.9940** (0.9933-0.9947) | **0.9943** (0.9936-0.9949) | **0.9944** (0.9937-0.9950) | **0.9919** (0.9911-0.9927) | **0.9881** (0.9871-0.9890) | **0.9758** (0.9744-0.9771) | **0.9345** (0.9323-0.9366) |
