# Supplementary material for: Non‐parametric combination and related permutation tests for neuroimaging
Source: Hum Brain Mapp. 2016 Feb 5;37(4):1486–511. doi: 10.1002/hbm.23115 (PMC4783210; doi:10.1002/hbm.23115)
Supplement: Supplementary file 1 — Supporting Information [file HBM-37-1486-s001.zip › supporting_information/errorrates/histograms/simD_Weibull_ise_uncp.html]

Sim D, Weibull, ISE, uncp

# Simulations D, Weibull, ISE, uncp

---

| Method | ksig=2 / K=2 | ksig=3 / K=3 | ksig=4 / K=4 | ksig=5 / K=5 | ksig=6 / K=6 | ksig=7 / K=7 | ksig=8 / K=8 | ksig=9 / K=9 | ksig=10 / K=10 | ksig=11 / K=11 | ksig=12 / K=12 | ksig=13 / K=13 | ksig=14 / K=14 | ksig=15 / K=15 | ksig=16 / K=16 |
| --- | --- | --- | --- | --- | --- | --- | --- | --- | --- | --- | --- | --- | --- | --- | --- |
| Tippett | **0.8602** (0.8572-0.8632) | **0.9193** (0.9168-0.9216) | **0.9511** (0.9492-0.9530) | **0.9667** (0.9651-0.9682) | **0.9767** (0.9753-0.9779) | **0.9847** (0.9836-0.9858) | **0.9888** (0.9879-0.9897) | **0.9921** (0.9912-0.9928) | **0.9943** (0.9936-0.9950) | **0.9959** (0.9953-0.9965) | **0.9968** (0.9963-0.9973) | **0.9974** (0.9970-0.9978) | **0.9983** (0.9979-0.9986) | **0.9987** (0.9983-0.9990) | **0.9988** (0.9985-0.9991) |
| Fisher | **0.8703** (0.8673-0.8732) | **0.9403** (0.9382-0.9423) | **0.9718** (0.9703-0.9732) | **0.9864** (0.9853-0.9873) | **0.9931** (0.9923-0.9938) | **0.9964** (0.9958-0.9969) | **0.9981** (0.9977-0.9985) | **0.9991** (0.9988-0.9993) | **0.9996** (0.9994-0.9997) | **0.9998** (0.9996-0.9999) | **0.9998** (0.9997-0.9999) | **0.9999** (0.9998-1.0000) | **0.9999** (0.9998-1.0000) | **0.9999** (0.9998-1.0000) | **1.0000** (0.9999-1.0000) |
| Stouffer | **0.8531** (0.8500-0.8562) | **0.9292** (0.9270-0.9315) | **0.9654** (0.9637-0.9669) | **0.9831** (0.9820-0.9842) | **0.9910** (0.9901-0.9918) | **0.9959** (0.9953-0.9964) | **0.9978** (0.9974-0.9982) | **0.9989** (0.9986-0.9992) | **0.9994** (0.9992-0.9996) | **0.9997** (0.9995-0.9998) | **0.9998** (0.9996-0.9999) | **0.9999** (0.9998-1.0000) | **0.9999** (0.9997-0.9999) | **1.0000** (0.9999-1.0000) | **0.9999** (0.9998-1.0000) |
| Wilkinson | **0.0607** (0.0587-0.0629) | **0.1491** (0.1460-0.1522) | **0.2345** (0.2308-0.2383) | **0.3231** (0.3190-0.3272) | **0.4051** (0.4008-0.4095) | **0.4861** (0.4817-0.4905) | **0.5332** (0.5289-0.5376) | **0.5913** (0.5870-0.5956) | **0.6463** (0.6421-0.6505) | **0.6858** (0.6817-0.6899) | **0.7284** (0.7245-0.7323) | **0.7531** (0.7493-0.7568) | **0.7794** (0.7757-0.7830) | **0.8087** (0.8052-0.8121) | **0.8363** (0.8331-0.8395) |
| Winer | **0.8601** (0.8570-0.8631) | **0.9334** (0.9312-0.9355) | **0.9691** (0.9675-0.9705) | **0.9849** (0.9838-0.9859) | **0.9927** (0.9919-0.9934) | **0.9963** (0.9957-0.9968) | **0.9981** (0.9976-0.9984) | **0.9990** (0.9987-0.9993) | **0.9995** (0.9992-0.9996) | **0.9998** (0.9996-0.9999) | **0.9999** (0.9997-0.9999) | **0.9999** (0.9998-1.0000) | **0.9999** (0.9998-1.0000) | **1.0000** (0.9999-1.0000) | **1.0000** (0.9999-1.0000) |
| Edgington | **0.7142** (0.7102-0.7181) | **0.7333** (0.7294-0.7372) | **0.7814** (0.7778-0.7850) | **0.8323** (0.8290-0.8356) | **0.8712** (0.8682-0.8741) | **0.8979** (0.8952-0.9005) | **0.9245** (0.9222-0.9268) | **0.9395** (0.9374-0.9416) | **0.9518** (0.9499-0.9536) | **0.9634** (0.9617-0.9650) | **0.9705** (0.9690-0.9719) | **0.9755** (0.9741-0.9768) | **0.9805** (0.9793-0.9817) | **0.9846** (0.9835-0.9856) | **0.9875** (0.9865-0.9885) |
| Mudholkar-George | **0.8649** (0.8619-0.8678) | **0.9376** (0.9355-0.9397) | **0.9701** (0.9686-0.9716) | **0.9857** (0.9846-0.9867) | **0.9929** (0.9921-0.9936) | **0.9965** (0.9960-0.9970) | **0.9983** (0.9979-0.9986) | **0.9992** (0.9989-0.9994) | **0.9995** (0.9993-0.9997) | **0.9997** (0.9995-0.9998) | **0.9999** (0.9997-0.9999) | **0.9999** (0.9998-1.0000) | **1.0000** (0.9999-1.0000) | **1.0000** (0.9999-1.0000) | **1.0000** (0.9999-1.0000) |
| Friston | **0.6748** (0.6707-0.6789) | **0.6409** (0.6367-0.6451) | **0.6190** (0.6147-0.6232) | **0.6055** (0.6012-0.6098) | **0.5973** (0.5930-0.6016) | **0.5969** (0.5926-0.6012) | **0.5948** (0.5905-0.5991) | **0.5979** (0.5936-0.6022) | **0.5957** (0.5914-0.6000) | **0.5979** (0.5936-0.6022) | **0.5976** (0.5933-0.6019) | **0.6002** (0.5959-0.6045) | **0.5981** (0.5938-0.6024) | **0.6010** (0.5967-0.6053) | **0.6010** (0.5967-0.6053) |
| Darlington-Hayes | **0.8607** (0.8577-0.8637) | **0.9377** (0.9356-0.9398) | **0.9682** (0.9666-0.9697) | **0.9846** (0.9835-0.9857) | **0.9919** (0.9911-0.9927) | **0.9963** (0.9957-0.9968) | **0.9978** (0.9973-0.9982) | **0.9989** (0.9986-0.9992) | **0.9993** (0.9991-0.9995) | **0.9996** (0.9993-0.9997) | **0.9998** (0.9997-0.9999) | **0.9999** (0.9998-1.0000) | **0.9999** (0.9998-1.0000) | **0.9999** (0.9998-1.0000) | **1.0000** (0.9999-1.0000) |
| Zaykin | **0.8663** (0.8633-0.8693) | **0.9368** (0.9346-0.9389) | **0.9696** (0.9681-0.9711) | **0.9840** (0.9829-0.9851) | **0.9921** (0.9913-0.9928) | **0.9958** (0.9952-0.9963) | **0.9977** (0.9972-0.9980) | **0.9988** (0.9984-0.9991) | **0.9994** (0.9992-0.9996) | **0.9996** (0.9994-0.9997) | **0.9999** (0.9998-1.0000) | **1.0000** (0.9999-1.0000) | **1.0000** (0.9999-1.0000) | **1.0000** (0.9999-1.0000) | **1.0000** (0.9999-1.0000) |
| Dudbridge-Koeleman | **0.8605** (0.8574-0.8635) | **0.9383** (0.9361-0.9404) | **0.9666** (0.9649-0.9681) | **0.9844** (0.9833-0.9855) | **0.9908** (0.9899-0.9916) | **0.9959** (0.9953-0.9964) | **0.9976** (0.9972-0.9980) | **0.9989** (0.9986-0.9992) | **0.9993** (0.9990-0.9995) | **0.9996** (0.9994-0.9998) | **0.9998** (0.9996-0.9999) | **0.9999** (0.9997-0.9999) | **0.9999** (0.9998-1.0000) | **0.9999** (0.9998-1.0000) | **0.9999** (0.9998-1.0000) |
| Dudbridge-Koeleman2 | **0.8593** (0.8563-0.8624) | **0.9380** (0.9358-0.9401) | **0.9671** (0.9655-0.9687) | **0.9845** (0.9834-0.9855) | **0.9911** (0.9902-0.9918) | **0.9961** (0.9955-0.9966) | **0.9976** (0.9971-0.9980) | **0.9989** (0.9986-0.9992) | **0.9992** (0.9990-0.9994) | **0.9995** (0.9993-0.9997) | **0.9998** (0.9996-0.9999) | **0.9999** (0.9998-1.0000) | **0.9999** (0.9998-1.0000) | **0.9999** (0.9998-1.0000) | **1.0000** (0.9999-1.0000) |
| Taylor-Tibshirani | **0.7337** (0.7298-0.7375) | **0.7850** (0.7814-0.7886) | **0.8534** (0.8503-0.8565) | **0.8987** (0.8960-0.9013) | **0.9268** (0.9245-0.9290) | **0.9493** (0.9474-0.9512) | **0.9626** (0.9609-0.9642) | **0.9741** (0.9727-0.9754) | **0.9803** (0.9790-0.9815) | **0.9863** (0.9853-0.9873) | **0.9886** (0.9877-0.9895) | **0.9919** (0.9911-0.9927) | **0.9927** (0.9919-0.9934) | **0.9952** (0.9946-0.9958) | **0.9954** (0.9948-0.9960) |
| Jiang | **0.7574** (0.7536-0.7611) | **0.8044** (0.8009-0.8079) | **0.8451** (0.8419-0.8483) | **0.8754** (0.8725-0.8782) | **0.8997** (0.8971-0.9023) | **0.9210** (0.9186-0.9233) | **0.9382** (0.9361-0.9403) | **0.9495** (0.9475-0.9514) | **0.9593** (0.9575-0.9610) | **0.9645** (0.9628-0.9660) | **0.9714** (0.9699-0.9728) | **0.9773** (0.9759-0.9785) | **0.9817** (0.9805-0.9828) | **0.9839** (0.9827-0.9849) | **0.9863** (0.9853-0.9873) |
| Hotelling | **0.8176** (0.8142-0.8210) | **0.8929** (0.8902-0.8956) | **0.9335** (0.9313-0.9357) | **0.9565** (0.9547-0.9583) | **0.9671** (0.9655-0.9687) | **0.9758** (0.9744-0.9771) | **0.9805** (0.9792-0.9816) | **0.9816** (0.9804-0.9827) | **0.9827** (0.9815-0.9838) | **0.9816** (0.9804-0.9827) | **0.9801** (0.9788-0.9813) | **0.9753** (0.9739-0.9767) | **0.9630** (0.9613-0.9646) | **0.9411** (0.9390-0.9431) | **0.8816** (0.8787-0.8844) |
| Wilks | **0.8175** (0.8141-0.8209) | **0.8928** (0.8901-0.8955) | **0.9337** (0.9315-0.9358) | **0.9560** (0.9541-0.9577) | **0.9671** (0.9655-0.9686) | **0.9757** (0.9743-0.9770) | **0.9799** (0.9787-0.9811) | **0.9814** (0.9802-0.9826) | **0.9827** (0.9815-0.9838) | **0.9817** (0.9805-0.9829) | **0.9800** (0.9788-0.9812) | **0.9747** (0.9733-0.9760) | **0.9644** (0.9627-0.9659) | **0.9400** (0.9379-0.9421) | **0.8816** (0.8788-0.8844) |
