# Supplementary material for: Non‐parametric combination and related permutation tests for neuroimaging
Source: Hum Brain Mapp. 2016 Feb 5;37(4):1486–511. doi: 10.1002/hbm.23115 (PMC4783210; doi:10.1002/hbm.23115)
Supplement: Supplementary file 1 — Supporting Information [file HBM-37-1486-s001.zip › supporting_information/pain/pain_vox_fwep.html]

vox, fwep

# Pain study, Voxel-level, Corrected

---

## Not combined

Face:

Hand:

Foot:

---

## Combined

Tippett:

Fisher:

Stouffer:

Edgington:

Mudholkar–George:

Friston:

IUT/Conjunction/Nichols:

Hotelling *T*^2^:
