# Supplementary material for: Non‐parametric combination and related permutation tests for neuroimaging
Source: Hum Brain Mapp. 2016 Feb 5;37(4):1486–511. doi: 10.1002/hbm.23115 (PMC4783210; doi:10.1002/hbm.23115)
Supplement: Supplementary file 1 — Supporting Information [file HBM-37-1486-s001.zip › supporting_information/supporting_information.html]

Combining/Correcting


# Non-Parametric Combination and related permutation tests for neuroimaging

# Supporting Information

Winkler AM, Webster MA, Brooks JC, Tracey I, Smith SM, Nichols TE.

---

## 1. Validity of the modified NPC

|  | Tippett | | Fisher | |
| --- | --- | --- | --- | --- |
| Set 1 | Histograms | Bland–Altman | Histograms | Bland–Altman |
| Set 2 | Histograms | Bland–Altman | Histograms | Bland–Altman |
| Set 3 | Histograms | Bland–Altman | Histograms | Bland–Altman |
| Set 4 | Histograms | Bland–Altman | Histograms | Bland–Altman |

---

## 2. Error rates and power

| Error distribution | Simulations A | | | Simulations B | | | Simulations C | | | Simulations D | | |
| --- | --- | --- | --- | --- | --- | --- | --- | --- | --- | --- | --- | --- |
| Gauss | EE | ISE | EE+ISE | EE | ISE | EE+ISE | EE | ISE | EE+ISE | EE | ISE | EE+ISE |
| Weibull | EE | ISE | EE+ISE | EE | ISE | EE+ISE | EE | ISE | EE+ISE | EE | ISE | EE+ISE |

Line plots with just the first bars of each histograms:

| Error distribution |  | Simulations A | Simulations B | Simulations C | Simulations D |
| --- | --- | --- | --- | --- | --- |
| Gauss | EE |  |  |  |  |
|  | ISE |  |  |  |  |
|  | EE+ISE |  |  |  |  |
| Weibull | EE |  |  |  |  |
|  | ISE |  |  |  |  |
|  | EE+ISE |  |  |  |  |

---

## 3. Pain study

|  |  |  |
| --- | --- | --- |
| Voxel-level | uncp | fwep |
| Cluster extent | — | fwep |
| TFCE | uncp | fwep |

---

## 4. Implementation

Click **here** for the unified algorithm.

---
