# Supplementary material for: Non‐parametric combination and related permutation tests for neuroimaging
Source: Hum Brain Mapp. 2016 Feb 5;37(4):1486–511. doi: 10.1002/hbm.23115 (PMC4783210; doi:10.1002/hbm.23115)
Supplement: Supplementary file 1 — Supporting Information [file HBM-37-1486-s001.zip › supporting_information/validity/ba_Fisher_set1.html]

Fisher, Set 1

# Fisher, Set 1

---

|  | Uncorrected | | | Corrected | | |
| --- | --- | --- | --- | --- | --- | --- |
| N | Gaussian–Gaussian | Weibull–Weibull | Gaussian–Weibull | Gaussian–Gaussian | Weibull–Weibull | Gaussian–Weibull |
| 6 | 78.9796% | 55.4048% | 58.7732% | 98.3984% | 98.2804% | 98.378% |
| 8 | 90.4072% | 65.3776% | 70.2868% | 98.6% | 98.4044% | 98.5268% |
| 12 | 95.062% | 76.4268% | 80.3448% | 98.7724% | 98.6108% | 98.6092% |
| 20 | 96.6052% | 85.182% | 88.2604% | 98.796% | 98.6732% | 98.7228% |
| 30 | 96.804% | 89.7404% | 91.8408% | 98.8156% | 98.7932% | 98.7064% |
| 40 | 96.8668% | 92.4648% | 93.7748% | 98.7972% | 98.7592% | 98.8036% |
| 50 | 96.9168% | 93.6696% | 94.3892% | 98.7976% | 98.7748% | 98.7648% |
| 60 | 96.9108% | 94.6168% | 95.2376% | 98.8188% | 98.8224% | 98.7992% |
| 80 | 96.9712% | 95.488% | 95.8948% | 98.8068% | 98.798% | 98.7756% |
| 120 | 96.972% | 96.1996% | 96.3932% | 98.8228% | 98.8124% | 98.804% |
| 200 | 96.9996% | 96.66% | 96.7236% | 98.8116% | 98.838% | 98.8168% |
