# Supplementary material for: Non‐parametric combination and related permutation tests for neuroimaging
Source: Hum Brain Mapp. 2016 Feb 5;37(4):1486–511. doi: 10.1002/hbm.23115 (PMC4783210; doi:10.1002/hbm.23115)
Supplement: Supplementary file 1 — Supporting Information [file HBM-37-1486-s001.zip › supporting_information/validity/ba_Fisher_set2.html]

Fisher, Set 2

# Fisher, Set 2

---

|  | Uncorrected | | | Corrected | | |
| --- | --- | --- | --- | --- | --- | --- |
| N | Gaussian–Gaussian | Weibull–Weibull | Gaussian–Weibull | Gaussian–Gaussian | Weibull–Weibull | Gaussian–Weibull |
| 6 | 67.8772% | 58.332% | 49.7264% | 75.2096% | 17.5896% | 67.422% |
| 8 | 81.7496% | 68.4184% | 61.162% | 78.126% | 18.9392% | 69.8504% |
| 12 | 87.8424% | 67.6152% | 71.2392% | 79.334% | 22.3384% | 72.854% |
| 20 | 89.4276% | 65.2884% | 79.4124% | 79.778% | 27.856% | 76.9432% |
| 30 | 89.6148% | 64.7776% | 83.406% | 80.7252% | 31.9908% | 78.7244% |
| 40 | 89.5576% | 65.4252% | 85.4204% | 81.3932% | 35.4016% | 80.012% |
| 50 | 89.5072% | 66.7788% | 85.8464% | 81.716% | 38.5752% | 80.2632% |
| 60 | 89.538% | 67.9668% | 87.2168% | 82.1692% | 41.2248% | 81.072% |
| 80 | 89.3672% | 70.248% | 87.818% | 81.9252% | 45.5864% | 81.5264% |
| 120 | 89.2364% | 73.4544% | 88.4056% | 82.3968% | 51.7776% | 82.2384% |
| 200 | 89.0964% | 77.3724% | 88.7172% | 82.1824% | 59.3328% | 82.2844% |
