# Supplementary material for: Non‐parametric combination and related permutation tests for neuroimaging
Source: Hum Brain Mapp. 2016 Feb 5;37(4):1486–511. doi: 10.1002/hbm.23115 (PMC4783210; doi:10.1002/hbm.23115)
Supplement: Supplementary file 1 — Supporting Information [file HBM-37-1486-s001.zip › supporting_information/validity/ba_Fisher_set3.html]

Fisher, Set 3

# Fisher, Set 3

---

|  | Uncorrected | | | Corrected | | |
| --- | --- | --- | --- | --- | --- | --- |
| N | Gaussian–Gaussian | Weibull–Weibull | Gaussian–Weibull | Gaussian–Gaussian | Weibull–Weibull | Gaussian–Weibull |
| 6 | 67.8852% | 58.1952% | 64.1268% | 75.3052% | 17.3788% | 21.2368% |
| 8 | 81.7452% | 68.2728% | 76.736% | 78.2204% | 18.9744% | 22.6964% |
| 12 | 87.8416% | 67.532% | 72.116% | 79.5632% | 22.1948% | 24.0232% |
| 20 | 89.4416% | 65.3916% | 66.6272% | 79.79% | 27.9128% | 27.9916% |
| 30 | 89.5652% | 64.7984% | 65.0716% | 80.7328% | 31.9616% | 32.2592% |
| 40 | 89.5992% | 65.7552% | 66.2644% | 81.3604% | 35.3168% | 35.8588% |
| 50 | 89.604% | 66.7448% | 67.028% | 81.8048% | 38.4108% | 38.656% |
| 60 | 89.382% | 67.9704% | 68.1156% | 81.9376% | 40.9888% | 41.5664% |
| 80 | 89.1904% | 70.232% | 70.5348% | 81.8096% | 45.5168% | 45.6152% |
| 120 | 89.2724% | 73.4212% | 73.3556% | 82.414% | 51.6944% | 51.9668% |
| 200 | 89.1028% | 77.5348% | 77.0472% | 82.3816% | 59.414% | 58.852% |
