# Supplementary material for: Non‐parametric combination and related permutation tests for neuroimaging
Source: Hum Brain Mapp. 2016 Feb 5;37(4):1486–511. doi: 10.1002/hbm.23115 (PMC4783210; doi:10.1002/hbm.23115)
Supplement: Supplementary file 1 — Supporting Information [file HBM-37-1486-s001.zip › supporting_information/validity/ba_Fisher_set4.html]

Fisher, Set 4

# Fisher, Set 4

---

|  | Uncorrected | | | Corrected | | |
| --- | --- | --- | --- | --- | --- | --- |
| N | Gaussian–Gaussian | Weibull–Weibull | Gaussian–Weibull | Gaussian–Gaussian | Weibull–Weibull | Gaussian–Weibull |
| 6 | 77.3272% | 82.8644% | 72.0912% | 43.284% | 24.8988% | 18.5076% |
| 8 | 88.5908% | 85.9084% | 81.3884% | 50.3932% | 21.4032% | 20.1124% |
| 12 | 93.1696% | 85.9264% | 83.3016% | 52.498% | 26.3892% | 20.798% |
| 20 | 94.6864% | 87.1448% | 85.3844% | 51.784% | 36.2976% | 23.4044% |
| 30 | 94.8712% | 88.792% | 87.1132% | 52.1076% | 38.9344% | 25.5832% |
| 40 | 94.9596% | 89.7524% | 88.5832% | 52.5776% | 40.0372% | 28.254% |
| 50 | 95.004% | 90.452% | 89.342% | 53.0376% | 38.832% | 29.8016% |
| 60 | 94.984% | 91.0292% | 89.9624% | 53.6152% | 39.0564% | 31.4444% |
| 80 | 95.0596% | 91.652% | 91.154% | 53.324% | 38.5128% | 34.0652% |
| 120 | 95.056% | 92.4504% | 92.1184% | 54.25% | 38.3536% | 37.6468% |
| 200 | 95.1028% | 93.0104% | 93.0292% | 54.6344% | 39.062% | 41.7264% |
