# Supplementary material for: Non‐parametric combination and related permutation tests for neuroimaging
Source: Hum Brain Mapp. 2016 Feb 5;37(4):1486–511. doi: 10.1002/hbm.23115 (PMC4783210; doi:10.1002/hbm.23115)
Supplement: Supplementary file 1 — Supporting Information [file HBM-37-1486-s001.zip › supporting_information/validity/ba_Tippett_set1.html]

Tippett, Set 1

# Tippett, Set 1

---

|  | Uncorrected | | | Corrected | | |
| --- | --- | --- | --- | --- | --- | --- |
| N | Gaussian–Gaussian | Weibull–Weibull | Gaussian–Weibull | Gaussian–Gaussian | Weibull–Weibull | Gaussian–Weibull |
| 6 | 79.9556% | 64.5804% | 60.0564% | 98.2088% | 97.9504% | 98.134% |
| 8 | 89.7128% | 74.728% | 70.06% | 98.59% | 98.2804% | 98.4948% |
| 12 | 94.0628% | 84.0532% | 79.3744% | 98.8352% | 98.5296% | 98.6132% |
| 20 | 95.8472% | 90.1392% | 86.9752% | 98.888% | 98.6684% | 98.7552% |
| 30 | 96.22% | 92.6596% | 90.7216% | 98.8952% | 98.7588% | 98.73% |
| 40 | 96.1416% | 94.0416% | 92.7028% | 98.9004% | 98.778% | 98.8364% |
| 50 | 96.2664% | 94.6772% | 93.3648% | 98.8836% | 98.7652% | 98.8188% |
| 60 | 96.3612% | 95.1648% | 94.2984% | 98.906% | 98.8528% | 98.874% |
| 80 | 96.3792% | 95.6008% | 95.0744% | 98.8852% | 98.8244% | 98.834% |
| 120 | 96.3288% | 95.9148% | 95.5584% | 98.8832% | 98.8792% | 98.902% |
| 200 | 96.3864% | 96.182% | 95.9932% | 98.8924% | 98.9212% | 98.8864% |
