# Supplementary material for: Non‐parametric combination and related permutation tests for neuroimaging
Source: Hum Brain Mapp. 2016 Feb 5;37(4):1486–511. doi: 10.1002/hbm.23115 (PMC4783210; doi:10.1002/hbm.23115)
Supplement: Supplementary file 1 — Supporting Information [file HBM-37-1486-s001.zip › supporting_information/validity/ba_Tippett_set2.html]

Tippett, Set 2

# Tippett, Set 2

---

|  | Uncorrected | | | Corrected | | |
| --- | --- | --- | --- | --- | --- | --- |
| N | Gaussian–Gaussian | Weibull–Weibull | Gaussian–Weibull | Gaussian–Gaussian | Weibull–Weibull | Gaussian–Weibull |
| 6 | 67.732% | 50.2024% | 42.766% | 81.4344% | 18.566% | 72.4728% |
| 8 | 82.0384% | 75.3124% | 52.9828% | 82.1224% | 19.6708% | 73.192% |
| 12 | 89.3168% | 85.3932% | 64.6652% | 81.8408% | 22.8136% | 74.3312% |
| 20 | 92.0892% | 89.9248% | 75.4896% | 81.2216% | 27.794% | 76.8808% |
| 30 | 92.4968% | 91.72% | 81.7204% | 81.4456% | 31.5572% | 78.3956% |
| 40 | 92.6172% | 92.4072% | 85.0892% | 81.5348% | 34.6276% | 79.234% |
| 50 | 92.5992% | 92.6684% | 86.0172% | 81.6052% | 37.6576% | 79.5036% |
| 60 | 92.7632% | 92.714% | 88.3016% | 81.8232% | 40.1824% | 80.4756% |
| 80 | 92.6768% | 92.6412% | 89.6688% | 81.5168% | 44.2968% | 80.7896% |
| 120 | 92.7012% | 92.3876% | 91.0708% | 81.7336% | 49.9328% | 81.4068% |
| 200 | 92.6872% | 91.9172% | 91.894% | 81.4212% | 56.9148% | 81.536% |
