# Supplementary material for: Non‐parametric combination and related permutation tests for neuroimaging
Source: Hum Brain Mapp. 2016 Feb 5;37(4):1486–511. doi: 10.1002/hbm.23115 (PMC4783210; doi:10.1002/hbm.23115)
Supplement: Supplementary file 1 — Supporting Information [file HBM-37-1486-s001.zip › supporting_information/validity/ba_Tippett_set3.html]

Tippett, Set 3

# Tippett, Set 3

---

|  | Uncorrected | | | Corrected | | |
| --- | --- | --- | --- | --- | --- | --- |
| N | Gaussian–Gaussian | Weibull–Weibull | Gaussian–Weibull | Gaussian–Gaussian | Weibull–Weibull | Gaussian–Weibull |
| 6 | 67.6764% | 50.2344% | 55.6236% | 81.2372% | 18.5284% | 22.3428% |
| 8 | 81.9836% | 75.2928% | 83.196% | 82.3948% | 19.7604% | 22.7844% |
| 12 | 89.2772% | 85.3284% | 90.242% | 82.0192% | 22.6408% | 24.1732% |
| 20 | 91.944% | 90.0472% | 92.2172% | 80.9464% | 27.9104% | 28.13% |
| 30 | 92.5424% | 91.7488% | 92.132% | 81.2908% | 31.6032% | 32.0964% |
| 40 | 92.5844% | 92.4636% | 92.7116% | 81.6592% | 34.6092% | 35.5712% |
| 50 | 92.6608% | 92.6628% | 92.4712% | 81.722% | 37.5372% | 38.0944% |
| 60 | 92.79% | 92.7616% | 92.2608% | 81.7436% | 39.9496% | 40.876% |
| 80 | 92.5988% | 92.6588% | 92.5936% | 81.2884% | 44.1424% | 44.5864% |
| 120 | 92.656% | 92.3912% | 92.0316% | 81.7628% | 49.9816% | 50.2952% |
| 200 | 92.6356% | 91.9956% | 91.7528% | 81.3476% | 57.1964% | 56.78% |
