# Supplementary material for: Non‐parametric combination and related permutation tests for neuroimaging
Source: Hum Brain Mapp. 2016 Feb 5;37(4):1486–511. doi: 10.1002/hbm.23115 (PMC4783210; doi:10.1002/hbm.23115)
Supplement: Supplementary file 1 — Supporting Information [file HBM-37-1486-s001.zip › supporting_information/validity/ba_Tippett_set4.html]

Tippett, Set 4

# Tippett, Set 4

---

|  | Uncorrected | | | Corrected | | |
| --- | --- | --- | --- | --- | --- | --- |
| N | Gaussian–Gaussian | Weibull–Weibull | Gaussian–Weibull | Gaussian–Gaussian | Weibull–Weibull | Gaussian–Weibull |
| 6 | 68.5692% | 78.7736% | 60.3072% | 44.1756% | 5.1792% | 12.1884% |
| 8 | 81.726% | 92.2916% | 84.4252% | 50.8044% | 3.7972% | 13.3112% |
| 12 | 88.7516% | 95.35% | 90.0292% | 54.7924% | 3.9248% | 14.66% |
| 20 | 91.2508% | 95.6532% | 91.346% | 56.1328% | 5.6576% | 18.2392% |
| 30 | 91.6244% | 95.6364% | 91.2172% | 58.7216% | 7.55% | 21.5712% |
| 40 | 91.6272% | 95.4844% | 91.6912% | 60.6944% | 9.4728% | 24.8664% |
| 50 | 91.5932% | 95.236% | 91.4856% | 61.724% | 11.4664% | 27.1196% |
| 60 | 91.6056% | 94.9272% | 91.5624% | 62.7864% | 13.6324% | 29.9288% |
| 80 | 91.6568% | 94.5148% | 91.8672% | 62.8964% | 17.1748% | 33.392% |
| 120 | 91.5736% | 93.7536% | 91.5784% | 64.6452% | 23.1032% | 38.824% |
| 200 | 91.5004% | 92.5836% | 91.3116% | 65.1096% | 31.218% | 44.972% |
