# Supplementary material for: Non‐parametric combination and related permutation tests for neuroimaging
Source: Hum Brain Mapp. 2016 Feb 5;37(4):1486–511. doi: 10.1002/hbm.23115 (PMC4783210; doi:10.1002/hbm.23115)
Supplement: Supplementary file 1 — Supporting Information [file HBM-37-1486-s001.zip › supporting_information/validity/hist_Fisher_set1.html]

Fisher, Set 1

# Fisher, Set 1

---

|  | Original | | | Modified | | |
| --- | --- | --- | --- | --- | --- | --- |
| N | Gaussian–Gaussian | Weibull–Weibull | Gaussian–Weibull | Gaussian–Gaussian | Weibull–Weibull | Gaussian–Weibull |
| 6 | **0.04878** (0.0331–0.0713) | **0.04994** (0.0340–0.0727) | **0.04909** (0.0334–0.0717) | **0.04875** (0.0331–0.0713) | **0.04920** (0.0334–0.0718) | **0.04875** (0.0331–0.0713) |
| 8 | **0.05054** (0.0345–0.0734) | **0.05037** (0.0344–0.0732) | **0.04986** (0.0340–0.0726) | **0.05079** (0.0347–0.0737) | **0.04964** (0.0338–0.0724) | **0.04994** (0.0340–0.0727) |
| 12 | **0.04973** (0.0339–0.0725) | **0.05020** (0.0343–0.0730) | **0.05034** (0.0344–0.0732) | **0.04970** (0.0339–0.0724) | **0.05043** (0.0344–0.0733) | **0.05012** (0.0342–0.0729) |
| 20 | **0.04950** (0.0337–0.0722) | **0.04996** (0.0341–0.0727) | **0.04954** (0.0337–0.0722) | **0.04959** (0.0338–0.0723) | **0.05024** (0.0343–0.0730) | **0.04986** (0.0340–0.0726) |
| 30 | **0.04952** (0.0337–0.0722) | **0.04998** (0.0341–0.0727) | **0.04981** (0.0339–0.0725) | **0.04936** (0.0336–0.0720) | **0.05002** (0.0341–0.0728) | **0.05012** (0.0342–0.0729) |
| 40 | **0.05015** (0.0342–0.0729) | **0.05079** (0.0347–0.0737) | **0.04986** (0.0340–0.0726) | **0.05050** (0.0345–0.0734) | **0.05074** (0.0347–0.0736) | **0.04984** (0.0340–0.0726) |
| 50 | **0.04971** (0.0339–0.0724) | **0.04983** (0.0340–0.0726) | **0.04971** (0.0339–0.0724) | **0.04964** (0.0338–0.0723) | **0.04992** (0.0340–0.0727) | **0.04971** (0.0339–0.0724) |
| 60 | **0.04976** (0.0339–0.0725) | **0.05006** (0.0341–0.0728) | **0.04910** (0.0334–0.0717) | **0.04991** (0.0340–0.0727) | **0.04976** (0.0339–0.0725) | **0.04888** (0.0332–0.0715) |
| 80 | **0.05095** (0.0349–0.0739) | **0.05006** (0.0341–0.0728) | **0.05042** (0.0344–0.0733) | **0.05092** (0.0348–0.0738) | **0.05005** (0.0341–0.0728) | **0.05034** (0.0344–0.0732) |
| 120 | **0.04950** (0.0337–0.0722) | **0.04978** (0.0339–0.0725) | **0.04947** (0.0337–0.0721) | **0.04956** (0.0337–0.0723) | **0.05018** (0.0342–0.0730) | **0.04934** (0.0336–0.0720) |
| 200 | **0.05029** (0.0343–0.0731) | **0.05000** (0.0341–0.0728) | **0.04988** (0.0340–0.0726) | **0.05075** (0.0347–0.0736) | **0.04979** (0.0339–0.0725) | **0.04999** (0.0341–0.0728) |
