# Supplementary material for: Non‐parametric combination and related permutation tests for neuroimaging
Source: Hum Brain Mapp. 2016 Feb 5;37(4):1486–511. doi: 10.1002/hbm.23115 (PMC4783210; doi:10.1002/hbm.23115)
Supplement: Supplementary file 1 — Supporting Information [file HBM-37-1486-s001.zip › supporting_information/validity/hist_Fisher_set2.html]

Fisher, Set 2

# Fisher, Set 2

---

|  | Original | | | Modified | | |
| --- | --- | --- | --- | --- | --- | --- |
| N | Gaussian–Gaussian | Weibull–Weibull | Gaussian–Weibull | Gaussian–Gaussian | Weibull–Weibull | Gaussian–Weibull |
| 6 | **0.29423** (0.2560–0.3356) | **0.80806** (0.7712–0.8402) | **0.29483** (0.2566–0.3362) | **0.30751** (0.2687–0.3493) | **0.82406** (0.7882–0.8549) | **0.33088** (0.2911–0.3733) |
| 8 | **0.31942** (0.2801–0.3615) | **0.81409** (0.7776–0.8457) | **0.32466** (0.2851–0.3669) | **0.32654** (0.2869–0.3688) | **0.81865** (0.7825–0.8500) | **0.34772** (0.3073–0.3905) |
| 12 | **0.34039** (0.3002–0.3830) | **0.79842** (0.7610–0.8313) | **0.35101** (0.3105–0.3938) | **0.34542** (0.3051–0.3881) | **0.79818** (0.7608–0.8310) | **0.36380** (0.3228–0.4069) |
| 20 | **0.36202** (0.3211–0.4050) | **0.76800** (0.7290–0.8029) | **0.35748** (0.3167–0.4004) | **0.36594** (0.3249–0.4090) | **0.76632** (0.7273–0.8013) | **0.36517** (0.3241–0.4082) |
| 30 | **0.36432** (0.3233–0.4074) | **0.74476** (0.7048–0.7810) | **0.36330** (0.3223–0.4063) | **0.36839** (0.3273–0.4115) | **0.74412** (0.7041–0.7804) | **0.36668** (0.3256–0.4098) |
| 40 | **0.36421** (0.3232–0.4073) | **0.72541** (0.6847–0.7627) | **0.36423** (0.3232–0.4073) | **0.36895** (0.3278–0.4121) | **0.72654** (0.6858–0.7638) | **0.36889** (0.3277–0.4120) |
| 50 | **0.36442** (0.3234–0.4075) | **0.70697** (0.6656–0.7452) | **0.37009** (0.3289–0.4132) | **0.36864** (0.3275–0.4118) | **0.70938** (0.6681–0.7475) | **0.37504** (0.3337–0.4183) |
| 60 | **0.36259** (0.3216–0.4056) | **0.69166** (0.6498–0.7305) | **0.36712** (0.3260–0.4102) | **0.36720** (0.3261–0.4103) | **0.69438** (0.6526–0.7331) | **0.36995** (0.3288–0.4131) |
| 80 | **0.36919** (0.3280–0.4123) | **0.66650** (0.6240–0.7064) | **0.36968** (0.3285–0.4128) | **0.37402** (0.3327–0.4172) | **0.67012** (0.6277–0.7099) | **0.37416** (0.3329–0.4174) |
| 120 | **0.36958** (0.3284–0.4127) | **0.63069** (0.5875–0.6718) | **0.36962** (0.3285–0.4128) | **0.37437** (0.3331–0.4176) | **0.63506** (0.5920–0.6761) | **0.37358** (0.3323–0.4168) |
| 200 | **0.37694** (0.3356–0.4202) | **0.58285** (0.5392–0.6253) | **0.37384** (0.3325–0.4171) | **0.38150** (0.3400–0.4248) | **0.58744** (0.5438–0.6298) | **0.37813** (0.3367–0.4214) |
