# Supplementary material for: Non‐parametric combination and related permutation tests for neuroimaging
Source: Hum Brain Mapp. 2016 Feb 5;37(4):1486–511. doi: 10.1002/hbm.23115 (PMC4783210; doi:10.1002/hbm.23115)
Supplement: Supplementary file 1 — Supporting Information [file HBM-37-1486-s001.zip › supporting_information/validity/hist_Fisher_set3.html]

Fisher, Set 3

# Fisher, Set 3

---

|  | Original | | | Modified | | |
| --- | --- | --- | --- | --- | --- | --- |
| N | Gaussian–Gaussian | Weibull–Weibull | Gaussian–Weibull | Gaussian–Gaussian | Weibull–Weibull | Gaussian–Weibull |
| 6 | **0.29532** (0.2570–0.3367) | **0.80779** (0.7710–0.8399) | **0.80608** (0.7691–0.8383) | **0.30929** (0.2704–0.3511) | **0.82372** (0.7879–0.8546) | **0.81388** (0.7774–0.8456) |
| 8 | **0.31972** (0.2803–0.3618) | **0.81233** (0.7758–0.8441) | **0.81520** (0.7788–0.8468) | **0.32714** (0.2875–0.3694) | **0.81746** (0.7812–0.8489) | **0.81472** (0.7783–0.8463) |
| 12 | **0.33976** (0.2996–0.3824) | **0.79894** (0.7616–0.8317) | **0.80720** (0.7703–0.8394) | **0.34478** (0.3044–0.3875) | **0.79908** (0.7617–0.8319) | **0.80442** (0.7674–0.8368) |
| 20 | **0.36142** (0.3205–0.4044) | **0.76695** (0.7279–0.8019) | **0.77643** (0.7379–0.8108) | **0.36611** (0.3251–0.4092) | **0.76532** (0.7262–0.8004) | **0.77434** (0.7357–0.8088) |
| 30 | **0.36316** (0.3222–0.4062) | **0.74482** (0.7048–0.7811) | **0.74679** (0.7069–0.7829) | **0.36781** (0.3267–0.4109) | **0.74436** (0.7044–0.7806) | **0.74789** (0.7080–0.7840) |
| 40 | **0.36299** (0.3220–0.4060) | **0.72614** (0.6854–0.7634) | **0.72320** (0.6824–0.7606) | **0.36685** (0.3258–0.4100) | **0.72731** (0.6867–0.7645) | **0.72488** (0.6841–0.7622) |
| 50 | **0.36400** (0.3230–0.4071) | **0.70868** (0.6674–0.7468) | **0.70782** (0.6665–0.7460) | **0.36789** (0.3268–0.4110) | **0.71008** (0.6688–0.7481) | **0.70957** (0.6683–0.7476) |
| 60 | **0.36392** (0.3229–0.4070) | **0.69359** (0.6518–0.7324) | **0.68888** (0.6470–0.7279) | **0.36792** (0.3268–0.4110) | **0.69601** (0.6543–0.7347) | **0.69327** (0.6515–0.7321) |
| 80 | **0.37087** (0.3297–0.4140) | **0.66776** (0.6253–0.7076) | **0.66531** (0.6228–0.7053) | **0.37502** (0.3337–0.4183) | **0.67144** (0.6291–0.7112) | **0.66852** (0.6261–0.7084) |
| 120 | **0.37006** (0.3289–0.4132) | **0.63090** (0.5878–0.6721) | **0.62898** (0.5858–0.6702) | **0.37426** (0.3329–0.4175) | **0.63478** (0.5917–0.6758) | **0.63419** (0.5911–0.6752) |
| 200 | **0.37691** (0.3355–0.4202) | **0.58172** (0.5380–0.6242) | **0.58536** (0.5417–0.6277) | **0.38106** (0.3395–0.4244) | **0.58673** (0.5431–0.6291) | **0.59061** (0.5470–0.6329) |
