# Supplementary material for: Non‐parametric combination and related permutation tests for neuroimaging
Source: Hum Brain Mapp. 2016 Feb 5;37(4):1486–511. doi: 10.1002/hbm.23115 (PMC4783210; doi:10.1002/hbm.23115)
Supplement: Supplementary file 1 — Supporting Information [file HBM-37-1486-s001.zip › supporting_information/validity/hist_Fisher_set4.html]

Fisher, Set 4

# Fisher, Set 4

---

|  | Original | | | Modified | | |
| --- | --- | --- | --- | --- | --- | --- |
| N | Gaussian–Gaussian | Weibull–Weibull | Gaussian–Weibull | Gaussian–Gaussian | Weibull–Weibull | Gaussian–Weibull |
| 6 | **0.56589** (0.5221–0.6087) | **0.96611** (0.9464–0.9787) | **0.87749** (0.8458–0.9034) | **0.57717** (0.5334–0.6197) | **0.96715** (0.9476–0.9796) | **0.88054** (0.8492–0.9061) |
| 8 | **0.61663** (0.5733–0.6582) | **0.96855** (0.9493–0.9806) | **0.89168** (0.8614–0.9160) | **0.62134** (0.5781–0.6628) | **0.96835** (0.9491–0.9805) | **0.89127** (0.8609–0.9156) |
| 12 | **0.65337** (0.6106–0.6938) | **0.96570** (0.9459–0.9784) | **0.89789** (0.8682–0.9215) | **0.65562** (0.6129–0.6959) | **0.96469** (0.9446–0.9776) | **0.89712** (0.8674–0.9208) |
| 20 | **0.69481** (0.6531–0.7336) | **0.95605** (0.9343–0.9708) | **0.88634** (0.8555–0.9113) | **0.69524** (0.6535–0.7340) | **0.95454** (0.9326–0.9696) | **0.88634** (0.8555–0.9113) |
| 30 | **0.70072** (0.6592–0.7392) | **0.94900** (0.9261–0.9651) | **0.87790** (0.8463–0.9038) | **0.70138** (0.6599–0.7398) | **0.94768** (0.9245–0.9640) | **0.87724** (0.8456–0.9032) |
| 40 | **0.70181** (0.6603–0.7402) | **0.94387** (0.9201–0.9609) | **0.86917** (0.8368–0.8959) | **0.70232** (0.6608–0.7407) | **0.94282** (0.9189–0.9600) | **0.86947** (0.8371–0.8962) |
| 50 | **0.70585** (0.6645–0.7441) | **0.93727** (0.9125–0.9554) | **0.86438** (0.8316–0.8916) | **0.70594** (0.6646–0.7442) | **0.93692** (0.9121–0.9551) | **0.86433** (0.8315–0.8916) |
| 60 | **0.70302** (0.6615–0.7414) | **0.93117** (0.9055–0.9502) | **0.85743** (0.8241–0.8854) | **0.70324** (0.6618–0.7416) | **0.93053** (0.9048–0.9497) | **0.85775** (0.8244–0.8856) |
| 80 | **0.71416** (0.6730–0.7520) | **0.92130** (0.8944–0.9418) | **0.84862** (0.8146–0.8774) | **0.71475** (0.6737–0.7526) | **0.92108** (0.8941–0.9416) | **0.84896** (0.8149–0.8777) |
| 120 | **0.71711** (0.6761–0.7548) | **0.90453** (0.8756–0.9273) | **0.83540** (0.8004–0.8653) | **0.71760** (0.6766–0.7553) | **0.90462** (0.8757–0.9274) | **0.83584** (0.8008–0.8657) |
| 200 | **0.72346** (0.6827–0.7609) | **0.88047** (0.8491–0.9060) | **0.81925** (0.7831–0.8505) | **0.72388** (0.6831–0.7613) | **0.88092** (0.8496–0.9064) | **0.81961** (0.7835–0.8508) |
