# Supplementary material for: Non‐parametric combination and related permutation tests for neuroimaging
Source: Hum Brain Mapp. 2016 Feb 5;37(4):1486–511. doi: 10.1002/hbm.23115 (PMC4783210; doi:10.1002/hbm.23115)
Supplement: Supplementary file 1 — Supporting Information [file HBM-37-1486-s001.zip › supporting_information/validity/hist_Tippett_set1.html]

Tippett, Set 1

# Tippett, Set 1

---

|  | Original | | | Modified | | |
| --- | --- | --- | --- | --- | --- | --- |
| N | Gaussian–Gaussian | Weibull–Weibull | Gaussian–Weibull | Gaussian–Gaussian | Weibull–Weibull | Gaussian–Weibull |
| 6 | **0.04775** (0.0323–0.0701) | **0.04797** (0.0324–0.0704) | **0.04785** (0.0323–0.0702) | **0.04962** (0.0338–0.0723) | **0.04928** (0.0335–0.0719) | **0.04956** (0.0337–0.0723) |
| 8 | **0.04881** (0.0331–0.0714) | **0.04843** (0.0328–0.0709) | **0.04794** (0.0324–0.0704) | **0.05087** (0.0348–0.0738) | **0.05008** (0.0342–0.0729) | **0.04976** (0.0339–0.0725) |
| 12 | **0.04788** (0.0324–0.0703) | **0.04820** (0.0326–0.0707) | **0.04923** (0.0335–0.0719) | **0.04957** (0.0337–0.0723) | **0.04985** (0.0340–0.0726) | **0.05046** (0.0345–0.0733) |
| 20 | **0.04791** (0.0324–0.0703) | **0.04823** (0.0327–0.0707) | **0.04845** (0.0328–0.0710) | **0.04972** (0.0339–0.0724) | **0.05019** (0.0342–0.0730) | **0.05022** (0.0343–0.0730) |
| 30 | **0.04814** (0.0326–0.0706) | **0.04792** (0.0324–0.0703) | **0.04891** (0.0332–0.0715) | **0.04965** (0.0338–0.0724) | **0.04953** (0.0337–0.0722) | **0.05047** (0.0345–0.0733) |
| 40 | **0.04883** (0.0331–0.0714) | **0.04942** (0.0336–0.0721) | **0.04786** (0.0324–0.0703) | **0.05029** (0.0343–0.0731) | **0.05093** (0.0349–0.0739) | **0.04930** (0.0335–0.0719) |
| 50 | **0.04836** (0.0328–0.0708) | **0.04897** (0.0333–0.0716) | **0.04817** (0.0326–0.0706) | **0.05005** (0.0341–0.0728) | **0.05031** (0.0343–0.0731) | **0.04954** (0.0337–0.0722) |
| 60 | **0.04888** (0.0332–0.0715) | **0.04843** (0.0328–0.0709) | **0.04769** (0.0322–0.0701) | **0.05023** (0.0343–0.0730) | **0.04985** (0.0340–0.0726) | **0.04915** (0.0334–0.0718) |
| 80 | **0.04899** (0.0333–0.0716) | **0.04816** (0.0326–0.0706) | **0.04881** (0.0331–0.0714) | **0.05044** (0.0344–0.0733) | **0.04962** (0.0338–0.0723) | **0.05032** (0.0344–0.0731) |
| 120 | **0.04860** (0.0330–0.0711) | **0.04829** (0.0327–0.0708) | **0.04781** (0.0323–0.0702) | **0.04988** (0.0340–0.0726) | **0.04973** (0.0339–0.0725) | **0.04951** (0.0337–0.0722) |
| 200 | **0.04905** (0.0333–0.0717) | **0.04824** (0.0327–0.0707) | **0.04846** (0.0328–0.0710) | **0.05054** (0.0345–0.0734) | **0.04976** (0.0339–0.0725) | **0.04981** (0.0339–0.0725) |
