# Supplementary material for: Non‐parametric combination and related permutation tests for neuroimaging
Source: Hum Brain Mapp. 2016 Feb 5;37(4):1486–511. doi: 10.1002/hbm.23115 (PMC4783210; doi:10.1002/hbm.23115)
Supplement: Supplementary file 1 — Supporting Information [file HBM-37-1486-s001.zip › supporting_information/validity/hist_Tippett_set2.html]

Tippett, Set 2

# Tippett, Set 2

---

|  | Original | | | Modified | | |
| --- | --- | --- | --- | --- | --- | --- |
| N | Gaussian–Gaussian | Weibull–Weibull | Gaussian–Weibull | Gaussian–Gaussian | Weibull–Weibull | Gaussian–Weibull |
| 6 | **0.29462** (0.2563–0.3360) | **0.81473** (0.7783–0.8463) | **0.29444** (0.2562–0.3358) | **0.31334** (0.2742–0.3553) | **0.83376** (0.7986–0.8638) | **0.34453** (0.3042–0.3872) |
| 8 | **0.32271** (0.2832–0.3649) | **0.82001** (0.7839–0.8512) | **0.32626** (0.2866–0.3685) | **0.33518** (0.2952–0.3777) | **0.82882** (0.7933–0.8593) | **0.36161** (0.3207–0.4046) |
| 12 | **0.34463** (0.3043–0.3873) | **0.80435** (0.7673–0.8367) | **0.35588** (0.3152–0.3988) | **0.35469** (0.3140–0.3976) | **0.80712** (0.7702–0.8393) | **0.37534** (0.3340–0.4186) |
| 20 | **0.36958** (0.3284–0.4127) | **0.77398** (0.7353–0.8085) | **0.36424** (0.3232–0.4073) | **0.37754** (0.3361–0.4208) | **0.77412** (0.7355–0.8086) | **0.37521** (0.3339–0.4185) |
| 30 | **0.37223** (0.3310–0.4154) | **0.75131** (0.7116–0.7872) | **0.37106** (0.3298–0.4142) | **0.37937** (0.3379–0.4227) | **0.75363** (0.7140–0.7894) | **0.37646** (0.3351–0.4197) |
| 40 | **0.37277** (0.3315–0.4160) | **0.73448** (0.6941–0.7713) | **0.37290** (0.3316–0.4161) | **0.38027** (0.3388–0.4236) | **0.73746** (0.6972–0.7741) | **0.38090** (0.3394–0.4242) |
| 50 | **0.37308** (0.3318–0.4163) | **0.71576** (0.6747–0.7535) | **0.37816** (0.3367–0.4214) | **0.38104** (0.3395–0.4244) | **0.71959** (0.6787–0.7572) | **0.38664** (0.3450–0.4300) |
| 60 | **0.37133** (0.3301–0.4145) | **0.70234** (0.6608–0.7408) | **0.37609** (0.3347–0.4193) | **0.37857** (0.3371–0.4219) | **0.70604** (0.6647–0.7443) | **0.38201** (0.3405–0.4253) |
| 80 | **0.37830** (0.3369–0.4216) | **0.67827** (0.6361–0.7177) | **0.37848** (0.3370–0.4218) | **0.38579** (0.3441–0.4292) | **0.68276** (0.6407–0.7220) | **0.38663** (0.3450–0.4300) |
| 120 | **0.37966** (0.3382–0.4230) | **0.64400** (0.6011–0.6847) | **0.37994** (0.3385–0.4232) | **0.38726** (0.3456–0.4307) | **0.64907** (0.6062–0.6896) | **0.38647** (0.3448–0.4299) |
| 200 | **0.38636** (0.3447–0.4298) | **0.59880** (0.5552–0.6409) | **0.38262** (0.3411–0.4260) | **0.39344** (0.3516–0.4369) | **0.60463** (0.5611–0.6465) | **0.39020** (0.3484–0.4336) |
