# Supplementary material for: Non‐parametric combination and related permutation tests for neuroimaging
Source: Hum Brain Mapp. 2016 Feb 5;37(4):1486–511. doi: 10.1002/hbm.23115 (PMC4783210; doi:10.1002/hbm.23115)
Supplement: Supplementary file 1 — Supporting Information [file HBM-37-1486-s001.zip › supporting_information/validity/hist_Tippett_set3.html]

Tippett, Set 3

# Tippett, Set 3

---

|  | Original | | | Modified | | |
| --- | --- | --- | --- | --- | --- | --- |
| N | Gaussian–Gaussian | Weibull–Weibull | Gaussian–Weibull | Gaussian–Gaussian | Weibull–Weibull | Gaussian–Weibull |
| 6 | **0.29568** (0.2574–0.3371) | **0.81551** (0.7792–0.8471) | **0.81298** (0.7765–0.8447) | **0.31547** (0.2763–0.3575) | **0.83419** (0.7991–0.8642) | **0.82178** (0.7858–0.8528) |
| 8 | **0.32116** (0.2817–0.3633) | **0.81884** (0.7827–0.8501) | **0.82085** (0.7848–0.8520) | **0.33429** (0.2943–0.3768) | **0.82817** (0.7926–0.8587) | **0.82251** (0.7866–0.8535) |
| 12 | **0.34447** (0.3041–0.3872) | **0.80544** (0.7685–0.8378) | **0.81274** (0.7762–0.8445) | **0.35459** (0.3139–0.3975) | **0.80836** (0.7716–0.8405) | **0.81279** (0.7763–0.8445) |
| 20 | **0.36980** (0.3286–0.4130) | **0.77209** (0.7333–0.8067) | **0.78104** (0.7427–0.8151) | **0.37804** (0.3366–0.4213) | **0.77273** (0.7340–0.8073) | **0.78299** (0.7448–0.8169) |
| 30 | **0.37138** (0.3302–0.4146) | **0.75093** (0.7112–0.7868) | **0.75278** (0.7131–0.7886) | **0.37968** (0.3382–0.4230) | **0.75336** (0.7137–0.7891) | **0.75726** (0.7178–0.7928) |
| 40 | **0.37246** (0.3312–0.4157) | **0.73372** (0.6933–0.7706) | **0.73158** (0.6911–0.7686) | **0.37978** (0.3383–0.4231) | **0.73723** (0.6969–0.7739) | **0.73540** (0.6950–0.7722) |
| 50 | **0.37153** (0.3303–0.4147) | **0.71729** (0.6763–0.7550) | **0.71640** (0.6754–0.7541) | **0.37969** (0.3382–0.4230) | **0.72076** (0.6799–0.7583) | **0.72038** (0.6795–0.7579) |
| 60 | **0.37294** (0.3317–0.4161) | **0.70307** (0.6616–0.7414) | **0.70000** (0.6584–0.7385) | **0.38062** (0.3391–0.4239) | **0.70734** (0.6660–0.7455) | **0.70554** (0.6641–0.7438) |
| 80 | **0.38074** (0.3392–0.4241) | **0.67932** (0.6372–0.7187) | **0.67762** (0.6354–0.7171) | **0.38846** (0.3467–0.4319) | **0.68396** (0.6419–0.7232) | **0.68228** (0.6402–0.7216) |
| 120 | **0.37916** (0.3377–0.4225) | **0.64332** (0.6004–0.6841) | **0.64241** (0.5995–0.6832) | **0.38710** (0.3454–0.4305) | **0.64864** (0.6058–0.6892) | **0.64849** (0.6057–0.6891) |
| 200 | **0.38607** (0.3444–0.4295) | **0.59695** (0.5534–0.6390) | **0.60084** (0.5573–0.6428) | **0.39286** (0.3510–0.4363) | **0.60288** (0.5594–0.6448) | **0.60703** (0.5636–0.6489) |
