# Supplementary material for: Non‐parametric combination and related permutation tests for neuroimaging
Source: Hum Brain Mapp. 2016 Feb 5;37(4):1486–511. doi: 10.1002/hbm.23115 (PMC4783210; doi:10.1002/hbm.23115)
Supplement: Supplementary file 1 — Supporting Information [file HBM-37-1486-s001.zip › supporting_information/validity/hist_Tippett_set4.html]

Tippett, Set 4

# Tippett, Set 4

---

|  | Original | | | Modified | | |
| --- | --- | --- | --- | --- | --- | --- |
| N | Gaussian–Gaussian | Weibull–Weibull | Gaussian–Weibull | Gaussian–Gaussian | Weibull–Weibull | Gaussian–Weibull |
| 6 | **0.47326** (0.4299–0.5171) | **0.96189** (0.9413–0.9755) | **0.85790** (0.8246–0.8858) | **0.48963** (0.4461–0.5334) | **0.96392** (0.9437–0.9770) | **0.86378** (0.8309–0.8911) |
| 8 | **0.50998** (0.4663–0.5536) | **0.96335** (0.9430–0.9766) | **0.86870** (0.8363–0.8955) | **0.52241** (0.4786–0.5659) | **0.96406** (0.9439–0.9772) | **0.87056** (0.8383–0.8972) |
| 12 | **0.53967** (0.4958–0.5829) | **0.95770** (0.9363–0.9721) | **0.87008** (0.8378–0.8968) | **0.55008** (0.5063–0.5931) | **0.95748** (0.9360–0.9719) | **0.87120** (0.8390–0.8978) |
| 20 | **0.57682** (0.5331–0.6194) | **0.94295** (0.9190–0.9601) | **0.85055** (0.8166–0.8791) | **0.58481** (0.5411–0.6272) | **0.94309** (0.9192–0.9602) | **0.85198** (0.8182–0.8804) |
| 30 | **0.58099** (0.5373–0.6235) | **0.93217** (0.9067–0.9511) | **0.83359** (0.7984–0.8637) | **0.58892** (0.5453–0.6312) | **0.93267** (0.9072–0.9515) | **0.83665** (0.8017–0.8665) |
| 40 | **0.58297** (0.5393–0.6254) | **0.92391** (0.8973–0.9441) | **0.82090** (0.7849–0.8520) | **0.59116** (0.5475–0.6334) | **0.92506** (0.8986–0.9450) | **0.82422** (0.7884–0.8551) |
| 50 | **0.58343** (0.5397–0.6258) | **0.91437** (0.8866–0.9358) | **0.81179** (0.7752–0.8436) | **0.59186** (0.5482–0.6341) | **0.91598** (0.8884–0.9372) | **0.81571** (0.7794–0.8472) |
| 60 | **0.58324** (0.5395–0.6257) | **0.90520** (0.8764–0.9279) | **0.80243** (0.7653–0.8350) | **0.59111** (0.5475–0.6334) | **0.90689** (0.8782–0.9294) | **0.80668** (0.7698–0.8389) |
| 80 | **0.59283** (0.5492–0.6350) | **0.89045** (0.8600–0.9149) | **0.78784** (0.7499–0.8214) | **0.60101** (0.5575–0.6430) | **0.89276** (0.8626–0.9169) | **0.79266** (0.7550–0.8259) |
| 120 | **0.59436** (0.5508–0.6365) | **0.86506** (0.8323–0.8922) | **0.76608** (0.7270–0.8011) | **0.60268** (0.5592–0.6446) | **0.86808** (0.8356–0.8950) | **0.77148** (0.7327–0.8061) |
| 200 | **0.60212** (0.5586–0.6441) | **0.82889** (0.7934–0.8594) | **0.74066** (0.7005–0.7771) | **0.60961** (0.5662–0.6514) | **0.83349** (0.7983–0.8636) | **0.74658** (0.7067–0.7827) |
